# Inner and inter population structure construction of Chinese Jiangsu Han population based on Y23 STR system

**DOI:** 10.1371/journal.pone.0180921

**Published:** 2017-07-13

**Authors:** Huipin Wang, Huajie Ba, Chun Yang, Jianqiu Zhang, Yunchun Tai

**Affiliations:** 1 Zhongshan City People's Hospital, Zhongshan, Guangdong, P. R., China; 2 DNA Laboratory, Public Security Bureau of Changzhou, Changzhou, Jiangsu, P. R., China; 3 Department of Psychiatry, Psychiatry Center of Chinese People’s Liberation Army, No. 102 Hospital of People’s Liberation Army, Changzhou, Jiangsu, P. R., China; 4 DNA Laboratory, Public Security Bureau of Yangzhou, Yangzhou, Jiangsu, P. R., China; 5 School of Forensic Medicine, Southern Medical University, Guangzhou, Guangdong, P. R., China; National Cheng Kung University, TAIWAN

## Abstract

In this study, we analyzed the genetic polymorphisms of 23 Y-STR loci from PowerPlex^®^ Y23 system in 916 unrelated healthy male individuals from Chinese Jiangsu Han, and observed 912 different haplotypes including 908 unique haplotypes and 4 duplicate haplotypes. The haplotype diversity reached 0.99999 and the discrimination capacity and match probability were 0.9956 and 0.0011, respectively. The gene diversity values ranged from 0.3942 at DYS438 to 0.9607 at DYS385a/b. Population differentiation within 10 Jiangsu Han subpopulations were evaluated by R_ST_ values and visualized in Neighbor-Joining trees and Multi-Dimensional Scaling plots as well as population relationships between the Jiangsu Han population and other 18 Eastern Asian populations. Such results indicated that the 23 Y-STR loci were highly polymorphic in Jiangsu Han population and played crucial roles in forensic application as well as population genetics. For the first time, we reported the genetic diversity of male lineages in Jiangsu Han population at a high-resolution level of 23 Y-STR set and consequently contributed to familial searching, offender tracking, and anthropology analysis of Jiangsu Han population.

## Introduction

Genetic markers derived from Y chromosome independent of recurrent mutations or recombination [1], play special roles in uncovering genetic structure [2,3] and inferring human dispersal and important migration time range [4,5]. In Y chromosomes, genetic variations are inherited from father to son paternally. Thus, Y chromosome could reflect the gene flows and genetic differentiation of male lineages [6]. In addition, genetic markers in Y chromosome have smaller effective population size compared to those in autosomes. The advancement of new sequencing technologies and more attention paid to human genome project provided abundant genetic markers in Y-DNA [3,7,8]. Among the various markers in Y chromosome, short tandem repeats (STRs), are widely employed due to their hypervariability and hypermutability, thus it is available to perform multiplex amplifications at Y-STRs. So far, several commercial kits have been developed, such as PowerPlex Y (Promega, USA) [9], Yfiler™ Plus (Thermo Fisher Scientific, USA) [10], and PowerPlex^®^ Y23 [11]. The population data of Y-STRs now is being enriched to explore the genetic diversity of global male lineages [12–15]. Thus far, Y-STR Haplotype Reference Database (YHRD) has received a large-scale Y-STR data to generate reliable Y-STR haplotype frequencies for forensic purposes, as well as to assess male lineage stratification for population genetics studies (https://www.yhrd.org). In investigating authorities, Y-STR haplotype analysis serves to track male suspects in potential pedigrees sharing identical or highly similar Y-STR haplotypes to crime evidence [16].

**Fig 1:**
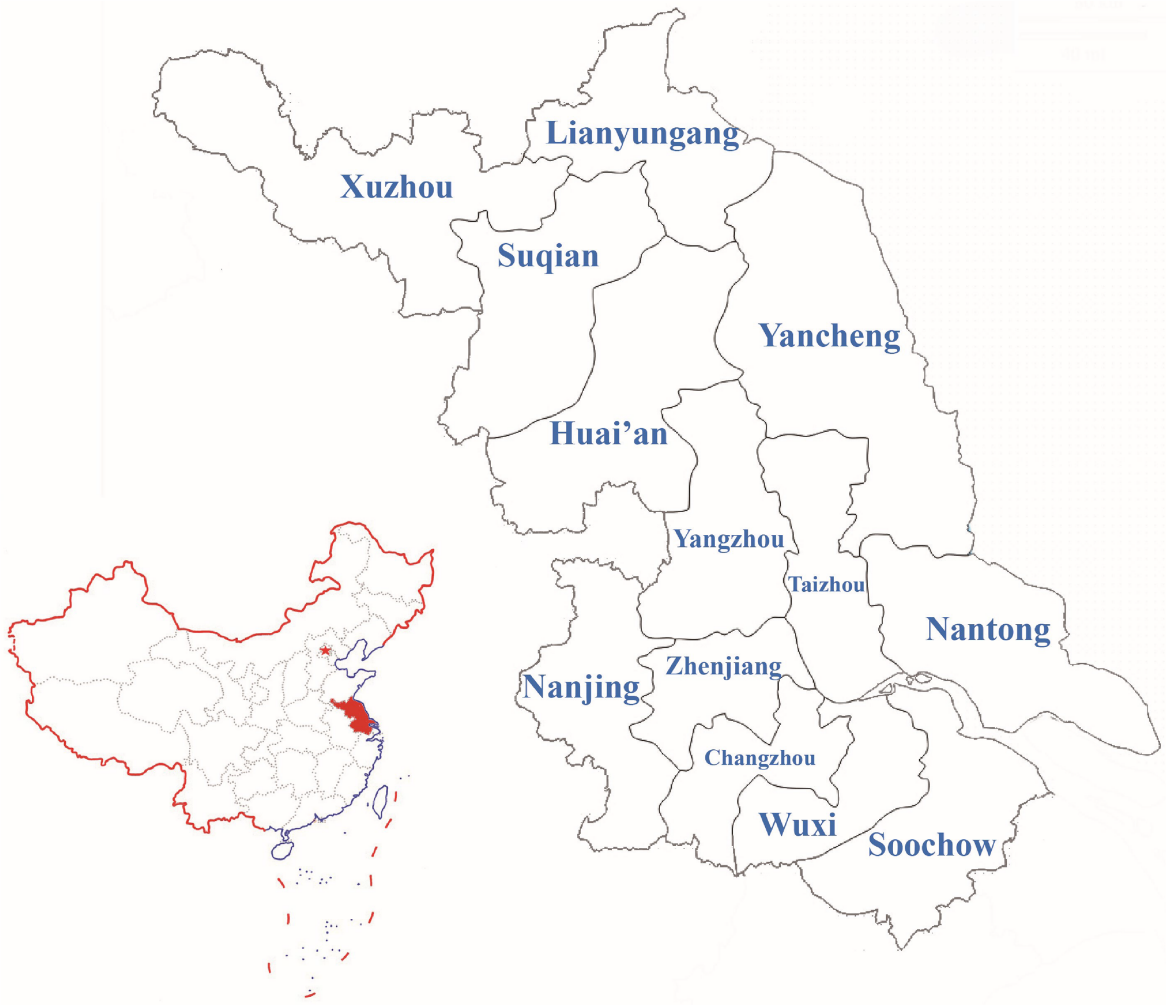
The geographic location of Jiangsu province in the scale of China. The names of 13 prefecture-level cities were labelled in the map. The red five-pointed star represented Beijing City.

Jiangsu, an eastern coastal province of China, borders Shandong province in north, Anhui province in west, Zhejiang province in south, and Shanghai City and the Yellow Sea in east. It is the most densely populated of 23 provinces of China. According to 2010 population census, the number of whole population in Jiangsu province is 78,659,903, ranking the 5^th^ of all China administrative regions. The peopling of Jiangsu province is mostly Han population (99.6%), followed by Hui ethnic minority (0.2%) [17]. The linguistic affiliation of Jiangsu Han is a sub-branch of Sino-Tibetan language. The culture of Jiangsu province originated in Zhou Dynasty (1046–256 BC), which showed regional differences. It was subdivided into three cultural regions, such as Wu cultural region in the south, Chuhan cultural region in the north and Jianghuai cultural region in the middle [18]. On the prefecture level, Jiangsu province is classified into 13 prefecture-level cities, including Xuzhou, Suqian, Lianyungang, Yancheng, and Huai’an in the north, Soochow, Wuxi, Changzhou, Zhenjiang, and Nanjing in the south, and Yangzhou, Taizhou, and Nantong in the middle (Fig 1). The complex culture background and extremely dense population require up-to-date Y-DNA profiling not only to provide information for forensic familial search, but also to study the genetic kinship between Jiangsu Han population and other populations worldwide. To our best knowledge, the most recent research on Y-STRs’ diversities of Jiangsu province was in 2015, that Li and collaborators performed the analysis on population data of 17 Y-STRs of 284 individuals [19].

To investigate the restricted regional distribution of Y-STR in Jiangsu Han population, we typed 916 male individuals from 13 prefecture cities by PowerPlex^®^ Y23 system. In this research, we studied the inner-population differentiation, followed by inter-population differentiation by comparisons with the population data with 18 Eastern Asian populations [12]. The enrichment patterns of Y chromosome haplotype data and the understanding of Jiangsu Han’s genetic structure are improved generally.

## Materials and methods

### Samples and DNA extraction

A total of 916 unrelated healthy male individuals of Han ancestry provided blood samples with informed consent, including 207 samples from Changzhou, 147 from Xuzhou, 111 from Suqian, 98 from Wuxi, 71 from Lianyungang, 62 from Yancheng, 55 from Huai’an, 47 from Soochow, 41 from Nanjing, 39 from Nantong, 17 from Zhenjiang, 14 from Yangzhou, and 7 from Taizhou. All individuals come from Chinese Jiangsu Han population and reside locally at least three generations. Chelex-100 protocol [20] was adopted to extract genomic DNA. The study was conducted in accordance to the human and ethical research principles of Zhongshan City People’s Hospital and approved by the ethics committee in Zhongshan City People’s Hospital.

### STR genotyping

Y-STR was typed for the PowerPlex^®^ Y23 System (Promega, USA) with GeneAmp PCR system 9700 (Thermofisher Scientific, Waltham, MA, USA). Amplified DNAs were separated on ABI3130XL DNA Genetic Analyzer (Thermofisher Scientific, Waltham, MA, USA) and analyzed using the GeneMapper ID-X software (Thermofisher Scientific, Waltham, MA, USA).

### Statistical analysis

After direct count of the allele frequencies and haplotype by Arlequin 3.0 [21], we calculated gene diversity (GD) following Nei [22,23]. We also calculated several representative forensic parameters, such as haplotype diversity (HD), discrimination capacity (DC), and match probability (MP) according Sabine et al [24]. DYS385a/b, a multi-copy locus, was analyzed as combined haplotypes. And we got the allele of DYS389II by the subtraction of DYS389I. The popular combination of computational R_ST_ values which referred to the excess similarity among alleles chosen randomly within the subgroup relative to the entire group [25] and Multidimensional Scaling plot (MDS) [26] was adopted as YHRD analyzed and many publications conducted [15,19,27–32]. Linkage patterns and Analysis of Molecular Variance (AMOVA) which generated the pairwise R_ST_ values and according significant values were performed using Arlequin 3.0 based on detailed haplotype information of eligible individuals (samples with the null, intermediate alleles, and copy-number variations were removed). Neighbor-Joining (N-J) tree was depicted and visualized by Mega 6.0 [33] as others conducted [31,34]. Based on the matrix of R_ST_ values, we illustrated MDS as YHRD recommended and achieved values of initial stress by employing “MASS” package (https://cran.r-project.org/web/packages/MASS/index.html).

### Quality control

Our laboratory’s ability to Y-STR genotyping was accredited by YHRD through Y-STR haplotyping quality test. The population accession number is YA004256 (https://www.yhrd.org). As modified by YHRD, the null, intermediate alleles, and copy-number variations were removed in the analysis of R_ST_. The study was carried out following ISFG recommendations with respect to DNA polymorphisms as Schneider described [35].

## Result and discussion

### Haplotypes and forensic parameters

After statistical analysis, we found 912 different haplotypes in total, comprising 908 singletons (99.6%) and 4 duplicates (0.4%). Also, two null allele was observed at the DYS448 and DYS456, which have been detected in several previously publications (S1 Table, available at the public repository “Figshare.com”, https://doi.org/10.6084/m9.figshare.5150128) [12,19,36,37]. As well, we presented the geographic locations of all samples in S2 Table (available at the public repository “Figshare.com”, https://doi.org/10.6084/m9.figshare.5150128). The HD was 0.9999952, which indicates high level of polymorphism. The values of MP and DC were 0.0011 and 0.9956, respectively, exerting admirable roles in practical application of PowerPlex^®^ Y23 System in Jiangsu Han population. Besides, we found 3 microvariants namely 16.1 at DYS458, 16.2 at DYS385 and 19.2 at DYS448, all have been covered in YHRD database after the validation option provided.

S3 and S4 Tables displayed allele frequencies and each GD value of 21 single-copy Y-STR loci and the multi-copy DYS385 locus. The size of alleles in each Y-STR locus ranged from 6 at DYS391 to 33 at DYS389II. Additionally, allele number ranged from 5 at DYS437 to 13 at DYS458 and 76 different allele combinations were observed at DYS385a/b, slightly higher than 53 in 12 worldwide populations [31] and 69 in four U.S. populations [32]. Except DYS391, DYS438 and DYS437, all loci got GD values higher than 0.5. The highest GD value was 0.9607 at DYS385, which was a multi-copy locus taken to be consisted of 76 various alleles, and the lowest was 0.3942 at DYS438 with the highest allele frequency of 0.7533 at allele 10. Actually, distributions of allele frequencies at the panel of 23 Y-STRs varied in different populations. At DYS438, the most frequent allele was 11 in Tibetan (F = 0.7016) [38] and Bangladeshi (F = 0.5330) [39]. However, the most frequent allele at DYS438 was still 10 in Zhuang (F = 0.6880) [40], Mongolian (F = 0.7483) [41], Miao (F = 0.8095) [42], South Korean (F = 0.4967) [43], Liaoning Han (F = 0.7016) [34], Chengdu Han (F = 0.6861) [44], and Hunan Han (F = 0.7323) [30]. In addition, similar to Jiangsu Han population, the frequency of allele 10 at DYS438 was also the highest among targeted Y-STRs in Liaoning Han, Guangdong Han, Hunan Han, and South Korean. However, the underlying reason was unclear and this genetic phenomenon may have connections with population bottleneck in population genetics, thus leading to genetic drift [45]. Likewise, this could also be induced by the founder effect outlined by Ernst Mayr [46]. From the new-established groups’ perspective, the genetic diversities of offspring were massively dependent of several emigrants from a large population. The randomization thus probably led to that the most abundant alleles were different among populations. Excluding DYS385, the maximum level of GD was DYS458 at 0.8233, which had the most alleles in single-copy locus. The distribution of various forensic parameters demonstrated that Jiangsu Han population was genetically of high diversity.

### Linkage disequilibrium analysis

**Fig 2:**
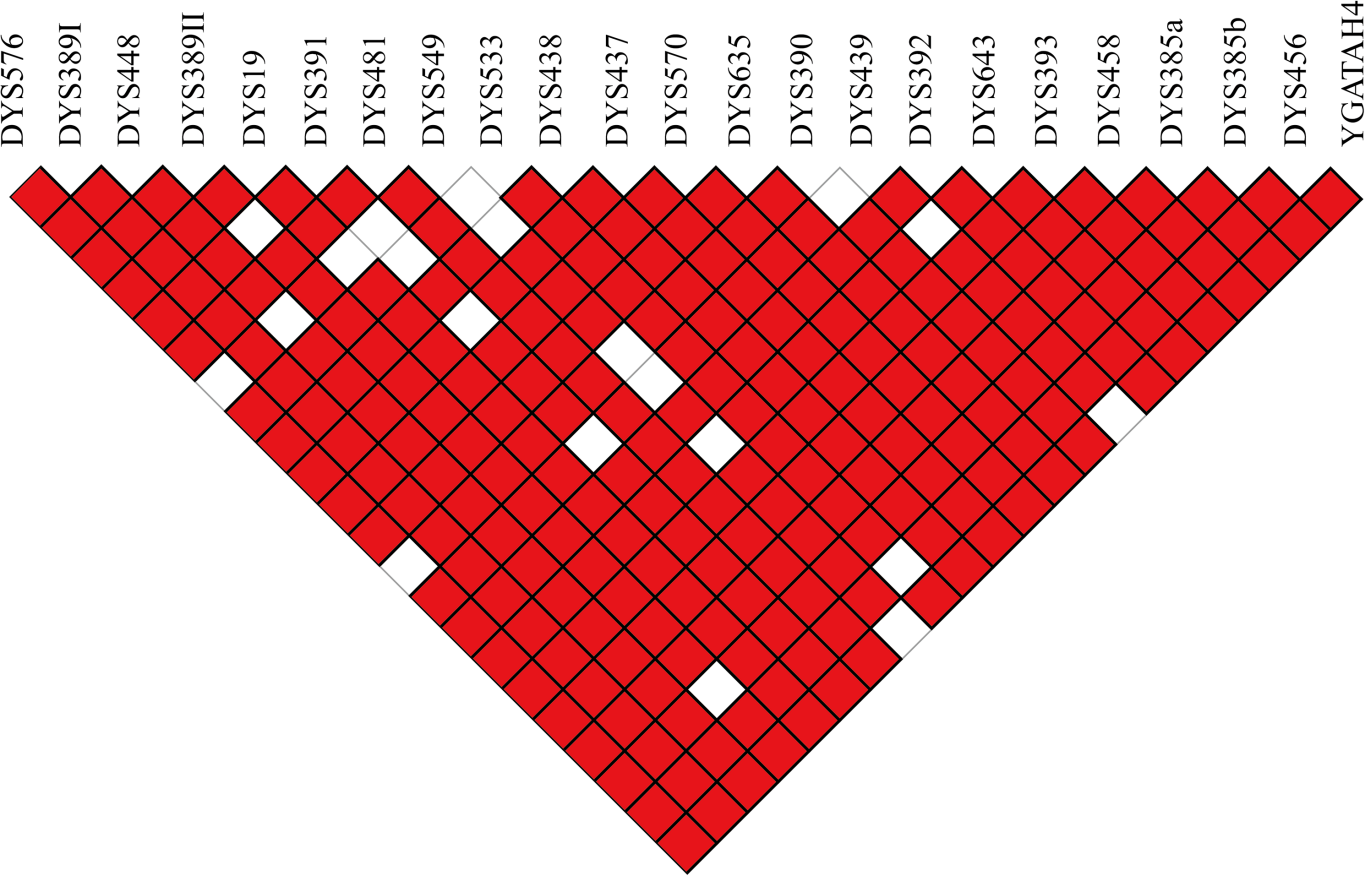
The LD analysis among 23 Y-STR loci. The red area encircled by thick black line represented significantly strong linkage relationship.

LD analysis of 23 Y-STRs in Jiangsu Han was performed in Fig 2. Among 253 pairwise comparisons, 233 showed the status of LD (red area encircled by bold black boxes), accounting for 92.1%. It showed high level of LD patterns among Jiangsu Han population, explained by previous genomic studies that 95% of Y chromosome, so-called male-specific region (MSY) where X-Y crossing was absent, was a non-recombining region [47].

### Inner-population differentiation

**Fig 3:**
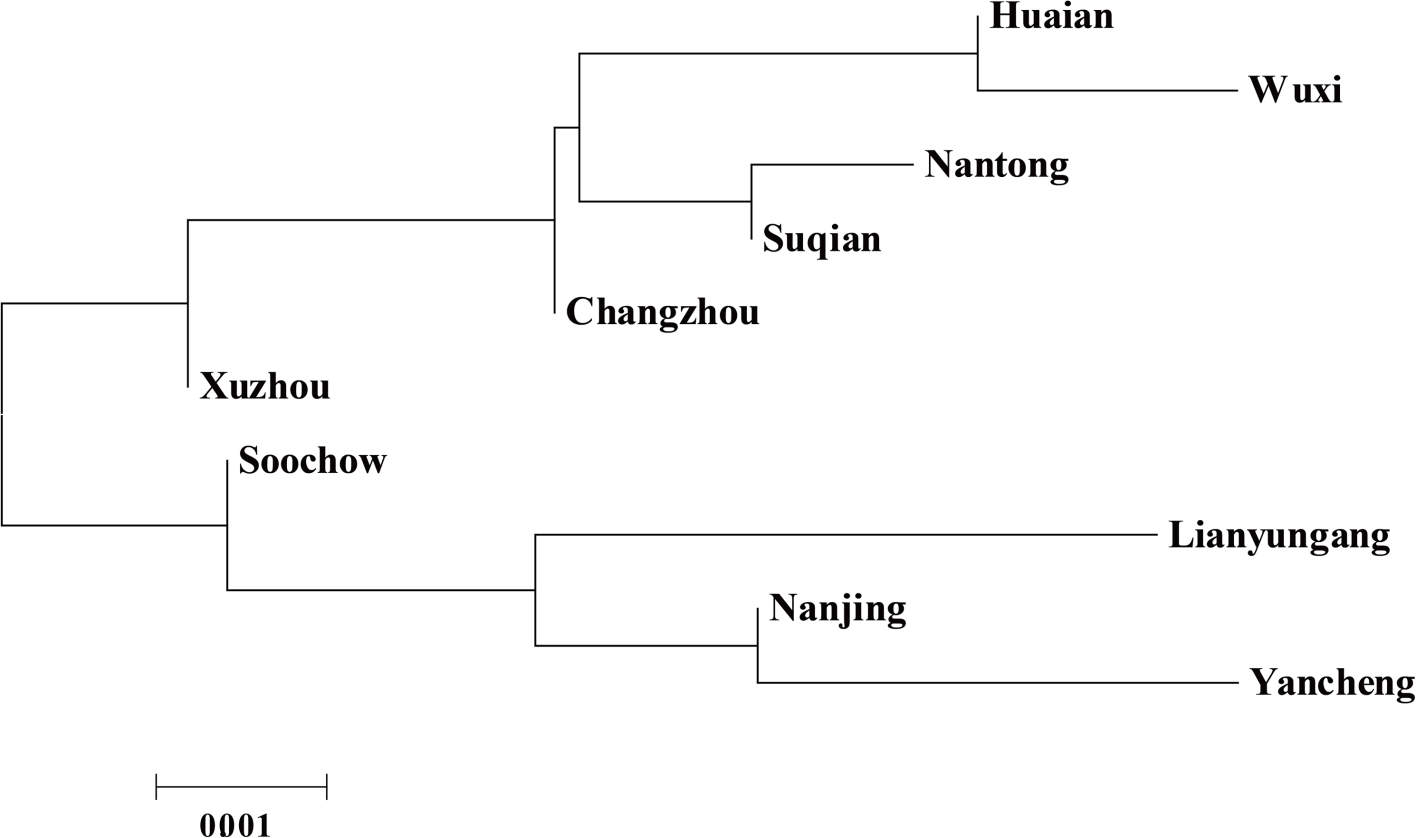
A Neighbor-Joining (N-J) tree composed of all 10 prefecture-level Han populations from Jiangsu province. The result of interior branch test to assess the reliability of NJ reconstruction and the ruler were signed in the figure (the sum of branch length = 0.01566328).

The values of pairwise R_ST_ genetic distances and significances among 10 prefecture-level divisions of Jiangsu province were shown in Table 1. Zhenjiang, Yangzhou, and Taizhou Han sub-populations were excluded because of their small sample sizes which could not reflect the comprehensive genetic milieu. There were little significant differences existed among these 10 prefectures. The lowest genetic distance was between Xuzhou Han and Nantong Han (R_ST_ = 0.0000, P = 0.1441), while the highest genetic distance was of significance between Lianyungang Han and Nantong Han (R_ST_ = 0.0292, P = 0.0451). In addition, 4 more significant differences were found between Xuzhou and Wuxi Hans (R_ST_ = 0.0101, P = 0.0000), Yancheng and Wuxi Hans (R_ST_ = 0.0153, P = 0.0360), Lianyungang and Wuxi Hans (R_ST_ = 0.0128, P = 0.0360), and Yancheng and Nantong Hans (R_ST_ = 0.0269, P = 0.0180). After Bonferroni Correction (P < 0.05/55 ≈ 0.0009), only the comparison between Wuxi Han and Xuzhou Han was found to be remarkable. In the map of Jiangsu province (Fig 1), Xuzhou Han and Wuxi Han were geographically far apart, respectively located in the farthest north and south part of Jiangsu, which contributed to the significant genetic differentiation. Fig 3 showed the optimal N-J tree of 10 prefecture-level divisions of Jiangsu with the sum of branch length (SBL) = 0.01566328. In general, the 10 Han populations clustered in the N-J tree were not in accordance with the geographical distribution. The phylogenetic reconstruction indicated the close relationships between Huaian and Wuxi Hans, Nantong and Suqian Hans. Nanjing Han was clustered with Yancheng Han first, then with Lianyungang Han, followed by Soochow Han. The Han groups from south were close to various Han groups from north Jiangsu province, demonstrating the population homogeneity among Jiangsu Han subgroups.

**Table 1:** The pairwise R_ST_ and p values among various Han populations from 10 prefecture-level cities in Jiangsu province excluding Zhenjiang, Yangzhou, and Taizhou Hans.

	Changzhou	Huaian	Lianyungang	Nanjing	Nantong	Soochow	Wuxi	Suqian	Xuzhou	Yancheng
Changzhou	#	0.3423	0.7748	0.2973	0.3153	0.4865	0.1892	0.4685	0.5496	0.8108
Huaian	-0.0017	#	0.0541	0.9369	0.6757	0.5586	0.4234	0.6847	0.2072	0.1081
Lianyungang	-0.0065	0.0118	#	0.2523	**0.0451**	0.3694	**0.0360**	0.7568	0.3964	0.9009
Nanjing	-0.0062	-0.0113	0.0046	#	0.3243	0.6577	0.5225	0.5676	0.2252	0.3153
Nantong	-0.0004	-0.0057	0.0292	0.0036	#	0.5135	0.6216	0.3153	0.1441	**0.0180**
Soochow	-0.0043	-0.0045	0.0025	-0.0081	-0.0044	#	0.6577	0.7117	0.2162	0.3063
Wuxi	0.0035	-0.0014	0.0128	-0.0035	-0.0043	-0.0042	#	0.1261	0.0000^*^	**0.0360**
Suqian	-0.0009	-0.0043	-0.0028	-0.0065	0.0006	-0.0060	0.0031	#	0.1532	0.7478
Xuzhou	-0.0002	-0.0009	-0.0021	-0.0059	0.0000	-0.0017	0.0101	0.0021	#	0.6577
Yancheng	-0.0076	0.0097	-0.0073	0.0020	0.0269	0.0025	0.0153	-0.0041	-0.0054	#

The left area of the diagonal line showed R_ST_ genetic distances; the right showed corresponding P values.

P < 0.05 was indicated in bold format; Genetic distance of significance was in underlined format.

“*” represented significance after Bonferroni Correction.

**Fig 4:**
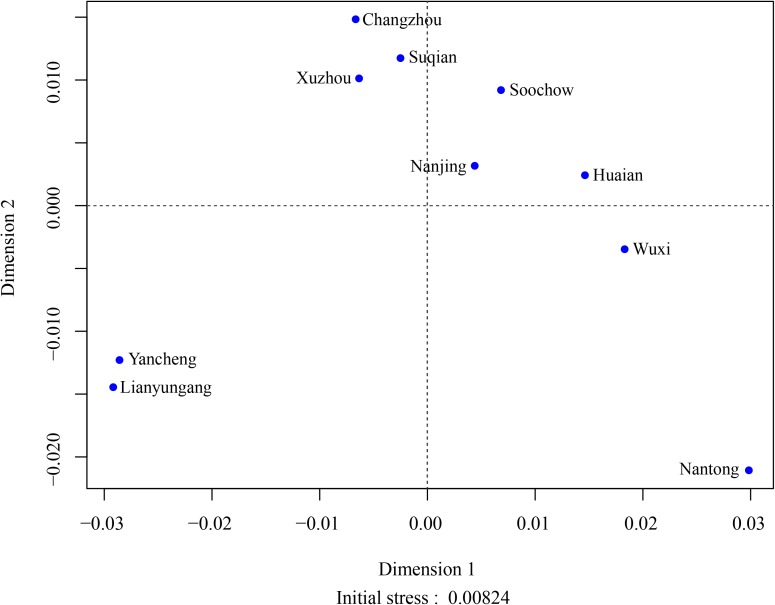
A Multi-Dimensional Scaling (MDS) plot constructed according to the R_ST_ genetic distance matrix among 10 Han populations in Jiangsu province. The initial stress was 0.00824.

For further validation, we drew the MDS plot whose stress reached 0.00824, indicating the perfect configuration (Fig 4). We observed representative genetic relationships between Yancheng and Lianyungang Hans, Nantong and Wuxi Hans, which were similar to N-J tree. Besides, Wuxi, Soochow, Changzhou, and Nanjing Hans were located dispersedly in the plot. The results of MDS conformed to that of N-J reconstruction. Changzhou, Lianyungang, and Nantong Han populations were observed as three outlier Han populations along both Dimensions 1 and 2, which were all situated at the margin areas of Jiangsu province (Fig 1) and had frequent gene exchanges with populations from neighbor provinces.

We associated the genetic mixture of southern and northern Jiangsu Han with the fact that southern Jiangsu absorbed many floating people derived from northern Jiangsu due to its high level of economic development, living conditions, education, and means of transportation. The genetic characteristics of 10 Han sub-populations in Jiangsu province covered 2 main aspects: one was the significant difference between southern and northern Han populations in Jiangsu; another was the modern living mode consistently influenced the population structure of various Han populations in southern Jiangsu, in addition, the frequent crosstalk among Jiangsu Han populations pulled them together from the view of gene flow.

To the best of our knowledge, only the population data and corresponding genetic background of Huai’an Han [48], and Nantong Han [19,49] have been collected and analyzed in the level of subpopulation. Our efforts to establish the 23 Y-STRs database of 10 Jiangsu Han populations illustrated the genetically gene exchange comprehensively and would provide references for investigating authorities to target the potential pedigree potently. Attentions have been paid to demonstrating the inner-population structure utilizing Y-STRs as genetic markers for the purpose of inferring the most likely geographic origin of a haplotype at Y chromosome. In 2014, Zeng et al. [27] discussed the Taiwanese origin of the Austronesian expansion within 9 major aboriginal tribes inhabiting Taiwan. And in 2016, Ulises et al. [29] reported a comprehensive study on population structure of 13 Argentine provinces. In this research, we initially focused on the interactive relationships among 10 inner-Jiangsu Han groups and point out its values on deducing the historical evolution and inferring the potential population trend nowadays. More importantly, our findings contributed to providing the investigating authorities with potential mixed relationships within Jiangsu province when solving cross-region crimes.

### Inter-population differentiation

Table 2 displayed the pairwise R_ST_ and P values among Jiangsu Han population and other 18 Eastern Asian populations, consisting of Beijing Han, Chengdu Han, Shantou Han, Singapore Han, Xuanwei Han, Southern Han, South Korean, Dai, Bai, Kinh, Gunma Japanese, Ibaraki Japanese, Shizuoka Japanese, Tokyo Japanese, Filipino, Luzon Filipino, Singapore Indian, and Singapore Malaysian, all chosen from the global analysis on haplotype diversities of 23 Y-STRs [12]. It showed that the pairwise R_ST_ values between the Jiangsu Han population and other 18 populations were commonly larger than that of inner Jiangsu Han population and the number of significances, which less than 0.0003 (0.05/171, Bonferroni correction), were much larger, indicating greater genetic distances than that of inner Jiangsu Han population. Jiangsu Han population was prominently different from Ibaraki Japanese (R_ST_ = 0.0341, P = 0.0000), Luzon Filipino (R_ST_ = 0.0095, P = 0.0000), Shantou Han (R_ST_ = 0.0906, P = 0.0000), Singapore Han (R_ST_ = 0.0410 P = 0.0000), Singapore Indian (R_ST_ = 0.1136, P = 0.0000), Singapore Malaysian (R_ST_ = 0.0799, P = 0.0000), and South Korean (R_ST_ = 0.0182, P = 0.0000). The remaining 11 populations were all genetically close to Jiangsu Han population, including 4 Chinese groups of Han ancestry. Dramatically, Singapore Indian had the greatest genetic distance with Jiangsu Han, in accordance with geographic location.

**Table 2:** The pairwise R_ST_ and p values among Jiangsu Han population and other 18 Eastern Asian populations.

Population	Beijing Han	Chengdu Han	Gunma Japanese	Kinh	Ibaraki Japanese	Luzon Filipino	Filipino	Shantou Han	Shizuoka Japanese	Singapore Han	Singapore Indian	Singapore Malaysian	South Korean	Southern Han	Tokyo Japan	Dai	Xuanwei Han	Bai	Jiangsu Han
Beijing Han	#	0.9369	0.1622	0.3784	0.0000*	**0.0180**	0.2342	0.0000*	0.0721	**0.0090**	0.0000*	0.0000*	0.0000*	0.2883	0.1171	0.3063	0.3333	0.0000*	0.1622
Chengdu Han	-0.0048	#	0.4865	0.9099	0.0541	0.1892	0.5225	**0.0270**	0.0991	0.2252	0.1441	0.0720	0.0721	0.9279	0.3423	0.5856	0.1171	0.0000*	**0.0451**
Gunma Japanese	0.0084	-0.0023	#	0.0000*	0.8739	0.4144	0.4505	0.0721	0.3694	0.1982	0.3153	0.3874	0.2973	0.0000*	0.4234	0.0000*	0.0000*	0.2072	0.1351
Kinh	-0.0071	-0.0102	0.0978	#	0.0631	0.8559	0.9640	0.1622	0.0000*	0.4955	0.2342	0.4414	0.2613	0.1532	0.0000*	0.0000*	0.1441	0.3243	0.1712
Ibaraki Japanese	0.0164	0.0095	-0.0102	0.0039	#	0.0000*	0.0000*	0.0000*	0.4955	**0.0090**	**0.0090**	0.0000*	0.3243	0.2432	0.5766	0.0811	0.0000*	0.0000*	0.0000*
Luzon Filipino	0.0049	0.0040	-0.0045	-0.0076	0.0133	#	0.8829	0.0000*	0.2613	0.0000*	0.0000*	0.0000*	**0.0090**	0.8469	0.2793	0.3063	0.2523	0.0000*	0.0000*
Filipino	0.0018	-0.0014	-0.0061	-0.0097	0.0110	-0.0024	#	0.0000*	0.3153	**0.0090**	**0.0270**	**0.0270**	0.0000*	0.7658	0.3874	0.5946	0.3694	0.0000*	**0.0180**
Shantou Han	0.0418	0.0195	0.0329	0.0185	0.0380	0.0370	0.0299	#	**0.0090**	0.5856	**0.0090**	**0.0360**	0.0000*	0.1081	**0.0360**	**0.0090**	0.0000*	0.0901	0.0000*
Shizuoka Japanese	0.0150	0.0053	-0.0015	0.0905	-0.0032	0.0016	0.0019	0.0372	#	0.0901	0.1441	0.1892	0.3063	0.0000*	0.6757	0.0000*	0.0000*	0.0991	**0.0270**
Singapore Han	0.0185	0.0062	0.0199	0.0035	0.0211	0.0160	0.0132	-0.0051	0.0231	#	0.0000*	0.0721	0.0000*	0.2523	**0.0270**	0.1532	0.0721	0.0541	0.0000*
Singapore Indian	0.0435	0.0203	0.0060	0.0102	0.0332	0.0451	0.0258	0.0303	0.0165	0.0245	#	0.6126	0.0000*	0.5766	0.2342	**0.0180**	**0.0451**	0.0000*	0.0000*
Singapore Malaysian	0.0302	0.0128	-0.0002	0.0025	0.0211	0.0287	0.0165	0.0149	0.0082	0.0106	-0.0074	#	0.0000*	0.5586	0.1261	**0.0180**	**0.0090**	**0.0180**	0.0000*
South Korean	0.0082	0.0050	-0.0031	0.0029	0.0021	0.0104	0.0092	0.0460	0.0011	0.0232	0.0552	0.0378	#	0.2973	0.2883	**0.0090**	0.0541	0.0000*	0.0000*
Southern Han	-0.0111	-0.0174	0.0928	0.0124	-0.0026	-0.0118	-0.0137	0.0119	0.0800	-0.0008	0.0017	-0.0048	-0.0064	#	**0.0090**	0.0000*	0.0000*	0.5946	**0.0451**
Tokyo Japanese	0.0123	0.0024	-0.0026	0.0776	-0.0039	0.0005	0.0005	0.0373	-0.0053	0.0233	0.0167	0.0085	0.0009	0.0643	#	0.0000*	0.0000*	0.0811	0.0541
Dai	0.0017	-0.0007	0.1842	0.0335	0.0177	0.0008	-0.0016	0.0351	0.1577	0.0171	0.0300	0.0189	0.0129	0.0900	0.1397	#	0.0000*	0.0000*	0.1802
Xuanwei Han	0.0008	0.0025	0.1126	0.0076	0.0170	0.0013	0.0007	0.0430	0.1015	0.0196	0.0349	0.0216	0.0110	0.0360	0.1002	0.0240	#	0.0000*	0.2793
Bai	0.0335	0.0142	0.0037	0.0026	0.0197	0.0464	0.0228	0.0075	0.0122	0.0079	0.0235	0.0143	0.0332	-0.0043	0.0111	0.0183	0.0236	#	0.0000*
Jiangsu Han	0.0007	0.0082	0.0281	-0.0017	0.0341	0.0095	0.0107	0.0906	0.0304	0.0410	0.1136	0.0799	0.0182	0.0024	0.0302	0.0082	0.0000	0.0930	#

The left area of the diagonal line showed R_ST_ genetic distances; the right showed corresponding P values.

P < 0.05 was indicated in bold format; Genetic distance of significance was in underlined format.

“*” represented significance after Bonferroni correction.

**Fig 5:**
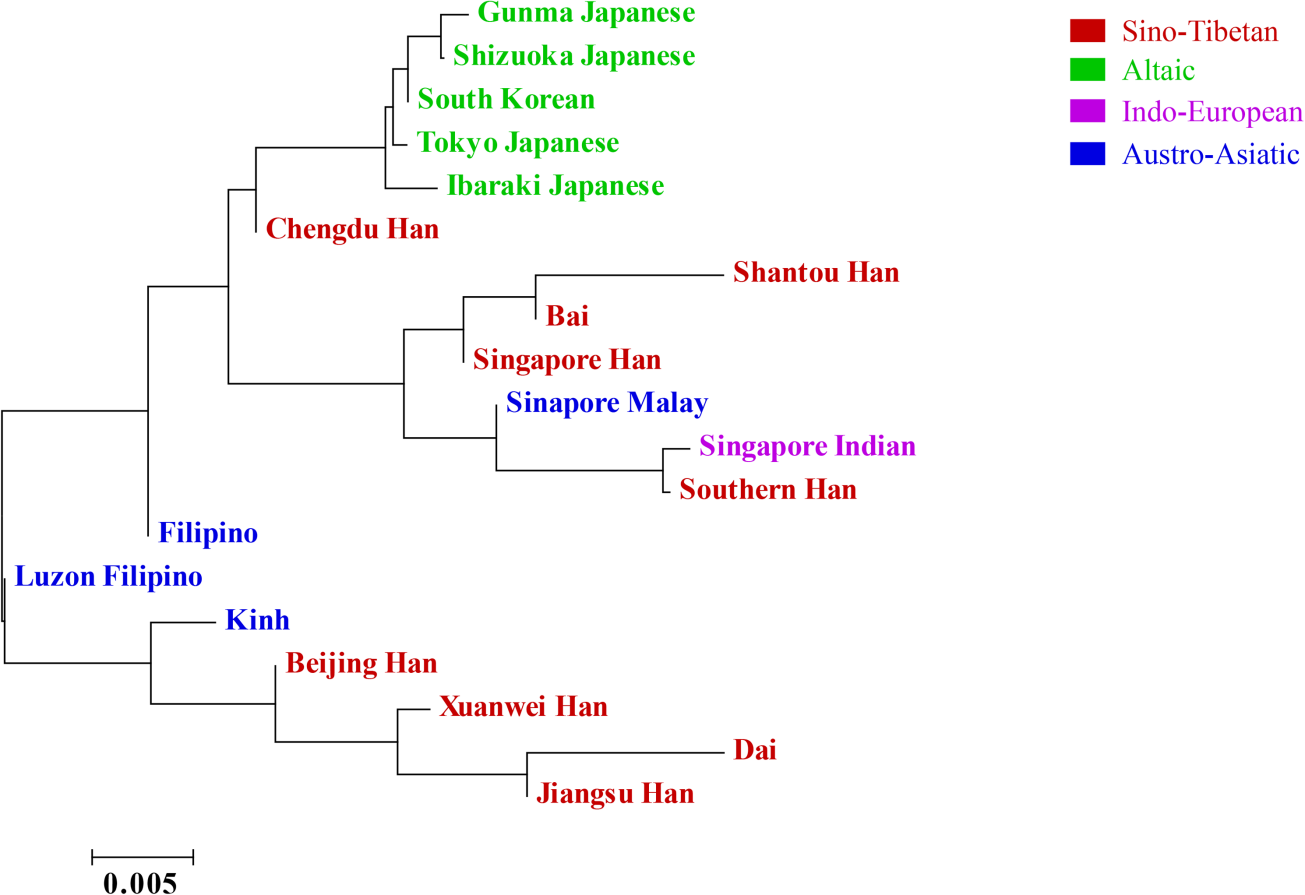
A Neighbor-Joining (N-J) tree composed of Jiangsu Han population and other 18 Eastern Asian populations, including Beijing Han, Chengdu Han, Shantou Han, Singapore Han, Xuanwei Han, Southern Han, South Korean, Dai, Bai, Kinh, Gunma Japanese, Ibaraki Japanese, Shizuoka Japanese, Tokyo Japanese, Filipino, Luzon Filipino, Singapore Indian, and Singapore Malaysian. The result of interior branch test to assess the reliability of NJ reconstruction and the ruler were signed in the figure (the sum of branch length = 0.07132081). The language affiliations of these 19 populations were also indicated in different colors (Red: Sino-Tibetan; Green: Altaic; Purple: Indo-European; Blue: Austro-Asiatic).

**Fig 6:**
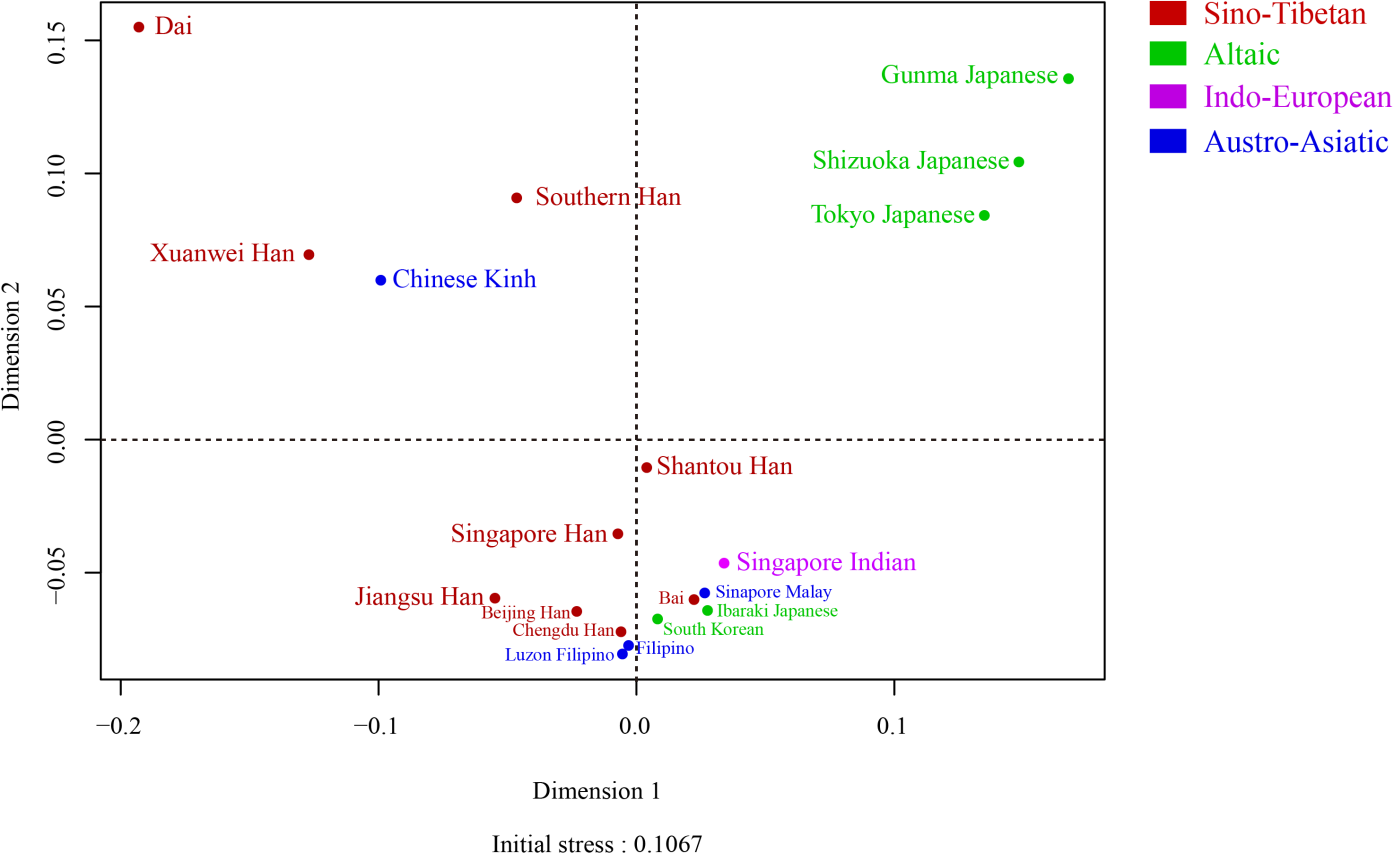
A Multi-Dimensional Scaling (MDS) plot constructed according to the RST genetic distance matrix among Jiangsu Han populations and other 18 populations in East Asia scale. The initial stress was 0.1067. The language affiliations of these 19 populations were also indicated in different colors (Red: Sino-Tibetan; Green: Altaic; Purple: Indo-European; Blue: Austro-Asiatic).

In addition, we performed N-J tree (Fig 5) and MDS plot (Fig 6) of above-mentioned populations labelled with language affinities. The result of branch length test (SBL = 0.07132081) of 19 East Asian populations, larger than that of 13 prefectures, verified again the greater genetic differences between Jiangsu Han population and various neighbor populations located in East Asia. The phylogeny clearly showed that Jiangsu Han and Dai were from the same node, then clustered with Xuanwei and Beijing Hans, all sharing the same language of Sino-Tibetan. Obviously, the 5 populations (Gunma Japanese, Shizuoka Japanese, South Korean, Tokyo Japanese, and Ibaraki Japanese groups) derived from Altaic language region were close to each other, located in the another flank of N-J tree compared to the bundle containing Jiangsu Han group.

As shown in Fig 6, the MDS plot had a good credibility as initial stress was 0.1067. Jiangsu Han, Beijing Han, Chengdu Han, and Singapore Han populations clustered together in the left lower quadrant. Besides, Jiangsu Han situated far away from Gunma Japanese, Shizuka Japanese and Tokyo Japanese, consistent with the result of N-J tree approximately. The structure reconstruction demonstrated that Jiangsu Han had the closest relationship with Beijing Han (Northern Han). A significant difference existed between the northern and the southern Han of China through archaeological analysis [50] and genetic evidence on Y-SNPs [51], microsatellites [52], and autosomal SNPs [53]. The contribution of Southern or Northern Han to Jiangsu Han population still remained ambiguous, caused by the marginal zones and complex historical population events of Jiangsu province. Previously, Yao et al. [54] have reported that Jiangsu Han was closer to Southern Han (Guangdong Han) than Northern Han (Liaoning Han) based on 15 autosomal STR data. These two opposite opinions provided strong evidence that Jiangsu Han was mixed with Han populations from both side. In population genetics of humans, mtDNA was the most popular molecular [55], unravelling the human origin out of Africa [56–58]. When comparing Y-specific markers and DNA fingerprints from other system, Eric et al. [59] concluded that striking differences showed between the history events and behavior patterns of male and female lineages. Thus, from the accurate perspective, the male portion of Jiangsu Han originated from Northern Han. In future, the population data based on mtDNA and X chromosome of Jiangsu Han would integrate the complex network underlying human dispersal of Jiangsu Han population.

In this research, we enriched the forensic molecular database of Jiangsu Han with the 916 typing files on 23 Y-STRs, which furthered the understanding of population genetics, as well as molecular anthropology. The structure patterns of either inner- or inter-population concerning Jiangsu Han were initially illustrated. This research called for more genotyping profiles of different genetic markers to improve the inference on where and how Jiangsu Han originated and dispersed divergently. The limitation to our research was the disaccord of sample sizes of inner Jiangsu populations, which might have some influences on the structure reconstruction. In next stage, we are dedicated to type more representative samples from Jiangsu Han population to explain the genetic background and population exchange of Jiangsu Han for forensic applications more comprehensively and scientifically.

## Conclusion

As we have noted above, 916 unrelated healthy Han males from Jiangsu province were genotyped at 23 Y-STR loci. 912 different haplotypes, including 908 singletons (99.6%) and 4 duplicates (0.4%), were found in total comprising, revealing that the 23 Y-STRs contained in the PowerPlex^®^ Y23 System were highly polymorphic (HD = 0.9999952) in Jiangsu Han population and were great valuable for forensic application (DC = 0.9956, MP = 0.0011). The highest GD value was 0.9607 at DYS385, and the lowest was 0.3942 at DYS438. Then, LD analysis elaborated the necessity of forming these markers into haplotype. Pairwise R_ST_ genetic distances and relevant significances among inner- and inter-population of Jiangsu Han, combined with N-J trees and MDS plots were conducted to illustrate the genetic background of Jiangsu Han population objectively. For the first time, we depicted the inner-population differentiation and genetic characteristics of Jiangsu Han population. Additionally, in the scale of East Asian, it indicated that the major male component of Han population in Jiangsu came from Northern Chinese Han.

## Supporting information

S1 Table The divisions of haplogroups comprised of 22 Y-STR loci in the Jiangsu Han population (n = 916). The value of "0" indicates the deletion of STR; Microvariants are labeled in bold form.
(DOCX)

S2 Table The distribution of 916 Jiangsu Han samples in prefecture-level geographic location. The value of "0" indicates the deletion of STR; Microvariants are labeled in bold form.
(DOCX)

S3 Table The distribution of allelic frequencies of 21 single-copy Y-STR loci in Jiangsu Han population (n = 916). GD: Gene Diversity.
(DOCX)

S4 Table The distribution of haplogroup frequencies of the multi-copy DYS385 locus in Jiangsu Han population (n = 916). GD: Gene Diversity.
(DOCX)
