# Supplementary material for: Inner and inter population structure construction of Chinese Jiangsu Han population based on Y23 STR system
Source: PLoS One. 2017 Jul 13;12(7):e0180921. doi: 10.1371/journal.pone.0180921 (PMC5509181; doi:10.1371/journal.pone.0180921)
Supplement: S1 Table — The value of "0" indicates the deletion of STR; Microvariants are labeled in bold form. (DOCX) [file pone.0180921.s001.docx]

**S1 Table. The divisions of haplogroups comprised of 22 Y-STR loci in the Jiangsu Han population (n = 916).**

| No. | Count | Haplotype Frequency | Y-STR loci | | | | | | | | | | | | | | | | | | | | | |
| --- | --- | --- | --- | --- | --- | --- | --- | --- | --- | --- | --- | --- | --- | --- | --- | --- | --- | --- | --- | --- | --- | --- | --- | --- |
|  |  |  | DYS576 | DYS389I | DYS448 | DYS389II | DYS19 | DYS391 | DYS481 | DYS549 | DYS533 | DYS438 | DYS437 | DYS570 | DYS635 | DYS390 | DYS439 | DYS392 | DYS643 | DYS393 | DYS458 | DYS385 | DYS456 | YGATAH4 |
| 1 | 2 | 0.0022 | 18 | 12 | 18 | 29 | 15 | 11 | 25 | 12 | 11 | 10 | 14 | 18 | 19 | 23 | 11 | 14 | 11 | 13 | 15 | 13,13 | 17 | 12 |
| 2 | 2 | 0.0022 | 21 | 12 | 20 | 27 | 14 | 10 | 23 | 12 | 12 | 11 | 15 | 20 | 21 | 24 | 12 | 10 | 11 | 12 | 17 | 13,19 | 15 | 11 |
| 3 | 2 | 0.0022 | 16 | 12 | 19 | 29 | 15 | 10 | 22 | 13 | 10 | 10 | 15 | 17 | 19 | 23 | 12 | 12 | 11 | 12 | 17 | 12,17 | 15 | 12 |
| 4 | 2 | 0.0022 | 18 | 14 | 18 | 30 | 16 | 11 | 23 | 12 | 11 | 10 | 14 | 19 | 22 | 25 | 11 | 13 | 10 | 15 | 16 | 12,18 | 14 | 12 |
| 5 | 1 | 0.0011 | 19 | 13 | 21 | 29 | 16 | 10 | 23 | 14 | 12 | 10 | 15 | 16 | 21 | 24 | 12 | 11 | 9 | 15 | 16 | 11,20 | 15 | 11 |
| 6 | 1 | 0.0011 | 18 | 12 | 19 | 27 | 15 | 10 | 23 | 12 | 13 | 10 | 15 | 16 | 21 | 24 | 11 | 13 | 11 | 14 | 17 | 14,18 | 15 | 12 |
| 7 | 1 | 0.0011 | 16 | 13 | 21 | 31 | 15 | 10 | 26 | 12 | 12 | 10 | 14 | 16 | 20 | 23 | 11 | 11 | 8 | 14 | 16 | 11,19 | 15 | 12 |
| 8 | 1 | 0.0011 | 14 | 13 | 19 | 29 | 14 | 10 | 22 | 12 | 12 | 10 | 14 | 17 | 24 | 23 | 11 | 14 | 10 | 13 | 15 | 11,12 | 16 | 12 |
| 9 | 1 | 0.0011 | 18 | 12 | 20 | 28 | 15 | 10 | 23 | 12 | 11 | 10 | 14 | 18 | 21 | 24 | 12 | 13 | 11 | 12 | 16 | 14,20 | 14 | 12 |
| 10 | 1 | 0.0011 | 18 | 14 | 19 | 30 | 14 | 10 | 23 | 12 | 12 | 10 | 14 | 19 | 21 | 22 | 10 | 14 | 10 | 14 | 14 | 12,12 | 15 | 11 |
| 11 | 1 | 0.0011 | 21 | 12 | 19 | 27 | 15 | 10 | 24 | 13 | 11 | 10 | 16 | 19 | 20 | 23 | 13 | 12 | 11 | 13 | 19 | 13,16 | 15 | 10 |
| 12 | 1 | 0.0011 | 19 | 12 | 19 | 27 | 16 | 10 | 22 | 14 | 11 | 11 | 14 | 18 | 20 | 25 | 13 | 14 | 11 | 12 | 18 | 13,17 | 15 | 12 |
| 13 | 1 | 0.0011 | 15 | 12 | 20 | 27 | 17 | 10 | 26 | 13 | 11 | 10 | 14 | 18 | 21 | 24 | 11 | 13 | 9 | 13 | 18 | 13,18 | 14 | 11 |
| 14 | 1 | 0.0011 | 18 | 13 | 20 | 29 | 14 | 11 | 23 | 13 | 11 | 11 | 14 | 19 | 20 | 23 | 12 | 14 | 11 | 12 | 18 | 12,20 | 15 | 12 |
| 15 | 1 | 0.0011 | 20 | 13 | 19 | 31 | 15 | 10 | 22 | 12 | 13 | 10 | 15 | 16 | 19 | 23 | 12 | 12 | 11 | 12 | 19 | 12,12 | 15 | 12 |
| 16 | 1 | 0.0011 | 20 | 14 | 19 | 30 | 13 | 9 | 24 | 11 | 12 | 12 | 14 | 18 | 22 | 24 | 12 | 14 | 10 | 14 | 17 | 15,22 | 15 | 10 |
| 17 | 1 | 0.0011 | 17 | 12 | 20 | 28 | 14 | 10 | 23 | 12 | 10 | 11 | 15 | 18 | 20 | 24 | 12 | 14 | 12 | 11 | 17 | 13,19 | 15 | 12 |
| 18 | 1 | 0.0011 | 17 | 13 | 20 | 29 | 15 | 11 | 23 | 12 | 12 | 10 | 15 | 14 | 21 | 24 | 12 | 13 | 11 | 12 | 15 | 12,17 | 16 | 11 |
| 19 | 1 | 0.0011 | 18 | 14 | 18 | 32 | 15 | 10 | 22 | 12 | 11 | 10 | 14 | 20 | 22 | 24 | 11 | 13 | 11 | 14 | 15 | 12,17 | 13 | 12 |
| 20 | 1 | 0.0011 | 20 | 13 | 21 | 30 | 17 | 10 | 26 | 14 | 13 | 10 | 14 | 18 | 21 | 23 | 12 | 11 | 8 | 15 | 16 | 12,18 | 15 | 11 |
| 21 | 1 | 0.0011 | 19 | 13 | 21 | 29 | 17 | 11 | 24 | 12 | 12 | 10 | 14 | 16 | 22 | 22 | 11 | 11 | 9 | 14 | 15 | 13,17 | 15 | 11 |
| 22 | 1 | 0.0011 | 18 | 14 | 20 | 29 | 14 | 10 | 22 | 12 | 11 | 10 | 14 | 19 | 23 | 23 | 10 | 14 | 10 | 13 | 15 | 11,11 | 17 | 12 |
| 23 | 1 | 0.0011 | 20 | 13 | 20 | 29 | 13 | 9 | 23 | 13 | 12 | 12 | 14 | 20 | 22 | 24 | 10 | 14 | 10 | 13 | 15 | 14,21 | 15 | 10 |
| 24 | 1 | 0.0011 | 17 | 14 | 20 | 31 | 16 | 10 | 23 | 12 | 11 | 10 | 15 | 16 | 21 | 25 | 12 | 13 | 11 | 13 | 18 | 14,20 | 13 | 12 |
| 25 | 1 | 0.0011 | 17 | 12 | 19 | 28 | 16 | 11 | 24 | 12 | 12 | 10 | 14 | 20 | 24 | 25 | 11 | 13 | 10 | 12 | 18 | 11,19 | 14 | 11 |
| 26 | 1 | 0.0011 | 16 | 13 | 18 | 28 | 14 | 10 | 25 | 12 | 12 | 10 | 15 | 19 | 20 | 23 | 13 | 14 | 11 | 13 | 15 | 13,14 | 15 | 12 |
| 27 | 1 | 0.0011 | 19 | 12 | 20 | 28 | 14 | 11 | 23 | 12 | 11 | 11 | 15 | 18 | 20 | 23 | 12 | 14 | 11 | 13 | 21 | 14,18 | 15 | 11 |
| 28 | 1 | 0.0011 | 19 | 13 | 21 | 30 | 17 | 10 | 25 | 12 | 12 | 10 | 14 | 18 | 21 | 23 | 13 | 11 | 8 | 14 | 17 | 11,18 | 15 | 11 |
| 29 | 1 | 0.0011 | 17 | 14 | 20 | 31 | 14 | 10 | 23 | 12 | 11 | 10 | 14 | 18 | 22 | 23 | 12 | 14 | 10 | 13 | 14 | 11,11 | 17 | 12 |
| 30 | 1 | 0.0011 | 17 | 13 | 19 | 26 | 17 | 10 | 24 | 13 | 10 | 10 | 14 | 18 | 25 | 25 | 12 | 13 | 10 | 12 | 18 | 13,19 | 14 | 14 |
| 31 | 1 | 0.0011 | 18 | 12 | 19 | 28 | 13 | 11 | 22 | 12 | 12 | 10 | 15 | 17 | 20 | 23 | 12 | 12 | 9 | 12 | 18 | 12,16 | 15 | 12 |
| 32 | 1 | 0.0011 | 19 | 14 | 19 | 30 | 15 | 10 | 23 | 12 | 11 | 10 | 15 | 16 | 20 | 23 | 12 | 12 | 10 | 12 | 20 | 13,13 | 15 | 13 |
| 33 | 1 | 0.0011 | 20 | 14 | 18 | 30 | 15 | 10 | 23 | 11 | 11 | 10 | 14 | 18 | 20 | 24 | 12 | 13 | 11 | 14 | 15 | 11,18 | 15 | 11 |
| 34 | 1 | 0.0011 | 19 | 12 | 19 | 29 | 16 | 10 | 22 | 12 | 12 | 10 | 15 | 18 | 19 | 23 | 11 | 12 | 11 | 12 | 20 | 12,12 | 15 | 13 |
| 35 | 1 | 0.0011 | 18 | 12 | 19 | 29 | 15 | 10 | 25 | 12 | 11 | 10 | 14 | 19 | 19 | 23 | 11 | 14 | 11 | 13 | 15 | 13,13 | 16 | 12 |
| 36 | 1 | 0.0011 | 19 | 13 | 21 | 29 | 14 | 11 | 23 | 13 | 10 | 11 | 15 | 19 | 21 | 25 | 11 | 13 | 12 | 12 | 17 | 13,16 | 17 | 11 |
| 37 | 1 | 0.0011 | 20 | 12 | 20 | 28 | 14 | 10 | 21 | 11 | 11 | 11 | 15 | 18 | 20 | 25 | 13 | 14 | 12 | 12 | 17 | 13,18 | 15 | 12 |
| 38 | 1 | 0.0011 | 19 | 12 | 20 | 28 | 14 | 10 | 23 | 13 | 11 | 11 | 14 | 19 | 20 | 23 | 12 | 14 | 11 | 12 | 18 | 13,18 | 15 | 12 |
| 39 | 1 | 0.0011 | 18 | 14 | 19 | 29 | 13 | 6 | 23 | 12 | 12 | 13 | 14 | 21 | 22 | 24 | 11 | 14 | 10 | 14 | 16 | 16,19 | 16 | 10 |
| 40 | 1 | 0.0011 | 14 | 14 | 21 | 29 | 16 | 11 | 24 | 13 | 12 | 10 | 14 | 17 | 21 | 23 | 12 | 12 | 9 | 14 | 17 | 10,20 | 15 | 11 |
| 41 | 1 | 0.0011 | 20 | 12 | 19 | 29 | 16 | 11 | 22 | 13 | 11 | 10 | 15 | 17 | 20 | 23 | 12 | 12 | 12 | 14 | 19 | 12,16 | 15 | 11 |
| 42 | 1 | 0.0011 | 17 | 14 | 18 | 31 | 15 | 10 | 23 | 12 | 11 | 10 | 14 | 18 | 22 | 24 | 11 | 13 | 11 | 14 | 14 | 12,16 | 15 | 12 |
| 43 | 1 | 0.0011 | 17 | 14 | 21 | 30 | 15 | 10 | 26 | 12 | 12 | 10 | 14 | 16 | 21 | 22 | 12 | 11 | 8 | 14 | 16 | 11,20 | 15 | 11 |
| 44 | 1 | 0.0011 | 19 | 12 | 19 | 28 | 14 | 10 | 22 | 13 | 11 | 10 | 15 | 19 | 19 | 23 | 12 | 12 | 11 | 12 | 18 | 12,17 | 15 | 12 |
| 45 | 1 | 0.0011 | 20 | 14 | 19 | 30 | 13 | 10 | 24 | 12 | 12 | 12 | 14 | 20 | 22 | 24 | 11 | 15 | 11 | 14 | 16 | 15,22 | 16 | 10 |
| 46 | 1 | 0.0011 | 18 | 12 | 19 | 28 | 16 | 10 | 22 | 13 | 11 | 10 | 15 | 17 | 20 | 23 | 13 | 12 | 10 | 12 | 17 | 12,16 | 15 | 11 |
| 47 | 1 | 0.0011 | 20 | 12 | 20 | 28 | 17 | 10 | 23 | 13 | 11 | 10 | 14 | 19 | 22 | 25 | 11 | 13 | 10 | 12 | 20 | 14,19 | 14 | 12 |
| 48 | 1 | 0.0011 | 18 | 12 | 21 | 29 | 16 | 10 | 23 | 13 | 11 | 10 | 15 | 19 | 22 | 23 | 12 | 13 | 13 | 12 | 16 | 12,21 | 15 | 12 |
| 49 | 1 | 0.0011 | 17 | 13 | 19 | 30 | 15 | 11 | 22 | 14 | 11 | 10 | 14 | 17 | 21 | 23 | 11 | 12 | 12 | 13 | 17 | 15,19 | 15 | 12 |
| 50 | 1 | 0.0011 | 18 | 12 | 20 | 28 | 15 | 10 | 23 | 12 | 11 | 10 | 15 | 19 | 21 | 24 | 10 | 13 | 11 | 12 | 18 | 13,20 | 15 | 11 |
| 51 | 1 | 0.0011 | 18 | 12 | 20 | 27 | 16 | 10 | 23 | 13 | 12 | 11 | 15 | 19 | 20 | 24 | 12 | 14 | 11 | 12 | 18 | 14,18 | 15 | 12 |
| 52 | 1 | 0.0011 | 20 | 12 | 19 | 30 | 15 | 10 | 22 | 12 | 11 | 10 | 15 | 16 | 20 | 23 | 13 | 12 | 12 | 12 | 20 | 12,17 | 15 | 13 |
| 53 | 1 | 0.0011 | 18 | 12 | 20 | 28 | 14 | 10 | 23 | 13 | 11 | 11 | 15 | 17 | 20 | 23 | 12 | 14 | 11 | 12 | 17 | 15,19 | 15 | 12 |
| 54 | 1 | 0.0011 | 17 | 14 | 18 | 30 | 15 | 10 | 21 | 12 | 13 | 10 | 14 | 20 | 20 | 23 | 10 | 14 | 11 | 13 | 16 | 10,12 | 14 | 11 |
| 55 | 1 | 0.0011 | 18 | 13 | 19 | 30 | 15 | 10 | 27 | 11 | 12 | 10 | 14 | 21 | 21 | 25 | 11 | 10 | 9 | 12 | 23 | 16,16 | 15 | 11 |
| 56 | 1 | 0.0011 | 18 | 14 | 19 | 31 | 13 | 9 | 24 | 13 | 12 | 12 | 14 | 19 | 22 | 24 | 11 | 14 | 10 | 13 | 16 | 16,21 | 16 | 10 |
| 57 | 1 | 0.0011 | 18 | 12 | 19 | 28 | 15 | 10 | 23 | 12 | 12 | 10 | 14 | 18 | 23 | 25 | 12 | 13 | 10 | 12 | 18 | 12,18 | 14 | 11 |
| 58 | 1 | 0.0011 | 19 | 14 | 18 | 31 | 15 | 10 | 24 | 12 | 11 | 10 | 14 | 19 | 21 | 25 | 11 | 13 | 11 | 14 | 15 | 12,17 | 15 | 12 |
| 59 | 1 | 0.0011 | 18 | 12 | 18 | 28 | 15 | 11 | 25 | 12 | 11 | 10 | 14 | 18 | 19 | 23 | 11 | 14 | 11 | 13 | 15 | 13,13 | 16 | 12 |
| 60 | 1 | 0.0011 | 17 | 12 | 20 | 29 | 16 | 10 | 24 | 12 | 12 | 10 | 15 | 16 | 22 | 23 | 11 | 13 | 12 | 13 | 18 | 13,18 | 14 | 11 |
| 61 | 1 | 0.0011 | 18 | 13 | 19 | 29 | 16 | 10 | 24 | 12 | 11 | 10 | 13 | 21 | 20 | 25 | 11 | 13 | 11 | 12 | 20 | 12,19 | 14 | 12 |
| 62 | 1 | 0.0011 | 19 | 12 | 0 | 29 | 15 | 10 | 26 | 15 | 12 | 9 | 15 | 14 | 21 | 24 | 11 | 13 | 12 | 12 | 15 | 12,18 | 15 | 12 |
| 63 | 1 | 0.0011 | 20 | 12 | 20 | 29 | 15 | 11 | 21 | 13 | 11 | 11 | 15 | 19 | 22 | 23 | 13 | 12 | 11 | 12 | 21 | 12,12 | 15 | 12 |
| 64 | 1 | 0.0011 | 14 | 13 | 19 | 29 | 14 | 10 | 22 | 11 | 12 | 10 | 14 | 18 | 22 | 23 | 10 | 14 | 10 | 13 | 15 | 11,12 | 17 | 12 |
| 65 | 1 | 0.0011 | 19 | 12 | 18 | 28 | 16 | 10 | 22 | 11 | 11 | 10 | 16 | 17 | 23 | 23 | 11 | 12 | 12 | 13 | 19 | 11,18 | 16 | 11 |
| 66 | 1 | 0.0011 | 18 | 11 | 20 | 27 | 15 | 10 | 24 | 13 | 11 | 10 | 15 | 21 | 20 | 24 | 12 | 13 | 12 | 13 | 16 | 13,26 | 14 | 11 |
| 67 | 1 | 0.0011 | 19 | 14 | 19 | 30 | 16 | 10 | 24 | 12 | 12 | 10 | 14 | 18 | 22 | 24 | 12 | 13 | 10 | 12 | 17 | 13,18 | 15 | 12 |
| 68 | 1 | 0.0011 | 18 | 12 | 19 | 27 | 15 | 10 | 23 | 12 | 11 | 10 | 14 | 17 | 23 | 25 | 11 | 13 | 10 | 12 | 18 | 12,19 | 15 | 11 |
| 69 | 1 | 0.0011 | 20 | 13 | 19 | 28 | 17 | 10 | 23 | 14 | 11 | 10 | 15 | 18 | 20 | 22 | 12 | 14 | 10 | 12 | 17 | 11,12 | 15 | 11 |
| 70 | 1 | 0.0011 | 17 | 13 | 18 | 30 | 15 | 11 | 25 | 13 | 11 | 10 | 14 | 18 | 19 | 23 | 11 | 14 | 11 | 13 | 15 | 13,13 | 17 | 12 |
| 71 | 1 | 0.0011 | 17 | 12 | 18 | 29 | 15 | 11 | 24 | 12 | 11 | 10 | 14 | 19 | 19 | 23 | 11 | 14 | 9 | 13 | 15 | 13,13 | 17 | 11 |
| 72 | 1 | 0.0011 | 17 | 12 | 20 | 30 | 15 | 10 | 24 | 13 | 11 | 10 | 16 | 19 | 21 | 25 | 11 | 13 | 11 | 12 | 16 | 13,20 | 14 | 13 |
| 73 | 1 | 0.0011 | 20 | 12 | 19 | 27 | 16 | 10 | 24 | 12 | 11 | 10 | 14 | 17 | 21 | 23 | 10 | 14 | 13 | 12 | 16 | 13,14 | 16 | 13 |
| 74 | 1 | 0.0011 | 19 | 13 | 19 | 30 | 17 | 10 | 25 | 12 | 11 | 10 | 14 | 19 | 22 | 24 | 12 | 13 | 10 | 12 | 20 | 12,19 | 14 | 11 |
| 75 | 1 | 0.0011 | 18 | 12 | 19 | 28 | 15 | 10 | 25 | 12 | 11 | 10 | 14 | 18 | 19 | 23 | 12 | 14 | 11 | 13 | 15 | 13,13 | 17 | 12 |
| 76 | 1 | 0.0011 | 17 | 12 | 18 | 28 | 15 | 11 | 25 | 12 | 11 | 10 | 14 | 18 | 19 | 23 | 11 | 14 | 11 | 13 | 17 | 13,13 | 17 | 12 |
| 77 | 1 | 0.0011 | 17 | 13 | 19 | 30 | 16 | 10 | 22 | 12 | 11 | 10 | 15 | 16 | 20 | 24 | 11 | 12 | 11 | 12 | 17 | 11,16 | 15 | 12 |
| 78 | 1 | 0.0011 | 19 | 12 | 19 | 28 | 17 | 10 | 24 | 13 | 11 | 10 | 14 | 17 | 22 | 25 | 12 | 13 | 10 | 12 | 16 | 12,22 | 14 | 11 |
| 79 | 1 | 0.0011 | 20 | 14 | 19 | 30 | 15 | 10 | 23 | 13 | 11 | 10 | 15 | 16 | 20 | 24 | 14 | 12 | 12 | 12 | 15 | 12,17 | 15 | 11 |
| 80 | 1 | 0.0011 | 18 | 13 | 18 | 29 | 15 | 10 | 23 | 12 | 11 | 11 | 14 | 19 | 22 | 25 | 12 | 13 | 12 | 13 | 16 | 13,19 | 15 | 11 |
| 81 | 1 | 0.0011 | 18 | 12 | 20 | 28 | 15 | 11 | 24 | 13 | 11 | 11 | 15 | 18 | 20 | 24 | 13 | 14 | 11 | 12 | 18 | 13,19 | 15 | 13 |
| 82 | 1 | 0.0011 | 19 | 12 | 18 | 27 | 15 | 11 | 25 | 13 | 11 | 10 | 14 | 20 | 19 | 23 | 12 | 14 | 11 | 13 | 15 | 13,13 | 17 | 12 |
| 83 | 1 | 0.0011 | 18 | 14 | 20 | 31 | 15 | 10 | 23 | 12 | 11 | 10 | 15 | 17 | 22 | 24 | 13 | 13 | 11 | 12 | 18 | 14,21 | 13 | 12 |
| 84 | 1 | 0.0011 | 20 | 13 | 19 | 28 | 17 | 10 | 24 | 12 | 11 | 10 | 14 | 18 | 23 | 25 | 12 | 13 | 10 | 12 | 17 | 13,19 | 14 | 12 |
| 85 | 1 | 0.0011 | 18 | 14 | 18 | 30 | 14 | 11 | 21 | 12 | 12 | 10 | 15 | 18 | 21 | 23 | 10 | 14 | 11 | 13 | 17 | 11,12 | 14 | 11 |
| 86 | 1 | 0.0011 | 17 | 12 | 20 | 28 | 15 | 10 | 24 | 13 | 12 | 11 | 15 | 17 | 20 | 24 | 12 | 13 | 11 | 12 | 17 | 13,20 | 15 | 12 |
| 87 | 1 | 0.0011 | 18 | 12 | 20 | 28 | 14 | 10 | 25 | 12 | 11 | 10 | 15 | 19 | 21 | 25 | 11 | 13 | 11 | 12 | 19 | 13,18 | 15 | 11 |
| 88 | 1 | 0.0011 | 18 | 12 | 20 | 27 | 16 | 10 | 24 | 14 | 11 | 9 | 14 | 18 | 22 | 25 | 12 | 13 | 10 | 12 | 18 | 13,19 | 14 | 12 |
| 89 | 1 | 0.0011 | 19 | 14 | 20 | 30 | 14 | 10 | 24 | 12 | 10 | 10 | 15 | 20 | 21 | 24 | 11 | 13 | 12 | 12 | 16 | 13,18 | 16 | 11 |
| 90 | 1 | 0.0011 | 18 | 13 | 20 | 29 | 16 | 10 | 25 | 13 | 11 | 10 | 11 | 17 | 23 | 24 | 13 | 13 | 10 | 12 | 17 | 12,18 | 15 | 11 |
| 91 | 1 | 0.0011 | 19 | 12 | 18 | 28 | 16 | 11 | 25 | 12 | 12 | 10 | 14 | 19 | 19 | 24 | 11 | 14 | 11 | 13 | 15 | 13,14 | 17 | 12 |
| 92 | 1 | 0.0011 | 18 | 12 | 20 | 28 | 15 | 10 | 25 | 12 | 12 | 11 | 15 | 18 | 19 | 24 | 11 | 14 | 11 | 12 | 19 | 13,18 | 15 | 12 |
| 93 | 1 | 0.0011 | 18 | 12 | 20 | 28 | 14 | 10 | 24 | 12 | 11 | 11 | 15 | 18 | 20 | 24 | 11 | 14 | 13 | 12 | 17 | 13,17 | 15 | 12 |
| 94 | 1 | 0.0011 | 20 | 12 | 19 | 29 | 15 | 11 | 21 | 12 | 11 | 10 | 14 | 16 | 20 | 23 | 12 | 12 | 11 | 12 | 19 | 12,17 | 16 | 12 |
| 95 | 1 | 0.0011 | 18 | 12 | 19 | 27 | 15 | 10 | 23 | 13 | 11 | 10 | 15 | 19 | 19 | 23 | 11 | 12 | 11 | 13 | 19 | 11,17 | 15 | 12 |
| 96 | 1 | 0.0011 | 17 | 14 | 20 | 32 | 15 | 10 | 23 | 12 | 11 | 10 | 15 | 17 | 21 | 25 | 12 | 13 | 11 | 12 | 17 | 15,21 | 14 | 12 |
| 97 | 1 | 0.0011 | 18 | 12 | 20 | 28 | 14 | 11 | 25 | 12 | 11 | 11 | 15 | 19 | 21 | 24 | 11 | 15 | 11 | 12 | 17 | 13,21 | 16 | 11 |
| 98 | 1 | 0.0011 | 19 | 12 | 18 | 28 | 17 | 11 | 23 | 12 | 11 | 10 | 14 | 18 | 21 | 25 | 11 | 13 | 10 | 12 | 18 | 12,19 | 14 | 12 |
| 99 | 1 | 0.0011 | 17 | 14 | 19 | 30 | 15 | 10 | 23 | 11 | 10 | 10 | 14 | 19 | 20 | 22 | 11 | 15 | 10 | 13 | 18 | 11,14 | 15 | 12 |
| 100 | 1 | 0.0011 | 19 | 12 | 18 | 27 | 16 | 11 | 21 | 13 | 11 | 10 | 14 | 18 | 20 | 23 | 12 | 14 | 11 | 13 | 15 | 12,13 | 17 | 12 |
| 101 | 1 | 0.0011 | 18 | 13 | 21 | 29 | 15 | 10 | 27 | 12 | 12 | 10 | 14 | 17 | 21 | 24 | 11 | 11 | 8 | 15 | 15 | 11,19 | 15 | 11 |
| 102 | 1 | 0.0011 | 16 | 12 | 18 | 28 | 15 | 10 | 24 | 14 | 11 | 10 | 14 | 20 | 21 | 23 | 11 | 14 | 11 | 13 | 15 | 12,13 | 15 | 12 |
| 103 | 1 | 0.0011 | 17 | 13 | 20 | 32 | 15 | 10 | 26 | 13 | 11 | 11 | 15 | 20 | 20 | 23 | 14 | 13 | 12 | 13 | 16 | 13,23 | 15 | 10 |
| 104 | 1 | 0.0011 | 21 | 13 | 19 | 30 | 15 | 10 | 23 | 12 | 11 | 10 | 14 | 16 | 21 | 23 | 11 | 13 | 11 | 12 | 17 | 19,19 | 14 | 11 |
| 105 | 1 | 0.0011 | 19 | 14 | 19 | 31 | 15 | 10 | 23 | 11 | 12 | 10 | 14 | 19 | 21 | 22 | 12 | 14 | 10 | 13 | 18 | 11,15 | 15 | 11 |
| 106 | 1 | 0.0011 | 18 | 12 | 18 | 27 | 16 | 10 | 24 | 12 | 11 | 10 | 14 | 18 | 24 | 25 | 11 | 13 | 10 | 12 | 19 | 14,18 | 14 | 12 |
| 107 | 1 | 0.0011 | 19 | 11 | 18 | 27 | 17 | 10 | 24 | 12 | 11 | 10 | 14 | 18 | 22 | 25 | 12 | 13 | 10 | 12 | 18 | 12,20 | 14 | 12 |
| 108 | 1 | 0.0011 | 18 | 11 | 21 | 27 | 16 | 10 | 25 | 12 | 13 | 10 | 14 | 16 | 21 | 23 | 11 | 11 | 9 | 14 | 16 | 11,17 | 15 | 11 |
| 109 | 1 | 0.0011 | 19 | 14 | 19 | 30 | 15 | 10 | 29 | 12 | 11 | 10 | 15 | 17 | 21 | 24 | 13 | 13 | 11 | 12 | 18 | 13,19 | 15 | 11 |
| 110 | 1 | 0.0011 | 20 | 12 | 19 | 28 | 14 | 10 | 22 | 13 | 11 | 11 | 15 | 18 | 19 | 23 | 12 | 12 | 10 | 12 | 18 | 12,18 | 15 | 12 |
| 111 | 1 | 0.0011 | 18 | 12 | 18 | 30 | 15 | 11 | 24 | 12 | 12 | 10 | 14 | 18 | 20 | 23 | 11 | 14 | 11 | 13 | 14 | 13,14 | 17 | 12 |
| 112 | 1 | 0.0011 | 18 | 12 | 19 | 28 | 14 | 11 | 22 | 12 | 11 | 10 | 15 | 18 | 19 | 23 | 11 | 12 | 11 | 12 | 18 | 12,17 | 15 | 12 |
| 113 | 1 | 0.0011 | 18 | 14 | 22 | 29 | 16 | 9 | 26 | 11 | 11 | 10 | 15 | 19 | 20 | 25 | 14 | 13 | 12 | 14 | 20 | 12,17 | 15 | 10 |
| 114 | 1 | 0.0011 | 18 | 13 | 20 | 29 | 13 | 6 | 24 | 12 | 12 | 12 | 14 | 20 | 22 | 24 | 11 | 14 | 10 | 14 | 14 | 16,20 | 17 | 10 |
| 115 | 1 | 0.0011 | 18 | 12 | 20 | 27 | 14 | 10 | 23 | 13 | 11 | 11 | 16 | 17 | 21 | 24 | 12 | 14 | 11 | 12 | 17 | 15,19 | 15 | 12 |
| 116 | 1 | 0.0011 | 16 | 13 | 19 | 28 | 16 | 10 | 23 | 13 | 11 | 10 | 15 | 17 | 20 | 21 | 12 | 14 | 10 | 12 | 18 | 11,11 | 15 | 13 |
| 117 | 1 | 0.0011 | 20 | 12 | 20 | 27 | 13 | 10 | 22 | 12 | 11 | 11 | 15 | 16 | 19 | 23 | 12 | 12 | 11 | 12 | 17 | 13,16 | 15 | 12 |
| 118 | 1 | 0.0011 | 18 | 12 | 20 | 29 | 15 | 10 | 24 | 13 | 11 | 10 | 15 | 19 | 21 | 25 | 12 | 13 | 11 | 12 | 16 | 13,20 | 14 | 13 |
| 119 | 1 | 0.0011 | 17 | 12 | 19 | 28 | 16 | 10 | 22 | 14 | 11 | 10 | 15 | 16 | 20 | 23 | 11 | 12 | 11 | 12 | 19 | 13,17 | 16 | 12 |
| 120 | 1 | 0.0011 | 15 | 13 | 21 | 29 | 16 | 10 | 23 | 13 | 12 | 9 | 15 | 18 | 21 | 23 | 12 | 11 | 10 | 13 | 15 | 13,16 | 15 | 12 |
| 121 | 1 | 0.0011 | 16 | 12 | 20 | 28 | 14 | 10 | 24 | 12 | 10 | 11 | 15 | 20 | 20 | 24 | 12 | 13 | 12 | 12 | 18 | 14,18 | 15 | 12 |
| 122 | 1 | 0.0011 | 16 | 13 | 22 | 30 | 15 | 10 | 25 | 12 | 12 | 10 | 14 | 17 | 21 | 23 | 13 | 11 | 9 | 15 | 16 | 11,17 | 15 | 12 |
| 123 | 1 | 0.0011 | 18 | 13 | 19 | 29 | 15 | 10 | 23 | 13 | 12 | 10 | 14 | 22 | 22 | 22 | 11 | 15 | 10 | 13 | 16 | 11,14 | 16 | 12 |
| 124 | 1 | 0.0011 | 22 | 12 | 20 | 28 | 13 | 11 | 24 | 11 | 13 | 11 | 14 | 21 | 20 | 24 | 12 | 14 | 10 | 12 | 18 | 13,14 | 15 | 13 |
| 125 | 1 | 0.0011 | 19 | 12 | 20 | 28 | 15 | 10 | 23 | 12 | 11 | 11 | 14 | 18 | 20 | 24 | 12 | 14 | 11 | 13 | 17 | 14,19 | 15 | 13 |
| 126 | 1 | 0.0011 | 17 | 12 | 19 | 28 | 15 | 10 | 23 | 12 | 11 | 10 | 14 | 16 | 19 | 23 | 13 | 12 | 14 | 12 | 16 | 11,16 | 16 | 12 |
| 127 | 1 | 0.0011 | 18 | 12 | 18 | 29 | 16 | 10 | 25 | 12 | 11 | 10 | 14 | 17 | 22 | 25 | 11 | 13 | 10 | 12 | 19 | 12,18 | 14 | 11 |
| 128 | 1 | 0.0011 | 18 | 12 | 21 | 28 | 16 | 10 | 23 | 13 | 11 | 10 | 15 | 20 | 22 | 23 | 13 | 14 | 13 | 12 | 18 | 12,20 | 15 | 12 |
| 129 | 1 | 0.0011 | 19 | 12 | 19 | 27 | 15 | 10 | 22 | 13 | 11 | 10 | 15 | 18 | 20 | 23 | 11 | 12 | 10 | 12 | 19 | 12,17 | 16 | 12 |
| 130 | 1 | 0.0011 | 19 | 12 | 20 | 29 | 14 | 11 | 23 | 11 | 11 | 11 | 15 | 19 | 21 | 23 | 12 | 14 | 11 | 12 | 18 | 13,18 | 15 | 11 |
| 131 | 1 | 0.0011 | 18 | 12 | 18 | 29 | 16 | 11 | 25 | 11 | 11 | 10 | 14 | 18 | 19 | 23 | 11 | 14 | 11 | 13 | 15 | 13,14 | 17 | 13 |
| 132 | 1 | 0.0011 | 17 | 14 | 19 | 31 | 15 | 10 | 28 | 11 | 12 | 10 | 14 | 19 | 21 | 25 | 11 | 10 | 9 | 12 | 22 | 16,16 | 15 | 11 |
| 133 | 1 | 0.0011 | 17 | 12 | 18 | 28 | 15 | 10 | 22 | 13 | 11 | 10 | 15 | 16 | 20 | 24 | 11 | 12 | 11 | 12 | 19 | 12,15 | 15 | 11 |
| 134 | 1 | 0.0011 | 17 | 12 | 18 | 28 | 15 | 11 | 23 | 13 | 11 | 10 | 14 | 19 | 21 | 23 | 12 | 14 | 11 | 13 | 14 | 13,14 | 16 | 12 |
| 135 | 1 | 0.0011 | 19 | 12 | 19 | 27 | 16 | 10 | 24 | 14 | 11 | 10 | 13 | 19 | 22 | 25 | 13 | 15 | 10 | 12 | 17 | 13,19 | 16 | 12 |
| 136 | 1 | 0.0011 | 16 | 12 | 20 | 31 | 16 | 10 | 24 | 13 | 11 | 10 | 14 | 16 | 22 | 24 | 11 | 13 | 10 | 12 | 19 | 14,17 | 13 | 12 |
| 137 | 1 | 0.0011 | 19 | 12 | 19 | 29 | 15 | 10 | 21 | 12 | 11 | 10 | 14 | 17 | 19 | 23 | 12 | 12 | 11 | 12 | 18 | 12,17 | 16 | 12 |
| 138 | 1 | 0.0011 | 18 | 12 | 19 | 28 | 16 | 10 | 23 | 14 | 12 | 10 | 14 | 18 | 21 | 25 | 12 | 13 | 10 | 12 | 18 | 14,18 | 13 | 11 |
| 139 | 1 | 0.0011 | 17 | 13 | 19 | 28 | 14 | 11 | 23 | 12 | 12 | 11 | 14 | 20 | 22 | 24 | 11 | 16 | 11 | 13 | 16 | 11,12 | 16 | 12 |
| 140 | 1 | 0.0011 | 18 | 14 | 19 | 29 | 15 | 10 | 23 | 13 | 11 | 10 | 15 | 19 | 22 | 21 | 11 | 14 | 11 | 12 | 18 | 11,11 | 14 | 11 |
| 141 | 1 | 0.0011 | 18 | 12 | 19 | 28 | 15 | 10 | 23 | 13 | 11 | 10 | 14 | 17 | 19 | 23 | 13 | 12 | 12 | 12 | 17 | 12,16 | 16 | 13 |
| 142 | 1 | 0.0011 | 19 | 12 | 18 | 27 | 15 | 10 | 21 | 12 | 11 | 10 | 14 | 18 | 20 | 23 | 12 | 14 | 11 | 13 | 15 | 12,13 | 18 | 13 |
| 143 | 1 | 0.0011 | 18 | 12 | 20 | 28 | 14 | 10 | 23 | 13 | 11 | 11 | 15 | 20 | 20 | 25 | 11 | 14 | 11 | 12 | 17 | 14,19 | 15 | 13 |
| 144 | 1 | 0.0011 | 18 | 14 | 21 | 30 | 15 | 10 | 26 | 13 | 13 | 10 | 14 | 17 | 21 | 23 | 12 | 11 | 8 | 14 | 15 | 11,16 | 15 | 11 |
| 145 | 1 | 0.0011 | 16 | 12 | 20 | 28 | 14 | 10 | 25 | 12 | 11 | 11 | 14 | 18 | 20 | 24 | 13 | 14 | 10 | 12 | 17 | 13,19 | 15 | 12 |
| 146 | 1 | 0.0011 | 18 | 12 | 20 | 28 | 15 | 10 | 21 | 13 | 11 | 11 | 15 | 17 | 24 | 23 | 12 | 12 | 12 | 12 | 18 | 11,12 | 15 | 12 |
| 147 | 1 | 0.0011 | 16 | 12 | 20 | 28 | 14 | 10 | 23 | 12 | 13 | 11 | 15 | 17 | 20 | 23 | 12 | 14 | 11 | 12 | 17 | 15,19 | 15 | 12 |
| 148 | 1 | 0.0011 | 19 | 12 | 18 | 30 | 16 | 10 | 26 | 12 | 12 | 10 | 14 | 15 | 20 | 23 | 11 | 13 | 11 | 13 | 17 | 12,17 | 15 | 12 |
| 149 | 1 | 0.0011 | 18 | 12 | 19 | 28 | 15 | 10 | 22 | 12 | 11 | 10 | 14 | 16 | 19 | 23 | 13 | 12 | 11 | 12 | 17 | 12,19 | 15 | 12 |
| 150 | 1 | 0.0011 | 20 | 12 | 19 | 27 | 16 | 10 | 24 | 13 | 11 | 10 | 13 | 18 | 22 | 25 | 13 | 15 | 10 | 12 | 19 | 12,19 | 15 | 12 |
| 151 | 1 | 0.0011 | 17 | 12 | 20 | 28 | 14 | 10 | 24 | 13 | 11 | 11 | 15 | 19 | 20 | 24 | 12 | 14 | 11 | 12 | 18 | 13,18 | 15 | 13 |
| 152 | 1 | 0.0011 | 17 | 14 | 19 | 30 | 15 | 11 | 22 | 12 | 12 | 11 | 14 | 21 | 22 | 24 | 12 | 15 | 10 | 13 | 16 | 11,12 | 16 | 12 |
| 153 | 1 | 0.0011 | 18 | 12 | 19 | 29 | 15 | 10 | 23 | 12 | 12 | 10 | 15 | 18 | 20 | 24 | 11 | 13 | 12 | 13 | 18 | 12,19 | 14 | 11 |
| 154 | 1 | 0.0011 | 18 | 12 | 20 | 27 | 15 | 10 | 22 | 15 | 11 | 11 | 15 | 18 | 20 | 25 | 12 | 14 | 11 | 12 | 18 | 13,17 | 16 | 12 |
| 155 | 1 | 0.0011 | 18 | 12 | 18 | 30 | 15 | 10 | 26 | 12 | 11 | 10 | 14 | 17 | 19 | 23 | 11 | 14 | 11 | 13 | 15 | 13,13 | 16 | 12 |
| 156 | 1 | 0.0011 | 16 | 12 | 20 | 29 | 15 | 10 | 24 | 13 | 12 | 10 | 15 | 14 | 21 | 24 | 12 | 13 | 11 | 12 | 17 | 12,20 | 15 | 11 |
| 157 | 1 | 0.0011 | 16 | 13 | 20 | 28 | 15 | 10 | 24 | 12 | 12 | 10 | 14 | 22 | 21 | 24 | 11 | 15 | 10 | 13 | 16 | 11,12 | 16 | 13 |
| 158 | 1 | 0.0011 | 18 | 12 | 19 | 28 | 15 | 10 | 21 | 12 | 11 | 10 | 14 | 16 | 20 | 23 | 12 | 12 | 11 | 12 | 17 | 12,17 | 16 | 13 |
| 159 | 1 | 0.0011 | 17 | 12 | 19 | 28 | 17 | 10 | 25 | 13 | 11 | 10 | 14 | 18 | 21 | 24 | 12 | 13 | 11 | 12 | 18 | 12,19 | 14 | 12 |
| 160 | 1 | 0.0011 | 18 | 14 | 18 | 29 | 15 | 11 | 23 | 11 | 13 | 10 | 14 | 18 | 21 | 23 | 10 | 14 | 11 | 14 | 17 | 12,12 | 14 | 11 |
| 161 | 1 | 0.0011 | 16 | 12 | 20 | 27 | 16 | 10 | 24 | 13 | 11 | 10 | 14 | 20 | 22 | 25 | 12 | 13 | 10 | 13 | 18 | 14,19 | 14 | 12 |
| 162 | 1 | 0.0011 | 18 | 12 | 20 | 28 | 14 | 10 | 23 | 12 | 11 | 11 | 15 | 19 | 20 | 25 | 13 | 14 | 12 | 12 | 17 | 14,19 | 15 | 12 |
| 163 | 1 | 0.0011 | 17 | 12 | 18 | 29 | 15 | 11 | 26 | 12 | 11 | 10 | 14 | 18 | 19 | 23 | 12 | 14 | 11 | 13 | 17 | 13,13 | 17 | 13 |
| 164 | 1 | 0.0011 | 18 | 14 | 18 | 30 | 15 | 10 | 23 | 12 | 11 | 10 | 15 | 20 | 21 | 24 | 13 | 13 | 11 | 15 | 15 | 12,17 | 15 | 12 |
| 165 | 1 | 0.0011 | 20 | 13 | 19 | 29 | 17 | 10 | 23 | 13 | 11 | 10 | 15 | 17 | 22 | 22 | 12 | 13 | 10 | 12 | 18 | 11,11 | 15 | 11 |
| 166 | 1 | 0.0011 | 16 | 13 | 17 | 30 | 14 | 10 | 28 | 14 | 11 | 10 | 14 | 19 | 21 | 24 | 10 | 11 | 10 | 13 | 17 | 14,19 | 15 | 11 |
| 167 | 1 | 0.0011 | 18 | 12 | 19 | 30 | 15 | 11 | 24 | 12 | 11 | 10 | 14 | 17 | 20 | 23 | 11 | 13 | 11 | 13 | 16 | 13,13 | 15 | 12 |
| 168 | 1 | 0.0011 | 20 | 13 | 20 | 29 | 14 | 10 | 23 | 13 | 11 | 11 | 15 | 20 | 22 | 25 | 11 | 13 | 13 | 13 | 17 | 14,19 | 16 | 11 |
| 169 | 1 | 0.0011 | 19 | 13 | 20 | 30 | 17 | 10 | 25 | 13 | 11 | 10 | 14 | 19 | 23 | 25 | 11 | 13 | 10 | 12 | 20 | 14,17 | 15 | 12 |
| 170 | 1 | 0.0011 | 18 | 12 | 20 | 29 | 14 | 10 | 23 | 12 | 11 | 11 | 15 | 18 | 20 | 24 | 12 | 14 | 11 | 12 | 17 | 13,21 | 15 | 12 |
| 171 | 1 | 0.0011 | 19 | 12 | 20 | 28 | 14 | 10 | 22 | 12 | 11 | 11 | 14 | 18 | 20 | 24 | 13 | 14 | 11 | 12 | 17 | 13,19 | 15 | 12 |
| 172 | 1 | 0.0011 | 18 | 12 | 20 | 29 | 14 | 10 | 23 | 12 | 11 | 11 | 15 | 18 | 20 | 23 | 13 | 14 | 11 | 12 | 18 | 13,19 | 16 | 13 |
| 173 | 1 | 0.0011 | 19 | 12 | 19 | 28 | 15 | 10 | 23 | 12 | 11 | 10 | 15 | 17 | 19 | 23 | 12 | 12 | 11 | 12 | 18 | 12,16 | 17 | 12 |
| 174 | 1 | 0.0011 | 21 | 12 | 21 | 28 | 14 | 10 | 23 | 13 | 11 | 11 | 15 | 18 | 20 | 24 | 12 | 14 | 11 | 12 | 18 | 13,19 | 14 | 12 |
| 175 | 1 | 0.0011 | 17 | 13 | 19 | 27 | 15 | 10 | 27 | 11 | 12 | 10 | 14 | 21 | 20 | 23 | 11 | 10 | 9 | 12 | 21 | 15,16 | 15 | 11 |
| 176 | 1 | 0.0011 | 20 | 12 | 21 | 28 | 14 | 10 | 23 | 12 | 10 | 11 | 15 | 18 | 18 | 24 | 13 | 14 | 11 | 12 | 18 | 14,19 | 15 | 12 |
| 177 | 1 | 0.0011 | 17 | 13 | 21 | 29 | 16 | 10 | 23 | 13 | 14 | 10 | 14 | 16 | 21 | 23 | 12 | 11 | 9 | 15 | 16 | **11,16.2** | 15 | 11 |
| 178 | 1 | 0.0011 | 18 | 12 | 19 | 28 | 15 | 10 | 25 | 13 | 11 | 10 | 14 | 18 | 20 | 23 | 11 | 14 | 11 | 13 | 15 | 13,14 | 16 | 13 |
| 179 | 1 | 0.0011 | 16 | 13 | 20 | 30 | 15 | 10 | 26 | 12 | 12 | 11 | 14 | 16 | 21 | 23 | 11 | 11 | 9 | 14 | 16 | 11,18 | 15 | 12 |
| 180 | 1 | 0.0011 | 19 | 14 | 19 | 30 | 14 | 10 | 24 | 11 | 12 | 10 | 14 | 19 | 21 | 22 | 12 | 14 | 10 | 13 | 17 | 11,14 | 15 | 12 |
| 181 | 1 | 0.0011 | 18 | 14 | 18 | 30 | 14 | 11 | 21 | 12 | 13 | 10 | 14 | 19 | 21 | 23 | 10 | 14 | 11 | 12 | 17 | 11,12 | 14 | 11 |
| 182 | 1 | 0.0011 | 18 | 13 | 18 | 29 | 14 | 9 | 24 | 12 | 10 | 10 | 14 | 19 | 21 | 23 | 11 | 15 | 10 | 14 | 16 | 12,18 | 15 | 12 |
| 183 | 1 | 0.0011 | 18 | 13 | 20 | 29 | 14 | 10 | 23 | 12 | 11 | 11 | 14 | 17 | 20 | 25 | 12 | 15 | 11 | 12 | 21 | 13,20 | 16 | 11 |
| 184 | 1 | 0.0011 | 18 | 12 | 19 | 27 | 16 | 10 | 24 | 15 | 11 | 10 | 13 | 19 | 21 | 25 | 12 | 15 | 11 | 13 | 19 | 12,21 | 15 | 12 |
| 185 | 1 | 0.0011 | 18 | 12 | 18 | 29 | 15 | 11 | 25 | 13 | 11 | 10 | 14 | 18 | 19 | 23 | 11 | 14 | 11 | 13 | 15 | 13,13 | 16 | 12 |
| 186 | 1 | 0.0011 | 18 | 12 | 18 | 28 | 17 | 10 | 24 | 11 | 11 | 10 | 14 | 18 | 21 | 25 | 11 | 13 | 11 | 12 | 19 | 12,18 | 14 | 11 |
| 187 | 1 | 0.0011 | 19 | 13 | 19 | 28 | 17 | 10 | 24 | 12 | 11 | 10 | 14 | 18 | 23 | 25 | 13 | 13 | 10 | 12 | 18 | 13,19 | 14 | 13 |
| 188 | 1 | 0.0011 | 18 | 12 | 19 | 28 | 13 | 10 | 22 | 13 | 12 | 10 | 15 | 18 | 21 | 26 | 11 | 12 | 9 | 13 | 17 | 12,12 | 14 | 12 |
| 189 | 1 | 0.0011 | 20 | 14 | 19 | 31 | 15 | 10 | 22 | 12 | 11 | 10 | 15 | 17 | 19 | 23 | 12 | 13 | 11 | 12 | 17 | 11,16 | 16 | 12 |
| 190 | 1 | 0.0011 | 20 | 12 | 19 | 28 | 15 | 10 | 21 | 12 | 11 | 10 | 14 | 16 | 19 | 23 | 12 | 13 | 11 | 12 | 18 | 12,17 | 16 | 12 |
| 191 | 1 | 0.0011 | 20 | 13 | 19 | 31 | 16 | 10 | 23 | 11 | 12 | 10 | 14 | 17 | 21 | 23 | 11 | 13 | 10 | 12 | 16 | 10,19 | 15 | 12 |
| 192 | 1 | 0.0011 | 18 | 12 | 20 | 28 | 15 | 10 | 23 | 13 | 11 | 10 | 14 | 19 | 24 | 25 | 11 | 13 | 9 | 12 | 16 | 14,18 | 14 | 13 |
| 193 | 1 | 0.0011 | 17 | 12 | 21 | 28 | 15 | 7 | 25 | 12 | 11 | 10 | 15 | 19 | 21 | 26 | 12 | 13 | 11 | 12 | 16 | 13,19 | 15 | 12 |
| 194 | 1 | 0.0011 | 20 | 14 | 19 | 30 | 17 | 10 | 24 | 12 | 11 | 10 | 14 | 19 | 22 | 25 | 12 | 13 | 11 | 12 | 18 | 12,19 | 14 | 11 |
| 195 | 1 | 0.0011 | 17 | 13 | 19 | 28 | 16 | 10 | 23 | 14 | 11 | 10 | 16 | 18 | 20 | 22 | 11 | 14 | 10 | 12 | 17 | 11,12 | 16 | 12 |
| 196 | 1 | 0.0011 | 18 | 12 | 20 | 27 | 14 | 10 | 23 | 13 | 11 | 11 | 14 | 18 | 21 | 23 | 11 | 14 | 10 | 12 | 17 | 14,19 | 15 | 12 |
| 197 | 1 | 0.0011 | 18 | 13 | 21 | 30 | 17 | 11 | 24 | 13 | 11 | 10 | 14 | 17 | 21 | 23 | 12 | 11 | 9 | 15 | 16 | 11,19 | 17 | 11 |
| 198 | 1 | 0.0011 | 17 | 14 | 21 | 30 | 14 | 10 | 24 | 12 | 12 | 10 | 14 | 17 | 21 | 23 | 11 | 11 | 8 | 14 | 15 | 11,19 | 15 | 11 |
| 199 | 1 | 0.0011 | 16 | 12 | 17 | 29 | 15 | 10 | 24 | 12 | 10 | 10 | 14 | 20 | 21 | 23 | 12 | 12 | 12 | 14 | 14 | 12,14 | 16 | 11 |
| 200 | 1 | 0.0011 | 20 | 12 | 19 | 27 | 15 | 10 | 21 | 12 | 11 | 10 | 14 | 16 | 19 | 23 | 12 | 12 | 11 | 12 | 19 | 12,17 | 16 | 12 |
| 201 | 1 | 0.0011 | 18 | 13 | 20 | 31 | 16 | 11 | 23 | 11 | 12 | 11 | 14 | 18 | 23 | 25 | 11 | 11 | 10 | 13 | 15 | 11,14 | 15 | 12 |
| 202 | 1 | 0.0011 | 21 | 12 | 19 | 29 | 15 | 11 | 22 | 12 | 11 | 10 | 16 | 16 | 19 | 24 | 14 | 12 | 11 | 12 | 19 | 12,16 | 15 | 12 |
| 203 | 1 | 0.0011 | 18 | 13 | 21 | 30 | 16 | 11 | 24 | 13 | 11 | 10 | 14 | 17 | 21 | 23 | 12 | 11 | 9 | 15 | 17 | 11,17 | 16 | 11 |
| 204 | 1 | 0.0011 | 18 | 12 | 19 | 28 | 15 | 10 | 22 | 13 | 11 | 10 | 15 | 17 | 20 | 23 | 12 | 14 | 13 | 12 | 16 | 12,17 | 15 | 12 |
| 205 | 1 | 0.0011 | 18 | 13 | 22 | 31 | 15 | 11 | 27 | 12 | 12 | 10 | 14 | 16 | 21 | 23 | 11 | 11 | 8 | 15 | 15 | 11,12 | 15 | 11 |
| 206 | 1 | 0.0011 | 20 | 14 | 21 | 30 | 15 | 10 | 25 | 12 | 13 | 10 | 14 | 16 | 21 | 23 | 11 | 11 | 10 | 15 | 15 | 11,19 | 16 | 11 |
| 207 | 1 | 0.0011 | 17 | 12 | 20 | 27 | 14 | 11 | 21 | 12 | 11 | 11 | 15 | 18 | 20 | 24 | 12 | 14 | 11 | 12 | 16 | 14,16 | 15 | 12 |
| 208 | 1 | 0.0011 | 17 | 12 | 20 | 27 | 14 | 10 | 23 | 12 | 12 | 11 | 15 | 18 | 20 | 25 | 13 | 14 | 11 | 12 | 18 | 13,19 | 15 | 11 |
| 209 | 1 | 0.0011 | 20 | 12 | 19 | 27 | 15 | 10 | 23 | 13 | 12 | 10 | 15 | 16 | 20 | 23 | 12 | 12 | 10 | 12 | 19 | 12,16 | 15 | 12 |
| 210 | 1 | 0.0011 | 19 | 12 | 18 | 26 | 15 | 11 | 26 | 13 | 11 | 10 | 14 | 19 | 19 | 23 | 11 | 14 | 11 | 13 | 15 | 13,14 | 17 | 12 |
| 211 | 1 | 0.0011 | 15 | 12 | 18 | 31 | 15 | 10 | 25 | 12 | 11 | 10 | 14 | 18 | 20 | 23 | 12 | 14 | 11 | 13 | 15 | 12,13 | 15 | 12 |
| 212 | 1 | 0.0011 | 19 | 12 | 18 | 29 | 16 | 11 | 25 | 12 | 11 | 10 | 14 | 19 | 19 | 23 | 11 | 14 | 11 | 13 | 15 | 13,13 | 16 | 12 |
| 213 | 1 | 0.0011 | 17 | 12 | 19 | 28 | 15 | 10 | 25 | 12 | 11 | 10 | 14 | 18 | 20 | 22 | 11 | 14 | 12 | 13 | 15 | 13,14 | 16 | 12 |
| 214 | 1 | 0.0011 | 17 | 14 | 20 | 30 | 16 | 10 | 24 | 12 | 12 | 10 | 14 | 16 | 20 | 24 | 11 | 11 | 9 | 13 | 18 | 11,18 | 15 | 12 |
| 215 | 1 | 0.0011 | 18 | 12 | 20 | 30 | 15 | 10 | 25 | 13 | 11 | 10 | 15 | 18 | 21 | 25 | 11 | 13 | 11 | 12 | 15 | 13,21 | 14 | 12 |
| 216 | 1 | 0.0011 | 17 | 13 | 18 | 31 | 15 | 10 | 24 | 12 | 11 | 10 | 14 | 17 | 20 | 24 | 11 | 14 | 11 | 13 | 15 | 13,13 | 16 | 10 |
| 217 | 1 | 0.0011 | 18 | 14 | 18 | 31 | 15 | 10 | 23 | 12 | 11 | 10 | 14 | 20 | 21 | 24 | 11 | 13 | 11 | 14 | 15 | 12,17 | 15 | 11 |
| 218 | 1 | 0.0011 | 18 | 13 | 18 | 29 | 15 | 11 | 24 | 13 | 11 | 10 | 14 | 19 | 21 | 23 | 12 | 14 | 11 | 13 | 14 | 12,13 | 15 | 11 |
| 219 | 1 | 0.0011 | 17 | 12 | 18 | 28 | 15 | 10 | 24 | 12 | 11 | 10 | 14 | 18 | 20 | 23 | 11 | 14 | 11 | 12 | 15 | 13,13 | 16 | 11 |
| 220 | 1 | 0.0011 | 19 | 12 | 20 | 28 | 15 | 10 | 22 | 12 | 12 | 10 | 14 | 16 | 21 | 22 | 12 | 12 | 10 | 13 | 17 | 12,17 | 15 | 12 |
| 221 | 1 | 0.0011 | 18 | 12 | 20 | 28 | 15 | 10 | 22 | 12 | 11 | 11 | 15 | 19 | 23 | 23 | 11 | 12 | 11 | 12 | 19 | 11,12 | 15 | 12 |
| 222 | 1 | 0.0011 | 20 | 12 | 19 | 28 | 15 | 10 | 24 | 12 | 12 | 10 | 15 | 15 | 20 | 23 | 12 | 12 | 11 | 12 | 19 | 12,17 | 15 | 11 |
| 223 | 1 | 0.0011 | 19 | 12 | 20 | 28 | 15 | 10 | 24 | 12 | 12 | 11 | 15 | 18 | 20 | 25 | 13 | 14 | 11 | 12 | 18 | 14,18 | 15 | 12 |
| 224 | 1 | 0.0011 | 21 | 12 | 19 | 27 | 15 | 10 | 24 | 12 | 12 | 10 | 15 | 17 | 21 | 24 | 12 | 13 | 11 | 14 | 18 | 14,17 | 16 | 12 |
| 225 | 1 | 0.0011 | 19 | 13 | 19 | 30 | 15 | 10 | 23 | 12 | 11 | 10 | 15 | 19 | 21 | 22 | 12 | 13 | 10 | 12 | 17 | 11,11 | 16 | 11 |
| 226 | 1 | 0.0011 | 19 | 14 | 19 | 29 | 13 | 9 | 23 | 12 | 12 | 11 | 14 | 21 | 22 | 25 | 11 | 14 | 10 | 14 | 16 | 15,23 | 17 | 10 |
| 227 | 1 | 0.0011 | 19 | 13 | 18 | 29 | 16 | 10 | 25 | 12 | 10 | 10 | 14 | 19 | 19 | 23 | 12 | 14 | 12 | 13 | 16 | 13,13 | 17 | 10 |
| 228 | 1 | 0.0011 | 21 | 12 | 18 | 29 | 15 | 10 | 25 | 11 | 10 | 10 | 14 | 17 | 19 | 25 | 11 | 14 | 11 | 13 | 15 | 13,13 | 17 | 13 |
| 229 | 1 | 0.0011 | 20 | 12 | 20 | 28 | 15 | 10 | 23 | 13 | 11 | 11 | 15 | 18 | 22 | 23 | 12 | 14 | 11 | 12 | 16 | 14,17 | 15 | 12 |
| 230 | 1 | 0.0011 | 18 | 12 | 19 | 28 | 15 | 10 | 22 | 12 | 11 | 10 | 15 | 17 | 20 | 23 | 12 | 12 | 11 | 12 | 18 | 13,16 | 16 | 12 |
| 231 | 1 | 0.0011 | 18 | 13 | 19 | 29 | 14 | 10 | 22 | 12 | 12 | 10 | 14 | 17 | 21 | 23 | 11 | 14 | 12 | 13 | 17 | 12,13 | 15 | 12 |
| 232 | 1 | 0.0011 | 18 | 13 | 21 | 30 | 16 | 10 | 23 | 13 | 14 | 10 | 14 | 17 | 22 | 23 | 11 | 11 | 9 | 15 | 16 | 11,17 | 15 | 11 |
| 233 | 1 | 0.0011 | 18 | 12 | 18 | 28 | 15 | 11 | 24 | 13 | 11 | 10 | 14 | 19 | 21 | 23 | 12 | 14 | 11 | 13 | 15 | 13,13 | 15 | 12 |
| 234 | 1 | 0.0011 | 20 | 12 | 19 | 29 | 15 | 10 | 25 | 12 | 11 | 10 | 14 | 19 | 21 | 23 | 12 | 14 | 11 | 12 | 16 | 13,13 | 15 | 12 |
| 235 | 1 | 0.0011 | 19 | 14 | 18 | 31 | 16 | 10 | 24 | 12 | 12 | 13 | 14 | 17 | 20 | 23 | 12 | 13 | 12 | 13 | 18 | 10,18 | 15 | 11 |
| 236 | 1 | 0.0011 | 17 | 13 | 19 | 28 | 15 | 11 | 22 | 13 | 11 | 10 | 14 | 17 | 21 | 22 | 12 | 12 | 12 | 12 | 17 | 1,17 | 16 | 12 |
| 237 | 1 | 0.0011 | 18 | 13 | 20 | 29 | 16 | 9 | 23 | 11 | 11 | 11 | 15 | 19 | 20 | 24 | 11 | 15 | 12 | 12 | 18 | 13,19 | 15 | 12 |
| 238 | 1 | 0.0011 | 17 | 12 | 19 | 28 | 13 | 10 | 24 | 12 | 11 | 10 | 14 | 19 | 23 | 25 | 13 | 14 | 10 | 12 | 17 | 12,18 | 14 | 12 |
| 239 | 1 | 0.0011 | 17 | 12 | 20 | 30 | 17 | 10 | 24 | 13 | 11 | 10 | 14 | 17 | 23 | 25 | 12 | 13 | 10 | 12 | 21 | 14,19 | 14 | 12 |
| 240 | 1 | 0.0011 | 18 | 12 | 20 | 28 | 14 | 10 | 23 | 12 | 11 | 11 | 15 | 18 | 21 | 23 | 11 | 14 | 11 | 12 | 17 | 13,17 | 16 | 12 |
| 241 | 1 | 0.0011 | 19 | 13 | 17 | 29 | 15 | 10 | 22 | 10 | 11 | 10 | 14 | 18 | 20 | 23 | 13 | 13 | 11 | 14 | 16 | 10,18 | 15 | 11 |
| 242 | 1 | 0.0011 | 19 | 12 | 20 | 28 | 14 | 10 | 23 | 13 | 11 | 11 | 14 | 18 | 20 | 23 | 11 | 15 | 11 | 12 | 17 | 13,19 | 15 | 12 |
| 243 | 1 | 0.0011 | 18 | 12 | 18 | 26 | 16 | 11 | 21 | 13 | 12 | 10 | 14 | 18 | 20 | 23 | 12 | 14 | 11 | 13 | 15 | 12,13 | 18 | 11 |
| 244 | 1 | 0.0011 | 18 | 12 | 21 | 28 | 16 | 10 | 23 | 12 | 11 | 10 | 15 | 21 | 22 | 23 | 12 | 13 | 13 | 12 | 16 | 12,22 | 15 | 12 |
| 245 | 1 | 0.0011 | 19 | 12 | 19 | 28 | 15 | 9 | 26 | 13 | 11 | 10 | 14 | 17 | 22 | 25 | 11 | 13 | 10 | 12 | 17 | 12,21 | 14 | 12 |
| 246 | 1 | 0.0011 | 18 | 12 | 20 | 28 | 16 | 10 | 24 | 13 | 11 | 9 | 14 | 19 | 21 | 25 | 12 | 13 | 10 | 12 | 19 | 12,18 | 14 | 12 |
| 247 | 1 | 0.0011 | 16 | 12 | 18 | 29 | 15 | 10 | 26 | 12 | 11 | 10 | 14 | 18 | 20 | 22 | 10 | 14 | 11 | 13 | 14 | 13,14 | 14 | 12 |
| 248 | 1 | 0.0011 | 18 | 14 | 20 | 29 | 14 | 11 | 23 | 13 | 10 | 10 | 14 | 19 | 21 | 25 | 13 | 13 | 12 | 12 | 16 | 13,18 | 16 | 11 |
| 249 | 1 | 0.0011 | 20 | 12 | 19 | 28 | 16 | 10 | 26 | 13 | 12 | 10 | 14 | 17 | 21 | 25 | 11 | 13 | 10 | 12 | 16 | 12,20 | 14 | 13 |
| 250 | 1 | 0.0011 | 18 | 12 | 19 | 27 | 15 | 10 | 25 | 12 | 12 | 10 | 15 | 16 | 23 | 24 | 13 | 13 | 12 | 13 | 17 | 13,18 | 15 | 12 |
| 251 | 1 | 0.0011 | 17 | 12 | 20 | 28 | 14 | 10 | 22 | 13 | 11 | 11 | 14 | 19 | 20 | 23 | 11 | 14 | 11 | 12 | 17 | 14,18 | 14 | 12 |
| 252 | 1 | 0.0011 | 17 | 12 | 19 | 28 | 16 | 10 | 25 | 12 | 11 | 10 | 14 | 18 | 21 | 25 | 13 | 13 | 10 | 12 | 17 | 12,20 | 14 | 12 |
| 253 | 1 | 0.0011 | 18 | 14 | 19 | 30 | 14 | 10 | 22 | 12 | 12 | 10 | 14 | 22 | 21 | 22 | 9 | 14 | 9 | 15 | **16.1** | 10,12 | 15 | 10 |
| 254 | 1 | 0.0011 | 18 | 12 | 18 | 30 | 15 | 10 | 23 | 11 | 10 | 10 | 14 | 17 | 23 | 24 | 11 | 13 | 12 | 13 | 17 | 14,18 | 15 | 10 |
| 255 | 1 | 0.0011 | 19 | 12 | 20 | 30 | 15 | 9 | 26 | 12 | 14 | 10 | 15 | 14 | 22 | 24 | 10 | 13 | 12 | 12 | 15 | 12,17 | 15 | 12 |
| 256 | 1 | 0.0011 | 17 | 14 | 21 | 30 | 17 | 10 | 24 | 12 | 11 | 10 | 15 | 17 | 21 | 24 | 12 | 13 | 11 | 12 | 16 | 14,20 | 13 | 12 |
| 257 | 1 | 0.0011 | 17 | 13 | 21 | 29 | 15 | 10 | 26 | 13 | 12 | 10 | 14 | 17 | 22 | 23 | 11 | 11 | 9 | 15 | 17 | 11,16 | 15 | 11 |
| 258 | 1 | 0.0011 | 17 | 12 | 18 | 28 | 15 | 11 | 25 | 12 | 11 | 10 | 14 | 18 | 19 | 23 | 11 | 14 | 11 | 13 | 15 | 13,13 | 17 | 12 |
| 259 | 1 | 0.0011 | 19 | 12 | 20 | 28 | 14 | 10 | 24 | 12 | 11 | 11 | 15 | 17 | 20 | 24 | 12 | 14 | 12 | 12 | 17 | 13,18 | 16 | 13 |
| 260 | 1 | 0.0011 | 18 | 12 | 19 | 28 | 15 | 10 | 22 | 14 | 12 | 10 | 15 | 17 | 20 | 23 | 12 | 12 | 11 | 12 | 17 | 12,18 | 15 | 12 |
| 261 | 1 | 0.0011 | 18 | 14 | 20 | 31 | 16 | 10 | 24 | 12 | 12 | 10 | 14 | 16 | 20 | 23 | 12 | 11 | 9 | 15 | 16 | 11,18 | 16 | 11 |
| 262 | 1 | 0.0011 | 17 | 12 | 20 | 27 | 14 | 10 | 23 | 13 | 9 | 11 | 15 | 19 | 21 | 24 | 12 | 15 | 11 | 12 | 17 | 13,15 | 15 | 12 |
| 263 | 1 | 0.0011 | 17 | 14 | 17 | 29 | 14 | 10 | 25 | 12 | 12 | 10 | 14 | 19 | 22 | 22 | 10 | 14 | 11 | 13 | 19 | 11,12 | 14 | 11 |
| 264 | 1 | 0.0011 | 17 | 12 | 20 | 29 | 16 | 10 | 22 | 12 | 11 | 10 | 15 | 18 | 20 | 23 | 12 | 13 | 11 | 13 | 17 | 11,11 | 16 | 12 |
| 265 | 1 | 0.0011 | 19 | 12 | 19 | 27 | 16 | 10 | 27 | 14 | 11 | 10 | 14 | 17 | 20 | 25 | 13 | 13 | 10 | 12 | 18 | 12,20 | 14 | 12 |
| 266 | 1 | 0.0011 | 18 | 13 | 19 | 28 | 15 | 10 | 29 | 12 | 11 | 10 | 15 | 18 | 20 | 24 | 13 | 14 | 12 | 12 | 18 | 13,20 | 15 | 11 |
| 267 | 1 | 0.0011 | 18 | 12 | 17 | 30 | 15 | 10 | 24 | 13 | 11 | 10 | 14 | 16 | 20 | 23 | 11 | 14 | 11 | 13 | 15 | 13,13 | 16 | 10 |
| 268 | 1 | 0.0011 | 19 | 12 | 20 | 28 | 14 | 10 | 23 | 13 | 12 | 11 | 16 | 18 | 20 | 25 | 13 | 14 | 11 | 12 | 19 | 17,19 | 15 | 12 |
| 269 | 1 | 0.0011 | 16 | 12 | 18 | 29 | 15 | 10 | 23 | 12 | 11 | 10 | 14 | 19 | 19 | 23 | 11 | 14 | 11 | 13 | 16 | 13,13 | 18 | 12 |
| 270 | 1 | 0.0011 | 18 | 12 | 18 | 29 | 15 | 11 | 25 | 12 | 11 | 10 | 14 | 19 | 19 | 23 | 11 | 14 | 11 | 13 | 15 | 13,13 | 17 | 12 |
| 271 | 1 | 0.0011 | 19 | 12 | 19 | 29 | 13 | 11 | 22 | 12 | 11 | 10 | 15 | 15 | 20 | 23 | 11 | 12 | 11 | 12 | 17 | 12,12 | 15 | 12 |
| 272 | 1 | 0.0011 | 16 | 12 | 19 | 28 | 16 | 10 | 25 | 12 | 12 | 10 | 13 | 20 | 22 | 23 | 11 | 13 | 11 | 12 | 19 | 15,16 | 14 | 12 |
| 273 | 1 | 0.0011 | 16 | 12 | 18 | 27 | 16 | 11 | 21 | 12 | 11 | 10 | 14 | 18 | 20 | 23 | 12 | 14 | 11 | 13 | 15 | 12,13 | 17 | 12 |
| 274 | 1 | 0.0011 | 18 | 13 | 18 | 30 | 17 | 10 | 24 | 12 | 10 | 10 | 14 | 18 | 21 | 25 | 12 | 13 | 13 | 13 | 16 | 13,18 | 15 | 11 |
| 275 | 1 | 0.0011 | 19 | 14 | 22 | 29 | 16 | 10 | 25 | 12 | 12 | 10 | 14 | 14 | 21 | 23 | 11 | 11 | 9 | 15 | 16 | 10,19 | 15 | 11 |
| 276 | 1 | 0.0011 | 17 | 15 | 18 | 33 | 16 | 10 | 24 | 13 | 12 | 10 | 14 | 18 | 21 | 23 | 12 | 13 | 10 | 12 | 17 | 11,20 | 16 | 12 |
| 277 | 1 | 0.0011 | 19 | 13 | 19 | 31 | 17 | 10 | 24 | 13 | 12 | 10 | 15 | 17 | 21 | 22 | 12 | 13 | 10 | 12 | 19 | 11,18 | 15 | 12 |
| 278 | 1 | 0.0011 | 18 | 13 | 20 | 29 | 15 | 10 | 28 | 11 | 12 | 10 | 14 | 21 | 20 | 24 | 12 | 10 | 9 | 12 | 21 | 15,17 | 14 | 11 |
| 279 | 1 | 0.0011 | 20 | 12 | 19 | 27 | 15 | 11 | 24 | 13 | 11 | 10 | 15 | 17 | 20 | 23 | 12 | 12 | 11 | 13 | 19 | 12,16 | 15 | 11 |
| 280 | 1 | 0.0011 | 19 | 14 | 19 | 31 | 15 | 10 | 24 | 12 | 11 | 10 | 14 | 19 | 21 | 23 | 11 | 13 | 11 | 14 | 15 | 12,19 | 15 | 11 |
| 281 | 1 | 0.0011 | 18 | 13 | 19 | 28 | 16 | 10 | 24 | 12 | 12 | 10 | 14 | 18 | 24 | 23 | 12 | 13 | 10 | 12 | 18 | 13,20 | 14 | 13 |
| 282 | 1 | 0.0011 | 17 | 14 | 21 | 30 | 16 | 10 | 24 | 12 | 14 | 10 | 14 | 17 | 21 | 23 | 11 | 11 | 10 | 15 | 16 | 11,17 | 15 | 11 |
| 283 | 1 | 0.0011 | 18 | 12 | 20 | 28 | 14 | 10 | 24 | 13 | 11 | 11 | 15 | 16 | 20 | 24 | 11 | 14 | 11 | 13 | 19 | 13,17 | 15 | 12 |
| 284 | 1 | 0.0011 | 20 | 12 | 19 | 29 | 15 | 10 | 22 | 13 | 11 | 10 | 14 | 16 | 19 | 23 | 12 | 12 | 11 | 13 | 16 | 13,17 | 15 | 13 |
| 285 | 1 | 0.0011 | 18 | 13 | 19 | 29 | 15 | 10 | 28 | 11 | 12 | 10 | 14 | 19 | 21 | 24 | 11 | 10 | 9 | 12 | 23 | 16,16 | 15 | 11 |
| 286 | 1 | 0.0011 | 17 | 12 | 19 | 29 | 16 | 11 | 24 | 14 | 12 | 10 | 15 | 19 | 23 | 24 | 11 | 12 | 11 | 12 | 15 | 13,19 | 15 | 12 |
| 287 | 1 | 0.0011 | 16 | 12 | 19 | 28 | 16 | 10 | 26 | 12 | 11 | 10 | 14 | 19 | 22 | 24 | 12 | 13 | 10 | 12 | 17 | 14,17 | 14 | 11 |
| 288 | 1 | 0.0011 | 19 | 13 | 19 | 30 | 16 | 10 | 23 | 12 | 11 | 10 | 14 | 18 | 21 | 22 | 13 | 13 | 10 | 12 | 18 | 11,20 | 16 | 11 |
| 289 | 1 | 0.0011 | 19 | 12 | 19 | 28 | 17 | 11 | 25 | 13 | 11 | 10 | 14 | 17 | 21 | 25 | 13 | 13 | 11 | 12 | 18 | 14,19 | 15 | 12 |
| 290 | 1 | 0.0011 | 17 | 13 | 19 | 29 | 15 | 10 | 28 | 11 | 12 | 10 | 14 | 20 | 21 | 24 | 11 | 10 | 9 | 12 | 23 | 16,16 | 15 | 11 |
| 291 | 1 | 0.0011 | 16 | 12 | 20 | 29 | 16 | 10 | 26 | 13 | 11 | 10 | 14 | 20 | 22 | 25 | 11 | 13 | 10 | 12 | 20 | 14,19 | 14 | 11 |
| 292 | 1 | 0.0011 | 16 | 13 | 19 | 27 | 14 | 11 | 24 | 12 | 12 | 10 | 14 | 19 | 21 | 24 | 11 | 15 | 11 | 13 | 16 | 11,12 | 15 | 12 |
| 293 | 1 | 0.0011 | 17 | 13 | 19 | 28 | 15 | 11 | 22 | 12 | 11 | 10 | 14 | 17 | 22 | 23 | 11 | 12 | 12 | 13 | 17 | 14,18 | 16 | 13 |
| 294 | 1 | 0.0011 | 18 | 14 | 19 | 30 | 13 | 9 | 26 | 12 | 12 | 12 | 14 | 19 | 22 | 24 | 10 | 14 | 10 | 14 | 17 | 15,19 | 15 | 10 |
| 295 | 1 | 0.0011 | 19 | 12 | 19 | 28 | 15 | 11 | 25 | 12 | 11 | 10 | 14 | 18 | 21 | 24 | 9 | 13 | 10 | 12 | 19 | 12,19 | 14 | 11 |
| 296 | 1 | 0.0011 | 16 | 12 | 20 | 28 | 17 | 10 | 24 | 13 | 11 | 11 | 14 | 19 | 22 | 25 | 11 | 13 | 10 | 12 | 19 | 17,18 | 14 | 13 |
| 297 | 1 | 0.0011 | 18 | 14 | 19 | 30 | 14 | 11 | 21 | 12 | 13 | 10 | 14 | 19 | 20 | 23 | 10 | 14 | 11 | 13 | 16 | 11,12 | 14 | 11 |
| 298 | 1 | 0.0011 | 17 | 12 | 19 | 28 | 17 | 11 | 24 | 13 | 12 | 10 | 15 | 19 | 22 | 24 | 11 | 12 | 11 | 12 | 15 | 13,20 | 15 | 12 |
| 299 | 1 | 0.0011 | 19 | 14 | 18 | 30 | 17 | 10 | 21 | 12 | 11 | 13 | 14 | 18 | 20 | 23 | 11 | 13 | 12 | 13 | 17 | 10,21 | 16 | 11 |
| 300 | 1 | 0.0011 | 20 | 12 | 19 | 28 | 15 | 11 | 22 | 15 | 11 | 11 | 15 | 14 | 20 | 23 | 11 | 12 | 11 | 12 | 17 | 12,16 | 15 | 12 |
| 301 | 1 | 0.0011 | 17 | 12 | 19 | 28 | 16 | 10 | 25 | 13 | 11 | 10 | 14 | 19 | 22 | 25 | 12 | 13 | 12 | 12 | 21 | 13,18 | 14 | 11 |
| 302 | 1 | 0.0011 | 18 | 12 | 19 | 29 | 15 | 10 | 22 | 11 | 11 | 10 | 15 | 17 | 21 | 23 | 13 | 12 | 12 | 12 | 20 | 12,15 | 15 | 12 |
| 303 | 1 | 0.0011 | 16 | 13 | 21 | 29 | 15 | 11 | 24 | 13 | 12 | 10 | 14 | 16 | 21 | 23 | 10 | 11 | 10 | 15 | 19 | 11,18 | 16 | 12 |
| 304 | 1 | 0.0011 | 18 | 14 | 20 | 30 | 15 | 10 | 24 | 11 | 12 | 10 | 14 | 17 | 22 | 23 | 11 | 13 | 11 | 14 | 17 | 13,17 | 14 | 12 |
| 305 | 1 | 0.0011 | 19 | 13 | 18 | 30 | 15 | 11 | 21 | 13 | 12 | 10 | 14 | 16 | 22 | 22 | 11 | 13 | 11 | 12 | 16 | 12,19 | 15 | 11 |
| 306 | 1 | 0.0011 | 18 | 12 | 20 | 30 | 15 | 10 | 24 | 12 | 11 | 11 | 15 | 18 | 20 | 25 | 11 | 14 | 11 | 12 | 17 | 13,19 | 15 | 12 |
| 307 | 1 | 0.0011 | 17 | 13 | 21 | 30 | 16 | 10 | 23 | 12 | 12 | 10 | 14 | 17 | 24 | 23 | 11 | 11 | 9 | 15 | 18 | 11,17 | 15 | 11 |
| 308 | 1 | 0.0011 | 16 | 13 | 18 | 30 | 15 | 10 | 23 | 11 | 11 | 10 | 14 | 21 | 21 | 24 | 11 | 13 | 11 | 14 | 15 | 12,17 | 15 | 12 |
| 309 | 1 | 0.0011 | 20 | 12 | 19 | 29 | 14 | 11 | 22 | 12 | 11 | 10 | 15 | 17 | 20 | 23 | 13 | 12 | 11 | 12 | 18 | 12,17 | 16 | 12 |
| 310 | 1 | 0.0011 | 19 | 12 | 18 | 28 | 16 | 11 | 25 | 12 | 11 | 10 | 14 | 16 | 20 | 23 | 10 | 14 | 11 | 13 | 15 | 13,13 | 17 | 12 |
| 311 | 1 | 0.0011 | 19 | 12 | 19 | 29 | 15 | 10 | 18 | 12 | 11 | 10 | 14 | 16 | 19 | 24 | 12 | 12 | 11 | 12 | 18 | 12,17 | 15 | 12 |
| 312 | 1 | 0.0011 | 18 | 12 | 20 | 28 | 14 | 10 | 23 | 12 | 11 | 11 | 14 | 18 | 21 | 24 | 13 | 14 | 11 | 12 | 16 | 12,17 | 15 | 12 |
| 313 | 1 | 0.0011 | 21 | 12 | 20 | 28 | 14 | 10 | 22 | 12 | 11 | 12 | 15 | 17 | 20 | 23 | 12 | 14 | 11 | 12 | 18 | 13,19 | 16 | 12 |
| 314 | 1 | 0.0011 | 18 | 13 | 22 | 29 | 15 | 10 | 25 | 12 | 11 | 10 | 14 | 16 | 23 | 23 | 11 | 11 | 9 | 15 | 17 | 11,18 | 15 | 12 |
| 315 | 1 | 0.0011 | 18 | 12 | 20 | 28 | 14 | 10 | 23 | 13 | 11 | 11 | 14 | 18 | 20 | 23 | 11 | 14 | 11 | 12 | 17 | 13,19 | 15 | 12 |
| 316 | 1 | 0.0011 | 19 | 12 | 20 | 28 | 16 | 11 | 25 | 13 | 11 | 10 | 15 | 18 | 20 | 24 | 13 | 13 | 11 | 12 | 18 | 13,18 | 15 | 12 |
| 317 | 1 | 0.0011 | 18 | 13 | 18 | 30 | 17 | 10 | 24 | 12 | 10 | 10 | 14 | 19 | 22 | 25 | 11 | 13 | 12 | 13 | 17 | 13,18 | 15 | 11 |
| 318 | 1 | 0.0011 | 22 | 12 | 19 | 29 | 15 | 10 | 22 | 12 | 12 | 10 | 15 | 17 | 19 | 23 | 12 | 7 | 11 | 12 | 16 | 12,16 | 15 | 12 |
| 319 | 1 | 0.0011 | 19 | 13 | 19 | 29 | 14 | 10 | 23 | 12 | 12 | 11 | 14 | 18 | 22 | 23 | 11 | 14 | 10 | 13 | 16 | 11,12 | 15 | 12 |
| 320 | 1 | 0.0011 | 19 | 12 | 20 | 29 | 16 | 10 | 23 | 13 | 11 | 10 | 15 | 19 | 21 | 25 | 12 | 13 | 13 | 12 | 19 | 15,20 | 15 | 12 |
| 321 | 1 | 0.0011 | 16 | 12 | 18 | 28 | 15 | 10 | 26 | 13 | 11 | 10 | 14 | 21 | 20 | 22 | 11 | 14 | 11 | 13 | 15 | 13,14 | 15 | 12 |
| 322 | 1 | 0.0011 | 20 | 14 | 18 | 30 | 13 | 9 | 23 | 12 | 12 | 12 | 14 | 20 | 23 | 24 | 11 | 15 | 10 | 14 | 16 | 15,22 | 16 | 10 |
| 323 | 1 | 0.0011 | 17 | 12 | 18 | 28 | 15 | 10 | 24 | 14 | 11 | 10 | 14 | 19 | 21 | 23 | 12 | 14 | 11 | 13 | 14 | 12,13 | 14 | 12 |
| 324 | 1 | 0.0011 | 18 | 12 | 18 | 29 | 16 | 11 | 24 | 12 | 11 | 10 | 14 | 18 | 19 | 23 | 11 | 14 | 11 | 13 | 15 | 13,13 | 17 | 12 |
| 325 | 1 | 0.0011 | 18 | 14 | 21 | 30 | 15 | 10 | 23 | 11 | 11 | 9 | 14 | 18 | 22 | 24 | 12 | 12 | 10 | 12 | 17 | 15,21 | 15 | 11 |
| 326 | 1 | 0.0011 | 19 | 13 | 20 | 29 | 14 | 10 | 23 | 12 | 10 | 10 | 15 | 20 | 21 | 24 | 14 | 13 | 12 | 12 | 16 | 13,20 | 15 | 10 |
| 327 | 1 | 0.0011 | 18 | 13 | 21 | 29 | 17 | 10 | 24 | 13 | 12 | 10 | 14 | 16 | 21 | 23 | 11 | 11 | 9 | 14 | 16 | 11,15 | 15 | 11 |
| 328 | 1 | 0.0011 | 16 | 14 | 21 | 33 | 13 | 11 | 25 | 12 | 10 | 10 | 14 | 20 | 23 | 25 | 12 | 11 | 12 | 12 | 18 | 15,17 | 15 | 11 |
| 329 | 1 | 0.0011 | 18 | 13 | 19 | 29 | 16 | 10 | 24 | 12 | 13 | 10 | 14 | 15 | 22 | 23 | 13 | 11 | 9 | 14 | 18 | 11,19 | 15 | 11 |
| 330 | 1 | 0.0011 | 18 | 12 | 20 | 28 | 15 | 10 | 23 | 12 | 11 | 11 | 15 | 18 | 21 | 25 | 13 | 14 | 11 | 12 | 16 | 14,17 | 14 | 12 |
| 331 | 1 | 0.0011 | 21 | 12 | 19 | 28 | 15 | 10 | 21 | 12 | 11 | 10 | 15 | 17 | 19 | 23 | 12 | 12 | 11 | 12 | 18 | 12,16 | 16 | 12 |
| 332 | 1 | 0.0011 | 17 | 12 | 19 | 28 | 15 | 10 | 25 | 14 | 11 | 10 | 14 | 18 | 22 | 25 | 12 | 13 | 11 | 12 | 19 | 12,19 | 13 | 13 |
| 333 | 1 | 0.0011 | 19 | 14 | 18 | 31 | 15 | 10 | 25 | 12 | 10 | 10 | 14 | 18 | 19 | 23 | 12 | 14 | 11 | 13 | 15 | 13,13 | 16 | 12 |
| 334 | 1 | 0.0011 | 17 | 13 | 19 | 28 | 17 | 12 | 24 | 12 | 11 | 10 | 14 | 18 | 23 | 25 | 12 | 15 | 10 | 11 | 19 | 13,19 | 14 | 13 |
| 335 | 1 | 0.0011 | 17 | 13 | 18 | 28 | 17 | 10 | 24 | 12 | 11 | 10 | 14 | 16 | 22 | 25 | 11 | 13 | 12 | 14 | 16 | 13,18 | 15 | 11 |
| 336 | 1 | 0.0011 | 19 | 12 | 20 | 27 | 17 | 10 | 25 | 13 | 11 | 10 | 16 | 18 | 22 | 24 | 12 | 13 | 12 | 12 | 17 | 13,21 | 14 | 11 |
| 337 | 1 | 0.0011 | 17 | 13 | 21 | 29 | 15 | 11 | 27 | 13 | 12 | 10 | 14 | 15 | 21 | 23 | 11 | 11 | 8 | 14 | 15 | 11,19 | 15 | 11 |
| 338 | 1 | 0.0011 | 17 | 12 | 18 | 29 | 15 | 12 | 27 | 13 | 11 | 10 | 14 | 17 | 19 | 23 | 11 | 14 | 11 | 13 | 16 | 13,13 | 17 | 11 |
| 339 | 1 | 0.0011 | 19 | 13 | 20 | 30 | 14 | 10 | 23 | 11 | 11 | 11 | 15 | 17 | 20 | 24 | 12 | 14 | 12 | 12 | 20 | 13,18 | 15 | 12 |
| 340 | 1 | 0.0011 | 20 | 12 | 19 | 27 | 16 | 10 | 23 | 12 | 11 | 10 | 15 | 16 | 19 | 23 | 13 | 12 | 11 | 13 | 18 | 12,12 | 15 | 12 |
| 341 | 1 | 0.0011 | 18 | 12 | 20 | 30 | 16 | 10 | 24 | 13 | 11 | 10 | 15 | 20 | 21 | 25 | 11 | 13 | 12 | 12 | 17 | 13,20 | 13 | 13 |
| 342 | 1 | 0.0011 | 18 | 14 | 20 | 31 | 15 | 10 | 24 | 13 | 11 | 10 | 15 | 19 | 21 | 24 | 12 | 13 | 12 | 13 | 17 | 13,24 | 15 | 12 |
| 343 | 1 | 0.0011 | 18 | 14 | 19 | 31 | 15 | 10 | 22 | 13 | 12 | 10 | 14 | 17 | 21 | 22 | 10 | 13 | 11 | 12 | 16 | 12,20 | 15 | 11 |
| 344 | 1 | 0.0011 | 19 | 12 | 19 | 30 | 15 | 10 | 22 | 13 | 11 | 10 | 14 | 17 | 19 | 23 | 12 | 12 | 11 | 12 | 18 | 12,17 | 16 | 12 |
| 345 | 1 | 0.0011 | 16 | 12 | 20 | 27 | 15 | 9 | 24 | 12 | 12 | 10 | 14 | 19 | 23 | 24 | 11 | 13 | 10 | 12 | 17 | 14,19 | 14 | 10 |
| 346 | 1 | 0.0011 | 17 | 13 | 19 | 29 | 15 | 11 | 23 | 12 | 12 | 10 | 14 | 17 | 21 | 23 | 11 | 13 | 11 | 12 | 18 | 12,19 | 15 | 11 |
| 347 | 1 | 0.0011 | 18 | 12 | 18 | 29 | 15 | 11 | 25 | 12 | 11 | 10 | 14 | 17 | 19 | 24 | 12 | 14 | 11 | 13 | 15 | 13,13 | 17 | 12 |
| 348 | 1 | 0.0011 | 18 | 12 | 20 | 28 | 14 | 10 | 23 | 12 | 11 | 11 | 15 | 18 | 20 | 24 | 12 | 14 | 11 | 12 | 16 | 13,17 | 16 | 12 |
| 349 | 1 | 0.0011 | 20 | 12 | 20 | 28 | 15 | 10 | 23 | 12 | 11 | 11 | 15 | 17 | 20 | 24 | 12 | 14 | 11 | 12 | 16 | 13,17 | 14 | 12 |
| 350 | 1 | 0.0011 | 23 | 12 | 20 | 28 | 13 | 10 | 23 | 12 | 11 | 10 | 15 | 18 | 19 | 23 | 13 | 12 | 11 | 12 | 18 | 12,16 | 15 | 12 |
| 351 | 1 | 0.0011 | 19 | 14 | 19 | 31 | 14 | 10 | 23 | 13 | 12 | 10 | 14 | 17 | 21 | 23 | 10 | 14 | 12 | 13 | 14 | 12,15 | 15 | 11 |
| 352 | 1 | 0.0011 | 17 | 12 | 19 | 28 | 15 | 10 | 26 | 12 | 11 | 10 | 14 | 17 | 22 | 26 | 12 | 13 | 10 | 12 | 17 | 12,19 | 16 | 11 |
| 353 | 1 | 0.0011 | 18 | 12 | 19 | 28 | 15 | 10 | 23 | 12 | 11 | 11 | 15 | 16 | 22 | 24 | 12 | 12 | 11 | 12 | 15 | 13,17 | 15 | 12 |
| 354 | 1 | 0.0011 | 17 | 12 | 20 | 28 | 15 | 10 | 21 | 13 | 11 | 11 | 15 | 19 | 22 | 23 | 11 | 12 | 11 | 12 | 18 | 12,12 | 15 | 12 |
| 355 | 1 | 0.0011 | 17 | 13 | 19 | 30 | 14 | 10 | 24 | 13 | 12 | 10 | 14 | 17 | 21 | 23 | 11 | 15 | 12 | 13 | 15 | 12,13 | 15 | 12 |
| 356 | 1 | 0.0011 | 20 | 12 | 19 | 29 | 15 | 10 | 22 | 12 | 11 | 10 | 15 | 16 | 19 | 23 | 11 | 12 | 10 | 12 | 17 | 12,13 | 15 | 12 |
| 357 | 1 | 0.0011 | 17 | 12 | 18 | 29 | 15 | 11 | 25 | 12 | 11 | 10 | 14 | 19 | 19 | 23 | 11 | 14 | 11 | 13 | 15 | 13,13 | 17 | 12 |
| 358 | 1 | 0.0011 | 17 | 13 | 18 | 30 | 15 | 11 | 25 | 13 | 11 | 11 | 14 | 15 | 21 | 23 | 13 | 13 | 12 | 14 | 16 | 12,17 | 16 | 12 |
| 359 | 1 | 0.0011 | 19 | 13 | 19 | 28 | 15 | 10 | 23 | 12 | 11 | 10 | 15 | 18 | 21 | 22 | 11 | 14 | 10 | 12 | 16 | 11,11 | 16 | 12 |
| 360 | 1 | 0.0011 | 19 | 12 | 18 | 28 | 15 | 10 | 25 | 12 | 11 | 10 | 14 | 18 | 19 | 23 | 11 | 14 | 11 | 13 | 15 | 13,13 | 17 | 12 |
| 361 | 1 | 0.0011 | 17 | 12 | 18 | 28 | 15 | 11 | 25 | 11 | 11 | 10 | 14 | 18 | 19 | 23 | 11 | 14 | 11 | 13 | 15 | 13,13 | 17 | 12 |
| 362 | 1 | 0.0011 | 19 | 13 | 19 | 29 | 14 | 11 | 21 | 12 | 12 | 11 | 14 | 18 | 24 | 23 | 11 | 14 | 10 | 13 | 15 | 11,12 | 16 | 12 |
| 363 | 1 | 0.0011 | 17 | 14 | 19 | 30 | 14 | 10 | 23 | 14 | 13 | 12 | 15 | 20 | 23 | 25 | 14 | 13 | 10 | 12 | 16 | 12,14 | 15 | 12 |
| 364 | 1 | 0.0011 | 17 | 13 | 21 | 30 | 14 | 10 | 23 | 12 | 11 | 9 | 15 | 15 | 21 | 22 | 12 | 11 | 10 | 12 | 16 | 13,21 | 15 | 12 |
| 365 | 1 | 0.0011 | 19 | 13 | 19 | 30 | 15 | 10 | 23 | 12 | 11 | 10 | 14 | 16 | 22 | 24 | 11 | 13 | 11 | 13 | 17 | 12,20 | 15 | 11 |
| 366 | 1 | 0.0011 | 18 | 13 | 19 | 30 | 15 | 10 | 22 | 12 | 12 | 11 | 14 | 18 | 22 | 21 | 11 | 14 | 10 | 13 | 16 | 12,13 | 16 | 13 |
| 367 | 1 | 0.0011 | 17 | 12 | 20 | 27 | 16 | 11 | 25 | 13 | 11 | 11 | 13 | 19 | 23 | 24 | 11 | 13 | 10 | 12 | 17 | 14,18 | 14 | 12 |
| 368 | 1 | 0.0011 | 19 | 12 | 18 | 28 | 15 | 11 | 25 | 12 | 11 | 10 | 14 | 17 | 19 | 23 | 11 | 14 | 11 | 13 | 15 | 13,13 | 16 | 12 |
| 369 | 1 | 0.0011 | 20 | 12 | 20 | 27 | 14 | 10 | 24 | 12 | 11 | 12 | 14 | 18 | 20 | 23 | 12 | 14 | 10 | 12 | 19 | 13,13 | 14 | 12 |
| 370 | 1 | 0.0011 | 18 | 13 | 21 | 29 | 16 | 10 | 23 | 13 | 11 | 10 | 15 | 17 | 22 | 24 | 13 | 13 | 11 | 12 | 17 | 12,18 | 13 | 11 |
| 371 | 1 | 0.0011 | 16 | 13 | 21 | 29 | 15 | 10 | 22 | 12 | 13 | 10 | 14 | 15 | 20 | 23 | 10 | 11 | 9 | 14 | 15 | 11,19 | 15 | 12 |
| 372 | 1 | 0.0011 | 20 | 14 | 19 | 30 | 13 | 9 | 24 | 10 | 11 | 12 | 14 | 20 | 22 | 24 | 10 | 14 | 11 | 14 | 20 | 16,21 | 16 | 10 |
| 373 | 1 | 0.0011 | 20 | 12 | 19 | 28 | 14 | 11 | 22 | 12 | 11 | 10 | 15 | 17 | 20 | 23 | 13 | 12 | 11 | 12 | 17 | 12,16 | 16 | 11 |
| 374 | 1 | 0.0011 | 21 | 12 | 20 | 28 | 14 | 10 | 23 | 13 | 11 | 11 | 15 | 17 | 20 | 25 | 11 | 14 | 12 | 13 | 18 | 13,17 | 15 | 12 |
| 375 | 1 | 0.0011 | 18 | 13 | 21 | 28 | 16 | 10 | 24 | 12 | 12 | 10 | 14 | 17 | 21 | 24 | 13 | 11 | 9 | 15 | 17 | 11,19 | 15 | 11 |
| 376 | 1 | 0.0011 | 19 | 12 | 18 | 29 | 15 | 10 | 25 | 12 | 11 | 10 | 14 | 18 | 19 | 23 | 12 | 14 | 11 | 13 | 15 | 13,13 | 17 | 12 |
| 377 | 1 | 0.0011 | 18 | 12 | 18 | 28 | 15 | 11 | 25 | 12 | 11 | 10 | 14 | 18 | 19 | 23 | 11 | 14 | 11 | 12 | 15 | 13,13 | 17 | 13 |
| 378 | 1 | 0.0011 | 14 | 14 | 19 | 30 | 14 | 10 | 22 | 12 | 12 | 10 | 14 | 19 | 23 | 23 | 11 | 12 | 10 | 13 | 15 | 11,12 | 16 | 12 |
| 379 | 1 | 0.0011 | 17 | 14 | 21 | 30 | 16 | 10 | 24 | 12 | 13 | 10 | 14 | 15 | 21 | 23 | 11 | 11 | 9 | 15 | 17 | 11,17 | 16 | 11 |
| 380 | 1 | 0.0011 | 20 | 12 | 18 | 27 | 16 | 10 | 21 | 13 | 11 | 10 | 14 | 19 | 20 | 23 | 12 | 14 | 10 | 13 | 15 | 12,13 | 18 | 12 |
| 381 | 1 | 0.0011 | 19 | 12 | 20 | 27 | 14 | 10 | 25 | 14 | 12 | 11 | 15 | 20 | 20 | 23 | 12 | 14 | 12 | 12 | 20 | 13,18 | 15 | 12 |
| 382 | 1 | 0.0011 | 19 | 12 | 19 | 27 | 14 | 10 | 22 | 13 | 11 | 10 | 15 | 16 | 19 | 23 | 12 | 12 | 11 | 12 | 18 | 13,16 | 16 | 12 |
| 383 | 1 | 0.0011 | 19 | 13 | 19 | 29 | 15 | 10 | 23 | 12 | 11 | 10 | 15 | 17 | 21 | 22 | 12 | 13 | 10 | 12 | 18 | 11,11 | 16 | 12 |
| 384 | 1 | 0.0011 | 18 | 12 | 18 | 28 | 15 | 10 | 24 | 12 | 11 | 10 | 14 | 19 | 19 | 23 | 12 | 14 | 11 | 13 | 15 | 13,13 | 17 | 12 |
| 385 | 1 | 0.0011 | 21 | 13 | 21 | 30 | 16 | 11 | 24 | 13 | 12 | 10 | 14 | 16 | 21 | 23 | 13 | 11 | 9 | 15 | 18 | 11,18 | 15 | 12 |
| 386 | 1 | 0.0011 | 18 | 12 | 20 | 28 | 14 | 10 | 24 | 13 | 11 | 11 | 14 | 20 | 20 | 23 | 11 | 14 | 11 | 12 | 17 | 13,19 | 15 | 12 |
| 387 | 1 | 0.0011 | 18 | 12 | 18 | 29 | 16 | 10 | 25 | 13 | 11 | 10 | 14 | 18 | 19 | 23 | 9 | 14 | 11 | 13 | 15 | 13,13 | 18 | 12 |
| 388 | 1 | 0.0011 | 20 | 14 | 20 | 31 | 16 | 10 | 28 | 12 | 12 | 10 | 14 | 17 | 22 | 23 | 11 | 11 | 8 | 15 | 16 | 12,19 | 16 | 11 |
| 389 | 1 | 0.0011 | 19 | 13 | 19 | 28 | 15 | 10 | 22 | 13 | 12 | 10 | 14 | 17 | 21 | 24 | 11 | 15 | 12 | 13 | 16 | 12,13 | 14 | 12 |
| 390 | 1 | 0.0011 | 17 | 12 | 19 | 29 | 15 | 10 | 22 | 12 | 11 | 10 | 15 | 17 | 21 | 23 | 12 | 12 | 11 | 12 | 19 | 12,12 | 15 | 12 |
| 391 | 1 | 0.0011 | 17 | 12 | 19 | 29 | 15 | 10 | 22 | 13 | 11 | 10 | 15 | 17 | 19 | 23 | 12 | 12 | 12 | 12 | 19 | 13,18 | 15 | 12 |
| 392 | 1 | 0.0011 | 14 | 14 | 19 | 31 | 14 | 10 | 22 | 12 | 12 | 10 | 14 | 19 | 23 | 23 | 11 | 12 | 10 | 13 | 15 | 11,12 | 18 | 12 |
| 393 | 1 | 0.0011 | 18 | 12 | 19 | 28 | 15 | 10 | 23 | 12 | 11 | 10 | 15 | 16 | 21 | 24 | 11 | 12 | 10 | 12 | 17 | 11,12 | 15 | 12 |
| 394 | 1 | 0.0011 | 19 | 13 | 20 | 28 | 14 | 11 | 23 | 12 | 11 | 11 | 15 | 22 | 20 | 24 | 12 | 14 | 11 | 12 | 19 | 13,18 | 15 | 10 |
| 395 | 1 | 0.0011 | 18 | 13 | 21 | 29 | 14 | 10 | 23 | 12 | 11 | 11 | 15 | 20 | 20 | 24 | 12 | 14 | 12 | 12 | 17 | 12,13 | 14 | 12 |
| 396 | 1 | 0.0011 | 19 | 14 | 19 | 31 | 15 | 10 | 23 | 11 | 12 | 10 | 14 | 18 | 21 | 22 | 11 | 14 | 10 | 13 | 20 | 11,14 | 17 | 12 |
| 397 | 1 | 0.0011 | 19 | 13 | 18 | 29 | 15 | 11 | 23 | 15 | 10 | 10 | 14 | 18 | 22 | 23 | 12 | 13 | 11 | 14 | 17 | 12,20 | 15 | 11 |
| 398 | 1 | 0.0011 | 17 | 12 | 19 | 28 | 16 | 10 | 24 | 12 | 11 | 10 | 14 | 17 | 20 | 24 | 11 | 14 | 11 | 13 | 15 | 13,14 | 15 | 11 |
| 399 | 1 | 0.0011 | 19 | 12 | 19 | 28 | 15 | 10 | 24 | 13 | 11 | 10 | 14 | 19 | 20 | 23 | 13 | 14 | 11 | 12 | 19 | 13,14 | 16 | 12 |
| 400 | 1 | 0.0011 | 17 | 13 | 21 | 29 | 16 | 10 | 25 | 14 | 12 | 10 | 14 | 17 | 22 | 23 | 11 | 11 | 9 | 14 | 16 | 11,20 | 15 | 12 |
| 401 | 1 | 0.0011 | 19 | 12 | 18 | 30 | 15 | 11 | 25 | 12 | 11 | 10 | 14 | 17 | 19 | 24 | 11 | 14 | 11 | 13 | 15 | 13,14 | 17 | 12 |
| 402 | 1 | 0.0011 | 19 | 12 | 20 | 28 | 14 | 10 | 23 | 13 | 12 | 11 | 15 | 21 | 20 | 24 | 12 | 14 | 11 | 12 | 17 | 14,18 | 15 | 12 |
| 403 | 1 | 0.0011 | 20 | 13 | 20 | 29 | 14 | 10 | 23 | 12 | 10 | 10 | 15 | 20 | 21 | 26 | 12 | 13 | 13 | 12 | 16 | 13,21 | 15 | 11 |
| 404 | 1 | 0.0011 | 17 | 12 | 20 | 29 | 16 | 10 | 26 | 14 | 10 | 10 | 14 | 18 | 21 | 25 | 12 | 13 | 10 | 12 | 17 | 14,18 | 14 | 12 |
| 405 | 1 | 0.0011 | 19 | 12 | 18 | 28 | 17 | 10 | 25 | 12 | 11 | 10 | 14 | 18 | 21 | 24 | 11 | 13 | 10 | 12 | 18 | 12,20 | 14 | 12 |
| 406 | 1 | 0.0011 | 17 | 12 | 18 | 28 | 15 | 11 | 25 | 12 | 11 | 10 | 14 | 19 | 19 | 23 | 11 | 14 | 11 | 13 | 15 | 13,13 | 17 | 12 |
| 407 | 1 | 0.0011 | 19 | 12 | 19 | 28 | 15 | 10 | 21 | 14 | 11 | 10 | 15 | 17 | 20 | 23 | 11 | 12 | 11 | 12 | 17 | 12,12 | 15 | 12 |
| 408 | 1 | 0.0011 | 19 | 12 | 20 | 29 | 16 | 10 | 24 | 12 | 11 | 10 | 15 | 16 | 22 | 23 | 10 | 13 | 12 | 12 | 18 | 13,18 | 14 | 11 |
| 409 | 1 | 0.0011 | 14 | 14 | 19 | 31 | 14 | 10 | 22 | 12 | 11 | 10 | 14 | 18 | 23 | 23 | 11 | 14 | 10 | 13 | 15 | 11,12 | 17 | 12 |
| 410 | 1 | 0.0011 | 19 | 13 | 19 | 30 | 15 | 10 | 21 | 12 | 11 | 10 | 14 | 16 | 20 | 23 | 12 | 12 | 11 | 12 | 20 | 12,16 | 17 | 12 |
| 411 | 1 | 0.0011 | 18 | 14 | 18 | 31 | 15 | 10 | 23 | 12 | 12 | 10 | 14 | 18 | 22 | 24 | 11 | 13 | 12 | 14 | 16 | 12,16 | 15 | 12 |
| 412 | 1 | 0.0011 | 17 | 12 | 18 | 28 | 15 | 10 | 24 | 12 | 11 | 10 | 14 | 19 | 19 | 23 | 12 | 14 | 11 | 14 | 15 | 13,13 | 19 | 12 |
| 413 | 1 | 0.0011 | 18 | 12 | 20 | 28 | 15 | 10 | 23 | 12 | 11 | 12 | 15 | 17 | 21 | 24 | 13 | 14 | 11 | 13 | 18 | 15,20 | 15 | 11 |
| 414 | 1 | 0.0011 | 19 | 12 | 19 | 29 | 15 | 10 | 21 | 12 | 11 | 10 | 14 | 16 | 19 | 23 | 12 | 12 | 11 | 12 | 18 | 12,17 | 15 | 12 |
| 415 | 1 | 0.0011 | 17 | 12 | 20 | 29 | 15 | 10 | 26 | 12 | 11 | 11 | 15 | 17 | 21 | 24 | 11 | 13 | 11 | 12 | 16 | 12,18 | 15 | 12 |
| 416 | 1 | 0.0011 | 21 | 12 | 19 | 28 | 15 | 10 | 22 | 12 | 11 | 10 | 15 | 18 | 19 | 23 | 12 | 11 | 12 | 12 | 17 | 12,17 | 16 | 12 |
| 417 | 1 | 0.0011 | 16 | 12 | 20 | 28 | 15 | 9 | 24 | 13 | 11 | 10 | 14 | 19 | 23 | 25 | 11 | 13 | 10 | 12 | 17 | 15,15 | 14 | 12 |
| 418 | 1 | 0.0011 | 21 | 12 | 19 | 28 | 13 | 10 | 22 | 13 | 11 | 10 | 15 | 19 | 21 | 25 | 13 | 14 | 9 | 13 | 17 | 12,13 | 15 | 12 |
| 419 | 1 | 0.0011 | 21 | 13 | 19 | 29 | 15 | 11 | 25 | 13 | 11 | 10 | 15 | 17 | 24 | 25 | 11 | 13 | 10 | 12 | 17 | 13,17 | 15 | 12 |
| 420 | 1 | 0.0011 | 18 | 11 | 19 | 27 | 15 | 11 | 23 | 11 | 12 | 10 | 15 | 15 | 24 | 24 | 11 | 13 | 11 | 13 | 18 | 12,17 | 14 | 11 |
| 421 | 1 | 0.0011 | 19 | 14 | 22 | 29 | 16 | 10 | 25 | 12 | 12 | 10 | 14 | 14 | 21 | 23 | 12 | 11 | 9 | 15 | 16 | 11,19 | 15 | 11 |
| 422 | 1 | 0.0011 | 19 | 12 | 18 | 28 | 16 | 10 | 26 | 12 | 12 | 10 | 14 | 15 | 20 | 23 | 11 | 13 | 11 | 13 | 17 | 12,17 | 15 | 12 |
| 423 | 1 | 0.0011 | 19 | 12 | 20 | 28 | 15 | 10 | 23 | 12 | 11 | 10 | 14 | 20 | 23 | 25 | 11 | 13 | 10 | 12 | 18 | 14,17 | 15 | 13 |
| 424 | 1 | 0.0011 | 19 | 12 | 20 | 28 | 14 | 10 | 23 | 14 | 11 | 11 | 15 | 17 | 20 | 26 | 11 | 15 | 12 | 12 | 17 | 13,19 | 16 | 12 |
| 425 | 1 | 0.0011 | 18 | 12 | 19 | 29 | 13 | 10 | 22 | 12 | 12 | 10 | 15 | 19 | 21 | 25 | 11 | 13 | 9 | 13 | 19 | 12,12 | 15 | 12 |
| 426 | 1 | 0.0011 | 18 | 13 | 19 | 31 | 17 | 11 | 24 | 12 | 11 | 10 | 15 | 20 | 22 | 23 | 11 | 13 | 10 | 13 | 20 | 12,19 | 15 | 12 |
| 427 | 1 | 0.0011 | 17 | 13 | 21 | 29 | 15 | 10 | 24 | 14 | 11 | 10 | 14 | 16 | 21 | 23 | 11 | 11 | 9 | 14 | 16 | 11,18 | 16 | 11 |
| 428 | 1 | 0.0011 | 16 | 13 | 19 | 30 | 15 | 10 | 23 | 12 | 11 | 11 | 14 | 17 | 21 | 25 | 11 | 13 | 10 | 12 | 17 | 13,19 | 16 | 12 |
| 429 | 1 | 0.0011 | 18 | 13 | 18 | 29 | 15 | 10 | 24 | 12 | 10 | 10 | 14 | 18 | 20 | 24 | 11 | 13 | 12 | 14 | 17 | 13,18 | 16 | 10 |
| 430 | 1 | 0.0011 | 18 | 14 | 21 | 29 | 16 | 10 | 24 | 13 | 12 | 10 | 14 | 16 | 21 | 23 | 12 | 11 | 9 | 14 | 17 | 11,21 | 14 | 11 |
| 431 | 1 | 0.0011 | 18 | 12 | 19 | 28 | 16 | 11 | 25 | 12 | 11 | 10 | 14 | 18 | 19 | 23 | 11 | 14 | 11 | 13 | 15 | 12,13 | 17 | 13 |
| 432 | 1 | 0.0011 | 18 | 12 | 18 | 29 | 15 | 11 | 25 | 13 | 11 | 10 | 14 | 18 | 19 | 23 | 11 | 14 | 11 | 13 | 15 | 13,14 | 18 | 13 |
| 433 | 1 | 0.0011 | 18 | 12 | 18 | 30 | 15 | 11 | 25 | 12 | 11 | 10 | 14 | 18 | 19 | 23 | 12 | 14 | 11 | 13 | 15 | 13,13 | 17 | 12 |
| 434 | 1 | 0.0011 | 16 | 12 | 20 | 28 | 16 | 10 | 24 | 12 | 11 | 10 | 14 | 18 | 22 | 25 | 12 | 13 | 10 | 12 | 20 | 14,18 | 14 | 12 |
| 435 | 1 | 0.0011 | 22 | 12 | 20 | 28 | 15 | 10 | 24 | 13 | 11 | 10 | 15 | 17 | 21 | 23 | 14 | 12 | 10 | 13 | 20 | 12,16 | 16 | 11 |
| 436 | 1 | 0.0011 | 18 | 12 | 20 | 31 | 14 | 10 | 24 | 12 | 11 | 11 | 15 | 18 | 21 | 24 | 11 | 14 | 11 | 12 | 17 | 15,18 | 16 | 13 |
| 437 | 1 | 0.0011 | 18 | 14 | 18 | 31 | 16 | 10 | 23 | 12 | 11 | 10 | 14 | 22 | 21 | 24 | 11 | 13 | 11 | 14 | 15 | 13,16 | 15 | 13 |
| 438 | 1 | 0.0011 | 18 | 12 | 20 | 29 | 15 | 10 | 24 | 13 | 11 | 10 | 15 | 18 | 21 | 24 | 11 | 13 | 11 | 12 | 16 | 13,21 | 14 | 14 |
| 439 | 1 | 0.0011 | 14 | 14 | 18 | 32 | 15 | 10 | 23 | 12 | 12 | 10 | 14 | 17 | 22 | 23 | 11 | 14 | 10 | 14 | 15 | 11,12 | 17 | 12 |
| 440 | 1 | 0.0011 | 17 | 12 | 20 | 30 | 15 | 10 | 24 | 13 | 11 | 10 | 15 | 19 | 21 | 25 | 11 | 13 | 11 | 12 | 16 | 13,21 | 14 | 13 |
| 441 | 1 | 0.0011 | 17 | 12 | 19 | 28 | 15 | 10 | 23 | 13 | 12 | 10 | 14 | 17 | 23 | 25 | 11 | 13 | 11 | 12 | 19 | 12,18 | 15 | 11 |
| 442 | 1 | 0.0011 | 15 | 14 | 19 | 31 | 14 | 10 | 22 | 12 | 12 | 10 | 14 | 18 | 23 | 23 | 11 | 14 | 10 | 13 | 16 | 11,12 | 18 | 12 |
| 443 | 1 | 0.0011 | 17 | 12 | 18 | 28 | 15 | 11 | 25 | 12 | 11 | 10 | 14 | 17 | 19 | 23 | 11 | 14 | 11 | 13 | 15 | 13,13 | 15 | 11 |
| 444 | 1 | 0.0011 | 14 | 14 | 19 | 31 | 14 | 10 | 22 | 12 | 12 | 10 | 14 | 18 | 23 | 23 | 11 | 14 | 10 | 13 | 16 | 11,12 | 17 | 11 |
| 445 | 1 | 0.0011 | 19 | 12 | 18 | 27 | 16 | 11 | 21 | 12 | 12 | 10 | 14 | 18 | 20 | 23 | 12 | 14 | 11 | 13 | 15 | 12,13 | 18 | 12 |
| 446 | 1 | 0.0011 | 18 | 13 | 21 | 29 | 15 | 10 | 26 | 12 | 11 | 10 | 14 | 16 | 21 | 23 | 11 | 11 | 9 | 15 | 17 | 10,19 | 15 | 12 |
| 447 | 1 | 0.0011 | 17 | 14 | 18 | 31 | 14 | 11 | 22 | 12 | 12 | 10 | 14 | 18 | 20 | 23 | 10 | 14 | 11 | 13 | 18 | 12,12 | 14 | 11 |
| 448 | 1 | 0.0011 | 22 | 12 | 19 | 29 | 15 | 11 | 23 | 12 | 11 | 10 | 15 | 16 | 19 | 24 | 13 | 12 | 11 | 12 | 21 | 12,16 | 15 | 12 |
| 449 | 1 | 0.0011 | 19 | 12 | 20 | 28 | 15 | 10 | 22 | 13 | 11 | 11 | 14 | 19 | 22 | 23 | 11 | 12 | 11 | 12 | 18 | 12,12 | 15 | 12 |
| 450 | 1 | 0.0011 | 18 | 12 | 19 | 28 | 13 | 10 | 22 | 12 | 11 | 10 | 15 | 16 | 21 | 25 | 11 | 12 | 10 | 12 | 19 | 12,16 | 14 | 12 |
| 451 | 1 | 0.0011 | 20 | 13 | 21 | 30 | 16 | 10 | 24 | 12 | 13 | 10 | 14 | 16 | 22 | 23 | 11 | 11 | 9 | 14 | 12 | 11,16 | 15 | 11 |
| 452 | 1 | 0.0011 | 19 | 12 | 19 | 27 | 15 | 10 | 22 | 12 | 10 | 10 | 15 | 16 | 20 | 24 | 11 | 12 | 10 | 12 | 16 | 12,18 | 15 | 11 |
| 453 | 1 | 0.0011 | 20 | 13 | 19 | 28 | 17 | 10 | 24 | 12 | 11 | 10 | 14 | 18 | 24 | 25 | 12 | 13 | 10 | 12 | 18 | 13,19 | 14 | 12 |
| 454 | 1 | 0.0011 | 17 | 12 | 19 | 29 | 16 | 10 | 24 | 13 | 11 | 10 | 14 | 18 | 22 | 25 | 10 | 13 | 11 | 12 | 20 | 12,19 | 14 | 11 |
| 455 | 1 | 0.0011 | 18 | 13 | 21 | 30 | 16 | 10 | 24 | 12 | 11 | 10 | 15 | 17 | 22 | 24 | 12 | 13 | 11 | 12 | 19 | 12,22 | 13 | 12 |
| 456 | 1 | 0.0011 | 17 | 14 | 19 | 32 | 15 | 11 | 23 | 13 | 12 | 10 | 14 | 16 | 24 | 23 | 13 | 13 | 12 | 12 | 16 | 12,19 | 14 | 11 |
| 457 | 1 | 0.0011 | 20 | 12 | 19 | 29 | 15 | 10 | 21 | 12 | 11 | 10 | 14 | 16 | 19 | 23 | 13 | 12 | 11 | 12 | 18 | 12,17 | 16 | 12 |
| 458 | 1 | 0.0011 | 18 | 12 | 18 | 29 | 16 | 11 | 24 | 12 | 11 | 10 | 14 | 17 | 19 | 23 | 11 | 14 | 11 | 13 | 14 | 13,13 | 17 | 12 |
| 459 | 1 | 0.0011 | 18 | 12 | 21 | 28 | 15 | 10 | 26 | 11 | 12 | 10 | 14 | 16 | 21 | 23 | 10 | 11 | 8 | 14 | 16 | 12,18 | 15 | 11 |
| 460 | 1 | 0.0011 | 17 | 12 | 18 | 30 | 16 | 11 | 26 | 12 | 12 | 9 | 14 | 18 | 19 | 23 | 11 | 14 | 11 | 13 | 15 | 13,13 | 17 | 11 |
| 461 | 1 | 0.0011 | 17 | 12 | 18 | 29 | 15 | 10 | 25 | 13 | 11 | 10 | 14 | 18 | 19 | 23 | 9 | 14 | 11 | 13 | 16 | 13,15 | 17 | 11 |
| 462 | 1 | 0.0011 | 21 | 12 | 19 | 30 | 15 | 10 | 24 | 12 | 11 | 10 | 15 | 16 | 20 | 23 | 12 | 12 | 12 | 12 | 18 | 12,17 | 15 | 12 |
| 463 | 1 | 0.0011 | 18 | 12 | 18 | 28 | 15 | 10 | 25 | 12 | 11 | 10 | 14 | 19 | 19 | 23 | 11 | 14 | 11 | 13 | 15 | 13,13 | 17 | 12 |
| 464 | 1 | 0.0011 | 19 | 12 | 19 | 28 | 15 | 10 | 23 | 12 | 11 | 10 | 14 | 19 | 21 | 25 | 12 | 13 | 10 | 12 | 19 | 12,18 | 14 | 11 |
| 465 | 1 | 0.0011 | 18 | 12 | 20 | 27 | 13 | 10 | 23 | 12 | 11 | 11 | 14 | 18 | 20 | 23 | 13 | 14 | 11 | 12 | 18 | 13,18 | 15 | 12 |
| 466 | 1 | 0.0011 | 18 | 13 | 18 | 29 | 15 | 10 | 21 | 12 | 12 | 10 | 15 | 18 | 20 | 23 | 11 | 14 | 11 | 12 | 19 | 12,13 | 14 | 10 |
| 467 | 1 | 0.0011 | 19 | 13 | 18 | 31 | 15 | 11 | 25 | 12 | 11 | 10 | 14 | 18 | 19 | 24 | 11 | 14 | 11 | 13 | 15 | 13,13 | 16 | 12 |
| 468 | 1 | 0.0011 | 19 | 14 | 18 | 30 | 15 | 10 | 23 | 12 | 10 | 10 | 14 | 16 | 21 | 26 | 11 | 13 | 12 | 14 | 16 | 14,20 | 16 | 11 |
| 469 | 1 | 0.0011 | 18 | 12 | 18 | 30 | 15 | 10 | 24 | 13 | 12 | 10 | 14 | 17 | 21 | 24 | 12 | 13 | 10 | 12 | 18 | 13,14 | 13 | 12 |
| 470 | 1 | 0.0011 | 19 | 12 | 19 | 29 | 15 | 10 | 28 | 16 | 11 | 10 | 14 | 17 | 23 | 25 | 12 | 13 | 11 | 13 | 19 | 12,18 | 16 | 12 |
| 471 | 1 | 0.0011 | 21 | 12 | 19 | 27 | 16 | 11 | 24 | 13 | 11 | 10 | 15 | 17 | 20 | 23 | 13 | 12 | 11 | 13 | 20 | 12,16 | 15 | 11 |
| 472 | 1 | 0.0011 | 18 | 12 | 20 | 28 | 15 | 10 | 23 | 12 | 11 | 11 | 15 | 18 | 20 | 24 | 12 | 14 | 11 | 13 | 17 | 14,21 | 15 | 12 |
| 473 | 1 | 0.0011 | 18 | 12 | 19 | 28 | 16 | 10 | 24 | 12 | 12 | 10 | 14 | 17 | 21 | 24 | 11 | 15 | 10 | 12 | 19 | 11,19 | 15 | 12 |
| 474 | 1 | 0.0011 | 16 | 12 | 21 | 29 | 14 | 10 | 22 | 12 | 14 | 10 | 14 | 17 | 23 | 25 | 10 | 14 | 11 | 13 | 16 | 10,12 | 15 | 12 |
| 475 | 1 | 0.0011 | 19 | 12 | 19 | 28 | 16 | 10 | 26 | 13 | 12 | 10 | 14 | 16 | 20 | 25 | 14 | 13 | 9 | 12 | 18 | 12,18 | 14 | 12 |
| 476 | 1 | 0.0011 | 19 | 12 | 20 | 28 | 14 | 10 | 23 | 13 | 11 | 11 | 15 | 17 | 20 | 23 | 12 | 14 | 11 | 13 | 17 | 15,19 | 15 | 12 |
| 477 | 1 | 0.0011 | 18 | 12 | 18 | 31 | 15 | 10 | 25 | 13 | 11 | 10 | 14 | 18 | 19 | 23 | 11 | 14 | 11 | 13 | 16 | 13,14 | 16 | 12 |
| 478 | 1 | 0.0011 | 20 | 12 | 19 | 27 | 15 | 10 | 25 | 13 | 11 | 10 | 14 | 17 | 20 | 23 | 11 | 12 | 11 | 13 | 20 | 12,16 | 15 | 11 |
| 479 | 1 | 0.0011 | 14 | 14 | 19 | 31 | 14 | 10 | 22 | 12 | 12 | 10 | 14 | 18 | 23 | 23 | 11 | 14 | 10 | 13 | 15 | 11,12 | 19 | 12 |
| 480 | 1 | 0.0011 | 18 | 14 | 19 | 31 | 13 | 9 | 26 | 12 | 11 | 12 | 14 | 18 | 22 | 24 | 10 | 14 | 10 | 14 | 16 | 15,22 | 15 | 10 |
| 481 | 1 | 0.0011 | 18 | 12 | 18 | 29 | 15 | 10 | 24 | 12 | 11 | 10 | 14 | 19 | 19 | 23 | 11 | 14 | 11 | 13 | 15 | 13,13 | 16 | 11 |
| 482 | 1 | 0.0011 | 20 | 14 | 19 | 30 | 13 | 9 | 24 | 12 | 12 | 12 | 14 | 19 | 22 | 24 | 12 | 14 | 10 | 15 | 17 | 15,22 | 16 | 10 |
| 483 | 1 | 0.0011 | 19 | 12 | 18 | 29 | 15 | 11 | 26 | 12 | 11 | 10 | 14 | 17 | 19 | 23 | 11 | 14 | 11 | 13 | 15 | 13,14 | 17 | 11 |
| 484 | 1 | 0.0011 | 18 | 14 | 18 | 31 | 15 | 10 | 23 | 13 | 11 | 10 | 14 | 18 | 21 | 24 | 11 | 13 | 11 | 14 | 15 | 12,17 | 15 | 12 |
| 485 | 1 | 0.0011 | 17 | 12 | 18 | 29 | 15 | 11 | 25 | 12 | 11 | 10 | 14 | 16 | 19 | 23 | 11 | 14 | 11 | 13 | 15 | 13,13 | 17 | 11 |
| 486 | 1 | 0.0011 | 18 | 11 | 20 | 26 | 15 | 10 | 22 | 13 | 11 | 11 | 15 | 19 | 20 | 24 | 11 | 14 | 11 | 12 | 18 | 13,18 | 14 | 12 |
| 487 | 1 | 0.0011 | 17 | 13 | 20 | 31 | 15 | 10 | 25 | 12 | 12 | 11 | 14 | 16 | 21 | 23 | 11 | 11 | 9 | 14 | 16 | 11,18 | 15 | 12 |
| 488 | 1 | 0.0011 | 17 | 13 | 19 | 29 | 14 | 11 | 24 | 12 | 13 | 10 | 14 | 18 | 21 | 25 | 11 | 15 | 11 | 13 | 16 | 12,12 | 15 | 13 |
| 489 | 1 | 0.0011 | 16 | 12 | 19 | 28 | 15 | 10 | 27 | 14 | 12 | 9 | 15 | 14 | 21 | 24 | 10 | 13 | 12 | 13 | 15 | 12,18 | 15 | 13 |
| 490 | 1 | 0.0011 | 20 | 13 | 21 | 29 | 18 | 10 | 24 | 13 | 12 | 10 | 14 | 16 | 21 | 22 | 11 | 11 | 9 | 14 | 15 | 12,17 | 15 | 11 |
| 491 | 1 | 0.0011 | 20 | 14 | 18 | 30 | 13 | 6 | 24 | 14 | 13 | 12 | 14 | 20 | 22 | 24 | 11 | 14 | 10 | 14 | 17 | 16,19 | 17 | 10 |
| 492 | 1 | 0.0011 | 19 | 12 | 18 | 30 | 14 | 11 | 23 | 12 | 11 | 10 | 14 | 18 | 19 | 23 | 12 | 14 | 11 | 13 | 15 | 13,14 | 17 | 12 |
| 493 | 1 | 0.0011 | 20 | 12 | 20 | 28 | 14 | 10 | 23 | 12 | 11 | 11 | 15 | 18 | 20 | 23 | 13 | 14 | 11 | 12 | 21 | 15,18 | 15 | 11 |
| 494 | 1 | 0.0011 | 19 | 13 | 19 | 30 | 14 | 10 | 22 | 13 | 13 | 11 | 14 | 18 | 22 | 23 | 11 | 14 | 9 | 13 | 16 | 11,12 | 15 | 12 |
| 495 | 1 | 0.0011 | 19 | 13 | 19 | 28 | 17 | 10 | 24 | 12 | 11 | 10 | 14 | 19 | 23 | 25 | 13 | 13 | 10 | 12 | 19 | 12,19 | 14 | 13 |
| 496 | 1 | 0.0011 | 19 | 12 | 19 | 27 | 15 | 11 | 25 | 13 | 11 | 10 | 14 | 18 | 21 | 25 | 11 | 13 | 10 | 12 | 18 | 12,19 | 14 | 11 |
| 497 | 1 | 0.0011 | 17 | 12 | 19 | 28 | 16 | 10 | 25 | 14 | 11 | 10 | 13 | 20 | 21 | 25 | 13 | 15 | 11 | 12 | 19 | 12,21 | 15 | 12 |
| 498 | 1 | 0.0011 | 19 | 12 | 18 | 30 | 15 | 11 | 25 | 12 | 11 | 10 | 14 | 20 | 19 | 23 | 11 | 14 | 11 | 13 | 15 | 13,13 | 16 | 12 |
| 499 | 1 | 0.0011 | 17 | 12 | 18 | 28 | 15 | 10 | 24 | 12 | 11 | 10 | 14 | 18 | 21 | 24 | 13 | 13 | 11 | 14 | 15 | 12,16 | 15 | 13 |
| 500 | 1 | 0.0011 | 19 | 12 | 18 | 28 | 15 | 11 | 25 | 11 | 11 | 10 | 14 | 18 | 19 | 23 | 11 | 14 | 13 | 13 | 15 | 13,13 | 17 | 12 |
| 501 | 1 | 0.0011 | 19 | 12 | 19 | 28 | 14 | 10 | 22 | 14 | 11 | 10 | 15 | 18 | 19 | 24 | 12 | 12 | 11 | 12 | 18 | 12,16 | 15 | 12 |
| 502 | 1 | 0.0011 | 19 | 12 | 19 | 30 | 15 | 11 | 23 | 13 | 11 | 10 | 14 | 17 | 21 | 22 | 10 | 13 | 11 | 12 | 18 | 12,20 | 15 | 11 |
| 503 | 1 | 0.0011 | 19 | 14 | 22 | 30 | 17 | 10 | 24 | 13 | 12 | 10 | 14 | 18 | 22 | 23 | 13 | 11 | 9 | 14 | 18 | 11,17 | 15 | 11 |
| 504 | 1 | 0.0011 | 19 | 12 | 18 | 27 | 16 | 10 | 21 | 13 | 11 | 10 | 14 | 18 | 20 | 23 | 12 | 14 | 11 | 13 | 15 | 12,14 | 17 | 11 |
| 505 | 1 | 0.0011 | 16 | 12 | 18 | 28 | 15 | 11 | 25 | 12 | 11 | 10 | 14 | 19 | 19 | 23 | 11 | 14 | 11 | 13 | 15 | 13,13 | 17 | 11 |
| 506 | 1 | 0.0011 | 18 | 13 | 19 | 28 | 16 | 10 | 24 | 12 | 11 | 10 | 14 | 18 | 23 | 25 | 12 | 13 | 10 | 12 | 18 | 13,19 | 14 | 13 |
| 507 | 1 | 0.0011 | 19 | 12 | 19 | 28 | 15 | 10 | 24 | 13 | 11 | 10 | 14 | 18 | 20 | 23 | 12 | 14 | 11 | 12 | 19 | 13,14 | 16 | 12 |
| 508 | 1 | 0.0011 | 19 | 14 | 19 | 29 | 13 | 10 | 24 | 12 | 12 | 12 | 14 | 22 | 22 | 24 | 11 | 14 | 10 | 14 | 17 | 16,22 | 15 | 10 |
| 509 | 1 | 0.0011 | 19 | 12 | 18 | 27 | 17 | 10 | 21 | 13 | 11 | 10 | 14 | 18 | 20 | 23 | 12 | 14 | 11 | 13 | 15 | 12,13 | 17 | 12 |
| 510 | 1 | 0.0011 | 19 | 13 | 19 | 29 | 17 | 10 | 24 | 13 | 11 | 10 | 14 | 19 | 21 | 25 | 12 | 13 | 11 | 12 | 18 | 12,19 | 14 | 11 |
| 511 | 1 | 0.0011 | 18 | 12 | 19 | 28 | 17 | 10 | 24 | 13 | 11 | 10 | 14 | 17 | 21 | 23 | 11 | 13 | 10 | 12 | 17 | 12,18 | 14 | 12 |
| 512 | 1 | 0.0011 | 21 | 12 | 18 | 28 | 16 | 10 | 25 | 12 | 12 | 10 | 15 | 16 | 21 | 23 | 11 | 14 | 13 | 12 | 18 | 12,16 | 16 | 12 |
| 513 | 1 | 0.0011 | 18 | 11 | 19 | 29 | 15 | 11 | 22 | 12 | 12 | 10 | 14 | 17 | 22 | 23 | 10 | 13 | 11 | 12 | 18 | 12,19 | 15 | 11 |
| 514 | 1 | 0.0011 | 18 | 12 | 18 | 28 | 14 | 11 | 25 | 12 | 11 | 10 | 14 | 18 | 19 | 23 | 11 | 14 | 11 | 12 | 16 | 13,13 | 16 | 12 |
| 515 | 1 | 0.0011 | 17 | 12 | 20 | 28 | 17 | 10 | 26 | 11 | 11 | 10 | 15 | 16 | 21 | 24 | 13 | 14 | 12 | 12 | 18 | 13,21 | 15 | 11 |
| 516 | 1 | 0.0011 | 17 | 13 | 20 | 29 | 17 | 10 | 25 | 12 | 11 | 10 | 14 | 21 | 23 | 24 | 11 | 14 | 10 | 12 | 18 | 15,18 | 14 | 12 |
| 517 | 1 | 0.0011 | 20 | 13 | 19 | 29 | 15 | 10 | 22 | 12 | 11 | 10 | 15 | 16 | 19 | 23 | 12 | 12 | 10 | 12 | 19 | 12,13 | 15 | 13 |
| 518 | 1 | 0.0011 | 18 | 13 | 19 | 28 | 17 | 10 | 24 | 12 | 11 | 10 | 14 | 17 | 26 | 25 | 12 | 13 | 10 | 12 | 20 | 13,19 | 14 | 12 |
| 519 | 1 | 0.0011 | 17 | 12 | 18 | 30 | 16 | 11 | 26 | 12 | 12 | 9 | 14 | 18 | 19 | 23 | 11 | 14 | 11 | 13 | 15 | 13,13 | 17 | 12 |
| 520 | 1 | 0.0011 | 17 | 13 | 19 | 31 | 16 | 11 | 24 | 12 | 13 | 11 | 14 | 20 | 23 | 26 | 10 | 11 | 10 | 13 | 15 | 11,14 | 15 | 13 |
| 521 | 1 | 0.0011 | 18 | 13 | 20 | 29 | 14 | 10 | 23 | 12 | 12 | 11 | 15 | 18 | 20 | 24 | 12 | 14 | 12 | 12 | 18 | 13,19 | 14 | 12 |
| 522 | 1 | 0.0011 | 14 | 14 | 19 | 31 | 14 | 10 | 22 | 12 | 12 | 10 | 14 | 18 | 23 | 23 | 11 | 14 | 10 | 13 | 15 | 11,12 | 16 | 12 |
| 523 | 1 | 0.0011 | 16 | 12 | 20 | 29 | 17 | 10 | 24 | 13 | 11 | 10 | 14 | 20 | 22 | 25 | 11 | 13 | 11 | 12 | 18 | 14,18 | 14 | 12 |
| 524 | 1 | 0.0011 | 19 | 13 | 19 | 28 | 15 | 10 | 22 | 14 | 11 | 10 | 15 | 18 | 20 | 22 | 11 | 14 | 10 | 12 | 17 | 11,11 | 16 | 12 |
| 525 | 1 | 0.0011 | 19 | 12 | 18 | 27 | 16 | 11 | 21 | 13 | 11 | 10 | 14 | 19 | 21 | 23 | 12 | 14 | 11 | 13 | 15 | 12,13 | 18 | 12 |
| 526 | 1 | 0.0011 | 18 | 12 | 19 | 28 | 15 | 11 | 23 | 13 | 11 | 10 | 15 | 18 | 20 | 23 | 13 | 12 | 11 | 12 | 17 | 12,16 | 16 | 11 |
| 527 | 1 | 0.0011 | 20 | 12 | 18 | 28 | 15 | 10 | 23 | 12 | 11 | 11 | 14 | 20 | 20 | 23 | 12 | 14 | 11 | 13 | 16 | 13,13 | 15 | 11 |
| 528 | 1 | 0.0011 | 17 | 12 | 18 | 30 | 15 | 10 | 25 | 14 | 11 | 10 | 14 | 17 | 19 | 23 | 11 | 14 | 11 | 13 | 15 | 13,13 | 16 | 11 |
| 529 | 1 | 0.0011 | 18 | 12 | 18 | 28 | 15 | 10 | 24 | 13 | 11 | 10 | 14 | 18 | 18 | 23 | 11 | 14 | 11 | 13 | 15 | 13,14 | 16 | 12 |
| 530 | 1 | 0.0011 | 14 | 14 | 19 | 30 | 14 | 10 | 22 | 12 | 12 | 10 | 14 | 18 | 22 | 23 | 11 | 12 | 10 | 13 | 15 | 11,12 | 17 | 12 |
| 531 | 1 | 0.0011 | 20 | 13 | 18 | 30 | 15 | 10 | 22 | 12 | 10 | 11 | 14 | 17 | 22 | 25 | 12 | 13 | 11 | 14 | 17 | 14,18 | 15 | 11 |
| 532 | 1 | 0.0011 | 17 | 13 | 19 | 28 | 15 | 11 | 22 | 14 | 11 | 10 | 14 | 17 | 21 | 24 | 12 | 12 | 12 | 12 | 17 | 14,18 | 16 | 11 |
| 533 | 1 | 0.0011 | 19 | 13 | 18 | 29 | 14 | 11 | 21 | 12 | 13 | 10 | 14 | 18 | 20 | 23 | 10 | 14 | 11 | 13 | 17 | 11,12 | 13 | 11 |
| 534 | 1 | 0.0011 | 19 | 13 | 19 | 30 | 15 | 10 | 22 | 12 | 11 | 10 | 14 | 15 | 21 | 23 | 11 | 13 | 9 | 12 | 16 | 12,19 | 15 | 11 |
| 535 | 1 | 0.0011 | 17 | 13 | 20 | 29 | 15 | 10 | 26 | 12 | 12 | 10 | 14 | 16 | 20 | 23 | 11 | 11 | 9 | 14 | 16 | 11,21 | 16 | 12 |
| 536 | 1 | 0.0011 | 18 | 12 | 18 | 29 | 16 | 11 | 28 | 12 | 12 | 10 | 14 | 17 | 19 | 23 | 12 | 14 | 12 | 13 | 15 | 13,13 | 16 | 12 |
| 537 | 1 | 0.0011 | 16 | 13 | 17 | 30 | 14 | 11 | 27 | 13 | 11 | 10 | 14 | 21 | 21 | 24 | 10 | 11 | 10 | 13 | 17 | 14,19 | 16 | 11 |
| 538 | 1 | 0.0011 | 18 | 13 | 20 | 29 | 14 | 10 | 22 | 12 | 12 | 10 | 14 | 18 | 21 | 23 | 11 | 15 | 11 | 13 | 17 | 12,13 | 15 | 11 |
| 539 | 1 | 0.0011 | 16 | 12 | 17 | 29 | 16 | 10 | 24 | 12 | 10 | 10 | 14 | 19 | 21 | 23 | 12 | 12 | 11 | 14 | 14 | 12,14 | 16 | 12 |
| 540 | 1 | 0.0011 | 18 | 13 | 18 | 29 | 16 | 10 | 23 | 13 | 10 | 10 | 14 | 19 | 23 | 23 | 12 | 13 | 12 | 14 | 17 | 12,21 | 15 | 11 |
| 541 | 1 | 0.0011 | 14 | 14 | 19 | 29 | 14 | 10 | 22 | 12 | 12 | 10 | 14 | 17 | 22 | 23 | 11 | 14 | 10 | 13 | 15 | 11,12 | 16 | 12 |
| 542 | 1 | 0.0011 | 19 | 13 | 19 | 29 | 17 | 10 | 24 | 12 | 11 | 10 | 14 | 20 | 21 | 25 | 12 | 13 | 11 | 12 | 19 | 12,18 | 14 | 11 |
| 543 | 1 | 0.0011 | 17 | 12 | 20 | 28 | 14 | 10 | 23 | 12 | 11 | 11 | 15 | 20 | 20 | 23 | 11 | 14 | 12 | 12 | 22 | 13,19 | 14 | 12 |
| 544 | 1 | 0.0011 | 20 | 12 | 18 | 30 | 15 | 11 | 25 | 13 | 11 | 10 | 14 | 20 | 21 | 22 | 13 | 14 | 11 | 13 | 15 | 13,14 | 16 | 12 |
| 545 | 1 | 0.0011 | 17 | 12 | 20 | 30 | 15 | 10 | 24 | 12 | 11 | 10 | 15 | 18 | 21 | 25 | 11 | 13 | 11 | 12 | 16 | 13,21 | 14 | 13 |
| 546 | 1 | 0.0011 | 19 | 14 | 19 | 31 | 13 | 9 | 24 | 13 | 12 | 12 | 14 | 18 | 22 | 24 | 11 | 14 | 10 | 13 | 16 | 16,21 | 16 | 10 |
| 547 | 1 | 0.0011 | 17 | 13 | 20 | 30 | 16 | 11 | 28 | 13 | 11 | 10 | 15 | 15 | 22 | 25 | 11 | 13 | 10 | 12 | 18 | 12,20 | 16 | 11 |
| 548 | 1 | 0.0011 | 18 | 12 | 18 | 29 | 16 | 11 | 25 | 12 | 11 | 10 | 14 | 16 | 19 | 23 | 12 | 15 | 11 | 13 | 15 | 13,13 | 17 | 11 |
| 549 | 1 | 0.0011 | 17 | 14 | 19 | 30 | 14 | 10 | 23 | 13 | 12 | 10 | 14 | 20 | 21 | 22 | 10 | 14 | 11 | 13 | 18 | 11,12 | 15 | 13 |
| 550 | 1 | 0.0011 | 19 | 12 | 20 | 29 | 14 | 11 | 23 | 13 | 10 | 11 | 15 | 19 | 21 | 25 | 12 | 13 | 12 | 12 | 17 | 13,17 | 16 | 11 |
| 551 | 1 | 0.0011 | 19 | 12 | 19 | 27 | 15 | 10 | 22 | 14 | 11 | 11 | 15 | 18 | 20 | 25 | 11 | 14 | 11 | 12 | 18 | 13,17 | 15 | 12 |
| 552 | 1 | 0.0011 | 18 | 12 | 18 | 27 | 16 | 11 | 21 | 13 | 11 | 10 | 14 | 18 | 20 | 23 | 12 | 14 | 10 | 13 | 15 | 12,13 | 16 | 12 |
| 553 | 1 | 0.0011 | 18 | 12 | 18 | 28 | 15 | 11 | 25 | 12 | 11 | 10 | 14 | 19 | 19 | 23 | 11 | 14 | 11 | 13 | 14 | 13,14 | 17 | 12 |
| 554 | 1 | 0.0011 | 19 | 14 | 18 | 30 | 14 | 9 | 24 | 12 | 12 | 12 | 14 | 20 | 22 | 24 | 11 | 14 | 10 | 14 | 17 | 15,21 | 17 | 10 |
| 555 | 1 | 0.0011 | 18 | 12 | 19 | 28 | 15 | 10 | 23 | 12 | 11 | 11 | 14 | 19 | 20 | 23 | 12 | 14 | 11 | 12 | 18 | 13,15 | 16 | 12 |
| 556 | 1 | 0.0011 | 18 | 13 | 20 | 29 | 14 | 10 | 23 | 12 | 11 | 11 | 15 | 18 | 20 | 24 | 11 | 14 | 11 | 12 | 19 | 13,19 | 15 | 12 |
| 557 | 1 | 0.0011 | 18 | 13 | 21 | 29 | 16 | 10 | 24 | 13 | 12 | 10 | 14 | 15 | 23 | 23 | 12 | 11 | 9 | 14 | 15 | 11,17 | 15 | 11 |
| 558 | 1 | 0.0011 | 18 | 12 | 19 | 28 | 13 | 11 | 23 | 12 | 11 | 10 | 15 | 17 | 19 | 24 | 13 | 12 | 12 | 12 | 18 | 12,16 | 15 | 12 |
| 559 | 1 | 0.0011 | 20 | 13 | 19 | 29 | 15 | 10 | 25 | 12 | 12 | 10 | 15 | 14 | 21 | 24 | 12 | 13 | 11 | 12 | 19 | 12,17 | 16 | 11 |
| 560 | 1 | 0.0011 | 16 | 12 | 20 | 27 | 16 | 10 | 22 | 12 | 11 | 11 | 14 | 19 | 21 | 25 | 11 | 13 | 10 | 12 | 18 | 14,17 | 15 | 12 |
| 561 | 1 | 0.0011 | 18 | 12 | 20 | 28 | 14 | 10 | 24 | 12 | 11 | 11 | 15 | 19 | 20 | 24 | 11 | 14 | 11 | 12 | 19 | 14,20 | 15 | 12 |
| 562 | 1 | 0.0011 | 19 | 12 | 19 | 31 | 15 | 10 | 22 | 12 | 11 | 8 | 15 | 16 | 19 | 23 | 12 | 12 | 10 | 13 | 18 | 12,13 | 15 | 12 |
| 563 | 1 | 0.0011 | 18 | 12 | 18 | 27 | 15 | 10 | 27 | 12 | 11 | 10 | 14 | 18 | 20 | 22 | 13 | 14 | 11 | 13 | 16 | 13,13 | 15 | 12 |
| 564 | 1 | 0.0011 | 18 | 12 | 19 | 28 | 14 | 10 | 22 | 12 | 12 | 10 | 14 | 15 | 20 | 23 | 12 | 12 | 11 | 12 | 17 | 12,16 | 17 | 12 |
| 565 | 1 | 0.0011 | 19 | 13 | 19 | 29 | 16 | 10 | 23 | 12 | 11 | 10 | 15 | 16 | 19 | 23 | 11 | 12 | 12 | 13 | 18 | 12,16 | 16 | 12 |
| 566 | 1 | 0.0011 | 16 | 12 | 19 | 28 | 14 | 11 | 24 | 13 | 11 | 11 | 15 | 17 | 21 | 24 | 11 | 14 | 11 | 12 | 19 | 13,19 | 15 | 12 |
| 567 | 1 | 0.0011 | 18 | 13 | 19 | 29 | 17 | 10 | 22 | 12 | 11 | 10 | 15 | 17 | 20 | 23 | 14 | 12 | 11 | 12 | 20 | 12,16 | 16 | 12 |
| 568 | 1 | 0.0011 | 19 | 12 | 19 | 28 | 16 | 12 | 25 | 13 | 11 | 10 | 14 | 19 | 21 | 25 | 12 | 13 | 10 | 12 | 17 | 14,19 | 15 | 11 |
| 569 | 1 | 0.0011 | 17 | 12 | 20 | 29 | 15 | 11 | 22 | 13 | 11 | 10 | 15 | 16 | 20 | 23 | 14 | 12 | 11 | 12 | 16 | 12,16 | 15 | 11 |
| 570 | 1 | 0.0011 | 19 | 12 | 18 | 28 | 15 | 11 | 25 | 12 | 11 | 10 | 14 | 18 | 19 | 23 | 11 | 14 | 11 | 13 | 15 | 13,13 | 17 | 12 |
| 571 | 1 | 0.0011 | 17 | 12 | 18 | 29 | 15 | 10 | 25 | 12 | 11 | 10 | 14 | 18 | 19 | 23 | 11 | 14 | 11 | 13 | 15 | 13,15 | 17 | 12 |
| 572 | 1 | 0.0011 | 16 | 14 | 19 | 30 | 14 | 10 | 21 | 12 | 12 | 10 | 14 | 17 | 22 | 22 | 10 | 14 | 11 | 13 | 17 | 12,12 | 15 | 12 |
| 573 | 1 | 0.0011 | 17 | 13 | 19 | 29 | 15 | 11 | 22 | 13 | 12 | 10 | 14 | 17 | 22 | 23 | 11 | 13 | 11 | 12 | 20 | 12,12 | 15 | 12 |
| 574 | 1 | 0.0011 | 18 | 14 | 18 | 30 | 15 | 10 | 23 | 12 | 11 | 10 | 14 | 18 | 21 | 24 | 11 | 13 | 11 | 14 | 14 | 12,15 | 15 | 12 |
| 575 | 1 | 0.0011 | 20 | 14 | 19 | 30 | 13 | 9 | 24 | 12 | 12 | 12 | 14 | 19 | 22 | 24 | 11 | 14 | 11 | 14 | 19 | 15,22 | 17 | 10 |
| 576 | 1 | 0.0011 | 17 | 12 | 18 | 30 | 15 | 10 | 24 | 12 | 11 | 10 | 14 | 16 | 20 | 23 | 11 | 14 | 11 | 13 | 15 | 13,14 | 15 | 11 |
| 577 | 1 | 0.0011 | 19 | 12 | 19 | 28 | 13 | 11 | 22 | 12 | 11 | 10 | 15 | 17 | 19 | 23 | 12 | 12 | 11 | 12 | 18 | 12,12 | 15 | 12 |
| 578 | 1 | 0.0011 | 15 | 12 | 20 | 28 | 16 | 10 | 25 | 12 | 11 | 10 | 14 | 17 | 21 | 24 | 12 | 13 | 11 | 12 | 18 | 12,18 | 14 | 12 |
| 579 | 1 | 0.0011 | 18 | 12 | 19 | 27 | 16 | 10 | 22 | 13 | 11 | 10 | 14 | 17 | 22 | 25 | 12 | 13 | 10 | 12 | 18 | 11,22 | 14 | 12 |
| 580 | 1 | 0.0011 | 19 | 12 | 18 | 28 | 15 | 10 | 25 | 12 | 11 | 10 | 14 | 21 | 19 | 23 | 11 | 14 | 11 | 14 | 17 | 13,13 | 18 | 12 |
| 581 | 1 | 0.0011 | 18 | 14 | 18 | 31 | 15 | 10 | 23 | 13 | 11 | 10 | 14 | 20 | 21 | 24 | 11 | 14 | 11 | 14 | 15 | 12,16 | 14 | 12 |
| 582 | 1 | 0.0011 | 18 | 14 | 18 | 30 | 15 | 10 | 23 | 12 | 11 | 10 | 14 | 20 | 21 | 24 | 11 | 13 | 11 | 14 | 15 | 12,17 | 15 | 12 |
| 583 | 1 | 0.0011 | 18 | 14 | 21 | 30 | 15 | 10 | 26 | 13 | 13 | 10 | 14 | 16 | 21 | 23 | 11 | 11 | 8 | 15 | 15 | 12,16 | 15 | 11 |
| 584 | 1 | 0.0011 | 15 | 13 | 19 | 30 | 15 | 10 | 22 | 12 | 11 | 10 | 14 | 17 | 21 | 23 | 11 | 12 | 12 | 13 | 18 | 15,19 | 15 | 11 |
| 585 | 1 | 0.0011 | 17 | 12 | 20 | 28 | 16 | 11 | 25 | 11 | 12 | 11 | 14 | 17 | 21 | 25 | 11 | 13 | 12 | 12 | 17 | 13,19 | 15 | 11 |
| 586 | 1 | 0.0011 | 18 | 14 | 18 | 29 | 16 | 10 | 23 | 12 | 11 | 13 | 14 | 19 | 20 | 23 | 12 | 13 | 13 | 13 | 17 | 10,18 | 15 | 11 |
| 587 | 1 | 0.0011 | 20 | 14 | 20 | 30 | 14 | 10 | 23 | 12 | 11 | 11 | 15 | 18 | 22 | 25 | 14 | 14 | 11 | 12 | 16 | 13,19 | 16 | 12 |
| 588 | 1 | 0.0011 | 18 | 12 | 19 | 30 | 17 | 10 | 25 | 12 | 11 | 10 | 14 | 16 | 24 | 24 | 13 | 13 | 10 | 12 | 16 | 12,19 | 14 | 11 |
| 589 | 1 | 0.0011 | 15 | 12 | 19 | 28 | 15 | 10 | 26 | 12 | 11 | 10 | 14 | 17 | 22 | 24 | 12 | 13 | 10 | 12 | 17 | 12,19 | 15 | 12 |
| 590 | 1 | 0.0011 | 18 | 13 | 21 | 29 | 15 | 11 | 24 | 12 | 14 | 10 | 14 | 16 | 21 | 23 | 11 | 11 | 9 | 15 | 16 | 11,17 | 15 | 11 |
| 591 | 1 | 0.0011 | 20 | 12 | 20 | 28 | 15 | 11 | 22 | 13 | 11 | 10 | 15 | 17 | 20 | 24 | 13 | 12 | 11 | 12 | 18 | 12,16 | 16 | 12 |
| 592 | 1 | 0.0011 | 17 | 12 | 18 | 30 | 15 | 10 | 24 | 12 | 11 | 10 | 14 | 17 | 20 | 23 | 11 | 14 | 11 | 13 | 15 | 13,13 | 16 | 11 |
| 593 | 1 | 0.0011 | 17 | 14 | 19 | 30 | 15 | 11 | 24 | 13 | 11 | 10 | 14 | 20 | 21 | 24 | 11 | 13 | 11 | 14 | 15 | 12,18 | 15 | 12 |
| 594 | 1 | 0.0011 | 20 | 14 | 19 | 31 | 15 | 10 | 27 | 13 | 11 | 11 | 15 | 18 | 21 | 22 | 13 | 14 | 12 | 12 | 19 | 14,18 | 15 | 11 |
| 595 | 1 | 0.0011 | 18 | 12 | 20 | 28 | 14 | 10 | 23 | 11 | 11 | 11 | 15 | 17 | 20 | 24 | 12 | 14 | 11 | 12 | 16 | 13,17 | 14 | 12 |
| 596 | 1 | 0.0011 | 18 | 12 | 19 | 28 | 15 | 10 | 22 | 12 | 11 | 10 | 15 | 17 | 19 | 23 | 12 | 12 | 11 | 12 | 17 | 12,16 | 15 | 12 |
| 597 | 1 | 0.0011 | 16 | 12 | 19 | 28 | 15 | 10 | 22 | 13 | 10 | 10 | 15 | 17 | 19 | 23 | 12 | 12 | 11 | 12 | 17 | 12,17 | 15 | 12 |
| 598 | 1 | 0.0011 | 19 | 12 | 19 | 27 | 14 | 10 | 21 | 13 | 11 | 11 | 15 | 17 | 20 | 24 | 14 | 14 | 12 | 12 | 18 | 14,19 | 15 | 12 |
| 599 | 1 | 0.0011 | 17 | 13 | 21 | 28 | 15 | 11 | 24 | 12 | 11 | 10 | 14 | 18 | 21 | 22 | 12 | 11 | 9 | 14 | 17 | 11,19 | 15 | 10 |
| 600 | 1 | 0.0011 | 20 | 12 | 19 | 29 | 15 | 10 | 22 | 12 | 11 | 10 | 15 | 17 | 19 | 23 | 13 | 12 | 11 | 12 | 16 | 12,16 | 16 | 12 |
| 601 | 1 | 0.0011 | 16 | 12 | 17 | 29 | 15 | 10 | 26 | 13 | 11 | 10 | 14 | 18 | 21 | 24 | 12 | 12 | 11 | 14 | 15 | 13,15 | 16 | 12 |
| 602 | 1 | 0.0011 | 18 | 14 | 20 | 30 | 14 | 10 | 24 | 12 | 11 | 11 | 14 | 19 | 20 | 23 | 12 | 14 | 11 | 12 | 19 | 13,20 | 15 | 12 |
| 603 | 1 | 0.0011 | 20 | 13 | 18 | 29 | 15 | 10 | 21 | 13 | 13 | 10 | 14 | 16 | 22 | 22 | 11 | 13 | 12 | 12 | 16 | 12,19 | 15 | 11 |
| 604 | 1 | 0.0011 | 18 | 13 | 20 | 29 | 15 | 11 | 22 | 12 | 12 | 11 | 15 | 17 | 24 | 23 | 12 | 13 | 11 | 12 | 18 | 12,19 | 15 | 11 |
| 605 | 1 | 0.0011 | 18 | 12 | 18 | 29 | 15 | 11 | 25 | 12 | 11 | 10 | 14 | 20 | 19 | 23 | 11 | 14 | 11 | 13 | 15 | 13,14 | 17 | 12 |
| 606 | 1 | 0.0011 | 19 | 13 | 19 | 32 | 16 | 10 | 23 | 15 | 11 | 10 | 15 | 16 | 21 | 24 | 11 | 13 | 10 | 12 | 17 | 13,14 | 13 | 11 |
| 607 | 1 | 0.0011 | 20 | 12 | 19 | 27 | 16 | 10 | 22 | 11 | 11 | 10 | 15 | 16 | 19 | 23 | 12 | 12 | 11 | 12 | 20 | 12,12 | 15 | 12 |
| 608 | 1 | 0.0011 | 17 | 12 | 18 | 30 | 16 | 10 | 24 | 12 | 11 | 10 | 14 | 17 | 20 | 23 | 11 | 14 | 11 | 13 | 15 | 13,13 | 16 | 11 |
| 609 | 1 | 0.0011 | 21 | 13 | 19 | 29 | 16 | 9 | 26 | 13 | 11 | 10 | 15 | 17 | 22 | 24 | 12 | 13 | 10 | 12 | 15 | 12,19 | 14 | 12 |
| 610 | 1 | 0.0011 | 18 | 12 | 20 | 29 | 14 | 10 | 23 | 14 | 12 | 11 | 15 | 20 | 22 | 25 | 11 | 15 | 11 | 13 | 17 | 14,18 | 15 | 12 |
| 611 | 1 | 0.0011 | 19 | 12 | 19 | 28 | 15 | 10 | 23 | 12 | 11 | 10 | 14 | 17 | 21 | 23 | 11 | 12 | 11 | 12 | 20 | 12,18 | 15 | 9 |
| 612 | 1 | 0.0011 | 19 | 12 | 19 | 30 | 14 | 11 | 23 | 13 | 11 | 10 | 15 | 16 | 20 | 24 | 14 | 12 | 11 | 12 | 17 | 12,17 | 16 | 12 |
| 613 | 1 | 0.0011 | 19 | 13 | 23 | 30 | 15 | 10 | 23 | 13 | 10 | 10 | 15 | 19 | 21 | 24 | 13 | 13 | 13 | 12 | 17 | 12,20 | 15 | 11 |
| 614 | 1 | 0.0011 | 17 | 13 | 17 | 29 | 15 | 10 | 24 | 12 | 12 | 10 | 14 | 18 | 20 | 23 | 12 | 13 | 11 | 14 | 16 | 10,15 | 15 | 11 |
| 615 | 1 | 0.0011 | 19 | 14 | 21 | 30 | 15 | 10 | 27 | 13 | 13 | 10 | 14 | 16 | 21 | 23 | 11 | 11 | 8 | 15 | 15 | 12,17 | 15 | 11 |
| 616 | 1 | 0.0011 | 17 | 13 | 21 | 29 | 17 | 11 | 25 | 12 | 12 | 10 | 14 | 16 | 23 | 23 | 12 | 11 | 9 | 15 | 17 | 11,18 | 15 | 12 |
| 617 | 1 | 0.0011 | 18 | 12 | 19 | 30 | 17 | 10 | 25 | 12 | 11 | 10 | 14 | 16 | 24 | 25 | 13 | 13 | 10 | 12 | 16 | 12,19 | 14 | 11 |
| 618 | 1 | 0.0011 | 19 | 13 | 18 | 30 | 16 | 10 | 25 | 12 | 10 | 10 | 14 | 20 | 19 | 23 | 12 | 14 | 12 | 13 | 15 | 13,13 | 17 | 10 |
| 619 | 1 | 0.0011 | 18 | 13 | 18 | 30 | 15 | 11 | 21 | 13 | 12 | 10 | 14 | 16 | 21 | 22 | 11 | 13 | 11 | 12 | 16 | 12,19 | 15 | 11 |
| 620 | 1 | 0.0011 | 19 | 13 | 20 | 29 | 14 | 10 | 23 | 12 | 11 | 11 | 15 | 18 | 20 | 24 | 12 | 14 | 11 | 12 | 16 | 13,19 | 15 | 12 |
| 621 | 1 | 0.0011 | 18 | 12 | 20 | 28 | 14 | 10 | 23 | 13 | 11 | 11 | 14 | 19 | 21 | 23 | 11 | 14 | 11 | 12 | 16 | 13,19 | 15 | 12 |
| 622 | 1 | 0.0011 | 17 | 12 | 18 | 29 | 14 | 10 | 25 | 13 | 11 | 11 | 14 | 18 | 19 | 23 | 11 | 14 | 11 | 13 | 15 | 12,12 | 19 | 12 |
| 623 | 1 | 0.0011 | 18 | 14 | 18 | 32 | 16 | 10 | 22 | 12 | 11 | 10 | 14 | 17 | 21 | 22 | 11 | 13 | 11 | 12 | 16 | 11,20 | 15 | 12 |
| 624 | 1 | 0.0011 | 18 | 12 | 19 | 28 | 14 | 11 | 22 | 14 | 11 | 10 | 15 | 17 | 22 | 23 | 12 | 12 | 11 | 12 | 19 | 12,16 | 16 | 12 |
| 625 | 1 | 0.0011 | 19 | 13 | 20 | 29 | 16 | 11 | 24 | 12 | 12 | 10 | 14 | 16 | 21 | 23 | 14 | 13 | 9 | 14 | 16 | 11,18 | 15 | 11 |
| 626 | 1 | 0.0011 | 19 | 12 | 20 | 28 | 15 | 11 | 24 | 14 | 11 | 10 | 15 | 19 | 24 | 24 | 12 | 13 | 10 | 12 | 18 | 13,18 | 13 | 12 |
| 627 | 1 | 0.0011 | 16 | 13 | 18 | 29 | 15 | 10 | 25 | 13 | 11 | 11 | 14 | 18 | 22 | 23 | 13 | 13 | 8 | 14 | 16 | 12,19 | 16 | 12 |
| 628 | 1 | 0.0011 | 19 | 12 | 20 | 28 | 14 | 10 | 23 | 13 | 11 | 11 | 15 | 17 | 20 | 23 | 11 | 14 | 11 | 12 | 17 | 15,20 | 15 | 12 |
| 629 | 1 | 0.0011 | 17 | 13 | 19 | 30 | 15 | 10 | 24 | 12 | 11 | 10 | 14 | 18 | 19 | 23 | 11 | 13 | 11 | 13 | 15 | 13,13 | 0 | 12 |
| 630 | 1 | 0.0011 | 19 | 13 | 20 | 30 | 14 | 10 | 23 | 12 | 12 | 12 | 14 | 18 | 21 | 25 | 12 | 14 | 11 | 12 | 17 | 13,18 | 15 | 11 |
| 631 | 1 | 0.0011 | 20 | 13 | 18 | 28 | 17 | 10 | 22 | 13 | 11 | 10 | 15 | 17 | 19 | 23 | 12 | 12 | 11 | 12 | 19 | 12,12 | 15 | 12 |
| 632 | 1 | 0.0011 | 20 | 12 | 19 | 28 | 15 | 10 | 22 | 13 | 11 | 11 | 15 | 16 | 19 | 23 | 12 | 12 | 11 | 12 | 20 | 12,17 | 16 | 12 |
| 633 | 1 | 0.0011 | 17 | 12 | 18 | 28 | 15 | 10 | 26 | 12 | 11 | 10 | 14 | 18 | 20 | 23 | 11 | 14 | 12 | 13 | 16 | 13,13 | 17 | 12 |
| 634 | 1 | 0.0011 | 18 | 12 | 20 | 27 | 16 | 9 | 23 | 13 | 12 | 10 | 14 | 18 | 21 | 25 | 12 | 13 | 10 | 12 | 19 | 14,18 | 14 | 11 |
| 635 | 1 | 0.0011 | 19 | 14 | 19 | 30 | 13 | 6 | 24 | 12 | 12 | 12 | 14 | 21 | 22 | 24 | 12 | 13 | 10 | 14 | 16 | 16,20 | 17 | 10 |
| 636 | 1 | 0.0011 | 18 | 13 | 20 | 29 | 15 | 10 | 25 | 11 | 12 | 10 | 14 | 18 | 22 | 23 | 11 | 11 | 9 | 14 | 17 | 11,19 | 16 | 12 |
| 637 | 1 | 0.0011 | 18 | 12 | 20 | 28 | 15 | 10 | 23 | 12 | 11 | 10 | 14 | 19 | 23 | 25 | 11 | 13 | 10 | 12 | 18 | 14,17 | 16 | 13 |
| 638 | 1 | 0.0011 | 19 | 14 | 20 | 30 | 14 | 11 | 23 | 12 | 10 | 10 | 15 | 18 | 20 | 28 | 13 | 13 | 12 | 12 | 17 | 13,18 | 16 | 11 |
| 639 | 1 | 0.0011 | 19 | 13 | 20 | 29 | 14 | 10 | 25 | 13 | 11 | 11 | 16 | 17 | 20 | 24 | 13 | 14 | 12 | 12 | 18 | 13,18 | 15 | 12 |
| 640 | 1 | 0.0011 | 17 | 12 | 22 | 27 | 15 | 10 | 24 | 13 | 12 | 10 | 14 | 16 | 21 | 22 | 12 | 11 | 9 | 14 | 16 | 11,18 | 15 | 11 |
| 641 | 1 | 0.0011 | 17 | 12 | 20 | 26 | 17 | 10 | 28 | 12 | 11 | 10 | 14 | 19 | 21 | 25 | 12 | 13 | 10 | 13 | 18 | 13,19 | 14 | 11 |
| 642 | 1 | 0.0011 | 18 | 12 | 18 | 31 | 15 | 11 | 23 | 12 | 10 | 10 | 14 | 18 | 22 | 24 | 11 | 13 | 12 | 15 | 15 | 14,18 | 15 | 10 |
| 643 | 1 | 0.0011 | 20 | 13 | 19 | 30 | 15 | 10 | 25 | 12 | 12 | 10 | 15 | 14 | 21 | 24 | 12 | 13 | 11 | 12 | 20 | 12,17 | 17 | 11 |
| 644 | 1 | 0.0011 | 16 | 12 | 19 | 27 | 17 | 10 | 27 | 13 | 11 | 10 | 14 | 19 | 22 | 23 | 11 | 13 | 10 | 12 | 17 | 14,15 | 14 | 12 |
| 645 | 1 | 0.0011 | 17 | 13 | 21 | 30 | 17 | 10 | 22 | 12 | 10 | 10 | 14 | 18 | 19 | 23 | 13 | 14 | 11 | 12 | 17 | 12,16 | 15 | 12 |
| 646 | 1 | 0.0011 | 20 | 12 | 21 | 29 | 14 | 10 | 23 | 12 | 11 | 11 | 15 | 18 | 20 | 24 | 13 | 14 | 11 | 12 | 17 | 13,20 | 16 | 12 |
| 647 | 1 | 0.0011 | 19 | 14 | 21 | 32 | 15 | 10 | 28 | 11 | 12 | 10 | 14 | 17 | 21 | 23 | 12 | 11 | 8 | 15 | 16 | 12,19 | 15 | 11 |
| 648 | 1 | 0.0011 | 18 | 14 | 21 | 30 | 15 | 10 | 26 | 14 | 13 | 10 | 14 | 16 | 19 | 23 | 11 | 12 | 8 | 15 | 15 | 12,17 | 15 | 11 |
| 649 | 1 | 0.0011 | 20 | 12 | 19 | 29 | 15 | 11 | 22 | 12 | 11 | 10 | 15 | 16 | 19 | 24 | 14 | 12 | 11 | 12 | 19 | 12,16 | 15 | 12 |
| 650 | 1 | 0.0011 | 17 | 12 | 19 | 29 | 13 | 10 | 25 | 12 | 11 | 10 | 14 | 18 | 23 | 26 | 11 | 13 | 10 | 12 | 17 | 12,19 | 14 | 12 |
| 651 | 1 | 0.0011 | 19 | 14 | 18 | 30 | 15 | 10 | 23 | 13 | 12 | 10 | 14 | 19 | 20 | 24 | 12 | 13 | 11 | 13 | 16 | 12,17 | 16 | 12 |
| 652 | 1 | 0.0011 | 20 | 12 | 20 | 27 | 14 | 9 | 24 | 13 | 11 | 11 | 15 | 19 | 21 | 24 | 12 | 14 | 11 | 13 | 17 | 13,18 | 16 | 12 |
| 653 | 1 | 0.0011 | 16 | 12 | 19 | 29 | 15 | 10 | 22 | 12 | 11 | 10 | 15 | 17 | 19 | 23 | 13 | 12 | 11 | 12 | 17 | 13,18 | 15 | 12 |
| 654 | 1 | 0.0011 | 18 | 14 | 17 | 30 | 15 | 10 | 24 | 12 | 11 | 10 | 14 | 22 | 21 | 24 | 11 | 13 | 11 | 13 | 17 | 13,20 | 15 | 12 |
| 655 | 1 | 0.0011 | 17 | 12 | 20 | 28 | 17 | 10 | 25 | 14 | 11 | 10 | 14 | 19 | 21 | 25 | 11 | 13 | 10 | 13 | 17 | 14,18 | 14 | 12 |
| 656 | 1 | 0.0011 | 19 | 12 | 20 | 28 | 15 | 10 | 29 | 12 | 11 | 10 | 15 | 18 | 22 | 22 | 11 | 13 | 11 | 12 | 17 | 12,16 | 14 | 11 |
| 657 | 1 | 0.0011 | 19 | 12 | 19 | 29 | 17 | 10 | 24 | 13 | 11 | 10 | 15 | 18 | 21 | 24 | 12 | 13 | 10 | 12 | 19 | 12,18 | 14 | 12 |
| 658 | 1 | 0.0011 | 20 | 12 | 19 | 28 | 15 | 10 | 23 | 12 | 12 | 10 | 15 | 19 | 21 | 23 | 12 | 12 | 12 | 14 | 16 | 12,16 | 15 | 11 |
| 659 | 1 | 0.0011 | 18 | 13 | 19 | 28 | 15 | 10 | 23 | 13 | 11 | 10 | 15 | 19 | 20 | 22 | 11 | 14 | 10 | 12 | 17 | 11,11 | 16 | 12 |
| 660 | 1 | 0.0011 | 18 | 11 | 19 | 28 | 15 | 10 | 22 | 14 | 11 | 10 | 14 | 17 | 20 | 23 | 12 | 12 | 11 | 12 | 18 | 12,18 | 15 | 12 |
| 661 | 1 | 0.0011 | 19 | 13 | 19 | 29 | 14 | 11 | 22 | 13 | 11 | 10 | 15 | 17 | 20 | 23 | 13 | 12 | 11 | 12 | 17 | 12,16 | 18 | 13 |
| 662 | 1 | 0.0011 | 17 | 13 | 19 | 28 | 14 | 11 | 24 | 12 | 12 | 10 | 14 | 19 | 22 | 24 | 13 | 15 | 11 | 13 | 15 | 11,13 | 15 | 13 |
| 663 | 1 | 0.0011 | 16 | 13 | 19 | 28 | 14 | 10 | 24 | 12 | 12 | 10 | 14 | 18 | 22 | 26 | 11 | 15 | 11 | 13 | 16 | 9,12 | 15 | 12 |
| 664 | 1 | 0.0011 | 16 | 13 | 21 | 29 | 17 | 11 | 25 | 13 | 13 | 10 | 14 | 16 | 23 | 23 | 12 | 11 | 9 | 15 | 17 | 11,18 | 15 | 12 |
| 665 | 1 | 0.0011 | 19 | 12 | 19 | 29 | 15 | 10 | 22 | 12 | 11 | 10 | 15 | 15 | 19 | 23 | 12 | 12 | 11 | 12 | 18 | 12,16 | 15 | 12 |
| 666 | 1 | 0.0011 | 16 | 13 | 18 | 30 | 15 | 10 | 24 | 12 | 10 | 10 | 14 | 17 | 22 | 23 | 11 | 15 | 10 | 15 | 17 | 12,19 | 15 | 12 |
| 667 | 1 | 0.0011 | 19 | 13 | 21 | 31 | 15 | 10 | 25 | 12 | 12 | 10 | 14 | 18 | 21 | 23 | 11 | 11 | 9 | 15 | 18 | 12,18 | 15 | 11 |
| 668 | 1 | 0.0011 | 17 | 13 | 21 | 30 | 16 | 10 | 24 | 12 | 12 | 10 | 14 | 19 | 21 | 24 | 13 | 11 | 9 | 15 | 16 | 11,17 | 15 | 11 |
| 669 | 1 | 0.0011 | 19 | 12 | 18 | 29 | 15 | 11 | 24 | 14 | 11 | 10 | 14 | 19 | 19 | 23 | 11 | 14 | 11 | 13 | 15 | 12,13 | 17 | 12 |
| 670 | 1 | 0.0011 | 20 | 12 | 20 | 28 | 14 | 9 | 22 | 12 | 11 | 11 | 15 | 19 | 20 | 24 | 13 | 14 | 12 | 13 | 20 | 13,19 | 15 | 13 |
| 671 | 1 | 0.0011 | 18 | 14 | 21 | 30 | 15 | 10 | 25 | 12 | 12 | 10 | 14 | 20 | 23 | 23 | 10 | 11 | 8 | 15 | 15 | 11,19 | 15 | 11 |
| 672 | 1 | 0.0011 | 18 | 13 | 18 | 31 | 15 | 10 | 23 | 12 | 10 | 12 | 14 | 19 | 23 | 24 | 12 | 13 | 11 | 14 | 18 | 12,12 | 15 | 11 |
| 673 | 1 | 0.0011 | 16 | 12 | 20 | 29 | 16 | 10 | 25 | 13 | 12 | 10 | 13 | 18 | 21 | 24 | 11 | 13 | 10 | 13 | 19 | 13,18 | 14 | 12 |
| 674 | 1 | 0.0011 | 19 | 13 | 16 | 31 | 14 | 10 | 26 | 13 | 12 | 10 | 14 | 19 | 21 | 24 | 10 | 11 | 9 | 12 | 18 | 14,14 | 15 | 12 |
| 675 | 1 | 0.0011 | 18 | 12 | 18 | 29 | 16 | 11 | 25 | 12 | 11 | 10 | 14 | 18 | 19 | 23 | 11 | 14 | 11 | 14 | 15 | 13,13 | 16 | 12 |
| 676 | 1 | 0.0011 | 19 | 14 | 19 | 30 | 15 | 10 | 24 | 11 | 13 | 10 | 14 | 16 | 21 | 24 | 10 | 13 | 11 | 12 | 17 | 13,19 | 15 | 12 |
| 677 | 1 | 0.0011 | 18 | 14 | 19 | 31 | 14 | 10 | 22 | 12 | 13 | 11 | 14 | 18 | 22 | 23 | 11 | 14 | 10 | 13 | 17 | 11,12 | 16 | 12 |
| 678 | 1 | 0.0011 | 18 | 12 | 19 | 29 | 16 | 10 | 28 | 12 | 11 | 10 | 15 | 17 | 21 | 24 | 12 | 13 | 11 | 12 | 18 | 13,17 | 15 | 12 |
| 679 | 1 | 0.0011 | 17 | 12 | 19 | 29 | 16 | 11 | 25 | 13 | 12 | 10 | 16 | 17 | 22 | 24 | 11 | 12 | 11 | 12 | 15 | 13,20 | 15 | 12 |
| 680 | 1 | 0.0011 | 19 | 13 | 20 | 31 | 16 | 10 | 26 | 13 | 12 | 10 | 14 | 20 | 23 | 25 | 11 | 13 | 12 | 12 | 18 | 15,19 | 15 | 11 |
| 681 | 1 | 0.0011 | 19 | 12 | 18 | 30 | 15 | 11 | 25 | 13 | 11 | 10 | 14 | 19 | 19 | 23 | 11 | 14 | 11 | 13 | 16 | 13,13 | 16 | 12 |
| 682 | 1 | 0.0011 | 18 | 12 | 19 | 29 | 16 | 10 | 29 | 11 | 12 | 11 | 14 | 19 | 21 | 24 | 11 | 13 | 11 | 13 | 19 | 14,19 | 15 | 11 |
| 683 | 1 | 0.0011 | 18 | 14 | 18 | 29 | 15 | 10 | 23 | 12 | 10 | 10 | 14 | 19 | 21 | 25 | 11 | 13 | 11 | 14 | 16 | 12,17 | 14 | 12 |
| 684 | 1 | 0.0011 | 19 | 12 | 20 | 29 | 13 | 10 | 23 | 13 | 11 | 10 | 15 | 17 | 22 | 25 | 12 | 14 | 9 | 13 | 18 | 11,12 | 15 | 12 |
| 685 | 1 | 0.0011 | 16 | 14 | 18 | 30 | 14 | 11 | 21 | 12 | 13 | 10 | 14 | 19 | 20 | 23 | 11 | 14 | 11 | 12 | 19 | 11,12 | 14 | 11 |
| 686 | 1 | 0.0011 | 18 | 13 | 20 | 28 | 17 | 10 | 24 | 13 | 11 | 10 | 14 | 18 | 24 | 24 | 13 | 13 | 10 | 12 | 18 | 12,19 | 14 | 12 |
| 687 | 1 | 0.0011 | 17 | 14 | 20 | 29 | 16 | 11 | 24 | 12 | 12 | 10 | 14 | 16 | 21 | 23 | 13 | 11 | 9 | 14 | 17 | 11,19 | 15 | 10 |
| 688 | 1 | 0.0011 | 18 | 12 | 19 | 29 | 16 | 10 | 26 | 11 | 12 | 11 | 14 | 19 | 21 | 24 | 11 | 13 | 11 | 13 | 19 | 14,19 | 15 | 11 |
| 689 | 1 | 0.0011 | 17 | 13 | 21 | 29 | 17 | 10 | 24 | 13 | 14 | 10 | 14 | 17 | 23 | 20 | 11 | 11 | 9 | 15 | 16 | 10,17 | 15 | 11 |
| 690 | 1 | 0.0011 | 18 | 12 | 20 | 28 | 17 | 10 | 25 | 12 | 11 | 10 | 14 | 17 | 23 | 24 | 11 | 13 | 10 | 12 | 18 | 14,16 | 14 | 12 |
| 691 | 1 | 0.0011 | 21 | 12 | 20 | 26 | 14 | 10 | 23 | 15 | 11 | 11 | 15 | 18 | 20 | 24 | 13 | 14 | 11 | 12 | 20 | 13,20 | 15 | 12 |
| 692 | 1 | 0.0011 | 18 | 12 | 19 | 30 | 15 | 10 | 22 | 11 | 11 | 10 | 15 | 16 | 20 | 23 | 12 | 12 | 11 | 12 | 18 | 12,13 | 15 | 12 |
| 693 | 1 | 0.0011 | 17 | 12 | **19.2** | 28 | 15 | 11 | 24 | 12 | 11 | 10 | 14 | 22 | 23 | 25 | 12 | 13 | 10 | 12 | 17 | 12,12 | 14 | 13 |
| 694 | 1 | 0.0011 | 16 | 13 | 18 | 28 | 15 | 11 | 26 | 13 | 12 | 11 | 14 | 17 | 21 | 23 | 12 | 13 | 11 | 14 | 15 | 12,18 | 16 | 12 |
| 695 | 1 | 0.0011 | 18 | 12 | 21 | 28 | 15 | 10 | 23 | 11 | 11 | 11 | 15 | 19 | 21 | 24 | 11 | 14 | 12 | 12 | 18 | 13,19 | 15 | 11 |
| 696 | 1 | 0.0011 | 16 | 12 | 20 | 29 | 15 | 10 | 26 | 12 | 11 | 12 | 15 | 19 | 22 | 24 | 10 | 13 | 11 | 12 | 15 | 12,18 | 15 | 12 |
| 697 | 1 | 0.0011 | 19 | 12 | 20 | 28 | 15 | 10 | 23 | 12 | 11 | 11 | 15 | 18 | 20 | 24 | 13 | 14 | 11 | 13 | 16 | 13,17 | 15 | 12 |
| 698 | 1 | 0.0011 | 19 | 13 | 18 | 28 | 14 | 10 | 24 | 13 | 10 | 10 | 14 | 19 | 23 | 23 | 13 | 13 | 11 | 14 | 18 | 12,20 | 15 | 11 |
| 699 | 1 | 0.0011 | 20 | 13 | 19 | 29 | 14 | 10 | 21 | 12 | 12 | 9 | 14 | 17 | 20 | 22 | 10 | 14 | 9 | 14 | 16 | 10,13 | 18 | 12 |
| 700 | 1 | 0.0011 | 18 | 12 | 21 | 29 | 14 | 10 | 23 | 13 | 11 | 11 | 15 | 20 | 20 | 24 | 11 | 14 | 11 | 12 | 18 | 14,19 | 15 | 13 |
| 701 | 1 | 0.0011 | 18 | 11 | 19 | 27 | 15 | 10 | 22 | 12 | 11 | 10 | 15 | 17 | 19 | 23 | 12 | 12 | 11 | 12 | 20 | 12,18 | 15 | 12 |
| 702 | 1 | 0.0011 | 19 | 12 | 19 | 28 | 15 | 10 | 21 | 12 | 11 | 10 | 15 | 18 | 19 | 23 | 13 | 12 | 12 | 12 | 18 | 12,13 | 15 | 13 |
| 703 | 1 | 0.0011 | 20 | 12 | 20 | 28 | 14 | 10 | 23 | 12 | 11 | 11 | 15 | 19 | 20 | 24 | 11 | 14 | 10 | 12 | 18 | 15,16 | 15 | 11 |
| 704 | 1 | 0.0011 | 15 | 12 | 20 | 28 | 16 | 10 | 25 | 12 | 11 | 10 | 14 | 21 | 23 | 25 | 11 | 13 | 10 | 12 | 17 | 14,18 | 15 | 12 |
| 705 | 1 | 0.0011 | 19 | 12 | 18 | 29 | 15 | 11 | 25 | 12 | 11 | 10 | 14 | 18 | 20 | 22 | 11 | 14 | 11 | 13 | 15 | 13,13 | 15 | 13 |
| 706 | 1 | 0.0011 | 18 | 14 | 18 | 30 | 14 | 10 | 23 | 12 | 10 | 10 | 14 | 20 | 23 | 24 | 10 | 13 | 11 | 14 | 15 | 12,18 | 15 | 12 |
| 707 | 1 | 0.0011 | 19 | 12 | 20 | 29 | 14 | 10 | 23 | 12 | 11 | 11 | 15 | 18 | 20 | 24 | 12 | 14 | 11 | 12 | 17 | 13,19 | 15 | 13 |
| 708 | 1 | 0.0011 | 18 | 12 | 20 | 28 | 14 | 10 | 23 | 13 | 11 | 11 | 14 | 18 | 20 | 23 | 12 | 14 | 12 | 12 | 17 | 13,19 | 15 | 12 |
| 709 | 1 | 0.0011 | 19 | 12 | 20 | 27 | 14 | 10 | 24 | 13 | 11 | 11 | 15 | 18 | 21 | 24 | 13 | 12 | 11 | 12 | 17 | 13,18 | 15 | 12 |
| 710 | 1 | 0.0011 | 20 | 12 | 19 | 29 | 15 | 10 | 21 | 12 | 11 | 10 | 14 | 16 | 19 | 24 | 13 | 12 | 11 | 12 | 20 | 13,15 | 16 | 12 |
| 711 | 1 | 0.0011 | 19 | 13 | 18 | 30 | 13 | 10 | 26 | 11 | 12 | 10 | 14 | 19 | 21 | 24 | 14 | 11 | 11 | 14 | 20 | 13,15 | 16 | 12 |
| 712 | 1 | 0.0011 | 18 | 13 | 20 | 28 | 15 | 10 | 23 | 12 | 11 | 10 | 15 | 17 | 21 | 22 | 13 | 13 | 11 | 12 | 21 | 11,12 | 15 | 11 |
| 713 | 1 | 0.0011 | 18 | 13 | 18 | 31 | 15 | 10 | 23 | 13 | 10 | 10 | 14 | 20 | 24 | 24 | 13 | 13 | 11 | 14 | 19 | 12,20 | 15 | 11 |
| 714 | 1 | 0.0011 | 17 | 13 | 20 | 31 | 16 | 11 | 24 | 13 | 12 | 11 | 14 | 19 | 23 | 24 | 10 | 11 | 10 | 13 | 15 | 11,15 | 15 | 13 |
| 715 | 1 | 0.0011 | 19 | 12 | 19 | 28 | 13 | 10 | 22 | 13 | 11 | 10 | 15 | 17 | 19 | 23 | 12 | 12 | 11 | 13 | 20 | 12,12 | 15 | 11 |
| 716 | 1 | 0.0011 | 15 | 12 | 20 | 28 | 15 | 10 | 24 | 12 | 11 | 10 | 14 | 19 | 22 | 24 | 11 | 13 | 10 | 12 | 17 | 14,19 | 14 | 12 |
| 717 | 1 | 0.0011 | 17 | 12 | 20 | 28 | 14 | 11 | 23 | 12 | 12 | 11 | 15 | 18 | 20 | 24 | 12 | 14 | 11 | 12 | 17 | 12,17 | 16 | 12 |
| 718 | 1 | 0.0011 | 19 | 12 | 20 | 28 | 14 | 10 | 22 | 13 | 11 | 11 | 16 | 19 | 21 | 25 | 12 | 14 | 11 | 13 | 17 | 13,18 | 15 | 12 |
| 719 | 1 | 0.0011 | 19 | 14 | 18 | 31 | 15 | 10 | 23 | 12 | 11 | 10 | 14 | 18 | 21 | 24 | 11 | 13 | 11 | 14 | 15 | 12,18 | 15 | 12 |
| 720 | 1 | 0.0011 | 19 | 13 | 20 | 29 | 16 | 10 | 24 | 13 | 12 | 10 | 14 | 16 | 21 | 23 | 11 | 11 | 9 | 15 | 16 | 11,11 | 15 | 11 |
| 721 | 1 | 0.0011 | 19 | 13 | 20 | 30 | 16 | 9 | 27 | 11 | 11 | 10 | 14 | 17 | 24 | 24 | 11 | 11 | 10 | 13 | 17 | 12,12 | 15 | 10 |
| 722 | 1 | 0.0011 | 19 | 13 | 19 | 28 | 17 | 10 | 24 | 12 | 12 | 10 | 14 | 18 | 24 | 25 | 12 | 13 | 10 | 12 | 18 | 13,19 | 14 | 12 |
| 723 | 1 | 0.0011 | 17 | 14 | 19 | 31 | 14 | 10 | 22 | 12 | 12 | 10 | 14 | 19 | 23 | 23 | 13 | 14 | 10 | 13 | 14 | 11,13 | 17 | 12 |
| 724 | 1 | 0.0011 | 21 | 12 | 20 | 27 | 13 | 10 | 23 | 12 | 11 | 11 | 15 | 18 | 20 | 24 | 13 | 14 | 11 | 12 | 18 | 14,21 | 15 | 12 |
| 725 | 1 | 0.0011 | 19 | 12 | 20 | 29 | 16 | 10 | 25 | 11 | 12 | 10 | 14 | 19 | 23 | 25 | 11 | 13 | 10 | 12 | 22 | 15,18 | 14 | 12 |
| 726 | 1 | 0.0011 | 18 | 12 | 19 | 29 | 15 | 10 | 22 | 13 | 11 | 10 | 15 | 17 | 19 | 23 | 12 | 12 | 11 | 12 | 17 | 12,18 | 15 | 12 |
| 727 | 1 | 0.0011 | 18 | 12 | 18 | 27 | 15 | 10 | 22 | 13 | 11 | 10 | 15 | 16 | 20 | 23 | 11 | 12 | 10 | 12 | 19 | 13,17 | 15 | 12 |
| 728 | 1 | 0.0011 | 17 | 15 | 19 | 33 | 17 | 10 | 22 | 12 | 11 | 10 | 15 | 19 | 21 | 25 | 12 | 13 | 11 | 12 | 19 | 11,18 | 15 | 10 |
| 729 | 1 | 0.0011 | 16 | 11 | 20 | 27 | 17 | 10 | 24 | 14 | 12 | 10 | 14 | 20 | 24 | 24 | 12 | 13 | 10 | 14 | 22 | 14,17 | 13 | 12 |
| 730 | 1 | 0.0011 | 19 | 13 | 19 | 29 | 15 | 10 | 25 | 14 | 12 | 10 | 15 | 16 | 21 | 24 | 12 | 13 | 11 | 12 | 17 | 11,18 | 17 | 11 |
| 731 | 1 | 0.0011 | 21 | 12 | 18 | 28 | 15 | 12 | 23 | 13 | 11 | 10 | 14 | 15 | 20 | 23 | 12 | 13 | 11 | 13 | 18 | 13,13 | 15 | 12 |
| 732 | 1 | 0.0011 | 19 | 14 | 21 | 31 | 16 | 10 | 23 | 12 | 12 | 10 | 15 | 20 | 22 | 24 | 13 | 13 | 11 | 12 | 16 | 12,19 | 13 | 11 |
| 733 | 1 | 0.0011 | 18 | 12 | 19 | 28 | 15 | 10 | 22 | 12 | 11 | 11 | 15 | 16 | 20 | 23 | 11 | 12 | 11 | 12 | 17 | 12,16 | 15 | 12 |
| 734 | 1 | 0.0011 | 18 | 12 | 18 | 27 | 15 | 10 | 22 | 13 | 11 | 10 | 15 | 16 | 20 | 23 | 11 | 12 | 10 | 12 | 20 | 13,17 | 15 | 12 |
| 735 | 1 | 0.0011 | 19 | 12 | 19 | 29 | 15 | 10 | 26 | 13 | 11 | 10 | 14 | 17 | 20 | 24 | 12 | 13 | 10 | 12 | 17 | 12,19 | 14 | 13 |
| 736 | 1 | 0.0011 | 19 | 12 | 19 | 28 | 15 | 10 | 21 | 12 | 11 | 10 | 14 | 16 | 19 | 23 | 11 | 12 | 11 | 12 | 18 | 12,17 | 16 | 11 |
| 737 | 1 | 0.0011 | 19 | 13 | 19 | 30 | 16 | 10 | 23 | 12 | 11 | 10 | 14 | 16 | 21 | 24 | 11 | 13 | 11 | 13 | 17 | 12,19 | 15 | 11 |
| 738 | 1 | 0.0011 | 18 | 14 | 22 | 30 | 16 | 10 | 24 | 13 | 12 | 10 | 14 | 16 | 23 | 23 | 12 | 11 | 9 | 14 | 18 | 11,17 | 16 | 11 |
| 739 | 1 | 0.0011 | 19 | 12 | 18 | 27 | 15 | 11 | 22 | 13 | 11 | 10 | 15 | 16 | 20 | 23 | 11 | 12 | 10 | 12 | 20 | 13,17 | 15 | 12 |
| 740 | 1 | 0.0011 | 17 | 12 | 20 | 31 | 16 | 10 | 24 | 13 | 11 | 10 | 14 | 18 | 23 | 24 | 11 | 13 | 10 | 12 | 19 | 14,18 | 14 | 12 |
| 741 | 1 | 0.0011 | 19 | 12 | 21 | 28 | 15 | 10 | 23 | 12 | 10 | 11 | 16 | 18 | 21 | 25 | 12 | 14 | 12 | 14 | 17 | 13,13 | 15 | 12 |
| 742 | 1 | 0.0011 | 19 | 13 | 19 | 28 | 15 | 10 | 23 | 13 | 11 | 10 | 15 | 19 | 21 | 24 | 13 | 13 | 11 | 12 | 18 | 11,11 | 16 | 12 |
| 743 | 1 | 0.0011 | 18 | 13 | 21 | 30 | 16 | 10 | 24 | 12 | 14 | 10 | 14 | 15 | 21 | 23 | 12 | 11 | 9 | 15 | 17 | 11,17 | 15 | 11 |
| 744 | 1 | 0.0011 | 20 | 12 | 19 | 28 | 17 | 10 | 24 | 13 | 11 | 10 | 14 | 17 | 22 | 24 | 13 | 13 | 11 | 13 | 19 | 11,21 | 14 | 12 |
| 745 | 1 | 0.0011 | 20 | 12 | 20 | 28 | 14 | 10 | 24 | 12 | 13 | 11 | 15 | 19 | 21 | 24 | 12 | 14 | 11 | 12 | 17 | 12,18 | 15 | 11 |
| 746 | 1 | 0.0011 | 21 | 12 | 19 | 27 | 17 | 10 | 24 | 12 | 11 | 10 | 14 | 18 | 24 | 25 | 13 | 13 | 11 | 12 | 22 | 13,20 | 14 | 13 |
| 747 | 1 | 0.0011 | 17 | 12 | 19 | 28 | 15 | 10 | 26 | 14 | 13 | 9 | 15 | 14 | 21 | 24 | 12 | 13 | 11 | 12 | 15 | 12,21 | 15 | 13 |
| 748 | 1 | 0.0011 | 19 | 12 | 19 | 28 | 15 | 10 | 23 | 12 | 11 | 10 | 14 | 18 | 24 | 24 | 11 | 13 | 10 | 12 | 21 | 12,20 | 15 | 11 |
| 749 | 1 | 0.0011 | 20 | 12 | 19 | 30 | 15 | 10 | 21 | 13 | 11 | 10 | 14 | 16 | 19 | 24 | 12 | 12 | 11 | 12 | 19 | 12,18 | 15 | 12 |
| 750 | 1 | 0.0011 | 22 | 12 | 20 | 28 | 14 | 10 | 23 | 13 | 12 | 11 | 15 | 19 | 22 | 24 | 12 | 15 | 11 | 13 | 17 | 14,20 | 15 | 12 |
| 751 | 1 | 0.0011 | 19 | 12 | 19 | 28 | 15 | 10 | 21 | 12 | 11 | 10 | 14 | 16 | 19 | 23 | 14 | 12 | 11 | 12 | 18 | 12,17 | 16 | 11 |
| 752 | 1 | 0.0011 | 18 | 12 | 20 | 28 | 14 | 10 | 23 | 13 | 11 | 11 | 14 | 19 | 20 | 23 | 11 | 14 | 11 | 12 | 17 | 13,19 | 15 | 12 |
| 753 | 1 | 0.0011 | 16 | 12 | 20 | 29 | 15 | 10 | 25 | 13 | 12 | 11 | 15 | 16 | 21 | 24 | 10 | 13 | 11 | 12 | 17 | 12,18 | 14 | 12 |
| 754 | 1 | 0.0011 | 19 | 12 | 19 | 29 | 15 | 10 | 22 | 12 | 11 | 10 | 15 | 16 | 19 | 23 | 12 | 12 | 11 | 12 | 16 | 12,16 | 16 | 12 |
| 755 | 1 | 0.0011 | 18 | 12 | 19 | 28 | 15 | 10 | 22 | 12 | 11 | 10 | 15 | 16 | 19 | 23 | 11 | 12 | 10 | 12 | 17 | 12,13 | 15 | 12 |
| 756 | 1 | 0.0011 | 18 | 12 | 19 | 28 | 15 | 11 | 21 | 12 | 11 | 10 | 14 | 18 | 21 | 23 | 13 | 12 | 11 | 12 | 17 | 12,16 | 16 | 12 |
| 757 | 1 | 0.0011 | 17 | 12 | 20 | 29 | 14 | 10 | 23 | 12 | 11 | 11 | 15 | 18 | 20 | 24 | 12 | 14 | 12 | 12 | 16 | 13,18 | 15 | 12 |
| 758 | 1 | 0.0011 | 18 | 14 | 21 | 30 | 15 | 10 | 24 | 12 | 12 | 10 | 15 | 20 | 21 | 23 | 11 | 13 | 12 | 13 | 20 | 13,19 | 15 | 12 |
| 759 | 1 | 0.0011 | 19 | 12 | 20 | 28 | 14 | 10 | 25 | 12 | 11 | 11 | 16 | 18 | 20 | 24 | 12 | 14 | 11 | 12 | 17 | 13,18 | 15 | 12 |
| 760 | 1 | 0.0011 | 19 | 12 | 18 | 28 | 14 | 11 | 25 | 12 | 11 | 10 | 14 | 18 | 20 | 24 | 11 | 14 | 11 | 13 | 15 | 13,13 | 16 | 12 |
| 761 | 1 | 0.0011 | 17 | 13 | 18 | 30 | 15 | 10 | 23 | 13 | 11 | 10 | 14 | 20 | 21 | 24 | 11 | 13 | 11 | 14 | 15 | 12,17 | 15 | 11 |
| 762 | 1 | 0.0011 | 17 | 13 | 18 | 28 | 15 | 10 | 26 | 13 | 11 | 11 | 14 | 16 | 21 | 23 | 13 | 13 | 11 | 13 | 16 | 12,17 | 15 | 12 |
| 763 | 1 | 0.0011 | 18 | 12 | 19 | 28 | 14 | 10 | 23 | 13 | 11 | 11 | 15 | 18 | 21 | 25 | 12 | 14 | 11 | 12 | 17 | 13,20 | 15 | 13 |
| 764 | 1 | 0.0011 | 17 | 14 | 21 | 31 | 16 | 10 | 24 | 12 | 14 | 10 | 14 | 16 | 21 | 23 | 11 | 11 | 8 | 15 | 17 | 11,17 | 14 | 11 |
| 765 | 1 | 0.0011 | 18 | 12 | 20 | 28 | 14 | 10 | 24 | 13 | 11 | 11 | 14 | 19 | 20 | 23 | 12 | 14 | 11 | 12 | 17 | 13,19 | 15 | 11 |
| 766 | 1 | 0.0011 | 20 | 12 | 20 | 28 | 14 | 10 | 23 | 12 | 11 | 11 | 14 | 19 | 20 | 23 | 11 | 14 | 11 | 12 | 18 | 13,19 | 15 | 12 |
| 767 | 1 | 0.0011 | 21 | 12 | 19 | 29 | 16 | 8 | 24 | 11 | 11 | 10 | 14 | 16 | 23 | 25 | 12 | 13 | 10 | 12 | 19 | 12,20 | 14 | 11 |
| 768 | 1 | 0.0011 | 17 | 12 | 20 | 27 | 15 | 10 | 24 | 13 | 11 | 11 | 15 | 18 | 21 | 24 | 12 | 14 | 11 | 13 | 17 | 13,18 | 15 | 13 |
| 769 | 1 | 0.0011 | 19 | 12 | 19 | 29 | 15 | 10 | 21 | 12 | 11 | 10 | 14 | 17 | 19 | 23 | 13 | 12 | 10 | 12 | 18 | 12,17 | 15 | 12 |
| 770 | 1 | 0.0011 | 15 | 13 | 22 | 29 | 14 | 10 | 23 | 12 | 13 | 9 | 15 | 17 | 21 | 23 | 11 | 11 | 10 | 12 | 16 | 13,17 | 14 | 12 |
| 771 | 1 | 0.0011 | 18 | 12 | 20 | 28 | 14 | 11 | 23 | 13 | 11 | 11 | 14 | 19 | 20 | 24 | 12 | 14 | 11 | 12 | 17 | 12,19 | 15 | 12 |
| 772 | 1 | 0.0011 | 18 | 14 | 18 | 31 | 15 | 10 | 23 | 12 | 11 | 10 | 14 | 19 | 22 | 24 | 12 | 13 | 11 | 14 | 16 | 12,17 | 15 | 12 |
| 773 | 1 | 0.0011 | 17 | 11 | 19 | 28 | 15 | 11 | 24 | 12 | 12 | 10 | 15 | 15 | 22 | 24 | 11 | 13 | 11 | 13 | 20 | 12,17 | 14 | 11 |
| 774 | 1 | 0.0011 | 19 | 12 | 19 | 28 | 15 | 10 | 26 | 13 | 12 | 10 | 14 | 19 | 22 | 25 | 12 | 13 | 10 | 13 | 19 | 12,18 | 15 | 12 |
| 775 | 1 | 0.0011 | 18 | 12 | 20 | 27 | 17 | 10 | 25 | 13 | 11 | 10 | 14 | 19 | 21 | 25 | 12 | 13 | 10 | 12 | 18 | 12,18 | 14 | 12 |
| 776 | 1 | 0.0011 | 18 | 12 | 20 | 28 | 14 | 10 | 23 | 13 | 11 | 11 | 14 | 19 | 20 | 23 | 11 | 14 | 11 | 12 | 17 | 13,19 | 14 | 11 |
| 777 | 1 | 0.0011 | 17 | 12 | 20 | 29 | 15 | 11 | 24 | 13 | 12 | 10 | 14 | 18 | 21 | 24 | 11 | 13 | 10 | 12 | 18 | 14,19 | 14 | 12 |
| 778 | 1 | 0.0011 | 19 | 12 | 19 | 28 | 15 | 10 | 22 | 12 | 11 | 10 | 15 | 17 | 19 | 23 | 13 | 12 | 12 | 12 | 18 | 11,16 | 15 | 12 |
| 779 | 1 | 0.0011 | 20 | 14 | 21 | 30 | 15 | 10 | 25 | 12 | 13 | 10 | 14 | 16 | 21 | 22 | 11 | 11 | 8 | 15 | 13 | 11,21 | 14 | 11 |
| 780 | 1 | 0.0011 | 18 | 13 | 21 | 29 | 15 | 10 | 25 | 12 | 12 | 10 | 14 | 17 | 23 | 23 | 12 | 11 | 9 | 14 | 17 | 11,18 | 15 | 11 |
| 781 | 1 | 0.0011 | 19 | 12 | 18 | 28 | 15 | 10 | 23 | 11 | 11 | 10 | 15 | 15 | 22 | 23 | 12 | 13 | 11 | 12 | 19 | 13,17 | 17 | 13 |
| 782 | 1 | 0.0011 | 19 | 13 | 20 | 30 | 15 | 9 | 26 | 11 | 12 | 10 | 14 | 18 | 21 | 23 | 13 | 14 | 11 | 13 | 16 | 13,17 | 19 | 12 |
| 783 | 1 | 0.0011 | 19 | 12 | 19 | 27 | 15 | 10 | 22 | 12 | 11 | 10 | 15 | 16 | 19 | 23 | 12 | 12 | 11 | 12 | 17 | 13,16 | 16 | 11 |
| 784 | 1 | 0.0011 | 17 | 12 | 20 | 29 | 15 | 10 | 22 | 13 | 11 | 11 | 15 | 19 | 25 | 23 | 14 | 12 | 11 | 12 | 18 | 12,13 | 15 | 12 |
| 785 | 1 | 0.0011 | 17 | 14 | 21 | 30 | 15 | 10 | 24 | 13 | 12 | 10 | 14 | 16 | 22 | 23 | 11 | 11 | 8 | 15 | 15 | 12,19 | 15 | 11 |
| 786 | 1 | 0.0011 | 19 | 12 | 20 | 28 | 14 | 10 | 24 | 12 | 11 | 11 | 15 | 20 | 22 | 25 | 12 | 14 | 11 | 12 | 19 | 13,19 | 16 | 11 |
| 787 | 1 | 0.0011 | 18 | 12 | 19 | 27 | 15 | 10 | 22 | 13 | 11 | 10 | 15 | 15 | 19 | 23 | 12 | 12 | 11 | 12 | 17 | 12,16 | 15 | 11 |
| 788 | 1 | 0.0011 | 17 | 13 | 21 | 28 | 16 | 10 | 24 | 12 | 12 | 10 | 15 | 16 | 21 | 23 | 11 | 11 | 9 | 14 | 16 | 11,20 | 14 | 11 |
| 789 | 1 | 0.0011 | 16 | 12 | 20 | 28 | 15 | 9 | 23 | 12 | 11 | 10 | 14 | 21 | 22 | 25 | 11 | 13 | 10 | 12 | 17 | 16,18 | 13 | 12 |
| 790 | 1 | 0.0011 | 18 | 13 | 21 | 29 | 17 | 11 | 26 | 14 | 12 | 10 | 14 | 16 | 21 | 23 | 11 | 11 | 9 | 14 | 18 | 11,18 | 15 | 11 |
| 791 | 1 | 0.0011 | 20 | 14 | 20 | 30 | 14 | 10 | 24 | 12 | 10 | 11 | 15 | 20 | 21 | 25 | 12 | 13 | 13 | 12 | 17 | 14,18 | 15 | 11 |
| 792 | 1 | 0.0011 | 21 | 13 | 19 | 29 | 17 | 10 | 24 | 13 | 12 | 10 | 14 | 19 | 22 | 23 | 11 | 13 | 10 | 9 | 18 | 12,19 | 14 | 12 |
| 793 | 1 | 0.0011 | 18 | 14 | 21 | 29 | 15 | 10 | 24 | 13 | 12 | 10 | 14 | 16 | 21 | 23 | 12 | 11 | 9 | 14 | 17 | 19,20 | 14 | 11 |
| 794 | 1 | 0.0011 | 19 | 12 | 19 | 29 | 15 | 10 | 20 | 12 | 11 | 10 | 14 | 16 | 19 | 24 | 12 | 12 | 11 | 12 | 18 | 12,17 | 16 | 12 |
| 795 | 1 | 0.0011 | 17 | 12 | 20 | 28 | 16 | 10 | 22 | 12 | 11 | 10 | 14 | 21 | 23 | 25 | 11 | 13 | 10 | 12 | 17 | 14,18 | 15 | 12 |
| 796 | 1 | 0.0011 | 17 | 12 | 18 | 28 | 15 | 11 | 25 | 12 | 11 | 10 | 14 | 20 | 19 | 23 | 11 | 14 | 11 | 13 | 16 | 13,13 | 17 | 12 |
| 797 | 1 | 0.0011 | 17 | 13 | 21 | 29 | 14 | 10 | 22 | 13 | 13 | 9 | 15 | 17 | 21 | 23 | 11 | 11 | 11 | 12 | 15 | 13,17 | 16 | 12 |
| 798 | 1 | 0.0011 | 18 | 14 | 19 | 31 | 14 | 10 | 23 | 10 | 12 | 11 | 14 | 18 | 22 | 23 | 13 | 14 | 11 | 13 | 15 | 11,12 | 16 | 12 |
| 799 | 1 | 0.0011 | 16 | 12 | 19 | 29 | 16 | 10 | 22 | 14 | 12 | 10 | 15 | 17 | 19 | 23 | 12 | 12 | 11 | 12 | 19 | 12,17 | 15 | 12 |
| 800 | 1 | 0.0011 | 17 | 12 | 20 | 28 | 14 | 11 | 23 | 12 | 11 | 11 | 15 | 20 | 22 | 23 | 12 | 14 | 12 | 12 | 18 | 13,16 | 15 | 12 |
| 801 | 1 | 0.0011 | 17 | 14 | 20 | 30 | 15 | 10 | 25 | 13 | 12 | 10 | 14 | 17 | 21 | 23 | 13 | 11 | 8 | 15 | 16 | 12,18 | 16 | 11 |
| 802 | 1 | 0.0011 | 19 | 12 | 20 | 29 | 17 | 10 | 22 | 12 | 14 | 10 | 15 | 18 | 19 | 23 | 12 | 12 | 12 | 12 | 18 | 12,17 | 15 | 13 |
| 803 | 1 | 0.0011 | 18 | 12 | 18 | 29 | 15 | 11 | 25 | 11 | 11 | 10 | 14 | 18 | 21 | 24 | 12 | 14 | 11 | 13 | 15 | 13,13 | 17 | 12 |
| 804 | 1 | 0.0011 | 18 | 12 | 20 | 28 | 15 | 10 | 24 | 13 | 11 | 10 | 14 | 19 | 21 | 24 | 11 | 13 | 11 | 12 | 16 | 13,18 | 14 | 12 |
| 805 | 1 | 0.0011 | 18 | 13 | 21 | 29 | 16 | 10 | 24 | 13 | 12 | 10 | 14 | 16 | 22 | 23 | 11 | 11 | 9 | 14 | 15 | 12,18 | 15 | 12 |
| 806 | 1 | 0.0011 | 18 | 13 | 19 | 28 | 15 | 11 | 24 | 13 | 12 | 10 | 15 | 15 | 21 | 22 | 12 | 13 | 11 | 12 | 16 | 12,18 | 15 | 11 |
| 807 | 1 | 0.0011 | 17 | 13 | 20 | 30 | 17 | 10 | 25 | 12 | 11 | 10 | 14 | 18 | 22 | 23 | 12 | 13 | 10 | 12 | 17 | 12,19 | 16 | 11 |
| 808 | 1 | 0.0011 | 19 | 12 | 19 | 29 | 15 | 10 | 21 | 12 | 12 | 10 | 14 | 17 | 19 | 23 | 12 | 12 | 12 | 12 | 20 | 12,17 | 15 | 12 |
| 809 | 1 | 0.0011 | 18 | 12 | 21 | 28 | 17 | 10 | 24 | 13 | 11 | 9 | 14 | 17 | 22 | 25 | 12 | 13 | 10 | 12 | 18 | 12,19 | 14 | 12 |
| 810 | 1 | 0.0011 | 20 | 12 | 20 | 28 | 13 | 10 | 23 | 12 | 11 | 11 | 16 | 19 | 20 | 24 | 12 | 14 | 11 | 13 | 19 | 14,17 | 15 | 11 |
| 811 | 1 | 0.0011 | 20 | 14 | 20 | 30 | 14 | 10 | 23 | 13 | 10 | 10 | 15 | 19 | 21 | 25 | 12 | 13 | 12 | 12 | 14 | 13,20 | 16 | 11 |
| 812 | 1 | 0.0011 | 20 | 12 | 19 | 28 | 17 | 10 | 24 | 13 | 13 | 10 | 14 | 19 | 22 | 24 | 12 | 14 | 11 | 12 | 16 | 14,18 | 15 | 11 |
| 813 | 1 | 0.0011 | 18 | 12 | 19 | 27 | 15 | 10 | 21 | 13 | 12 | 10 | 15 | 17 | 23 | 24 | 12 | 12 | 12 | 12 | 18 | 12,12 | 15 | 11 |
| 814 | 1 | 0.0011 | 16 | 12 | 20 | 29 | 15 | 10 | 25 | 12 | 12 | 11 | 15 | 19 | 22 | 24 | 11 | 13 | 11 | 12 | 15 | 12,18 | 15 | 12 |
| 815 | 1 | 0.0011 | 19 | 12 | 19 | 28 | 16 | 10 | 24 | 12 | 11 | 10 | 14 | 20 | 24 | 24 | 12 | 13 | 10 | 12 | 18 | 12,20 | 14 | 11 |
| 816 | 1 | 0.0011 | 19 | 12 | 20 | 26 | 14 | 10 | 23 | 14 | 11 | 11 | 15 | 17 | 20 | 24 | 12 | 13 | 11 | 12 | 18 | 13,22 | 15 | 13 |
| 817 | 1 | 0.0011 | 22 | 12 | 19 | 29 | 15 | 11 | 23 | 13 | 11 | 10 | 15 | 17 | 20 | 23 | 14 | 12 | 12 | 12 | 17 | 12,12 | 16 | 12 |
| 818 | 1 | 0.0011 | 18 | 13 | 19 | 30 | 16 | 11 | 22 | 12 | 12 | 10 | 14 | 16 | 22 | 23 | 11 | 13 | 11 | 12 | 20 | 12,19 | 15 | 11 |
| 819 | 1 | 0.0011 | 14 | 14 | 20 | 32 | 14 | 10 | 22 | 12 | 12 | 10 | 14 | 18 | 23 | 23 | 11 | 14 | 10 | 13 | 15 | 11,12 | 18 | 12 |
| 820 | 1 | 0.0011 | 19 | 12 | 19 | 27 | 15 | 10 | 25 | 13 | 12 | 10 | 15 | 16 | 21 | 24 | 13 | 13 | 12 | 13 | 17 | 14,17 | 15 | 12 |
| 821 | 1 | 0.0011 | 20 | 12 | 19 | 27 | 15 | 11 | 25 | 14 | 11 | 10 | 14 | 18 | 21 | 25 | 13 | 13 | 10 | 12 | 19 | 12,18 | 14 | 11 |
| 822 | 1 | 0.0011 | 18 | 13 | 20 | 29 | 16 | 10 | 23 | 13 | 10 | 10 | 15 | 18 | 21 | 23 | 12 | 13 | 11 | 12 | 19 | 12,21 | 13 | 12 |
| 823 | 1 | 0.0011 | 10 | 12 | 19 | 28 | 13 | 9 | 24 | 12 | 12 | 12 | 14 | 20 | 22 | 24 | 12 | 14 | 10 | 14 | 16 | 15,22 | 15 | 10 |
| 824 | 1 | 0.0011 | 18 | 12 | 19 | 29 | 16 | 10 | 25 | 13 | 12 | 10 | 15 | 15 | 21 | 25 | 11 | 13 | 11 | 12 | 16 | 12,20 | 17 | 11 |
| 825 | 1 | 0.0011 | 20 | 12 | 19 | 30 | 15 | 10 | 22 | 12 | 11 | 8 | 15 | 16 | 19 | 23 | 13 | 12 | 10 | 13 | 19 | 12,13 | 15 | 12 |
| 826 | 1 | 0.0011 | 18 | 12 | 20 | 28 | 15 | 10 | 26 | 12 | 12 | 10 | 14 | 22 | 20 | 24 | 11 | 10 | 9 | 12 | 20 | 17,17 | 14 | 12 |
| 827 | 1 | 0.0011 | 18 | 12 | 18 | 29 | 15 | 11 | 26 | 12 | 11 | 10 | 14 | 18 | 19 | 23 | 11 | 14 | 11 | 13 | 15 | 13,13 | 16 | 12 |
| 828 | 1 | 0.0011 | 19 | 13 | 20 | 30 | 15 | 10 | 27 | 12 | 12 | 10 | 14 | 16 | 22 | 23 | 11 | 11 | 8 | 15 | 15 | 10,11 | 15 | 11 |
| 829 | 1 | 0.0011 | 18 | 12 | 19 | 28 | 13 | 10 | 22 | 15 | 12 | 10 | 15 | 19 | 21 | 25 | 13 | 13 | 9 | 14 | 18 | 12,12 | 14 | 12 |
| 830 | 1 | 0.0011 | 22 | 13 | 19 | 31 | 15 | 10 | 22 | 12 | 11 | 10 | 15 | 16 | 19 | 24 | 11 | 12 | 11 | 12 | 17 | 12,13 | 15 | 12 |
| 831 | 1 | 0.0011 | 17 | 14 | 19 | 29 | 16 | 10 | 23 | 13 | 12 | 10 | 15 | 18 | 22 | 22 | 11 | 14 | 10 | 12 | 17 | 11,11 | 16 | 12 |
| 832 | 1 | 0.0011 | 17 | 13 | 20 | 31 | 16 | 11 | 24 | 12 | 13 | 10 | 14 | 16 | 21 | 24 | 11 | 11 | 9 | 13 | 17 | 11,17 | 15 | 11 |
| 833 | 1 | 0.0011 | 19 | 12 | 19 | 29 | 13 | 11 | 23 | 12 | 11 | 11 | 15 | 18 | 22 | 24 | 12 | 14 | 11 | 13 | 17 | 13,20 | 16 | 12 |
| 834 | 1 | 0.0011 | 17 | 13 | 20 | 31 | 15 | 10 | 23 | 13 | 12 | 11 | 15 | 19 | 23 | 24 | 10 | 11 | 10 | 13 | 16 | 11,12 | 16 | 13 |
| 835 | 1 | 0.0011 | 17 | 12 | 20 | 28 | 14 | 10 | 23 | 13 | 11 | 11 | 15 | 20 | 21 | 23 | 12 | 14 | 10 | 12 | 18 | 13,20 | 15 | 12 |
| 836 | 1 | 0.0011 | 20 | 12 | 19 | 30 | 14 | 10 | 24 | 12 | 12 | 10 | 15 | 15 | 19 | 23 | 11 | 12 | 11 | 12 | 17 | 12,12 | 15 | 12 |
| 837 | 1 | 0.0011 | 18 | 13 | 18 | 29 | 15 | 10 | 23 | 14 | 10 | 10 | 14 | 20 | 22 | 23 | 13 | 13 | 11 | 14 | 17 | 12,20 | 15 | 11 |
| 838 | 1 | 0.0011 | 18 | 12 | 19 | 29 | 14 | 10 | 22 | 14 | 12 | 10 | 15 | 16 | 19 | 22 | 11 | 12 | 11 | 12 | 21 | 12,17 | 15 | 12 |
| 839 | 1 | 0.0011 | 18 | 12 | 20 | 28 | 17 | 10 | 26 | 13 | 11 | 10 | 14 | 19 | 23 | 25 | 11 | 13 | 10 | 12 | 16 | 14,17 | 14 | 12 |
| 840 | 1 | 0.0011 | 19 | 14 | 19 | 30 | 16 | 10 | 24 | 13 | 12 | 10 | 14 | 20 | 21 | 24 | 12 | 13 | 11 | 12 | 18 | 13,19 | 15 | 11 |
| 841 | 1 | 0.0011 | 17 | 13 | 18 | 30 | 16 | 10 | 23 | 12 | 10 | 10 | 14 | 19 | 21 | 25 | 12 | 13 | 12 | 14 | 18 | 13,19 | 16 | 11 |
| 842 | 1 | 0.0011 | 19 | 12 | 19 | 28 | 17 | 10 | 25 | 14 | 11 | 10 | 14 | 16 | 20 | 25 | 12 | 13 | 10 | 12 | 17 | 12,20 | 14 | 12 |
| 843 | 1 | 0.0011 | 17 | 14 | 20 | 29 | 14 | 10 | 23 | 12 | 11 | 10 | 15 | 16 | 20 | 25 | 11 | 11 | 11 | 13 | 16 | 13,18 | 14 | 12 |
| 844 | 1 | 0.0011 | 18 | 12 | 20 | 26 | 14 | 10 | 23 | 13 | 11 | 10 | 15 | 17 | 20 | 24 | 13 | 14 | 11 | 12 | 17 | 13,21 | 15 | 12 |
| 845 | 1 | 0.0011 | 18 | 12 | 18 | 27 | 15 | 11 | 25 | 12 | 11 | 10 | 14 | 18 | 19 | 23 | 13 | 15 | 11 | 13 | 15 | 13,13 | 17 | 12 |
| 846 | 1 | 0.0011 | 18 | 12 | 20 | 27 | 14 | 10 | 23 | 13 | 11 | 11 | 14 | 19 | 20 | 23 | 11 | 14 | 11 | 12 | 17 | 13,20 | 15 | 12 |
| 847 | 1 | 0.0011 | 17 | 12 | 19 | 28 | 15 | 10 | 24 | 13 | 11 | 10 | 14 | 17 | 23 | 25 | 11 | 13 | 11 | 12 | 18 | 12,18 | 14 | 11 |
| 848 | 1 | 0.0011 | 19 | 12 | 19 | 30 | 15 | 10 | 22 | 12 | 11 | 10 | 14 | 16 | 20 | 23 | 13 | 12 | 12 | 12 | 18 | 12,16 | 15 | 11 |
| 849 | 1 | 0.0011 | 21 | 13 | 21 | 29 | 17 | 10 | 24 | 12 | 11 | 9 | 14 | 17 | 22 | 24 | 12 | 13 | 10 | 12 | 19 | 12,20 | 13 | 12 |
| 850 | 1 | 0.0011 | 18 | 12 | 19 | 29 | 14 | 11 | 22 | 13 | 11 | 10 | 16 | 18 | 20 | 23 | 12 | 12 | 11 | 12 | 19 | 12,16 | 16 | 12 |
| 851 | 1 | 0.0011 | 18 | 14 | 21 | 29 | 14 | 10 | 24 | 12 | 13 | 10 | 14 | 15 | 21 | 23 | 14 | 11 | 9 | 13 | 17 | 18,18 | 16 | 11 |
| 852 | 1 | 0.0011 | 19 | 14 | 18 | 31 | 15 | 10 | 23 | 11 | 12 | 10 | 14 | 21 | 21 | 22 | 11 | 14 | 10 | 13 | 19 | 11,14 | 15 | 11 |
| 853 | 1 | 0.0011 | 17 | 14 | 19 | 28 | 15 | 10 | 23 | 13 | 11 | 10 | 14 | 19 | 22 | 23 | 11 | 12 | 12 | 14 | 17 | 15,17 | 15 | 13 |
| 854 | 1 | 0.0011 | 19 | 13 | 18 | 29 | 15 | 11 | 25 | 12 | 11 | 10 | 14 | 18 | 19 | 23 | 11 | 14 | 11 | 13 | 15 | 13,13 | 15 | 12 |
| 855 | 1 | 0.0011 | 19 | 12 | 19 | 25 | 16 | 9 | 26 | 11 | 11 | 10 | 14 | 17 | 20 | 24 | 12 | 13 | 10 | 12 | 17 | 13,19 | 14 | 12 |
| 856 | 1 | 0.0011 | 17 | 12 | 22 | 28 | 16 | 10 | 24 | 14 | 12 | 10 | 14 | 17 | 21 | 23 | 11 | 11 | 9 | 14 | 17 | 11,16 | 16 | 11 |
| 857 | 1 | 0.0011 | 17 | 12 | 20 | 28 | 14 | 10 | 23 | 12 | 12 | 11 | 15 | 17 | 20 | 25 | 12 | 14 | 11 | 12 | 16 | 13,20 | 15 | 12 |
| 858 | 1 | 0.0011 | 20 | 12 | 18 | 28 | 15 | 10 | 22 | 12 | 11 | 10 | 15 | 18 | 23 | 24 | 11 | 12 | 12 | 12 | 18 | 11,19 | 15 | 11 |
| 859 | 1 | 0.0011 | 18 | 12 | 19 | 28 | 15 | 10 | 24 | 13 | 11 | 10 | 15 | 17 | 21 | 23 | 13 | 12 | 11 | 12 | 17 | 12,14 | 17 | 11 |
| 860 | 1 | 0.0011 | 17 | 13 | 19 | 28 | 16 | 10 | 26 | 13 | 11 | 10 | 15 | 18 | 24 | 25 | 12 | 13 | 9 | 12 | 21 | 13,18 | 15 | 13 |
| 861 | 1 | 0.0011 | 19 | 12 | 20 | 27 | 14 | 10 | 22 | 12 | 12 | 11 | 15 | 18 | 20 | 23 | 12 | 14 | 11 | 12 | 17 | 13,19 | 15 | 12 |
| 862 | 1 | 0.0011 | 20 | 12 | 19 | 30 | 15 | 10 | 22 | 12 | 11 | 8 | 15 | 16 | 19 | 23 | 12 | 12 | 10 | 13 | 20 | 12,13 | 15 | 12 |
| 863 | 1 | 0.0011 | 19 | 12 | 18 | 29 | 16 | 11 | 25 | 12 | 11 | 10 | 14 | 16 | 20 | 23 | 11 | 14 | 11 | 13 | 15 | 13,13 | 17 | 12 |
| 864 | 1 | 0.0011 | 19 | 12 | 20 | 30 | 17 | 10 | 25 | 11 | 11 | 10 | 14 | 18 | 22 | 24 | 11 | 14 | 11 | 12 | 18 | 13,13 | 15 | 12 |
| 865 | 1 | 0.0011 | 16 | 12 | 19 | 28 | 16 | 10 | 24 | 13 | 11 | 10 | 14 | 18 | 21 | 23 | 11 | 13 | 10 | 12 | 17 | 12,20 | 14 | 12 |
| 866 | 1 | 0.0011 | 17 | 12 | 20 | 28 | 14 | 10 | 24 | 12 | 11 | 10 | 15 | 18 | 20 | 24 | 12 | 14 | 11 | 12 | 16 | 13,19 | 15 | 13 |
| 867 | 1 | 0.0011 | 18 | 13 | 20 | 29 | 17 | 11 | 24 | 14 | 11 | 10 | 14 | 18 | 22 | 23 | 12 | 13 | 10 | 12 | 18 | 12,20 | 15 | 11 |
| 868 | 1 | 0.0011 | 17 | 13 | 18 | 28 | 15 | 10 | 25 | 12 | 10 | 10 | 14 | 18 | 22 | 25 | 11 | 13 | 13 | 14 | 17 | 13,18 | 16 | 10 |
| 869 | 1 | 0.0011 | 19 | 12 | 20 | 29 | 16 | 11 | 25 | 12 | 11 | 9 | 14 | 17 | 21 | 25 | 12 | 13 | 10 | 12 | 18 | 12,22 | 14 | 12 |
| 870 | 1 | 0.0011 | 20 | 12 | 20 | 27 | 14 | 11 | 23 | 12 | 12 | 11 | 15 | 20 | 20 | 24 | 11 | 14 | 11 | 12 | 18 | 13,18 | 15 | 11 |
| 871 | 1 | 0.0011 | 16 | 13 | 18 | 28 | 15 | 10 | 23 | 12 | 10 | 10 | 14 | 17 | 21 | 23 | 11 | 15 | 10 | 14 | 17 | 12,19 | 15 | 12 |
| 872 | 1 | 0.0011 | 15 | 12 | 21 | 30 | 14 | 11 | 23 | 11 | 12 | 10 | 14 | 18 | 22 | 25 | 10 | 14 | 11 | 13 | 16 | 9,12 | 15 | 12 |
| 873 | 1 | 0.0011 | 18 | 13 | 22 | 29 | 16 | 10 | 26 | 13 | 13 | 10 | 14 | 17 | 22 | 23 | 12 | 11 | 9 | 14 | 17 | 11,18 | 16 | 11 |
| 874 | 1 | 0.0011 | 16 | 12 | 20 | 28 | 16 | 11 | 25 | 12 | 11 | 10 | 14 | 20 | 23 | 25 | 12 | 13 | 10 | 12 | 16 | 14,18 | 14 | 12 |
| 875 | 1 | 0.0011 | 17 | 12 | 18 | 28 | 15 | 11 | 26 | 12 | 11 | 10 | 14 | 18 | 19 | 23 | 12 | 14 | 11 | 13 | 15 | 13,13 | 17 | 12 |
| 876 | 1 | 0.0011 | 19 | 12 | 20 | 27 | 14 | 10 | 23 | 12 | 11 | 11 | 15 | 20 | 21 | 24 | 12 | 14 | 11 | 12 | 18 | 11,18 | 15 | 12 |
| 877 | 1 | 0.0011 | 20 | 13 | 19 | 31 | 15 | 10 | 23 | 13 | 11 | 10 | 14 | 16 | 21 | 24 | 11 | 13 | 12 | 13 | 17 | 12,19 | 15 | 11 |
| 878 | 1 | 0.0011 | 17 | 13 | 20 | 29 | 16 | 10 | 24 | 15 | 14 | 10 | 14 | 16 | 22 | 23 | 12 | 11 | 9 | 15 | 17 | 11,18 | 17 | 12 |
| 879 | 1 | 0.0011 | 17 | 12 | 20 | 28 | 16 | 10 | 27 | 12 | 11 | 10 | 14 | 18 | 21 | 23 | 11 | 13 | 11 | 12 | 19 | 13,13 | 15 | 12 |
| 880 | 1 | 0.0011 | 19 | 12 | 19 | 28 | 15 | 10 | 22 | 12 | 11 | 10 | 15 | 17 | 19 | 23 | 13 | 12 | 11 | 12 | 18 | 13,16 | 15 | 12 |
| 881 | 1 | 0.0011 | 17 | 12 | 20 | 28 | 14 | 10 | 22 | 12 | 12 | 11 | 15 | 17 | 20 | 24 | 13 | 14 | 11 | 12 | 17 | 13,18 | 17 | 13 |
| 882 | 1 | 0.0011 | 19 | 14 | 18 | 30 | 15 | 11 | 24 | 13 | 11 | 10 | 14 | 19 | 21 | 24 | 11 | 13 | 11 | 14 | 14 | 12,17 | 16 | 12 |
| 883 | 1 | 0.0011 | 20 | 12 | 20 | 28 | 14 | 10 | 23 | 13 | 12 | 11 | 14 | 19 | 20 | 23 | 11 | 14 | 11 | 13 | 18 | 13,18 | 15 | 12 |
| 884 | 1 | 0.0011 | 16 | 13 | 20 | 31 | 15 | 10 | 25 | 12 | 12 | 11 | 14 | 16 | 21 | 23 | 12 | 11 | 9 | 14 | 16 | 11,18 | 15 | 12 |
| 885 | 1 | 0.0011 | 19 | 14 | 20 | 30 | 15 | 10 | 24 | 14 | 12 | 10 | 14 | 17 | 21 | 23 | 11 | 11 | 9 | 14 | 15 | 11,18 | 16 | 12 |
| 886 | 1 | 0.0011 | 19 | 12 | 20 | 29 | 15 | 10 | 24 | 13 | 12 | 10 | 14 | 17 | 20 | 23 | 13 | 13 | 11 | 13 | 17 | 12,20 | 15 | 12 |
| 887 | 1 | 0.0011 | 20 | 13 | 21 | 29 | 16 | 10 | 24 | 11 | 14 | 10 | 14 | 16 | 21 | 23 | 11 | 11 | 9 | 15 | 15 | 11,18 | 15 | 11 |
| 888 | 1 | 0.0011 | 18 | 14 | 18 | 31 | 15 | 10 | 19 | 10 | 10 | 10 | 14 | 17 | 21 | 23 | 11 | 15 | 10 | 15 | 17 | 12,19 | 15 | 12 |
| 889 | 1 | 0.0011 | 18 | 12 | 20 | 28 | 14 | 10 | 23 | 11 | 11 | 11 | 14 | 18 | 20 | 24 | 12 | 14 | 11 | 12 | 16 | 12,20 | 15 | 12 |
| 890 | 1 | 0.0011 | 16 | 12 | 18 | 29 | 17 | 10 | 25 | 12 | 12 | 10 | 14 | 19 | 22 | 23 | 13 | 14 | 11 | 13 | 15 | 14,14 | 15 | 12 |
| 891 | 1 | 0.0011 | 19 | 12 | 19 | 29 | 16 | 10 | 21 | 13 | 11 | 10 | 15 | 17 | 21 | 23 | 14 | 12 | 11 | 12 | 18 | 12,17 | 15 | 13 |
| 892 | 1 | 0.0011 | 18 | 12 | 18 | 29 | 16 | 11 | 25 | 12 | 11 | 10 | 14 | 18 | 19 | 23 | 11 | 14 | 11 | 13 | 15 | 13,13 | 16 | 13 |
| 893 | 1 | 0.0011 | 17 | 14 | 19 | 30 | 14 | 11 | 22 | 9 | 12 | 11 | 14 | 19 | 25 | 23 | 11 | 15 | 10 | 13 | 15 | 12,12 | 16 | 12 |
| 894 | 1 | 0.0011 | 20 | 12 | 18 | 27 | 16 | 11 | 23 | 13 | 11 | 10 | 14 | 18 | 21 | 23 | 13 | 14 | 11 | 13 | 14 | 13,13 | 16 | 12 |
| 895 | 1 | 0.0011 | 18 | 12 | 18 | 29 | 15 | 11 | 25 | 12 | 11 | 10 | 14 | 18 | 19 | 23 | 11 | 14 | 12 | 13 | 14 | 13,13 | 17 | 12 |
| 896 | 1 | 0.0011 | 18 | 12 | 19 | 28 | 13 | 10 | 22 | 12 | 12 | 10 | 15 | 18 | 22 | 25 | 13 | 13 | 9 | 13 | 18 | 12,12 | 14 | 12 |
| 897 | 1 | 0.0011 | 18 | 12 | 18 | 28 | 15 | 10 | 22 | 13 | 11 | 10 | 15 | 17 | 23 | 23 | 12 | 12 | 12 | 12 | 16 | 11,18 | 16 | 11 |
| 898 | 1 | 0.0011 | 19 | 12 | 19 | 28 | 15 | 11 | 23 | 12 | 11 | 10 | 15 | 16 | 19 | 23 | 11 | 11 | 11 | 12 | 20 | 12,12 | 15 | 12 |
| 899 | 1 | 0.0011 | 18 | 13 | 18 | 29 | 14 | 10 | 23 | 12 | 10 | 10 | 14 | 20 | 21 | 24 | 13 | 13 | 10 | 14 | 14 | 12,21 | 15 | 11 |
| 900 | 1 | 0.0011 | 18 | 12 | 19 | 28 | 14 | 10 | 22 | 12 | 11 | 10 | 15 | 16 | 19 | 23 | 11 | 12 | 12 | 12 | 18 | 12,16 | 15 | 12 |
| 901 | 1 | 0.0011 | 18 | 12 | 20 | 28 | 14 | 11 | 23 | 11 | 11 | 11 | 14 | 18 | 21 | 24 | 13 | 14 | 11 | 12 | 16 | 13,21 | 15 | 12 |
| 902 | 1 | 0.0011 | 19 | 12 | 19 | 28 | 15 | 10 | 21 | 12 | 11 | 10 | 14 | 16 | 19 | 23 | 12 | 12 | 11 | 12 | 19 | 12,18 | 15 | 12 |
| 903 | 1 | 0.0011 | 17 | 13 | 18 | 29 | 15 | 10 | 24 | 12 | 10 | 10 | 14 | 20 | 22 | 24 | 11 | 13 | 12 | 14 | 15 | 12,16 | 16 | 12 |
| 904 | 1 | 0.0011 | 18 | 12 | 18 | 29 | 15 | 10 | 26 | 12 | 11 | 10 | 14 | 18 | 19 | 23 | 11 | 13 | 11 | 13 | 15 | 13,14 | 17 | 12 |
| 905 | 1 | 0.0011 | 18 | 12 | 18 | 29 | 15 | 10 | 23 | 11 | 10 | 10 | 14 | 18 | 22 | 24 | 11 | 13 | 12 | 13 | 15 | 15,17 | 15 | 10 |
| 906 | 1 | 0.0011 | 18 | 12 | 19 | 27 | 15 | 10 | 24 | 12 | 12 | 10 | 14 | 19 | 24 | 25 | 12 | 13 | 10 | 12 | 18 | 13,19 | 14 | 13 |
| 907 | 1 | 0.0011 | 19 | 12 | 18 | 30 | 15 | 11 | 25 | 11 | 11 | 10 | 14 | 18 | 19 | 23 | 11 | 14 | 11 | 13 | 15 | 13,13 | 17 | 12 |
| 908 | 1 | 0.0011 | 18 | 12 | 19 | 29 | 15 | 10 | 28 | 11 | 12 | 10 | 14 | 20 | 21 | 25 | 11 | 10 | 9 | 12 | 22 | 16,16 | 15 | 12 |
| 909 | 1 | 0.0011 | 19 | 12 | 19 | 27 | 16 | 10 | 22 | 14 | 11 | 11 | 14 | 18 | 20 | 25 | 12 | 14 | 11 | 12 | 17 | 13,17 | 14 | 12 |
| 910 | 1 | 0.0011 | 18 | 12 | 21 | 27 | 14 | 10 | 24 | 12 | 11 | 12 | 15 | 18 | 22 | 24 | 12 | 14 | 11 | 13 | 17 | 14,18 | 15 | 12 |
| 911 | 1 | 0.0011 | 18 | 12 | 19 | 29 | 17 | 10 | 24 | 12 | 11 | 10 | 14 | 17 | 21 | 23 | 14 | 13 | 10 | 12 | 20 | 12,16 | 14 | 12 |
| 912 | 1 | 0.0011 | 17 | 12 | 22 | 28 | 14 | 10 | 23 | 12 | 11 | 11 | 15 | 19 | 20 | 24 | 12 | 14 | 10 | 12 | 16 | 13,20 | 16 | 12 |

The value of "0" indicates the deletion of STR; Microvariants are labeled in bold form.
