# Supplementary material for: Inner and inter population structure construction of Chinese Jiangsu Han population based on Y23 STR system
Source: PLoS One. 2017 Jul 13;12(7):e0180921. doi: 10.1371/journal.pone.0180921 (PMC5509181; doi:10.1371/journal.pone.0180921)
Supplement: S2 Table — The value of "0" indicates the deletion of STR; Microvariants are labeled in bold form. (DOCX) [file pone.0180921.s002.docx]

**S2 Table. The distribution of 916 Jiangsu Han samples in prefecture-level geographic location.**

| Sample name | Y loci | | | | | | | | | | | | | | | | | | | | | |
| --- | --- | --- | --- | --- | --- | --- | --- | --- | --- | --- | --- | --- | --- | --- | --- | --- | --- | --- | --- | --- | --- | --- |
|  | DYS  576 | DYS389I | DYS448 | DYS  389II | DYS  19 | DYS  391 | DYS  481 | DYS  549 | DYS  533 | DYS  438 | DYS  437 | DYS  570 | DYS  635 | DYS  390 | DYS  439 | DYS  392 | DYS  643 | DYS  393 | DYS  458 | DYS  385 | DYS  456 | YGATAH4 |
| Changzhou001 | 17 | 12 | 18 | 30 | 15 | 10 | 24 | 12 | 11 | 10 | 14 | 16 | 20 | 23 | 11 | 14 | 11 | 13 | 15 | 13,14 | 15 | 11 |
| Changzhou002 | 19 | 12 | 19 | 28 | 13 | 11 | 22 | 12 | 11 | 10 | 15 | 17 | 19 | 23 | 12 | 12 | 11 | 12 | 18 | 12,12 | 15 | 12 |
| Changzhou003 | 19 | 13 | 21 | 29 | 16 | 10 | 23 | 14 | 12 | 10 | 15 | 16 | 21 | 24 | 12 | 11 | 9 | 15 | 16 | 11,20 | 15 | 11 |
| Changzhou004 | 18 | 12 | 19 | 27 | 15 | 10 | 23 | 12 | 13 | 10 | 15 | 16 | 21 | 24 | 11 | 13 | 11 | 14 | 17 | 14,18 | 15 | 12 |
| Changzhou005 | 16 | 13 | 21 | 31 | 15 | 10 | 26 | 12 | 12 | 10 | 14 | 16 | 20 | 23 | 11 | 11 | 8 | 14 | 16 | 11,19 | 15 | 12 |
| Changzhou006 | 14 | 13 | 19 | 29 | 14 | 10 | 22 | 12 | 12 | 10 | 14 | 17 | 24 | 23 | 11 | 14 | 10 | 13 | 15 | 11,12 | 16 | 12 |
| Changzhou007 | 18 | 12 | 20 | 28 | 15 | 10 | 23 | 12 | 11 | 10 | 14 | 18 | 21 | 24 | 12 | 13 | 11 | 12 | 16 | 14,20 | 14 | 12 |
| Changzhou008 | 18 | 14 | 19 | 30 | 14 | 10 | 23 | 12 | 12 | 10 | 14 | 19 | 21 | 22 | 10 | 14 | 10 | 14 | 14 | 12,12 | 15 | 11 |
| Changzhou009 | 21 | 12 | 19 | 27 | 15 | 10 | 24 | 13 | 11 | 10 | 16 | 19 | 20 | 23 | 13 | 12 | 11 | 13 | 19 | 13,16 | 15 | 10 |
| Changzhou010 | 19 | 12 | 19 | 27 | 16 | 10 | 22 | 14 | 11 | 11 | 14 | 18 | 20 | 25 | 13 | 14 | 11 | 12 | 18 | 13,17 | 15 | 12 |
| Changzhou011 | 15 | 12 | 20 | 27 | 17 | 10 | 26 | 13 | 11 | 10 | 14 | 18 | 21 | 24 | 11 | 13 | 9 | 13 | 18 | 13,18 | 14 | 11 |
| Changzhou012 | 18 | 13 | 20 | 29 | 14 | 11 | 23 | 13 | 11 | 11 | 14 | 19 | 20 | 23 | 12 | 14 | 11 | 12 | 18 | 12,20 | 15 | 12 |
| Changzhou013 | 20 | 13 | 19 | 31 | 15 | 10 | 22 | 12 | 13 | 10 | 15 | 16 | 19 | 23 | 12 | 12 | 11 | 12 | 19 | 12,12 | 15 | 12 |
| Changzhou014 | 20 | 14 | 19 | 30 | 13 | 9 | 24 | 11 | 12 | 12 | 14 | 18 | 22 | 24 | 12 | 14 | 10 | 14 | 17 | 15,22 | 15 | 10 |
| Changzhou015 | 17 | 12 | 20 | 28 | 14 | 10 | 23 | 12 | 10 | 11 | 15 | 18 | 20 | 24 | 12 | 14 | 12 | 11 | 17 | 13,19 | 15 | 12 |
| Changzhou016 | 17 | 13 | 20 | 29 | 15 | 11 | 23 | 12 | 12 | 10 | 15 | 14 | 21 | 24 | 12 | 13 | 11 | 12 | 15 | 12,17 | 16 | 11 |
| Changzhou017 | 18 | 14 | 18 | 32 | 15 | 10 | 22 | 12 | 11 | 10 | 14 | 20 | 22 | 24 | 11 | 13 | 11 | 14 | 15 | 12,17 | 13 | 12 |
| Changzhou018 | 20 | 13 | 21 | 30 | 17 | 10 | 26 | 14 | 13 | 10 | 14 | 18 | 21 | 23 | 12 | 11 | 8 | 15 | 16 | 12,18 | 15 | 11 |
| Changzhou019 | 19 | 13 | 21 | 29 | 17 | 11 | 24 | 12 | 12 | 10 | 14 | 16 | 22 | 22 | 11 | 11 | 9 | 14 | 15 | 13,17 | 15 | 11 |
| Changzhou020 | 18 | 14 | 20 | 29 | 14 | 10 | 22 | 12 | 11 | 10 | 14 | 19 | 23 | 23 | 10 | 14 | 10 | 13 | 15 | 11,11 | 17 | 12 |
| Changzhou021 | 20 | 13 | 20 | 29 | 13 | 9 | 23 | 13 | 12 | 12 | 14 | 20 | 22 | 24 | 10 | 14 | 10 | 13 | 15 | 14,21 | 15 | 10 |
| Changzhou022 | 17 | 14 | 20 | 31 | 16 | 10 | 23 | 12 | 11 | 10 | 15 | 16 | 21 | 25 | 12 | 13 | 11 | 13 | 18 | 14,20 | 13 | 12 |
| Changzhou023 | 17 | 12 | 19 | 28 | 16 | 11 | 24 | 12 | 12 | 10 | 14 | 20 | 24 | 25 | 11 | 13 | 10 | 12 | 18 | 11,19 | 14 | 11 |
| Changzhou024 | 16 | 13 | 18 | 28 | 14 | 10 | 25 | 12 | 12 | 10 | 15 | 19 | 20 | 23 | 13 | 14 | 11 | 13 | 15 | 13,14 | 15 | 12 |
| Changzhou025 | 19 | 12 | 20 | 28 | 14 | 11 | 23 | 12 | 11 | 11 | 15 | 18 | 20 | 23 | 12 | 14 | 11 | 13 | 21 | 14,18 | 15 | 11 |
| Changzhou026 | 19 | 13 | 21 | 30 | 17 | 10 | 25 | 12 | 12 | 10 | 14 | 18 | 21 | 23 | 13 | 11 | 8 | 14 | 17 | 11,18 | 15 | 11 |
| Changzhou027 | 17 | 14 | 20 | 31 | 14 | 10 | 23 | 12 | 11 | 10 | 14 | 18 | 22 | 23 | 12 | 14 | 10 | 13 | 14 | 11,11 | 17 | 12 |
| Changzhou028 | 17 | 13 | 19 | 26 | 17 | 10 | 24 | 13 | 10 | 10 | 14 | 18 | 25 | 25 | 12 | 13 | 10 | 12 | 18 | 13,19 | 14 | 14 |
| Changzhou029 | 18 | 12 | 19 | 28 | 13 | 11 | 22 | 12 | 12 | 10 | 15 | 17 | 20 | 23 | 12 | 12 | 9 | 12 | 18 | 12,16 | 15 | 12 |
| Changzhou030 | 19 | 14 | 19 | 30 | 15 | 10 | 23 | 12 | 11 | 10 | 15 | 16 | 20 | 23 | 12 | 12 | 10 | 12 | 20 | 13,13 | 15 | 13 |
| Changzhou031 | 20 | 14 | 18 | 30 | 15 | 10 | 23 | 11 | 11 | 10 | 14 | 18 | 20 | 24 | 12 | 13 | 11 | 14 | 15 | 11,18 | 15 | 11 |
| Changzhou032 | 19 | 12 | 19 | 29 | 16 | 10 | 22 | 12 | 12 | 10 | 15 | 18 | 19 | 23 | 11 | 12 | 11 | 12 | 20 | 12,12 | 15 | 13 |
| Changzhou033 | 18 | 12 | 19 | 29 | 15 | 10 | 25 | 12 | 11 | 10 | 14 | 19 | 19 | 23 | 11 | 14 | 11 | 13 | 15 | 13,13 | 16 | 12 |
| Changzhou034 | 19 | 13 | 21 | 29 | 14 | 11 | 23 | 13 | 10 | 11 | 15 | 19 | 21 | 25 | 11 | 13 | 12 | 12 | 17 | 13,16 | 17 | 11 |
| Changzhou035 | 20 | 12 | 20 | 28 | 14 | 10 | 21 | 11 | 11 | 11 | 15 | 18 | 20 | 25 | 13 | 14 | 12 | 12 | 17 | 13,18 | 15 | 12 |
| Changzhou036 | 19 | 12 | 20 | 28 | 14 | 10 | 23 | 13 | 11 | 11 | 14 | 19 | 20 | 23 | 12 | 14 | 11 | 12 | 18 | 13,18 | 15 | 12 |
| Changzhou037 | 18 | 14 | 19 | 29 | 13 | 6 | 23 | 12 | 12 | 13 | 14 | 21 | 22 | 24 | 11 | 14 | 10 | 14 | 16 | 16,19 | 16 | 10 |
| Changzhou038 | 14 | 14 | 21 | 29 | 16 | 11 | 24 | 13 | 12 | 10 | 14 | 17 | 21 | 23 | 12 | 12 | 9 | 14 | 17 | 10,20 | 15 | 11 |
| Changzhou039 | 20 | 12 | 19 | 29 | 16 | 11 | 22 | 13 | 11 | 10 | 15 | 17 | 20 | 23 | 12 | 12 | 12 | 14 | 19 | 12,16 | 15 | 11 |
| Changzhou040 | 17 | 14 | 18 | 31 | 15 | 10 | 23 | 12 | 11 | 10 | 14 | 18 | 22 | 24 | 11 | 13 | 11 | 14 | 14 | 12,16 | 15 | 12 |
| Changzhou041 | 17 | 14 | 21 | 30 | 15 | 10 | 26 | 12 | 12 | 10 | 14 | 16 | 21 | 22 | 12 | 11 | 8 | 14 | 16 | 11,20 | 15 | 11 |
| Changzhou042 | 19 | 12 | 19 | 28 | 14 | 10 | 22 | 13 | 11 | 10 | 15 | 19 | 19 | 23 | 12 | 12 | 11 | 12 | 18 | 12,17 | 15 | 12 |
| Changzhou043 | 20 | 14 | 19 | 30 | 13 | 10 | 24 | 12 | 12 | 12 | 14 | 20 | 22 | 24 | 11 | 15 | 11 | 14 | 16 | 15,22 | 16 | 10 |
| Changzhou044 | 18 | 12 | 19 | 28 | 16 | 10 | 22 | 13 | 11 | 10 | 15 | 17 | 20 | 23 | 13 | 12 | 10 | 12 | 17 | 12,16 | 15 | 11 |
| Changzhou045 | 20 | 12 | 20 | 28 | 17 | 10 | 23 | 13 | 11 | 10 | 14 | 19 | 22 | 25 | 11 | 13 | 10 | 12 | 20 | 14,19 | 14 | 12 |
| Changzhou046 | 18 | 12 | 21 | 29 | 16 | 10 | 23 | 13 | 11 | 10 | 15 | 19 | 22 | 23 | 12 | 13 | 13 | 12 | 16 | 12,21 | 15 | 12 |
| Changzhou047 | 17 | 13 | 19 | 30 | 15 | 11 | 22 | 14 | 11 | 10 | 14 | 17 | 21 | 23 | 11 | 12 | 12 | 13 | 17 | 15,19 | 15 | 12 |
| Changzhou048 | 18 | 12 | 20 | 28 | 15 | 10 | 23 | 12 | 11 | 10 | 15 | 19 | 21 | 24 | 10 | 13 | 11 | 12 | 18 | 13,20 | 15 | 11 |
| Changzhou049 | 18 | 12 | 20 | 27 | 16 | 10 | 23 | 13 | 12 | 11 | 15 | 19 | 20 | 24 | 12 | 14 | 11 | 12 | 18 | 14,18 | 15 | 12 |
| Changzhou050 | 20 | 12 | 19 | 30 | 15 | 10 | 22 | 12 | 11 | 10 | 15 | 16 | 20 | 23 | 13 | 12 | 12 | 12 | 20 | 12,17 | 15 | 13 |
| Changzhou051 | 18 | 12 | 20 | 28 | 14 | 10 | 23 | 13 | 11 | 11 | 15 | 17 | 20 | 23 | 12 | 14 | 11 | 12 | 17 | 15,19 | 15 | 12 |
| Changzhou052 | 17 | 14 | 18 | 30 | 15 | 10 | 21 | 12 | 13 | 10 | 14 | 20 | 20 | 23 | 10 | 14 | 11 | 13 | 16 | 10,12 | 14 | 11 |
| Changzhou053 | 18 | 13 | 19 | 30 | 15 | 10 | 27 | 11 | 12 | 10 | 14 | 21 | 21 | 25 | 11 | 10 | 9 | 12 | 23 | 16,16 | 15 | 11 |
| Changzhou054 | 18 | 14 | 19 | 31 | 13 | 9 | 24 | 13 | 12 | 12 | 14 | 19 | 22 | 24 | 11 | 14 | 10 | 13 | 16 | 16,21 | 16 | 10 |
| Changzhou055 | 18 | 12 | 19 | 28 | 15 | 10 | 23 | 12 | 12 | 10 | 14 | 18 | 23 | 25 | 12 | 13 | 10 | 12 | 18 | 12,18 | 14 | 11 |
| Changzhou056 | 19 | 14 | 18 | 31 | 15 | 10 | 24 | 12 | 11 | 10 | 14 | 19 | 21 | 25 | 11 | 13 | 11 | 14 | 15 | 12,17 | 15 | 12 |
| Changzhou057 | 18 | 12 | 18 | 28 | 15 | 11 | 25 | 12 | 11 | 10 | 14 | 18 | 19 | 23 | 11 | 14 | 11 | 13 | 15 | 13,13 | 16 | 12 |
| Changzhou058 | 17 | 12 | 20 | 29 | 16 | 10 | 24 | 12 | 12 | 10 | 15 | 16 | 22 | 23 | 11 | 13 | 12 | 13 | 18 | 13,18 | 14 | 11 |
| Changzhou059 | 18 | 13 | 19 | 29 | 16 | 10 | 24 | 12 | 11 | 10 | 13 | 21 | 20 | 25 | 11 | 13 | 11 | 12 | 20 | 12,19 | 14 | 12 |
| Changzhou060 | 19 | 12 | 0 | 29 | 15 | 10 | 26 | 15 | 12 | 9 | 15 | 14 | 21 | 24 | 11 | 13 | 12 | 12 | 15 | 12,18 | 15 | 12 |
| Changzhou061 | 20 | 12 | 20 | 29 | 15 | 11 | 21 | 13 | 11 | 11 | 15 | 19 | 22 | 23 | 13 | 12 | 11 | 12 | 21 | 12,12 | 15 | 12 |
| Changzhou062 | 14 | 13 | 19 | 29 | 14 | 10 | 22 | 11 | 12 | 10 | 14 | 18 | 22 | 23 | 10 | 14 | 10 | 13 | 15 | 11,12 | 17 | 12 |
| Changzhou063 | 19 | 12 | 18 | 28 | 16 | 10 | 22 | 11 | 11 | 10 | 16 | 17 | 23 | 23 | 11 | 12 | 12 | 13 | 19 | 11,18 | 16 | 11 |
| Changzhou064 | 18 | 11 | 20 | 27 | 15 | 10 | 24 | 13 | 11 | 10 | 15 | 21 | 20 | 24 | 12 | 13 | 12 | 13 | 16 | 13,26 | 14 | 11 |
| Changzhou065 | 19 | 14 | 19 | 30 | 16 | 10 | 24 | 12 | 12 | 10 | 14 | 18 | 22 | 24 | 12 | 13 | 10 | 12 | 17 | 13,18 | 15 | 12 |
| Changzhou066 | 18 | 12 | 19 | 27 | 15 | 10 | 23 | 12 | 11 | 10 | 14 | 17 | 23 | 25 | 11 | 13 | 10 | 12 | 18 | 12,19 | 15 | 11 |
| Changzhou067 | 20 | 13 | 19 | 28 | 17 | 10 | 23 | 14 | 11 | 10 | 15 | 18 | 20 | 22 | 12 | 14 | 10 | 12 | 17 | 11,12 | 15 | 11 |
| Changzhou068 | 17 | 13 | 18 | 30 | 15 | 11 | 25 | 13 | 11 | 10 | 14 | 18 | 19 | 23 | 11 | 14 | 11 | 13 | 15 | 13,13 | 17 | 12 |
| Changzhou069 | 17 | 12 | 18 | 29 | 15 | 11 | 24 | 12 | 11 | 10 | 14 | 19 | 19 | 23 | 11 | 14 | 9 | 13 | 15 | 13,13 | 17 | 11 |
| Changzhou070 | 17 | 12 | 20 | 30 | 15 | 10 | 24 | 13 | 11 | 10 | 16 | 19 | 21 | 25 | 11 | 13 | 11 | 12 | 16 | 13,20 | 14 | 13 |
| Changzhou071 | 20 | 12 | 19 | 27 | 16 | 10 | 24 | 12 | 11 | 10 | 14 | 17 | 21 | 23 | 10 | 14 | 13 | 12 | 16 | 13,14 | 16 | 13 |
| Changzhou072 | 19 | 13 | 19 | 30 | 17 | 10 | 25 | 12 | 11 | 10 | 14 | 19 | 22 | 24 | 12 | 13 | 10 | 12 | 20 | 12,19 | 14 | 11 |
| Changzhou073 | 18 | 12 | 19 | 28 | 15 | 10 | 25 | 12 | 11 | 10 | 14 | 18 | 19 | 23 | 12 | 14 | 11 | 13 | 15 | 13,13 | 17 | 12 |
| Changzhou074 | 17 | 12 | 18 | 28 | 15 | 11 | 25 | 12 | 11 | 10 | 14 | 18 | 19 | 23 | 11 | 14 | 11 | 13 | 17 | 13,13 | 17 | 12 |
| Changzhou075 | 17 | 13 | 19 | 30 | 16 | 10 | 22 | 12 | 11 | 10 | 15 | 16 | 20 | 24 | 11 | 12 | 11 | 12 | 17 | 11,16 | 15 | 12 |
| Changzhou076 | 19 | 12 | 19 | 28 | 17 | 10 | 24 | 13 | 11 | 10 | 14 | 17 | 22 | 25 | 12 | 13 | 10 | 12 | 16 | 12,22 | 14 | 11 |
| Changzhou077 | 20 | 14 | 19 | 30 | 15 | 10 | 23 | 13 | 11 | 10 | 15 | 16 | 20 | 24 | 14 | 12 | 12 | 12 | 15 | 12,17 | 15 | 11 |
| Changzhou078 | 18 | 13 | 18 | 29 | 15 | 10 | 23 | 12 | 11 | 11 | 14 | 19 | 22 | 25 | 12 | 13 | 12 | 13 | 16 | 13,19 | 15 | 11 |
| Changzhou079 | 18 | 12 | 20 | 28 | 15 | 11 | 24 | 13 | 11 | 11 | 15 | 18 | 20 | 24 | 13 | 14 | 11 | 12 | 18 | 13,19 | 15 | 13 |
| Changzhou080 | 19 | 12 | 18 | 27 | 15 | 11 | 25 | 13 | 11 | 10 | 14 | 20 | 19 | 23 | 12 | 14 | 11 | 13 | 15 | 13,13 | 17 | 12 |
| Changzhou081 | 18 | 14 | 20 | 31 | 15 | 10 | 23 | 12 | 11 | 10 | 15 | 17 | 22 | 24 | 13 | 13 | 11 | 12 | 18 | 14,21 | 13 | 12 |
| Changzhou082 | 20 | 13 | 19 | 28 | 17 | 10 | 24 | 12 | 11 | 10 | 14 | 18 | 23 | 25 | 12 | 13 | 10 | 12 | 17 | 13,19 | 14 | 12 |
| Changzhou083 | 18 | 14 | 18 | 30 | 14 | 11 | 21 | 12 | 12 | 10 | 15 | 18 | 21 | 23 | 10 | 14 | 11 | 13 | 17 | 11,12 | 14 | 11 |
| Changzhou084 | 17 | 12 | 20 | 28 | 15 | 10 | 24 | 13 | 12 | 11 | 15 | 17 | 20 | 24 | 12 | 13 | 11 | 12 | 17 | 13,20 | 15 | 12 |
| Changzhou085 | 18 | 12 | 20 | 28 | 14 | 10 | 25 | 12 | 11 | 10 | 15 | 19 | 21 | 25 | 11 | 13 | 11 | 12 | 19 | 13,18 | 15 | 11 |
| Changzhou086 | 18 | 12 | 20 | 27 | 16 | 10 | 24 | 14 | 11 | 9 | 14 | 18 | 22 | 25 | 12 | 13 | 10 | 12 | 18 | 13,19 | 14 | 12 |
| Changzhou087 | 19 | 14 | 20 | 30 | 14 | 10 | 24 | 12 | 10 | 10 | 15 | 20 | 21 | 24 | 11 | 13 | 12 | 12 | 16 | 13,18 | 16 | 11 |
| Changzhou088 | 18 | 13 | 20 | 29 | 16 | 10 | 25 | 13 | 11 | 10 | 11 | 17 | 23 | 24 | 13 | 13 | 10 | 12 | 17 | 12,18 | 15 | 11 |
| Changzhou089 | 19 | 12 | 18 | 28 | 16 | 11 | 25 | 12 | 12 | 10 | 14 | 19 | 19 | 24 | 11 | 14 | 11 | 13 | 15 | 13,14 | 17 | 12 |
| Changzhou090 | 18 | 12 | 20 | 28 | 15 | 10 | 25 | 12 | 12 | 11 | 15 | 18 | 19 | 24 | 11 | 14 | 11 | 12 | 19 | 13,18 | 15 | 12 |
| Changzhou091 | 18 | 12 | 20 | 28 | 14 | 10 | 24 | 12 | 11 | 11 | 15 | 18 | 20 | 24 | 11 | 14 | 13 | 12 | 17 | 13,17 | 15 | 12 |
| Changzhou092 | 20 | 12 | 19 | 29 | 15 | 11 | 21 | 12 | 11 | 10 | 14 | 16 | 20 | 23 | 12 | 12 | 11 | 12 | 19 | 12,17 | 16 | 12 |
| Changzhou093 | 18 | 12 | 19 | 27 | 15 | 10 | 23 | 13 | 11 | 10 | 15 | 19 | 19 | 23 | 11 | 12 | 11 | 13 | 19 | 11,17 | 15 | 12 |
| Changzhou094 | 17 | 14 | 20 | 32 | 15 | 10 | 23 | 12 | 11 | 10 | 15 | 17 | 21 | 25 | 12 | 13 | 11 | 12 | 17 | 15,21 | 14 | 12 |
| Changzhou095 | 18 | 12 | 20 | 28 | 14 | 11 | 25 | 12 | 11 | 11 | 15 | 19 | 21 | 24 | 11 | 15 | 11 | 12 | 17 | 13,21 | 16 | 11 |
| Changzhou096 | 19 | 12 | 18 | 28 | 17 | 11 | 23 | 12 | 11 | 10 | 14 | 18 | 21 | 25 | 11 | 13 | 10 | 12 | 18 | 12,19 | 14 | 12 |
| Changzhou097 | 17 | 14 | 19 | 30 | 15 | 10 | 23 | 11 | 10 | 10 | 14 | 19 | 20 | 22 | 11 | 15 | 10 | 13 | 18 | 11,14 | 15 | 12 |
| Changzhou098 | 19 | 12 | 18 | 27 | 16 | 11 | 21 | 13 | 11 | 10 | 14 | 18 | 20 | 23 | 12 | 14 | 11 | 13 | 15 | 12,13 | 17 | 12 |
| Changzhou099 | 18 | 13 | 21 | 29 | 15 | 10 | 27 | 12 | 12 | 10 | 14 | 17 | 21 | 24 | 11 | 11 | 8 | 15 | 15 | 11,19 | 15 | 11 |
| Changzhou100 | 16 | 12 | 18 | 28 | 15 | 10 | 24 | 14 | 11 | 10 | 14 | 20 | 21 | 23 | 11 | 14 | 11 | 13 | 15 | 12,13 | 15 | 12 |
| Changzhou101 | 17 | 13 | 20 | 32 | 15 | 10 | 26 | 13 | 11 | 11 | 15 | 20 | 20 | 23 | 14 | 13 | 12 | 13 | 16 | 13,23 | 15 | 10 |
| Changzhou102 | 21 | 13 | 19 | 30 | 15 | 10 | 23 | 12 | 11 | 10 | 14 | 16 | 21 | 23 | 11 | 13 | 11 | 12 | 17 | 19,19 | 14 | 11 |
| Changzhou103 | 19 | 14 | 19 | 31 | 15 | 10 | 23 | 11 | 12 | 10 | 14 | 19 | 21 | 22 | 12 | 14 | 10 | 13 | 18 | 11,15 | 15 | 11 |
| Changzhou104 | 18 | 12 | 18 | 27 | 16 | 10 | 24 | 12 | 11 | 10 | 14 | 18 | 24 | 25 | 11 | 13 | 10 | 12 | 19 | 14,18 | 14 | 12 |
| Changzhou105 | 19 | 11 | 18 | 27 | 17 | 10 | 24 | 12 | 11 | 10 | 14 | 18 | 22 | 25 | 12 | 13 | 10 | 12 | 18 | 12,20 | 14 | 12 |
| Changzhou106 | 18 | 11 | 21 | 27 | 16 | 10 | 25 | 12 | 13 | 10 | 14 | 16 | 21 | 23 | 11 | 11 | 9 | 14 | 16 | 11,17 | 15 | 11 |
| Changzhou107 | 19 | 14 | 19 | 30 | 15 | 10 | 29 | 12 | 11 | 10 | 15 | 17 | 21 | 24 | 13 | 13 | 11 | 12 | 18 | 13,19 | 15 | 11 |
| Changzhou108 | 20 | 12 | 19 | 28 | 14 | 10 | 22 | 13 | 11 | 11 | 15 | 18 | 19 | 23 | 12 | 12 | 10 | 12 | 18 | 12,18 | 15 | 12 |
| Changzhou109 | 18 | 12 | 18 | 30 | 15 | 11 | 24 | 12 | 12 | 10 | 14 | 18 | 20 | 23 | 11 | 14 | 11 | 13 | 14 | 13,14 | 17 | 12 |
| Changzhou110 | 18 | 12 | 19 | 28 | 14 | 11 | 22 | 12 | 11 | 10 | 15 | 18 | 19 | 23 | 11 | 12 | 11 | 12 | 18 | 12,17 | 15 | 12 |
| Changzhou111 | 18 | 14 | 22 | 29 | 16 | 9 | 26 | 11 | 11 | 10 | 15 | 19 | 20 | 25 | 14 | 13 | 12 | 14 | 20 | 12,17 | 15 | 10 |
| Changzhou112 | 18 | 13 | 20 | 29 | 13 | 6 | 24 | 12 | 12 | 12 | 14 | 20 | 22 | 24 | 11 | 14 | 10 | 14 | 14 | 16,20 | 17 | 10 |
| Changzhou113 | 18 | 12 | 20 | 27 | 14 | 10 | 23 | 13 | 11 | 11 | 16 | 17 | 21 | 24 | 12 | 14 | 11 | 12 | 17 | 15,19 | 15 | 12 |
| Changzhou114 | 16 | 13 | 19 | 28 | 16 | 10 | 23 | 13 | 11 | 10 | 15 | 17 | 20 | 21 | 12 | 14 | 10 | 12 | 18 | 11,11 | 15 | 13 |
| Changzhou115 | 20 | 12 | 20 | 27 | 13 | 10 | 22 | 12 | 11 | 11 | 15 | 16 | 19 | 23 | 12 | 12 | 11 | 12 | 17 | 13,16 | 15 | 12 |
| Changzhou116 | 18 | 12 | 20 | 29 | 15 | 10 | 24 | 13 | 11 | 10 | 15 | 19 | 21 | 25 | 12 | 13 | 11 | 12 | 16 | 13,20 | 14 | 13 |
| Changzhou117 | 17 | 12 | 19 | 28 | 16 | 10 | 22 | 14 | 11 | 10 | 15 | 16 | 20 | 23 | 11 | 12 | 11 | 12 | 19 | 13,17 | 16 | 12 |
| Changzhou118 | 15 | 13 | 21 | 29 | 16 | 10 | 23 | 13 | 12 | 9 | 15 | 18 | 21 | 23 | 12 | 11 | 10 | 13 | 15 | 13,16 | 15 | 12 |
| Changzhou119 | 16 | 12 | 20 | 28 | 14 | 10 | 24 | 12 | 10 | 11 | 15 | 20 | 20 | 24 | 12 | 13 | 12 | 12 | 18 | 14,18 | 15 | 12 |
| Changzhou120 | 16 | 13 | 22 | 30 | 15 | 10 | 25 | 12 | 12 | 10 | 14 | 17 | 21 | 23 | 13 | 11 | 9 | 15 | 16 | 11,17 | 15 | 12 |
| Changzhou121 | 18 | 13 | 19 | 29 | 15 | 10 | 23 | 13 | 12 | 10 | 14 | 22 | 22 | 22 | 11 | 15 | 10 | 13 | 16 | 11,14 | 16 | 12 |
| Changzhou122 | 22 | 12 | 20 | 28 | 13 | 11 | 24 | 11 | 13 | 11 | 14 | 21 | 20 | 24 | 12 | 14 | 10 | 12 | 18 | 13,14 | 15 | 13 |
| Changzhou123 | 19 | 12 | 20 | 28 | 15 | 10 | 23 | 12 | 11 | 11 | 14 | 18 | 20 | 24 | 12 | 14 | 11 | 13 | 17 | 14,19 | 15 | 13 |
| Changzhou124 | 17 | 12 | 19 | 28 | 15 | 10 | 23 | 12 | 11 | 10 | 14 | 16 | 19 | 23 | 13 | 12 | 14 | 12 | 16 | 11,16 | 16 | 12 |
| Changzhou125 | 18 | 12 | 18 | 29 | 16 | 10 | 25 | 12 | 11 | 10 | 14 | 17 | 22 | 25 | 11 | 13 | 10 | 12 | 19 | 12,18 | 14 | 11 |
| Changzhou126 | 18 | 12 | 21 | 28 | 16 | 10 | 23 | 13 | 11 | 10 | 15 | 20 | 22 | 23 | 13 | 14 | 13 | 12 | 18 | 12,20 | 15 | 12 |
| Changzhou127 | 19 | 12 | 19 | 27 | 15 | 10 | 22 | 13 | 11 | 10 | 15 | 18 | 20 | 23 | 11 | 12 | 10 | 12 | 19 | 12,17 | 16 | 12 |
| Changzhou128 | 19 | 12 | 20 | 29 | 14 | 11 | 23 | 11 | 11 | 11 | 15 | 19 | 21 | 23 | 12 | 14 | 11 | 12 | 18 | 13,18 | 15 | 11 |
| Changzhou129 | 18 | 12 | 18 | 29 | 16 | 11 | 25 | 11 | 11 | 10 | 14 | 18 | 19 | 23 | 11 | 14 | 11 | 13 | 15 | 13,14 | 17 | 13 |
| Changzhou130 | 17 | 14 | 19 | 31 | 15 | 10 | 28 | 11 | 12 | 10 | 14 | 19 | 21 | 25 | 11 | 10 | 9 | 12 | 22 | 16,16 | 15 | 11 |
| Changzhou131 | 17 | 12 | 18 | 28 | 15 | 10 | 22 | 13 | 11 | 10 | 15 | 16 | 20 | 24 | 11 | 12 | 11 | 12 | 19 | 12,15 | 15 | 11 |
| Changzhou132 | 17 | 12 | 18 | 28 | 15 | 11 | 23 | 13 | 11 | 10 | 14 | 19 | 21 | 23 | 12 | 14 | 11 | 13 | 14 | 13,14 | 16 | 12 |
| Changzhou133 | 19 | 12 | 19 | 27 | 16 | 10 | 24 | 14 | 11 | 10 | 13 | 19 | 22 | 25 | 13 | 15 | 10 | 12 | 17 | 13,19 | 16 | 12 |
| Changzhou134 | 16 | 12 | 20 | 31 | 16 | 10 | 24 | 13 | 11 | 10 | 14 | 16 | 22 | 24 | 11 | 13 | 10 | 12 | 19 | 14,17 | 13 | 12 |
| Changzhou135 | 19 | 12 | 19 | 29 | 15 | 10 | 21 | 12 | 11 | 10 | 14 | 17 | 19 | 23 | 12 | 12 | 11 | 12 | 18 | 12,17 | 16 | 12 |
| Changzhou136 | 18 | 12 | 19 | 28 | 16 | 10 | 23 | 14 | 12 | 10 | 14 | 18 | 21 | 25 | 12 | 13 | 10 | 12 | 18 | 14,18 | 13 | 11 |
| Changzhou137 | 17 | 13 | 19 | 28 | 14 | 11 | 23 | 12 | 12 | 11 | 14 | 20 | 22 | 24 | 11 | 16 | 11 | 13 | 16 | 11,12 | 16 | 12 |
| Changzhou138 | 18 | 14 | 19 | 29 | 15 | 10 | 23 | 13 | 11 | 10 | 15 | 19 | 22 | 21 | 11 | 14 | 11 | 12 | 18 | 11,11 | 14 | 11 |
| Changzhou139 | 18 | 12 | 19 | 28 | 15 | 10 | 23 | 13 | 11 | 10 | 14 | 17 | 19 | 23 | 13 | 12 | 12 | 12 | 17 | 12,16 | 16 | 13 |
| Changzhou140 | 19 | 12 | 18 | 27 | 15 | 10 | 21 | 12 | 11 | 10 | 14 | 18 | 20 | 23 | 12 | 14 | 11 | 13 | 15 | 12,13 | 18 | 13 |
| Changzhou141 | 18 | 12 | 20 | 28 | 14 | 10 | 23 | 13 | 11 | 11 | 15 | 20 | 20 | 25 | 11 | 14 | 11 | 12 | 17 | 14,19 | 15 | 13 |
| Changzhou142 | 18 | 14 | 21 | 30 | 15 | 10 | 26 | 13 | 13 | 10 | 14 | 17 | 21 | 23 | 12 | 11 | 8 | 14 | 15 | 11,16 | 15 | 11 |
| Changzhou143 | 16 | 12 | 20 | 28 | 14 | 10 | 25 | 12 | 11 | 11 | 14 | 18 | 20 | 24 | 13 | 14 | 10 | 12 | 17 | 13,19 | 15 | 12 |
| Changzhou144 | 18 | 12 | 20 | 28 | 15 | 10 | 21 | 13 | 11 | 11 | 15 | 17 | 24 | 23 | 12 | 12 | 12 | 12 | 18 | 11,12 | 15 | 12 |
| Changzhou145 | 18 | 12 | 18 | 29 | 15 | 11 | 25 | 12 | 11 | 10 | 14 | 18 | 19 | 23 | 11 | 14 | 11 | 13 | 15 | 13,13 | 17 | 12 |
| Changzhou146 | 16 | 12 | 20 | 28 | 14 | 10 | 23 | 12 | 13 | 11 | 15 | 17 | 20 | 23 | 12 | 14 | 11 | 12 | 17 | 15,19 | 15 | 12 |
| Changzhou147 | 19 | 12 | 18 | 30 | 16 | 10 | 26 | 12 | 12 | 10 | 14 | 15 | 20 | 23 | 11 | 13 | 11 | 13 | 17 | 12,17 | 15 | 12 |
| Changzhou148 | 18 | 12 | 19 | 28 | 15 | 10 | 22 | 12 | 11 | 10 | 14 | 16 | 19 | 23 | 13 | 12 | 11 | 12 | 17 | 12,19 | 15 | 12 |
| Changzhou149 | 20 | 12 | 19 | 27 | 16 | 10 | 24 | 13 | 11 | 10 | 13 | 18 | 22 | 25 | 13 | 15 | 10 | 12 | 19 | 12,19 | 15 | 12 |
| Changzhou150 | 17 | 12 | 20 | 28 | 14 | 10 | 24 | 13 | 11 | 11 | 15 | 19 | 20 | 24 | 12 | 14 | 11 | 12 | 18 | 13,18 | 15 | 13 |
| Changzhou151 | 17 | 14 | 19 | 30 | 15 | 11 | 22 | 12 | 12 | 11 | 14 | 21 | 22 | 24 | 12 | 15 | 10 | 13 | 16 | 11,12 | 16 | 12 |
| Changzhou152 | 18 | 12 | 19 | 29 | 15 | 10 | 23 | 12 | 12 | 10 | 15 | 18 | 20 | 24 | 11 | 13 | 12 | 13 | 18 | 12,19 | 14 | 11 |
| Changzhou153 | 18 | 12 | 20 | 27 | 15 | 10 | 22 | 15 | 11 | 11 | 15 | 18 | 20 | 25 | 12 | 14 | 11 | 12 | 18 | 13,17 | 16 | 12 |
| Changzhou154 | 18 | 12 | 18 | 30 | 15 | 10 | 26 | 12 | 11 | 10 | 14 | 17 | 19 | 23 | 11 | 14 | 11 | 13 | 15 | 13,13 | 16 | 12 |
| Changzhou155 | 16 | 12 | 20 | 29 | 15 | 10 | 24 | 13 | 12 | 10 | 15 | 14 | 21 | 24 | 12 | 13 | 11 | 12 | 17 | 12,20 | 15 | 11 |
| Changzhou156 | 16 | 13 | 20 | 28 | 15 | 10 | 24 | 12 | 12 | 10 | 14 | 22 | 21 | 24 | 11 | 15 | 10 | 13 | 16 | 11,12 | 16 | 13 |
| Changzhou157 | 18 | 12 | 19 | 28 | 15 | 10 | 21 | 12 | 11 | 10 | 14 | 16 | 20 | 23 | 12 | 12 | 11 | 12 | 17 | 12,17 | 16 | 13 |
| Changzhou158 | 17 | 12 | 19 | 28 | 17 | 10 | 25 | 13 | 11 | 10 | 14 | 18 | 21 | 24 | 12 | 13 | 11 | 12 | 18 | 12,19 | 14 | 12 |
| Changzhou159 | 18 | 14 | 18 | 29 | 15 | 11 | 23 | 11 | 13 | 10 | 14 | 18 | 21 | 23 | 10 | 14 | 11 | 14 | 17 | 12,12 | 14 | 11 |
| Changzhou160 | 16 | 12 | 20 | 27 | 16 | 10 | 24 | 13 | 11 | 10 | 14 | 20 | 22 | 25 | 12 | 13 | 10 | 13 | 18 | 14,19 | 14 | 12 |
| Changzhou161 | 18 | 12 | 20 | 28 | 14 | 10 | 23 | 12 | 11 | 11 | 15 | 19 | 20 | 25 | 13 | 14 | 12 | 12 | 17 | 14,19 | 15 | 12 |
| Changzhou162 | 17 | 12 | 18 | 29 | 15 | 11 | 26 | 12 | 11 | 10 | 14 | 18 | 19 | 23 | 12 | 14 | 11 | 13 | 17 | 13,13 | 17 | 13 |
| Changzhou163 | 18 | 14 | 18 | 30 | 15 | 10 | 23 | 12 | 11 | 10 | 15 | 20 | 21 | 24 | 13 | 13 | 11 | 15 | 15 | 12,17 | 15 | 12 |
| Changzhou164 | 20 | 13 | 19 | 29 | 17 | 10 | 23 | 13 | 11 | 10 | 15 | 17 | 22 | 22 | 12 | 13 | 10 | 12 | 18 | 11,11 | 15 | 11 |
| Changzhou165 | 16 | 13 | 17 | 30 | 14 | 10 | 28 | 14 | 11 | 10 | 14 | 19 | 21 | 24 | 10 | 11 | 10 | 13 | 17 | 14,19 | 15 | 11 |
| Changzhou166 | 18 | 12 | 19 | 30 | 15 | 11 | 24 | 12 | 11 | 10 | 14 | 17 | 20 | 23 | 11 | 13 | 11 | 13 | 16 | 13,13 | 15 | 12 |
| Changzhou167 | 20 | 13 | 20 | 29 | 14 | 10 | 23 | 13 | 11 | 11 | 15 | 20 | 22 | 25 | 11 | 13 | 13 | 13 | 17 | 14,19 | 16 | 11 |
| Changzhou168 | 19 | 13 | 20 | 30 | 17 | 10 | 25 | 13 | 11 | 10 | 14 | 19 | 23 | 25 | 11 | 13 | 10 | 12 | 20 | 14,17 | 15 | 12 |
| Changzhou169 | 18 | 12 | 20 | 29 | 14 | 10 | 23 | 12 | 11 | 11 | 15 | 18 | 20 | 24 | 12 | 14 | 11 | 12 | 17 | 13,21 | 15 | 12 |
| Changzhou170 | 19 | 12 | 20 | 28 | 14 | 10 | 22 | 12 | 11 | 11 | 14 | 18 | 20 | 24 | 13 | 14 | 11 | 12 | 17 | 13,19 | 15 | 12 |
| Changzhou171 | 18 | 12 | 20 | 29 | 14 | 10 | 23 | 12 | 11 | 11 | 15 | 18 | 20 | 23 | 13 | 14 | 11 | 12 | 18 | 13,19 | 16 | 13 |
| Changzhou172 | 19 | 12 | 19 | 28 | 15 | 10 | 23 | 12 | 11 | 10 | 15 | 17 | 19 | 23 | 12 | 12 | 11 | 12 | 18 | 12,16 | 17 | 12 |
| Changzhou173 | 21 | 12 | 21 | 28 | 14 | 10 | 23 | 13 | 11 | 11 | 15 | 18 | 20 | 24 | 12 | 14 | 11 | 12 | 18 | 13,19 | 14 | 12 |
| Changzhou174 | 17 | 13 | 19 | 27 | 15 | 10 | 27 | 11 | 12 | 10 | 14 | 21 | 20 | 23 | 11 | 10 | 9 | 12 | 21 | 15,16 | 15 | 11 |
| Changzhou175 | 20 | 12 | 21 | 28 | 14 | 10 | 23 | 12 | 10 | 11 | 15 | 18 | 18 | 24 | 13 | 14 | 11 | 12 | 18 | 14,19 | 15 | 12 |
| Changzhou176 | 21 | 12 | 20 | 27 | 14 | 10 | 23 | 12 | 12 | 11 | 15 | 20 | 21 | 24 | 12 | 10 | 11 | 12 | 17 | 13,19 | 15 | 11 |
| Changzhou177 | 17 | 13 | 21 | 29 | 16 | 10 | 23 | 13 | 14 | 10 | 14 | 16 | 21 | 23 | 12 | 11 | 9 | 15 | 16 | **11,16.2** | 15 | 11 |
| Changzhou178 | 18 | 12 | 19 | 28 | 15 | 10 | 25 | 13 | 11 | 10 | 14 | 18 | 20 | 23 | 11 | 14 | 11 | 13 | 15 | 13,14 | 16 | 13 |
| Changzhou179 | 16 | 13 | 20 | 30 | 15 | 10 | 26 | 12 | 12 | 11 | 14 | 16 | 21 | 23 | 11 | 11 | 9 | 14 | 16 | 11,18 | 15 | 12 |
| Changzhou180 | 19 | 14 | 19 | 30 | 14 | 10 | 24 | 11 | 12 | 10 | 14 | 19 | 21 | 22 | 12 | 14 | 10 | 13 | 17 | 11,14 | 15 | 12 |
| Changzhou181 | 18 | 14 | 18 | 30 | 14 | 11 | 21 | 12 | 13 | 10 | 14 | 19 | 21 | 23 | 10 | 14 | 11 | 12 | 17 | 11,12 | 14 | 11 |
| Changzhou182 | 18 | 13 | 18 | 29 | 14 | 9 | 24 | 12 | 10 | 10 | 14 | 19 | 21 | 23 | 11 | 15 | 10 | 14 | 16 | 12,18 | 15 | 12 |
| Changzhou183 | 18 | 13 | 20 | 29 | 14 | 10 | 23 | 12 | 11 | 11 | 14 | 17 | 20 | 25 | 12 | 15 | 11 | 12 | 21 | 13,20 | 16 | 11 |
| Changzhou184 | 18 | 12 | 19 | 27 | 16 | 10 | 24 | 15 | 11 | 10 | 13 | 19 | 21 | 25 | 12 | 15 | 11 | 13 | 19 | 12,21 | 15 | 12 |
| Changzhou185 | 18 | 12 | 18 | 29 | 15 | 11 | 25 | 13 | 11 | 10 | 14 | 18 | 19 | 23 | 11 | 14 | 11 | 13 | 15 | 13,13 | 16 | 12 |
| Changzhou186 | 18 | 12 | 18 | 28 | 17 | 10 | 24 | 11 | 11 | 10 | 14 | 18 | 21 | 25 | 11 | 13 | 11 | 12 | 19 | 12,18 | 14 | 11 |
| Changzhou187 | 19 | 13 | 19 | 28 | 17 | 10 | 24 | 12 | 11 | 10 | 14 | 18 | 23 | 25 | 13 | 13 | 10 | 12 | 18 | 13,19 | 14 | 13 |
| Changzhou188 | 18 | 12 | 19 | 28 | 13 | 10 | 22 | 13 | 12 | 10 | 15 | 18 | 21 | 26 | 11 | 12 | 9 | 13 | 17 | 12,12 | 14 | 12 |
| Changzhou189 | 20 | 14 | 19 | 31 | 15 | 10 | 22 | 12 | 11 | 10 | 15 | 17 | 19 | 23 | 12 | 13 | 11 | 12 | 17 | 11,16 | 16 | 12 |
| Changzhou190 | 20 | 12 | 19 | 28 | 15 | 10 | 21 | 12 | 11 | 10 | 14 | 16 | 19 | 23 | 12 | 13 | 11 | 12 | 18 | 12,17 | 16 | 12 |
| Changzhou191 | 20 | 13 | 19 | 31 | 16 | 10 | 23 | 11 | 12 | 10 | 14 | 17 | 21 | 23 | 11 | 13 | 10 | 12 | 16 | 10,19 | 15 | 12 |
| Changzhou192 | 18 | 12 | 20 | 28 | 15 | 10 | 23 | 13 | 11 | 10 | 14 | 19 | 24 | 25 | 11 | 13 | 9 | 12 | 16 | 14,18 | 14 | 13 |
| Changzhou193 | 17 | 12 | 21 | 28 | 15 | 7 | 25 | 12 | 11 | 10 | 15 | 19 | 21 | 26 | 12 | 13 | 11 | 12 | 16 | 13,19 | 15 | 12 |
| Changzhou194 | 20 | 12 | 19 | 28 | 16 | 10 | 26 | 13 | 12 | 10 | 14 | 17 | 21 | 25 | 11 | 13 | 10 | 12 | 16 | 12,20 | 14 | 13 |
| Changzhou195 | 18 | 12 | 19 | 27 | 15 | 10 | 25 | 12 | 12 | 10 | 15 | 16 | 23 | 24 | 13 | 13 | 12 | 13 | 17 | 13,18 | 15 | 12 |
| Changzhou196 | 17 | 12 | 20 | 28 | 14 | 10 | 22 | 13 | 11 | 11 | 14 | 19 | 20 | 23 | 11 | 14 | 11 | 12 | 17 | 14,18 | 14 | 12 |
| Changzhou197 | 17 | 12 | 19 | 28 | 16 | 10 | 25 | 12 | 11 | 10 | 14 | 18 | 21 | 25 | 13 | 13 | 10 | 12 | 17 | 12,20 | 14 | 12 |
| Changzhou198 | 18 | 14 | 19 | 30 | 14 | 10 | 22 | 12 | 12 | 10 | 14 | 22 | 21 | 22 | 9 | 14 | 9 | 15 | **16.1** | 10,12 | 15 | 10 |
| Changzhou199 | 18 | 12 | 18 | 30 | 15 | 10 | 23 | 11 | 10 | 10 | 14 | 17 | 23 | 24 | 11 | 13 | 12 | 13 | 17 | 14,18 | 15 | 10 |
| Changzhou200 | 19 | 12 | 20 | 30 | 15 | 9 | 26 | 12 | 14 | 10 | 15 | 14 | 22 | 24 | 10 | 13 | 12 | 12 | 15 | 12,17 | 15 | 12 |
| Changzhou201 | 17 | 14 | 21 | 30 | 17 | 10 | 24 | 12 | 11 | 10 | 15 | 17 | 21 | 24 | 12 | 13 | 11 | 12 | 16 | 14,20 | 13 | 12 |
| Changzhou202 | 17 | 13 | 21 | 29 | 15 | 10 | 26 | 13 | 12 | 10 | 14 | 17 | 22 | 23 | 11 | 11 | 9 | 15 | 17 | 11,16 | 15 | 11 |
| Changzhou203 | 17 | 12 | 18 | 28 | 15 | 11 | 25 | 12 | 11 | 10 | 14 | 18 | 19 | 23 | 11 | 14 | 11 | 13 | 15 | 13,13 | 17 | 12 |
| Changzhou204 | 19 | 12 | 20 | 28 | 14 | 10 | 24 | 12 | 11 | 11 | 15 | 17 | 20 | 24 | 12 | 14 | 12 | 12 | 17 | 13,18 | 16 | 13 |
| Changzhou205 | 18 | 12 | 19 | 28 | 15 | 10 | 22 | 14 | 12 | 10 | 15 | 17 | 20 | 23 | 12 | 12 | 11 | 12 | 17 | 12,18 | 15 | 12 |
| Changzhou206 | 18 | 14 | 20 | 31 | 16 | 10 | 24 | 12 | 12 | 10 | 14 | 16 | 20 | 23 | 12 | 11 | 9 | 15 | 16 | 11,18 | 16 | 11 |
| Changzhou207 | 17 | 12 | 20 | 27 | 14 | 10 | 23 | 13 | 9 | 11 | 15 | 19 | 21 | 24 | 12 | 15 | 11 | 12 | 17 | 13,15 | 15 | 12 |
|  |  |  |  |  |  |  |  |  |  |  |  |  |  |  |  |  |  |  |  |  |  |  |
| Xuzhou001 | 19 | 12 | 19 | 29 | 15 | 10 | 21 | 12 | 11 | 10 | 14 | 16 | 19 | 23 | 12 | 12 | 11 | 12 | 18 | 12,17 | 15 | 12 |
| Xuzhou002 | 17 | 12 | 20 | 29 | 15 | 10 | 26 | 12 | 11 | 11 | 15 | 17 | 21 | 24 | 11 | 13 | 11 | 12 | 16 | 12,18 | 15 | 12 |
| Xuzhou003 | 21 | 12 | 19 | 28 | 15 | 10 | 22 | 12 | 11 | 10 | 15 | 18 | 19 | 23 | 12 | 11 | 12 | 12 | 17 | 12,17 | 16 | 12 |
| Xuzhou004 | 18 | 14 | 19 | 31 | 14 | 10 | 22 | 12 | 13 | 11 | 14 | 18 | 22 | 23 | 11 | 14 | 10 | 13 | 17 | 11,12 | 16 | 12 |
| Xuzhou005 | 18 | 12 | 19 | 29 | 16 | 10 | 28 | 12 | 11 | 10 | 15 | 17 | 21 | 24 | 12 | 13 | 11 | 12 | 18 | 13,17 | 15 | 12 |
| Xuzhou006 | 17 | 12 | 19 | 29 | 16 | 11 | 25 | 13 | 12 | 10 | 16 | 17 | 22 | 24 | 11 | 12 | 11 | 12 | 15 | 13,20 | 15 | 12 |
| Xuzhou007 | 19 | 13 | 20 | 31 | 16 | 10 | 26 | 13 | 12 | 10 | 14 | 20 | 23 | 25 | 11 | 13 | 12 | 12 | 18 | 15,19 | 15 | 11 |
| Xuzhou008 | 19 | 12 | 18 | 30 | 15 | 11 | 25 | 13 | 11 | 10 | 14 | 19 | 19 | 23 | 11 | 14 | 11 | 13 | 16 | 13,13 | 16 | 12 |
| Xuzhou009 | 18 | 12 | 19 | 29 | 16 | 10 | 29 | 11 | 12 | 11 | 14 | 19 | 21 | 24 | 11 | 13 | 11 | 13 | 19 | 14,19 | 15 | 11 |
| Xuzhou010 | 18 | 14 | 18 | 29 | 15 | 10 | 23 | 12 | 10 | 10 | 14 | 19 | 21 | 25 | 11 | 13 | 11 | 14 | 16 | 12,17 | 14 | 12 |
| Xuzhou011 | 19 | 12 | 20 | 29 | 13 | 10 | 23 | 13 | 11 | 10 | 15 | 17 | 22 | 25 | 12 | 14 | 9 | 13 | 18 | 11,12 | 15 | 12 |
| Xuzhou012 | 16 | 14 | 18 | 30 | 14 | 11 | 21 | 12 | 13 | 10 | 14 | 19 | 20 | 23 | 11 | 14 | 11 | 12 | 19 | 11,12 | 14 | 11 |
| Xuzhou013 | 18 | 13 | 20 | 28 | 17 | 10 | 24 | 13 | 11 | 10 | 14 | 18 | 24 | 24 | 13 | 13 | 10 | 12 | 18 | 12,19 | 14 | 12 |
| Xuzhou014 | 17 | 14 | 20 | 29 | 16 | 11 | 24 | 12 | 12 | 10 | 14 | 16 | 21 | 23 | 13 | 11 | 9 | 14 | 17 | 11,19 | 15 | 10 |
| Xuzhou015 | 18 | 12 | 19 | 29 | 16 | 10 | 26 | 11 | 12 | 11 | 14 | 19 | 21 | 24 | 11 | 13 | 11 | 13 | 19 | 14,19 | 15 | 11 |
| Xuzhou016 | 17 | 13 | 21 | 29 | 17 | 10 | 24 | 13 | 14 | 10 | 14 | 17 | 23 | 20 | 11 | 11 | 9 | 15 | 16 | 10,17 | 15 | 11 |
| Xuzhou017 | 18 | 12 | 20 | 28 | 17 | 10 | 25 | 12 | 11 | 10 | 14 | 17 | 23 | 24 | 11 | 13 | 10 | 12 | 18 | 14,16 | 14 | 12 |
| Xuzhou018 | 21 | 12 | 20 | 26 | 14 | 10 | 23 | 15 | 11 | 11 | 15 | 18 | 20 | 24 | 13 | 14 | 11 | 12 | 20 | 13,20 | 15 | 12 |
| Xuzhou019 | 18 | 12 | 19 | 30 | 15 | 10 | 22 | 11 | 11 | 10 | 15 | 16 | 20 | 23 | 12 | 12 | 11 | 12 | 18 | 12,13 | 15 | 12 |
| Xuzhou020 | 17 | 12 | **19.2** | 28 | 15 | 11 | 24 | 12 | 11 | 10 | 14 | 22 | 23 | 25 | 12 | 13 | 10 | 12 | 17 | 12,12 | 14 | 13 |
| Xuzhou021 | 16 | 13 | 18 | 28 | 15 | 11 | 26 | 13 | 12 | 11 | 14 | 17 | 21 | 23 | 12 | 13 | 11 | 14 | 15 | 12,18 | 16 | 12 |
| Xuzhou022 | 18 | 12 | 21 | 28 | 15 | 10 | 23 | 11 | 11 | 11 | 15 | 19 | 21 | 24 | 11 | 14 | 12 | 12 | 18 | 13,19 | 15 | 11 |
| Xuzhou023 | 16 | 12 | 20 | 29 | 15 | 10 | 26 | 12 | 11 | 12 | 15 | 19 | 22 | 24 | 10 | 13 | 11 | 12 | 15 | 12,18 | 15 | 12 |
| Xuzhou024 | 19 | 12 | 20 | 28 | 15 | 10 | 23 | 12 | 11 | 11 | 15 | 18 | 20 | 24 | 13 | 14 | 11 | 13 | 16 | 13,17 | 15 | 12 |
| Xuzhou025 | 19 | 13 | 18 | 28 | 14 | 10 | 24 | 13 | 10 | 10 | 14 | 19 | 23 | 23 | 13 | 13 | 11 | 14 | 18 | 12,20 | 15 | 11 |
| Xuzhou026 | 20 | 13 | 19 | 29 | 14 | 10 | 21 | 12 | 12 | 9 | 14 | 17 | 20 | 22 | 10 | 14 | 9 | 14 | 16 | 10,13 | 18 | 12 |
| Xuzhou027 | 18 | 12 | 21 | 29 | 14 | 10 | 23 | 13 | 11 | 11 | 15 | 20 | 20 | 24 | 11 | 14 | 11 | 12 | 18 | 14,19 | 15 | 13 |
| Xuzhou028 | 18 | 11 | 19 | 27 | 15 | 10 | 22 | 12 | 11 | 10 | 15 | 17 | 19 | 23 | 12 | 12 | 11 | 12 | 20 | 12,18 | 15 | 12 |
| Xuzhou029 | 19 | 12 | 19 | 28 | 15 | 10 | 21 | 12 | 11 | 10 | 15 | 18 | 19 | 23 | 13 | 12 | 12 | 12 | 18 | 12,13 | 15 | 13 |
| Xuzhou030 | 20 | 12 | 20 | 28 | 14 | 10 | 23 | 12 | 11 | 11 | 15 | 19 | 20 | 24 | 11 | 14 | 10 | 12 | 18 | 15,16 | 15 | 11 |
| Xuzhou031 | 15 | 12 | 20 | 28 | 16 | 10 | 25 | 12 | 11 | 10 | 14 | 21 | 23 | 25 | 11 | 13 | 10 | 12 | 17 | 14,18 | 15 | 12 |
| Xuzhou032 | 19 | 12 | 18 | 29 | 15 | 11 | 25 | 12 | 11 | 10 | 14 | 18 | 20 | 22 | 11 | 14 | 11 | 13 | 15 | 13,13 | 15 | 13 |
| Xuzhou033 | 18 | 14 | 18 | 30 | 14 | 10 | 23 | 12 | 10 | 10 | 14 | 20 | 23 | 24 | 10 | 13 | 11 | 14 | 15 | 12,18 | 15 | 12 |
| Xuzhou034 | 19 | 12 | 20 | 29 | 14 | 10 | 23 | 12 | 11 | 11 | 15 | 18 | 20 | 24 | 12 | 14 | 11 | 12 | 17 | 13,19 | 15 | 13 |
| Xuzhou035 | 18 | 12 | 20 | 28 | 14 | 10 | 23 | 13 | 11 | 11 | 14 | 18 | 20 | 23 | 12 | 14 | 12 | 12 | 17 | 13,19 | 15 | 12 |
| Xuzhou036 | 19 | 12 | 20 | 27 | 14 | 10 | 24 | 13 | 11 | 11 | 15 | 18 | 21 | 24 | 13 | 12 | 11 | 12 | 17 | 13,18 | 15 | 12 |
| Xuzhou037 | 20 | 12 | 19 | 29 | 15 | 10 | 21 | 12 | 11 | 10 | 14 | 16 | 19 | 24 | 13 | 12 | 11 | 12 | 20 | 13,15 | 16 | 12 |
| Xuzhou038 | 19 | 13 | 18 | 30 | 13 | 10 | 26 | 11 | 12 | 10 | 14 | 19 | 21 | 24 | 14 | 11 | 11 | 14 | 20 | 13,15 | 16 | 12 |
| Xuzhou039 | 18 | 13 | 20 | 28 | 15 | 10 | 23 | 12 | 11 | 10 | 15 | 17 | 21 | 22 | 13 | 13 | 11 | 12 | 21 | 11,12 | 15 | 11 |
| Xuzhou040 | 18 | 13 | 18 | 31 | 15 | 10 | 23 | 13 | 10 | 10 | 14 | 20 | 24 | 24 | 13 | 13 | 11 | 14 | 19 | 12,20 | 15 | 11 |
| Xuzhou041 | 17 | 13 | 20 | 31 | 16 | 11 | 24 | 13 | 12 | 11 | 14 | 19 | 23 | 24 | 10 | 11 | 10 | 13 | 15 | 11,15 | 15 | 13 |
| Xuzhou042 | 19 | 12 | 19 | 28 | 13 | 10 | 22 | 13 | 11 | 10 | 15 | 17 | 19 | 23 | 12 | 12 | 11 | 13 | 20 | 12,12 | 15 | 11 |
| Xuzhou043 | 15 | 12 | 20 | 28 | 15 | 10 | 24 | 12 | 11 | 10 | 14 | 19 | 22 | 24 | 11 | 13 | 10 | 12 | 17 | 14,19 | 14 | 12 |
| Xuzhou044 | 17 | 12 | 20 | 28 | 14 | 11 | 23 | 12 | 12 | 11 | 15 | 18 | 20 | 24 | 12 | 14 | 11 | 12 | 17 | 12,17 | 16 | 12 |
| Xuzhou045 | 19 | 12 | 20 | 28 | 14 | 10 | 22 | 13 | 11 | 11 | 16 | 19 | 21 | 25 | 12 | 14 | 11 | 13 | 17 | 13,18 | 15 | 12 |
| Xuzhou046 | 19 | 14 | 18 | 31 | 15 | 10 | 23 | 12 | 11 | 10 | 14 | 18 | 21 | 24 | 11 | 13 | 11 | 14 | 15 | 12,18 | 15 | 12 |
| Xuzhou047 | 19 | 13 | 20 | 29 | 16 | 10 | 24 | 13 | 12 | 10 | 14 | 16 | 21 | 23 | 11 | 11 | 9 | 15 | 16 | 11,11 | 15 | 11 |
| Xuzhou048 | 19 | 13 | 20 | 30 | 16 | 9 | 27 | 11 | 11 | 10 | 14 | 17 | 24 | 24 | 11 | 11 | 10 | 13 | 17 | 12,12 | 15 | 10 |
| Xuzhou049 | 19 | 13 | 19 | 28 | 17 | 10 | 24 | 12 | 12 | 10 | 14 | 18 | 24 | 25 | 12 | 13 | 10 | 12 | 18 | 13,19 | 14 | 12 |
| Xuzhou050 | 17 | 14 | 19 | 31 | 14 | 10 | 22 | 12 | 12 | 10 | 14 | 19 | 23 | 23 | 13 | 14 | 10 | 13 | 14 | 11,13 | 17 | 12 |
| Xuzhou051 | 21 | 12 | 20 | 27 | 13 | 10 | 23 | 12 | 11 | 11 | 15 | 18 | 20 | 24 | 13 | 14 | 11 | 12 | 18 | 14,21 | 15 | 12 |
| Xuzhou052 | 19 | 12 | 20 | 29 | 16 | 10 | 25 | 11 | 12 | 10 | 14 | 19 | 23 | 25 | 11 | 13 | 10 | 12 | 22 | 15,18 | 14 | 12 |
| Xuzhou053 | 18 | 12 | 19 | 29 | 15 | 10 | 22 | 13 | 11 | 10 | 15 | 17 | 19 | 23 | 12 | 12 | 11 | 12 | 17 | 12,18 | 15 | 12 |
| Xuzhou054 | 18 | 12 | 18 | 27 | 15 | 10 | 22 | 13 | 11 | 10 | 15 | 16 | 20 | 23 | 11 | 12 | 10 | 12 | 19 | 13,17 | 15 | 12 |
| Xuzhou055 | 17 | 15 | 19 | 33 | 17 | 10 | 22 | 12 | 11 | 10 | 15 | 19 | 21 | 25 | 12 | 13 | 11 | 12 | 19 | 11,18 | 15 | 10 |
| Xuzhou056 | 16 | 11 | 20 | 27 | 17 | 10 | 24 | 14 | 12 | 10 | 14 | 20 | 24 | 24 | 12 | 13 | 10 | 14 | 22 | 14,17 | 13 | 12 |
| Xuzhou057 | 19 | 13 | 19 | 29 | 15 | 10 | 25 | 14 | 12 | 10 | 15 | 16 | 21 | 24 | 12 | 13 | 11 | 12 | 17 | 11,18 | 17 | 11 |
| Xuzhou058 | 21 | 12 | 18 | 28 | 15 | 12 | 23 | 13 | 11 | 10 | 14 | 15 | 20 | 23 | 12 | 13 | 11 | 13 | 18 | 13,13 | 15 | 12 |
| Xuzhou059 | 19 | 14 | 21 | 31 | 16 | 10 | 23 | 12 | 12 | 10 | 15 | 20 | 22 | 24 | 13 | 13 | 11 | 12 | 16 | 12,19 | 13 | 11 |
| Xuzhou060 | 18 | 12 | 19 | 28 | 15 | 10 | 22 | 12 | 11 | 11 | 15 | 16 | 20 | 23 | 11 | 12 | 11 | 12 | 17 | 12,16 | 15 | 12 |
| Xuzhou061 | 18 | 12 | 18 | 27 | 15 | 10 | 22 | 13 | 11 | 10 | 15 | 16 | 20 | 23 | 11 | 12 | 10 | 12 | 20 | 13,17 | 15 | 12 |
| Xuzhou062 | 19 | 12 | 19 | 29 | 15 | 10 | 26 | 13 | 11 | 10 | 14 | 17 | 20 | 24 | 12 | 13 | 10 | 12 | 17 | 12,19 | 14 | 13 |
| Xuzhou063 | 19 | 12 | 19 | 28 | 15 | 10 | 21 | 12 | 11 | 10 | 14 | 16 | 19 | 23 | 11 | 12 | 11 | 12 | 18 | 12,17 | 16 | 11 |
| Xuzhou064 | 19 | 13 | 19 | 30 | 16 | 10 | 23 | 12 | 11 | 10 | 14 | 16 | 21 | 24 | 11 | 13 | 11 | 13 | 17 | 12,19 | 15 | 11 |
| Xuzhou065 | 18 | 14 | 22 | 30 | 16 | 10 | 24 | 13 | 12 | 10 | 14 | 16 | 23 | 23 | 12 | 11 | 9 | 14 | 18 | 11,17 | 16 | 11 |
| Xuzhou066 | 19 | 12 | 18 | 27 | 15 | 11 | 22 | 13 | 11 | 10 | 15 | 16 | 20 | 23 | 11 | 12 | 10 | 12 | 20 | 13,17 | 15 | 12 |
| Xuzhou067 | 17 | 12 | 20 | 31 | 16 | 10 | 24 | 13 | 11 | 10 | 14 | 18 | 23 | 24 | 11 | 13 | 10 | 12 | 19 | 14,18 | 14 | 12 |
| Xuzhou068 | 19 | 12 | 21 | 28 | 15 | 10 | 23 | 12 | 10 | 11 | 16 | 18 | 21 | 25 | 12 | 14 | 12 | 14 | 17 | 13,13 | 15 | 12 |
| Xuzhou069 | 19 | 13 | 19 | 28 | 15 | 10 | 23 | 13 | 11 | 10 | 15 | 19 | 21 | 24 | 13 | 13 | 11 | 12 | 18 | 11,11 | 16 | 12 |
| Xuzhou070 | 18 | 13 | 21 | 30 | 16 | 10 | 24 | 12 | 14 | 10 | 14 | 15 | 21 | 23 | 12 | 11 | 9 | 15 | 17 | 11,17 | 15 | 11 |
| Xuzhou071 | 20 | 12 | 19 | 28 | 17 | 10 | 24 | 13 | 11 | 10 | 14 | 17 | 22 | 24 | 13 | 13 | 11 | 13 | 19 | 11,21 | 14 | 12 |
| Xuzhou072 | 20 | 12 | 20 | 28 | 14 | 10 | 24 | 12 | 13 | 11 | 15 | 19 | 21 | 24 | 12 | 14 | 11 | 12 | 17 | 12,18 | 15 | 11 |
| Xuzhou073 | 21 | 12 | 19 | 27 | 17 | 10 | 24 | 12 | 11 | 10 | 14 | 18 | 24 | 25 | 13 | 13 | 11 | 12 | 22 | 13,20 | 14 | 13 |
| Xuzhou074 | 17 | 12 | 19 | 28 | 15 | 10 | 26 | 14 | 13 | 9 | 15 | 14 | 21 | 24 | 12 | 13 | 11 | 12 | 15 | 12,21 | 15 | 13 |
| Xuzhou075 | 19 | 12 | 19 | 28 | 15 | 10 | 23 | 12 | 11 | 10 | 14 | 18 | 24 | 24 | 11 | 13 | 10 | 12 | 21 | 12,20 | 15 | 11 |
| Xuzhou076 | 20 | 12 | 19 | 30 | 15 | 10 | 21 | 13 | 11 | 10 | 14 | 16 | 19 | 24 | 12 | 12 | 11 | 12 | 19 | 12,18 | 15 | 12 |
| Xuzhou077 | 22 | 12 | 20 | 28 | 14 | 10 | 23 | 13 | 12 | 11 | 15 | 19 | 22 | 24 | 12 | 15 | 11 | 13 | 17 | 14,20 | 15 | 12 |
| Xuzhou078 | 19 | 12 | 19 | 28 | 15 | 10 | 21 | 12 | 11 | 10 | 14 | 16 | 19 | 23 | 14 | 12 | 11 | 12 | 18 | 12,17 | 16 | 11 |
| Xuzhou079 | 18 | 12 | 20 | 28 | 14 | 10 | 23 | 13 | 11 | 11 | 14 | 19 | 20 | 23 | 11 | 14 | 11 | 12 | 17 | 13,19 | 15 | 12 |
| Xuzhou080 | 16 | 12 | 20 | 29 | 15 | 10 | 25 | 13 | 12 | 11 | 15 | 16 | 21 | 24 | 10 | 13 | 11 | 12 | 17 | 12,18 | 14 | 12 |
| Xuzhou081 | 19 | 12 | 19 | 29 | 15 | 10 | 22 | 12 | 11 | 10 | 15 | 16 | 19 | 23 | 12 | 12 | 11 | 12 | 16 | 12,16 | 16 | 12 |
| Xuzhou082 | 18 | 12 | 19 | 28 | 15 | 10 | 22 | 12 | 11 | 10 | 15 | 16 | 19 | 23 | 11 | 12 | 10 | 12 | 17 | 12,13 | 15 | 12 |
| Xuzhou083 | 18 | 12 | 19 | 28 | 15 | 11 | 21 | 12 | 11 | 10 | 14 | 18 | 21 | 23 | 13 | 12 | 11 | 12 | 17 | 12,16 | 16 | 12 |
| Xuzhou084 | 17 | 12 | 20 | 29 | 14 | 10 | 23 | 12 | 11 | 11 | 15 | 18 | 20 | 24 | 12 | 14 | 12 | 12 | 16 | 13,18 | 15 | 12 |
| Xuzhou085 | 18 | 14 | 21 | 30 | 15 | 10 | 24 | 12 | 12 | 10 | 15 | 20 | 21 | 23 | 11 | 13 | 12 | 13 | 20 | 13,19 | 15 | 12 |
| Xuzhou086 | 19 | 12 | 20 | 28 | 14 | 10 | 25 | 12 | 11 | 11 | 16 | 18 | 20 | 24 | 12 | 14 | 11 | 12 | 17 | 13,18 | 15 | 12 |
| Xuzhou087 | 19 | 12 | 18 | 28 | 14 | 11 | 25 | 12 | 11 | 10 | 14 | 18 | 20 | 24 | 11 | 14 | 11 | 13 | 15 | 13,13 | 16 | 12 |
| Xuzhou088 | 17 | 13 | 18 | 30 | 15 | 10 | 23 | 13 | 11 | 10 | 14 | 20 | 21 | 24 | 11 | 13 | 11 | 14 | 15 | 12,17 | 15 | 11 |
| Xuzhou089 | 17 | 13 | 18 | 28 | 15 | 10 | 26 | 13 | 11 | 11 | 14 | 16 | 21 | 23 | 13 | 13 | 11 | 13 | 16 | 12,17 | 15 | 12 |
| Xuzhou090 | 18 | 12 | 19 | 28 | 14 | 10 | 23 | 13 | 11 | 11 | 15 | 18 | 21 | 25 | 12 | 14 | 11 | 12 | 17 | 13,20 | 15 | 13 |
| Xuzhou091 | 17 | 14 | 21 | 31 | 16 | 10 | 24 | 12 | 14 | 10 | 14 | 16 | 21 | 23 | 11 | 11 | 8 | 15 | 17 | 11,17 | 14 | 11 |
| Xuzhou092 | 18 | 12 | 20 | 28 | 14 | 10 | 24 | 13 | 11 | 11 | 14 | 19 | 20 | 23 | 12 | 14 | 11 | 12 | 17 | 13,19 | 15 | 11 |
| Xuzhou093 | 20 | 12 | 20 | 28 | 14 | 10 | 23 | 12 | 11 | 11 | 14 | 19 | 20 | 23 | 11 | 14 | 11 | 12 | 18 | 13,19 | 15 | 12 |
| Xuzhou094 | 21 | 12 | 19 | 29 | 16 | 8 | 24 | 11 | 11 | 10 | 14 | 16 | 23 | 25 | 12 | 13 | 10 | 12 | 19 | 12,20 | 14 | 11 |
| Xuzhou095 | 17 | 12 | 20 | 27 | 15 | 10 | 24 | 13 | 11 | 11 | 15 | 18 | 21 | 24 | 12 | 14 | 11 | 13 | 17 | 13,18 | 15 | 13 |
| Xuzhou096 | 19 | 12 | 19 | 29 | 15 | 10 | 21 | 12 | 11 | 10 | 14 | 17 | 19 | 23 | 13 | 12 | 10 | 12 | 18 | 12,17 | 15 | 12 |
| Xuzhou097 | 15 | 13 | 22 | 29 | 14 | 10 | 23 | 12 | 13 | 9 | 15 | 17 | 21 | 23 | 11 | 11 | 10 | 12 | 16 | 13,17 | 14 | 12 |
| Xuzhou098 | 18 | 12 | 20 | 28 | 14 | 11 | 23 | 13 | 11 | 11 | 14 | 19 | 20 | 24 | 12 | 14 | 11 | 12 | 17 | 12,19 | 15 | 12 |
| Xuzhou099 | 18 | 14 | 18 | 31 | 15 | 10 | 23 | 12 | 11 | 10 | 14 | 19 | 22 | 24 | 12 | 13 | 11 | 14 | 16 | 12,17 | 15 | 12 |
| Xuzhou100 | 17 | 11 | 19 | 28 | 15 | 11 | 24 | 12 | 12 | 10 | 15 | 15 | 22 | 24 | 11 | 13 | 11 | 13 | 20 | 12,17 | 14 | 11 |
| Xuzhou101 | 19 | 12 | 19 | 28 | 15 | 10 | 26 | 13 | 12 | 10 | 14 | 19 | 22 | 25 | 12 | 13 | 10 | 13 | 19 | 12,18 | 15 | 12 |
| Xuzhou102 | 18 | 12 | 20 | 27 | 17 | 10 | 25 | 13 | 11 | 10 | 14 | 19 | 21 | 25 | 12 | 13 | 10 | 12 | 18 | 12,18 | 14 | 12 |
| Xuzhou103 | 18 | 12 | 20 | 28 | 14 | 10 | 23 | 13 | 11 | 11 | 14 | 19 | 20 | 23 | 11 | 14 | 11 | 12 | 17 | 13,19 | 14 | 11 |
| Xuzhou104 | 17 | 12 | 20 | 29 | 15 | 11 | 24 | 13 | 12 | 10 | 14 | 18 | 21 | 24 | 11 | 13 | 10 | 12 | 18 | 14,19 | 14 | 12 |
| Xuzhou105 | 19 | 12 | 19 | 28 | 15 | 10 | 22 | 12 | 11 | 10 | 15 | 17 | 19 | 23 | 13 | 12 | 12 | 12 | 18 | 11,16 | 15 | 12 |
| Xuzhou106 | 20 | 14 | 21 | 30 | 15 | 10 | 25 | 12 | 13 | 10 | 14 | 16 | 21 | 22 | 11 | 11 | 8 | 15 | 13 | 11,21 | 14 | 11 |
| Xuzhou107 | 18 | 13 | 21 | 29 | 15 | 10 | 25 | 12 | 12 | 10 | 14 | 17 | 23 | 23 | 12 | 11 | 9 | 14 | 17 | 11,18 | 15 | 11 |
| Xuzhou108 | 21 | 12 | 20 | 27 | 14 | 10 | 23 | 12 | 12 | 11 | 15 | 20 | 21 | 24 | 12 | 10 | 11 | 12 | 17 | 13,19 | 15 | 11 |
| Xuzhou109 | 19 | 12 | 18 | 28 | 15 | 10 | 23 | 11 | 11 | 10 | 15 | 15 | 22 | 23 | 12 | 13 | 11 | 12 | 19 | 13,17 | 17 | 13 |
| Xuzhou110 | 19 | 13 | 20 | 30 | 15 | 9 | 26 | 11 | 12 | 10 | 14 | 18 | 21 | 23 | 13 | 14 | 11 | 13 | 16 | 13,17 | 19 | 12 |
| Xuzhou111 | 19 | 12 | 19 | 27 | 15 | 10 | 22 | 12 | 11 | 10 | 15 | 16 | 19 | 23 | 12 | 12 | 11 | 12 | 17 | 13,16 | 16 | 11 |
| Xuzhou112 | 17 | 12 | 20 | 29 | 15 | 10 | 22 | 13 | 11 | 11 | 15 | 19 | 25 | 23 | 14 | 12 | 11 | 12 | 18 | 12,13 | 15 | 12 |
| Xuzhou113 | 17 | 14 | 21 | 30 | 15 | 10 | 24 | 13 | 12 | 10 | 14 | 16 | 22 | 23 | 11 | 11 | 8 | 15 | 15 | 12,19 | 15 | 11 |
| Xuzhou114 | 19 | 12 | 20 | 28 | 14 | 10 | 24 | 12 | 11 | 11 | 15 | 20 | 22 | 25 | 12 | 14 | 11 | 12 | 19 | 13,19 | 16 | 11 |
| Xuzhou115 | 18 | 12 | 19 | 27 | 15 | 10 | 22 | 13 | 11 | 10 | 15 | 15 | 19 | 23 | 12 | 12 | 11 | 12 | 17 | 12,16 | 15 | 11 |
| Xuzhou116 | 17 | 13 | 21 | 28 | 16 | 10 | 24 | 12 | 12 | 10 | 15 | 16 | 21 | 23 | 11 | 11 | 9 | 14 | 16 | 11,20 | 14 | 11 |
| Xuzhou117 | 16 | 12 | 20 | 28 | 15 | 9 | 23 | 12 | 11 | 10 | 14 | 21 | 22 | 25 | 11 | 13 | 10 | 12 | 17 | 16,18 | 13 | 12 |
| Xuzhou118 | 18 | 13 | 21 | 29 | 17 | 11 | 26 | 14 | 12 | 10 | 14 | 16 | 21 | 23 | 11 | 11 | 9 | 14 | 18 | 11,18 | 15 | 11 |
| Xuzhou119 | 20 | 14 | 20 | 30 | 14 | 10 | 24 | 12 | 10 | 11 | 15 | 20 | 21 | 25 | 12 | 13 | 13 | 12 | 17 | 14,18 | 15 | 11 |
| Xuzhou120 | 21 | 13 | 19 | 29 | 17 | 10 | 24 | 13 | 12 | 10 | 14 | 19 | 22 | 23 | 11 | 13 | 10 | 9 | 18 | 12,19 | 14 | 12 |
| Xuzhou121 | 18 | 14 | 21 | 29 | 15 | 10 | 24 | 13 | 12 | 10 | 14 | 16 | 21 | 23 | 12 | 11 | 9 | 14 | 17 | 19,20 | 14 | 11 |
| Xuzhou122 | 19 | 12 | 19 | 29 | 15 | 10 | 20 | 12 | 11 | 10 | 14 | 16 | 19 | 24 | 12 | 12 | 11 | 12 | 18 | 12,17 | 16 | 12 |
| Xuzhou123 | 17 | 12 | 20 | 28 | 16 | 10 | 22 | 12 | 11 | 10 | 14 | 21 | 23 | 25 | 11 | 13 | 10 | 12 | 17 | 14,18 | 15 | 12 |
| Xuzhou124 | 17 | 12 | 18 | 28 | 15 | 11 | 25 | 12 | 11 | 10 | 14 | 20 | 19 | 23 | 11 | 14 | 11 | 13 | 16 | 13,13 | 17 | 12 |
| Xuzhou125 | 17 | 13 | 21 | 29 | 14 | 10 | 22 | 13 | 13 | 9 | 15 | 17 | 21 | 23 | 11 | 11 | 11 | 12 | 15 | 13,17 | 16 | 12 |
| Xuzhou126 | 18 | 14 | 19 | 31 | 14 | 10 | 23 | 10 | 12 | 11 | 14 | 18 | 22 | 23 | 13 | 14 | 11 | 13 | 15 | 11,12 | 16 | 12 |
| Xuzhou127 | 16 | 12 | 19 | 29 | 16 | 10 | 22 | 14 | 12 | 10 | 15 | 17 | 19 | 23 | 12 | 12 | 11 | 12 | 19 | 12,17 | 15 | 12 |
| Xuzhou128 | 17 | 12 | 20 | 28 | 14 | 11 | 23 | 12 | 11 | 11 | 15 | 20 | 22 | 23 | 12 | 14 | 12 | 12 | 18 | 13,16 | 15 | 12 |
| Xuzhou129 | 17 | 14 | 20 | 30 | 15 | 10 | 25 | 13 | 12 | 10 | 14 | 17 | 21 | 23 | 13 | 11 | 8 | 15 | 16 | 12,18 | 16 | 11 |
| Xuzhou130 | 19 | 12 | 20 | 29 | 17 | 10 | 22 | 12 | 14 | 10 | 15 | 18 | 19 | 23 | 12 | 12 | 12 | 12 | 18 | 12,17 | 15 | 13 |
| Xuzhou131 | 18 | 12 | 18 | 29 | 15 | 11 | 25 | 11 | 11 | 10 | 14 | 18 | 21 | 24 | 12 | 14 | 11 | 13 | 15 | 13,13 | 17 | 12 |
| Xuzhou132 | 18 | 12 | 20 | 28 | 15 | 10 | 24 | 13 | 11 | 10 | 14 | 19 | 21 | 24 | 11 | 13 | 11 | 12 | 16 | 13,18 | 14 | 12 |
| Xuzhou133 | 18 | 13 | 21 | 29 | 16 | 10 | 24 | 13 | 12 | 10 | 14 | 16 | 22 | 23 | 11 | 11 | 9 | 14 | 15 | 12,18 | 15 | 12 |
| Xuzhou134 | 18 | 13 | 19 | 28 | 15 | 11 | 24 | 13 | 12 | 10 | 15 | 15 | 21 | 22 | 12 | 13 | 11 | 12 | 16 | 12,18 | 15 | 11 |
| Xuzhou135 | 17 | 13 | 20 | 30 | 17 | 10 | 25 | 12 | 11 | 10 | 14 | 18 | 22 | 23 | 12 | 13 | 10 | 12 | 17 | 12,19 | 16 | 11 |
| Xuzhou136 | 19 | 12 | 19 | 29 | 15 | 10 | 21 | 12 | 12 | 10 | 14 | 17 | 19 | 23 | 12 | 12 | 12 | 12 | 20 | 12,17 | 15 | 12 |
| Xuzhou137 | 18 | 12 | 21 | 28 | 17 | 10 | 24 | 13 | 11 | 9 | 14 | 17 | 22 | 25 | 12 | 13 | 10 | 12 | 18 | 12,19 | 14 | 12 |
| Xuzhou138 | 20 | 12 | 20 | 28 | 13 | 10 | 23 | 12 | 11 | 11 | 16 | 19 | 20 | 24 | 12 | 14 | 11 | 13 | 19 | 14,17 | 15 | 11 |
| Xuzhou139 | 20 | 14 | 20 | 30 | 14 | 10 | 23 | 13 | 10 | 10 | 15 | 19 | 21 | 25 | 12 | 13 | 12 | 12 | 14 | 13,20 | 16 | 11 |
| Xuzhou140 | 20 | 12 | 19 | 28 | 17 | 10 | 24 | 13 | 13 | 10 | 14 | 19 | 22 | 24 | 12 | 14 | 11 | 12 | 16 | 14,18 | 15 | 11 |
| Xuzhou141 | 18 | 12 | 19 | 27 | 15 | 10 | 21 | 13 | 12 | 10 | 15 | 17 | 23 | 24 | 12 | 12 | 12 | 12 | 18 | 12,12 | 15 | 11 |
| Xuzhou142 | 16 | 12 | 20 | 29 | 15 | 10 | 25 | 12 | 12 | 11 | 15 | 19 | 22 | 24 | 11 | 13 | 11 | 12 | 15 | 12,18 | 15 | 12 |
| Xuzhou143 | 19 | 12 | 19 | 28 | 16 | 10 | 24 | 12 | 11 | 10 | 14 | 20 | 24 | 24 | 12 | 13 | 10 | 12 | 18 | 12,20 | 14 | 11 |
| Xuzhou144 | 19 | 12 | 20 | 26 | 14 | 10 | 23 | 14 | 11 | 11 | 15 | 17 | 20 | 24 | 12 | 13 | 11 | 12 | 18 | 13,22 | 15 | 13 |
| Xuzhou145 | 22 | 12 | 19 | 29 | 15 | 11 | 23 | 13 | 11 | 10 | 15 | 17 | 20 | 23 | 14 | 12 | 12 | 12 | 17 | 12,12 | 16 | 12 |
| Xuzhou146 | 18 | 13 | 19 | 30 | 16 | 11 | 22 | 12 | 12 | 10 | 14 | 16 | 22 | 23 | 11 | 13 | 11 | 12 | 20 | 12,19 | 15 | 11 |
| Xuzhou147 | 14 | 14 | 20 | 32 | 14 | 10 | 22 | 12 | 12 | 10 | 14 | 18 | 23 | 23 | 11 | 14 | 10 | 13 | 15 | 11,12 | 18 | 12 |
|  |  |  |  |  |  |  |  |  |  |  |  |  |  |  |  |  |  |  |  |  |  |  |
| Suqian001 | 16 | 12 | 20 | 28 | 15 | 9 | 24 | 13 | 11 | 10 | 14 | 19 | 23 | 25 | 11 | 13 | 10 | 12 | 17 | 15,15 | 14 | 12 |
| Suqian002 | 21 | 12 | 19 | 28 | 13 | 10 | 22 | 13 | 11 | 10 | 15 | 19 | 21 | 25 | 13 | 14 | 9 | 13 | 17 | 12,13 | 15 | 12 |
| Suqian003 | 21 | 13 | 19 | 29 | 15 | 11 | 25 | 13 | 11 | 10 | 15 | 17 | 24 | 25 | 11 | 13 | 10 | 12 | 17 | 13,17 | 15 | 12 |
| Suqian004 | 18 | 11 | 19 | 27 | 15 | 11 | 23 | 11 | 12 | 10 | 15 | 15 | 24 | 24 | 11 | 13 | 11 | 13 | 18 | 12,17 | 14 | 11 |
| Suqian005 | 19 | 14 | 22 | 29 | 16 | 10 | 25 | 12 | 12 | 10 | 14 | 14 | 21 | 23 | 12 | 11 | 9 | 15 | 16 | 11,19 | 15 | 11 |
| Suqian006 | 19 | 12 | 18 | 28 | 16 | 10 | 26 | 12 | 12 | 10 | 14 | 15 | 20 | 23 | 11 | 13 | 11 | 13 | 17 | 12,17 | 15 | 12 |
| Suqian007 | 19 | 12 | 20 | 28 | 15 | 10 | 23 | 12 | 11 | 10 | 14 | 20 | 23 | 25 | 11 | 13 | 10 | 12 | 18 | 14,17 | 15 | 13 |
| Suqian008 | 19 | 12 | 20 | 28 | 14 | 10 | 23 | 14 | 11 | 11 | 15 | 17 | 20 | 26 | 11 | 15 | 12 | 12 | 17 | 13,19 | 16 | 12 |
| Suqian009 | 15 | 12 | 20 | 28 | 16 | 10 | 25 | 12 | 11 | 10 | 14 | 17 | 21 | 24 | 12 | 13 | 11 | 12 | 18 | 12,18 | 14 | 12 |
| Suqian010 | 18 | 12 | 19 | 27 | 16 | 10 | 22 | 13 | 11 | 10 | 14 | 17 | 22 | 25 | 12 | 13 | 10 | 12 | 18 | 11,22 | 14 | 12 |
| Suqian011 | 19 | 12 | 18 | 28 | 15 | 10 | 25 | 12 | 11 | 10 | 14 | 21 | 19 | 23 | 11 | 14 | 11 | 14 | 17 | 13,13 | 18 | 12 |
| Suqian012 | 18 | 14 | 18 | 31 | 15 | 10 | 23 | 13 | 11 | 10 | 14 | 20 | 21 | 24 | 11 | 14 | 11 | 14 | 15 | 12,16 | 14 | 12 |
| Suqian013 | 18 | 14 | 18 | 30 | 15 | 10 | 23 | 12 | 11 | 10 | 14 | 20 | 21 | 24 | 11 | 13 | 11 | 14 | 15 | 12,17 | 15 | 12 |
| Suqian014 | 18 | 14 | 21 | 30 | 15 | 10 | 26 | 13 | 13 | 10 | 14 | 16 | 21 | 23 | 11 | 11 | 8 | 15 | 15 | 12,16 | 15 | 11 |
| Suqian015 | 15 | 13 | 19 | 30 | 15 | 10 | 22 | 12 | 11 | 10 | 14 | 17 | 21 | 23 | 11 | 12 | 12 | 13 | 18 | 15,19 | 15 | 11 |
| Suqian016 | 17 | 12 | 20 | 28 | 16 | 11 | 25 | 11 | 12 | 11 | 14 | 17 | 21 | 25 | 11 | 13 | 12 | 12 | 17 | 13,19 | 15 | 11 |
| Suqian017 | 18 | 14 | 18 | 29 | 16 | 10 | 23 | 12 | 11 | 13 | 14 | 19 | 20 | 23 | 12 | 13 | 13 | 13 | 17 | 10,18 | 15 | 11 |
| Suqian018 | 20 | 14 | 20 | 30 | 14 | 10 | 23 | 12 | 11 | 11 | 15 | 18 | 22 | 25 | 14 | 14 | 11 | 12 | 16 | 13,19 | 16 | 12 |
| Suqian019 | 18 | 12 | 19 | 30 | 17 | 10 | 25 | 12 | 11 | 10 | 14 | 16 | 24 | 24 | 13 | 13 | 10 | 12 | 16 | 12,19 | 14 | 11 |
| Suqian020 | 15 | 12 | 19 | 28 | 15 | 10 | 26 | 12 | 11 | 10 | 14 | 17 | 22 | 24 | 12 | 13 | 10 | 12 | 17 | 12,19 | 15 | 12 |
| Suqian021 | 18 | 13 | 21 | 29 | 15 | 11 | 24 | 12 | 14 | 10 | 14 | 16 | 21 | 23 | 11 | 11 | 9 | 15 | 16 | 11,17 | 15 | 11 |
| Suqian022 | 20 | 12 | 20 | 28 | 15 | 11 | 22 | 13 | 11 | 10 | 15 | 17 | 20 | 24 | 13 | 12 | 11 | 12 | 18 | 12,16 | 16 | 12 |
| Suqian023 | 17 | 12 | 18 | 30 | 15 | 10 | 24 | 12 | 11 | 10 | 14 | 17 | 20 | 23 | 11 | 14 | 11 | 13 | 15 | 13,13 | 16 | 11 |
| Suqian024 | 17 | 14 | 19 | 30 | 15 | 11 | 24 | 13 | 11 | 10 | 14 | 20 | 21 | 24 | 11 | 13 | 11 | 14 | 15 | 12,18 | 15 | 12 |
| Suqian025 | 20 | 14 | 19 | 31 | 15 | 10 | 27 | 13 | 11 | 11 | 15 | 18 | 21 | 22 | 13 | 14 | 12 | 12 | 19 | 14,18 | 15 | 11 |
| Suqian026 | 18 | 12 | 20 | 28 | 14 | 10 | 23 | 11 | 11 | 11 | 15 | 17 | 20 | 24 | 12 | 14 | 11 | 12 | 16 | 13,17 | 14 | 12 |
| Suqian027 | 18 | 12 | 19 | 28 | 15 | 10 | 22 | 12 | 11 | 10 | 15 | 17 | 19 | 23 | 12 | 12 | 11 | 12 | 17 | 12,16 | 15 | 12 |
| Suqian028 | 16 | 12 | 19 | 28 | 15 | 10 | 22 | 13 | 10 | 10 | 15 | 17 | 19 | 23 | 12 | 12 | 11 | 12 | 17 | 12,17 | 15 | 12 |
| Suqian029 | 19 | 12 | 19 | 27 | 14 | 10 | 21 | 13 | 11 | 11 | 15 | 17 | 20 | 24 | 14 | 14 | 12 | 12 | 18 | 14,19 | 15 | 12 |
| Suqian030 | 17 | 13 | 21 | 28 | 15 | 11 | 24 | 12 | 11 | 10 | 14 | 18 | 21 | 22 | 12 | 11 | 9 | 14 | 17 | 11,19 | 15 | 10 |
| Suqian031 | 20 | 12 | 19 | 29 | 15 | 10 | 22 | 12 | 11 | 10 | 15 | 17 | 19 | 23 | 13 | 12 | 11 | 12 | 16 | 12,16 | 16 | 12 |
| Suqian032 | 16 | 12 | 17 | 29 | 15 | 10 | 26 | 13 | 11 | 10 | 14 | 18 | 21 | 24 | 12 | 12 | 11 | 14 | 15 | 13,15 | 16 | 12 |
| Suqian033 | 16 | 12 | 19 | 29 | 15 | 10 | 22 | 13 | 10 | 10 | 15 | 17 | 19 | 23 | 12 | 12 | 11 | 12 | 17 | 12,17 | 15 | 12 |
| Suqian034 | 18 | 14 | 20 | 30 | 14 | 10 | 24 | 12 | 11 | 11 | 14 | 19 | 20 | 23 | 12 | 14 | 11 | 12 | 19 | 13,20 | 15 | 12 |
| Suqian035 | 20 | 13 | 18 | 29 | 15 | 10 | 21 | 13 | 13 | 10 | 14 | 16 | 22 | 22 | 11 | 13 | 12 | 12 | 16 | 12,19 | 15 | 11 |
| Suqian036 | 18 | 13 | 20 | 29 | 15 | 11 | 22 | 12 | 12 | 11 | 15 | 17 | 24 | 23 | 12 | 13 | 11 | 12 | 18 | 12,19 | 15 | 11 |
| Suqian037 | 18 | 12 | 18 | 29 | 15 | 11 | 25 | 12 | 11 | 10 | 14 | 20 | 19 | 23 | 11 | 14 | 11 | 13 | 15 | 13,14 | 17 | 12 |
| Suqian038 | 19 | 13 | 19 | 32 | 16 | 10 | 23 | 15 | 11 | 10 | 15 | 16 | 21 | 24 | 11 | 13 | 10 | 12 | 17 | 13,14 | 13 | 11 |
| Suqian039 | 20 | 12 | 19 | 27 | 16 | 10 | 22 | 11 | 11 | 10 | 15 | 16 | 19 | 23 | 12 | 12 | 11 | 12 | 20 | 12,12 | 15 | 12 |
| Suqian040 | 17 | 12 | 18 | 30 | 16 | 10 | 24 | 12 | 11 | 10 | 14 | 17 | 20 | 23 | 11 | 14 | 11 | 13 | 15 | 13,13 | 16 | 11 |
| Suqian041 | 21 | 13 | 19 | 29 | 16 | 9 | 26 | 13 | 11 | 10 | 15 | 17 | 22 | 24 | 12 | 13 | 10 | 12 | 15 | 12,19 | 14 | 12 |
| Suqian042 | 18 | 12 | 20 | 29 | 14 | 10 | 23 | 14 | 12 | 11 | 15 | 20 | 22 | 25 | 11 | 15 | 11 | 13 | 17 | 14,18 | 15 | 12 |
| Suqian043 | 19 | 12 | 19 | 28 | 15 | 10 | 23 | 12 | 11 | 10 | 14 | 17 | 21 | 23 | 11 | 12 | 11 | 12 | 20 | 12,18 | 15 | 9 |
| Suqian044 | 19 | 12 | 19 | 30 | 14 | 11 | 23 | 13 | 11 | 10 | 15 | 16 | 20 | 24 | 14 | 12 | 11 | 12 | 17 | 12,17 | 16 | 12 |
| Suqian045 | 19 | 13 | 23 | 30 | 15 | 10 | 23 | 13 | 10 | 10 | 15 | 19 | 21 | 24 | 13 | 13 | 13 | 12 | 17 | 12,20 | 15 | 11 |
| Suqian046 | 17 | 13 | 17 | 29 | 15 | 10 | 24 | 12 | 12 | 10 | 14 | 18 | 20 | 23 | 12 | 13 | 11 | 14 | 16 | 10,15 | 15 | 11 |
| Suqian047 | 19 | 14 | 21 | 30 | 15 | 10 | 27 | 13 | 13 | 10 | 14 | 16 | 21 | 23 | 11 | 11 | 8 | 15 | 15 | 12,17 | 15 | 11 |
| Suqian048 | 17 | 13 | 21 | 29 | 17 | 11 | 25 | 12 | 12 | 10 | 14 | 16 | 23 | 23 | 12 | 11 | 9 | 15 | 17 | 11,18 | 15 | 12 |
| Suqian049 | 18 | 12 | 19 | 30 | 17 | 10 | 25 | 12 | 11 | 10 | 14 | 16 | 24 | 25 | 13 | 13 | 10 | 12 | 16 | 12,19 | 14 | 11 |
| Suqian050 | 19 | 13 | 18 | 30 | 16 | 10 | 25 | 12 | 10 | 10 | 14 | 20 | 19 | 23 | 12 | 14 | 12 | 13 | 15 | 13,13 | 17 | 10 |
| Suqian051 | 18 | 13 | 18 | 30 | 15 | 11 | 21 | 13 | 12 | 10 | 14 | 16 | 21 | 22 | 11 | 13 | 11 | 12 | 16 | 12,19 | 15 | 11 |
| Suqian052 | 19 | 13 | 20 | 29 | 14 | 10 | 23 | 12 | 11 | 11 | 15 | 18 | 20 | 24 | 12 | 14 | 11 | 12 | 16 | 13,19 | 15 | 12 |
| Suqian053 | 18 | 12 | 20 | 28 | 14 | 10 | 23 | 13 | 11 | 11 | 14 | 19 | 21 | 23 | 11 | 14 | 11 | 12 | 16 | 13,19 | 15 | 12 |
| Suqian054 | 17 | 12 | 18 | 29 | 14 | 10 | 25 | 13 | 11 | 11 | 14 | 18 | 19 | 23 | 11 | 14 | 11 | 13 | 15 | 12,12 | 19 | 12 |
| Suqian055 | 18 | 14 | 18 | 32 | 16 | 10 | 22 | 12 | 11 | 10 | 14 | 17 | 21 | 22 | 11 | 13 | 11 | 12 | 16 | 11,20 | 15 | 12 |
| Suqian056 | 18 | 12 | 19 | 28 | 14 | 11 | 22 | 14 | 11 | 10 | 15 | 17 | 22 | 23 | 12 | 12 | 11 | 12 | 19 | 12,16 | 16 | 12 |
| Suqian057 | 19 | 13 | 20 | 29 | 16 | 11 | 24 | 12 | 12 | 10 | 14 | 16 | 21 | 23 | 14 | 13 | 9 | 14 | 16 | 11,18 | 15 | 11 |
| Suqian058 | 19 | 12 | 20 | 28 | 15 | 11 | 24 | 14 | 11 | 10 | 15 | 19 | 24 | 24 | 12 | 13 | 10 | 12 | 18 | 13,18 | 13 | 12 |
| Suqian059 | 16 | 13 | 18 | 29 | 15 | 10 | 25 | 13 | 11 | 11 | 14 | 18 | 22 | 23 | 13 | 13 | 8 | 14 | 16 | 12,19 | 16 | 12 |
| Suqian060 | 19 | 12 | 20 | 28 | 14 | 10 | 23 | 13 | 11 | 11 | 15 | 17 | 20 | 23 | 11 | 14 | 11 | 12 | 17 | 15,20 | 15 | 12 |
| Suqian061 | 17 | 13 | 19 | 30 | 15 | 10 | 24 | 12 | 11 | 10 | 14 | 18 | 19 | 23 | 11 | 13 | 11 | 13 | 15 | 13,13 | 0 | 12 |
| Suqian062 | 19 | 13 | 20 | 30 | 14 | 10 | 23 | 12 | 12 | 12 | 14 | 18 | 21 | 25 | 12 | 14 | 11 | 12 | 17 | 13,18 | 15 | 11 |
| Suqian063 | 20 | 13 | 18 | 28 | 17 | 10 | 22 | 13 | 11 | 10 | 15 | 17 | 19 | 23 | 12 | 12 | 11 | 12 | 19 | 12,12 | 15 | 12 |
| Suqian064 | 20 | 12 | 19 | 28 | 15 | 10 | 22 | 13 | 11 | 11 | 15 | 16 | 19 | 23 | 12 | 12 | 11 | 12 | 20 | 12,17 | 16 | 12 |
| Suqian065 | 17 | 12 | 18 | 28 | 15 | 10 | 26 | 12 | 11 | 10 | 14 | 18 | 20 | 23 | 11 | 14 | 12 | 13 | 16 | 13,13 | 17 | 12 |
| Suqian066 | 18 | 12 | 20 | 27 | 16 | 9 | 23 | 13 | 12 | 10 | 14 | 18 | 21 | 25 | 12 | 13 | 10 | 12 | 19 | 14,18 | 14 | 11 |
| Suqian067 | 19 | 14 | 19 | 30 | 13 | 6 | 24 | 12 | 12 | 12 | 14 | 21 | 22 | 24 | 12 | 13 | 10 | 14 | 16 | 16,20 | 17 | 10 |
| Suqian068 | 18 | 13 | 20 | 29 | 15 | 10 | 25 | 11 | 12 | 10 | 14 | 18 | 22 | 23 | 11 | 11 | 9 | 14 | 17 | 11,19 | 16 | 12 |
| Suqian069 | 18 | 12 | 20 | 28 | 15 | 10 | 23 | 12 | 11 | 10 | 14 | 19 | 23 | 25 | 11 | 13 | 10 | 12 | 18 | 14,17 | 16 | 13 |
| Suqian070 | 19 | 14 | 20 | 30 | 14 | 11 | 23 | 12 | 10 | 10 | 15 | 18 | 20 | 28 | 13 | 13 | 12 | 12 | 17 | 13,18 | 16 | 11 |
| Suqian071 | 18 | 14 | 18 | 30 | 16 | 11 | 23 | 12 | 11 | 10 | 14 | 19 | 22 | 25 | 11 | 13 | 10 | 15 | 16 | 12,18 | 14 | 12 |
| Suqian072 | 19 | 13 | 20 | 29 | 14 | 10 | 25 | 13 | 11 | 11 | 16 | 17 | 20 | 24 | 13 | 14 | 12 | 12 | 18 | 13,18 | 15 | 12 |
| Suqian073 | 17 | 12 | 22 | 27 | 15 | 10 | 24 | 13 | 12 | 10 | 14 | 16 | 21 | 22 | 12 | 11 | 9 | 14 | 16 | 11,18 | 15 | 11 |
| Suqian074 | 17 | 12 | 20 | 26 | 17 | 10 | 28 | 12 | 11 | 10 | 14 | 19 | 21 | 25 | 12 | 13 | 10 | 13 | 18 | 13,19 | 14 | 11 |
| Suqian075 | 18 | 12 | 18 | 31 | 15 | 11 | 23 | 12 | 10 | 10 | 14 | 18 | 22 | 24 | 11 | 13 | 12 | 15 | 15 | 14,18 | 15 | 10 |
| Suqian076 | 20 | 13 | 19 | 30 | 15 | 10 | 25 | 12 | 12 | 10 | 15 | 14 | 21 | 24 | 12 | 13 | 11 | 12 | 20 | 12,17 | 17 | 11 |
| Suqian077 | 16 | 12 | 19 | 27 | 17 | 10 | 27 | 13 | 11 | 10 | 14 | 19 | 22 | 23 | 11 | 13 | 10 | 12 | 17 | 14,15 | 14 | 12 |
| Suqian078 | 17 | 13 | 21 | 30 | 17 | 10 | 22 | 12 | 10 | 10 | 14 | 18 | 19 | 23 | 13 | 14 | 11 | 12 | 17 | 12,16 | 15 | 12 |
| Suqian079 | 20 | 12 | 21 | 29 | 14 | 10 | 23 | 12 | 11 | 11 | 15 | 18 | 20 | 24 | 13 | 14 | 11 | 12 | 17 | 13,20 | 16 | 12 |
| Suqian080 | 19 | 14 | 21 | 32 | 15 | 10 | 28 | 11 | 12 | 10 | 14 | 17 | 21 | 23 | 12 | 11 | 8 | 15 | 16 | 12,19 | 15 | 11 |
| Suqian081 | 18 | 14 | 21 | 30 | 15 | 10 | 26 | 14 | 13 | 10 | 14 | 16 | 19 | 23 | 11 | 12 | 8 | 15 | 15 | 12,17 | 15 | 11 |
| Suqian082 | 20 | 12 | 19 | 29 | 15 | 11 | 22 | 12 | 11 | 10 | 15 | 16 | 19 | 24 | 14 | 12 | 11 | 12 | 19 | 12,16 | 15 | 12 |
| Suqian083 | 17 | 12 | 19 | 29 | 13 | 10 | 25 | 12 | 11 | 10 | 14 | 18 | 23 | 26 | 11 | 13 | 10 | 12 | 17 | 12,19 | 14 | 12 |
| Suqian084 | 19 | 14 | 18 | 30 | 15 | 10 | 23 | 13 | 12 | 10 | 14 | 19 | 20 | 24 | 12 | 13 | 11 | 13 | 16 | 12,17 | 16 | 12 |
| Suqian085 | 20 | 12 | 20 | 27 | 14 | 9 | 24 | 13 | 11 | 11 | 15 | 19 | 21 | 24 | 12 | 14 | 11 | 13 | 17 | 13,18 | 16 | 12 |
| Suqian086 | 16 | 12 | 19 | 29 | 15 | 10 | 22 | 12 | 11 | 10 | 15 | 17 | 19 | 23 | 13 | 12 | 11 | 12 | 17 | 13,18 | 15 | 12 |
| Suqian087 | 18 | 14 | 17 | 30 | 15 | 10 | 24 | 12 | 11 | 10 | 14 | 22 | 21 | 24 | 11 | 13 | 11 | 13 | 17 | 13,20 | 15 | 12 |
| Suqian088 | 17 | 12 | 20 | 28 | 17 | 10 | 25 | 14 | 11 | 10 | 14 | 19 | 21 | 25 | 11 | 13 | 10 | 13 | 17 | 14,18 | 14 | 12 |
| Suqian089 | 19 | 12 | 20 | 28 | 15 | 10 | 29 | 12 | 11 | 10 | 15 | 18 | 22 | 22 | 11 | 13 | 11 | 12 | 17 | 12,16 | 14 | 11 |
| Suqian090 | 19 | 12 | 19 | 29 | 17 | 10 | 24 | 13 | 11 | 10 | 15 | 18 | 21 | 24 | 12 | 13 | 10 | 12 | 19 | 12,18 | 14 | 12 |
| Suqian091 | 20 | 12 | 19 | 28 | 15 | 10 | 23 | 12 | 12 | 10 | 15 | 19 | 21 | 23 | 12 | 12 | 12 | 14 | 16 | 12,16 | 15 | 11 |
| Suqian092 | 18 | 13 | 19 | 28 | 15 | 10 | 23 | 13 | 11 | 10 | 15 | 19 | 20 | 22 | 11 | 14 | 10 | 12 | 17 | 11,11 | 16 | 12 |
| Suqian093 | 18 | 11 | 19 | 28 | 15 | 10 | 22 | 14 | 11 | 10 | 14 | 17 | 20 | 23 | 12 | 12 | 11 | 12 | 18 | 12,18 | 15 | 12 |
| Suqian094 | 19 | 13 | 19 | 29 | 14 | 11 | 22 | 13 | 11 | 10 | 15 | 17 | 20 | 23 | 13 | 12 | 11 | 12 | 17 | 12,16 | 18 | 13 |
| Suqian095 | 17 | 13 | 19 | 28 | 14 | 11 | 24 | 12 | 12 | 10 | 14 | 19 | 22 | 24 | 13 | 15 | 11 | 13 | 15 | 11,13 | 15 | 13 |
| Suqian096 | 16 | 13 | 19 | 28 | 14 | 10 | 24 | 12 | 12 | 10 | 14 | 18 | 22 | 26 | 11 | 15 | 11 | 13 | 16 | 9,12 | 15 | 12 |
| Suqian097 | 16 | 13 | 21 | 29 | 17 | 11 | 25 | 13 | 13 | 10 | 14 | 16 | 23 | 23 | 12 | 11 | 9 | 15 | 17 | 11,18 | 15 | 12 |
| Suqian098 | 19 | 12 | 19 | 29 | 15 | 10 | 22 | 12 | 11 | 10 | 15 | 15 | 19 | 23 | 12 | 12 | 11 | 12 | 18 | 12,16 | 15 | 12 |
| Suqian099 | 16 | 13 | 18 | 30 | 15 | 10 | 24 | 12 | 10 | 10 | 14 | 17 | 22 | 23 | 11 | 15 | 10 | 15 | 17 | 12,19 | 15 | 12 |
| Suqian100 | 19 | 13 | 21 | 31 | 15 | 10 | 25 | 12 | 12 | 10 | 14 | 18 | 21 | 23 | 11 | 11 | 9 | 15 | 18 | 12,18 | 15 | 11 |
| Suqian101 | 17 | 13 | 21 | 30 | 16 | 10 | 24 | 12 | 12 | 10 | 14 | 19 | 21 | 24 | 13 | 11 | 9 | 15 | 16 | 11,17 | 15 | 11 |
| Suqian102 | 19 | 12 | 18 | 29 | 15 | 11 | 24 | 14 | 11 | 10 | 14 | 19 | 19 | 23 | 11 | 14 | 11 | 13 | 15 | 12,13 | 17 | 12 |
| Suqian103 | 20 | 12 | 20 | 28 | 14 | 9 | 22 | 12 | 11 | 11 | 15 | 19 | 20 | 24 | 13 | 14 | 12 | 13 | 20 | 13,19 | 15 | 13 |
| Suqian104 | 18 | 14 | 21 | 30 | 15 | 10 | 25 | 12 | 12 | 10 | 14 | 20 | 23 | 23 | 10 | 11 | 8 | 15 | 15 | 11,19 | 15 | 11 |
| Suqian105 | 18 | 14 | 18 | 30 | 16 | 11 | 23 | 12 | 11 | 10 | 14 | 19 | 22 | 25 | 11 | 13 | 10 | 15 | 16 | 12,18 | 14 | 12 |
| Suqian106 | 18 | 13 | 18 | 31 | 15 | 10 | 23 | 12 | 10 | 12 | 14 | 19 | 23 | 24 | 12 | 13 | 11 | 14 | 18 | 12,12 | 15 | 11 |
| Suqian107 | 16 | 12 | 20 | 29 | 16 | 10 | 25 | 13 | 12 | 10 | 13 | 18 | 21 | 24 | 11 | 13 | 10 | 13 | 19 | 13,18 | 14 | 12 |
| Suqian108 | 19 | 13 | 16 | 31 | 14 | 10 | 26 | 13 | 12 | 10 | 14 | 19 | 21 | 24 | 10 | 11 | 9 | 12 | 18 | 14,14 | 15 | 12 |
| Suqian109 | 18 | 12 | 18 | 29 | 16 | 11 | 25 | 12 | 11 | 10 | 14 | 18 | 19 | 23 | 11 | 14 | 11 | 14 | 15 | 13,13 | 16 | 12 |
| Suqian110 | 19 | 14 | 19 | 30 | 15 | 10 | 24 | 11 | 13 | 10 | 14 | 16 | 21 | 24 | 10 | 13 | 11 | 12 | 17 | 13,19 | 15 | 12 |
| Suqian111 | 16 | 12 | 19 | 29 | 15 | 10 | 22 | 13 | 10 | 10 | 15 | 17 | 19 | 23 | 12 | 12 | 11 | 12 | 17 | 12,17 | 15 | 12 |
|  |  |  |  |  |  |  |  |  |  |  |  |  |  |  |  |  |  |  |  |  |  |  |
| Wuxi001 | 14 | 14 | 19 | 31 | 14 | 10 | 22 | 12 | 12 | 10 | 14 | 18 | 23 | 23 | 11 | 14 | 10 | 13 | 15 | 11,12 | 19 | 12 |
| Wuxi002 | 18 | 14 | 19 | 31 | 13 | 9 | 26 | 12 | 11 | 12 | 14 | 18 | 22 | 24 | 10 | 14 | 10 | 14 | 16 | 15,22 | 15 | 10 |
| Wuxi003 | 18 | 12 | 18 | 29 | 15 | 10 | 24 | 12 | 11 | 10 | 14 | 19 | 19 | 23 | 11 | 14 | 11 | 13 | 15 | 13,13 | 16 | 11 |
| Wuxi004 | 20 | 14 | 19 | 30 | 13 | 9 | 24 | 12 | 12 | 12 | 14 | 19 | 22 | 24 | 12 | 14 | 10 | 15 | 17 | 15,22 | 16 | 10 |
| Wuxi005 | 19 | 12 | 18 | 29 | 15 | 11 | 26 | 12 | 11 | 10 | 14 | 17 | 19 | 23 | 11 | 14 | 11 | 13 | 15 | 13,14 | 17 | 11 |
| Wuxi006 | 18 | 14 | 18 | 31 | 15 | 10 | 23 | 13 | 11 | 10 | 14 | 18 | 21 | 24 | 11 | 13 | 11 | 14 | 15 | 12,17 | 15 | 12 |
| Wuxi007 | 17 | 12 | 18 | 29 | 15 | 11 | 25 | 12 | 11 | 10 | 14 | 16 | 19 | 23 | 11 | 14 | 11 | 13 | 15 | 13,13 | 17 | 11 |
| Wuxi008 | 18 | 11 | 20 | 26 | 15 | 10 | 22 | 13 | 11 | 11 | 15 | 19 | 20 | 24 | 11 | 14 | 11 | 12 | 18 | 13,18 | 14 | 12 |
| Wuxi009 | 17 | 13 | 20 | 31 | 15 | 10 | 25 | 12 | 12 | 11 | 14 | 16 | 21 | 23 | 11 | 11 | 9 | 14 | 16 | 11,18 | 15 | 12 |
| Wuxi010 | 17 | 13 | 19 | 29 | 14 | 11 | 24 | 12 | 13 | 10 | 14 | 18 | 21 | 25 | 11 | 15 | 11 | 13 | 16 | 12,12 | 15 | 13 |
| Wuxi011 | 16 | 12 | 19 | 28 | 15 | 10 | 27 | 14 | 12 | 9 | 15 | 14 | 21 | 24 | 10 | 13 | 12 | 13 | 15 | 12,18 | 15 | 13 |
| Wuxi012 | 20 | 13 | 21 | 29 | 18 | 10 | 24 | 13 | 12 | 10 | 14 | 16 | 21 | 22 | 11 | 11 | 9 | 14 | 15 | 12,17 | 15 | 11 |
| Wuxi013 | 20 | 14 | 18 | 30 | 13 | 6 | 24 | 14 | 13 | 12 | 14 | 20 | 22 | 24 | 11 | 14 | 10 | 14 | 17 | 16,19 | 17 | 10 |
| Wuxi014 | 19 | 12 | 18 | 30 | 14 | 11 | 23 | 12 | 11 | 10 | 14 | 18 | 19 | 23 | 12 | 14 | 11 | 13 | 15 | 13,14 | 17 | 12 |
| Wuxi015 | 20 | 12 | 20 | 28 | 14 | 10 | 23 | 12 | 11 | 11 | 15 | 18 | 20 | 23 | 13 | 14 | 11 | 12 | 21 | 15,18 | 15 | 11 |
| Wuxi016 | 18 | 12 | 18 | 29 | 15 | 11 | 25 | 12 | 11 | 10 | 14 | 18 | 19 | 23 | 11 | 14 | 11 | 13 | 15 | 13,13 | 17 | 12 |
| Wuxi017 | 19 | 13 | 19 | 30 | 14 | 10 | 22 | 13 | 13 | 11 | 14 | 18 | 22 | 23 | 11 | 14 | 9 | 13 | 16 | 11,12 | 15 | 12 |
| Wuxi018 | 19 | 13 | 19 | 28 | 17 | 10 | 24 | 12 | 11 | 10 | 14 | 19 | 23 | 25 | 13 | 13 | 10 | 12 | 19 | 12,19 | 14 | 13 |
| Wuxi019 | 19 | 12 | 19 | 27 | 15 | 11 | 25 | 13 | 11 | 10 | 14 | 18 | 21 | 25 | 11 | 13 | 10 | 12 | 18 | 12,19 | 14 | 11 |
| Wuxi020 | 17 | 12 | 19 | 28 | 16 | 10 | 25 | 14 | 11 | 10 | 13 | 20 | 21 | 25 | 13 | 15 | 11 | 12 | 19 | 12,21 | 15 | 12 |
| Wuxi021 | 19 | 12 | 18 | 30 | 15 | 11 | 25 | 12 | 11 | 10 | 14 | 20 | 19 | 23 | 11 | 14 | 11 | 13 | 15 | 13,13 | 16 | 12 |
| Wuxi022 | 17 | 12 | 18 | 28 | 15 | 10 | 24 | 12 | 11 | 10 | 14 | 18 | 21 | 24 | 13 | 13 | 11 | 14 | 15 | 12,16 | 15 | 13 |
| Wuxi023 | 19 | 12 | 18 | 28 | 15 | 11 | 25 | 11 | 11 | 10 | 14 | 18 | 19 | 23 | 11 | 14 | 13 | 13 | 15 | 13,13 | 17 | 12 |
| Wuxi024 | 19 | 12 | 19 | 28 | 14 | 10 | 22 | 14 | 11 | 10 | 15 | 18 | 19 | 24 | 12 | 12 | 11 | 12 | 18 | 12,16 | 15 | 12 |
| Wuxi025 | 19 | 12 | 19 | 30 | 15 | 11 | 23 | 13 | 11 | 10 | 14 | 17 | 21 | 22 | 10 | 13 | 11 | 12 | 18 | 12,20 | 15 | 11 |
| Wuxi026 | 19 | 14 | 22 | 30 | 17 | 10 | 24 | 13 | 12 | 10 | 14 | 18 | 22 | 23 | 13 | 11 | 9 | 14 | 18 | 11,17 | 15 | 11 |
| Wuxi027 | 19 | 12 | 18 | 27 | 16 | 10 | 21 | 13 | 11 | 10 | 14 | 18 | 20 | 23 | 12 | 14 | 11 | 13 | 15 | 12,14 | 17 | 11 |
| Wuxi028 | 16 | 12 | 18 | 28 | 15 | 11 | 25 | 12 | 11 | 10 | 14 | 19 | 19 | 23 | 11 | 14 | 11 | 13 | 15 | 13,13 | 17 | 11 |
| Wuxi029 | 18 | 13 | 19 | 28 | 16 | 10 | 24 | 12 | 11 | 10 | 14 | 18 | 23 | 25 | 12 | 13 | 10 | 12 | 18 | 13,19 | 14 | 13 |
| Wuxi030 | 19 | 12 | 19 | 28 | 15 | 10 | 24 | 13 | 11 | 10 | 14 | 18 | 20 | 23 | 12 | 14 | 11 | 12 | 19 | 13,14 | 16 | 12 |
| Wuxi031 | 19 | 14 | 19 | 29 | 13 | 10 | 24 | 12 | 12 | 12 | 14 | 22 | 22 | 24 | 11 | 14 | 10 | 14 | 17 | 16,22 | 15 | 10 |
| Wuxi032 | 19 | 12 | 18 | 27 | 17 | 10 | 21 | 13 | 11 | 10 | 14 | 18 | 20 | 23 | 12 | 14 | 11 | 13 | 15 | 12,13 | 17 | 12 |
| Wuxi033 | 19 | 13 | 19 | 29 | 17 | 10 | 24 | 13 | 11 | 10 | 14 | 19 | 21 | 25 | 12 | 13 | 11 | 12 | 18 | 12,19 | 14 | 11 |
| Wuxi034 | 18 | 12 | 19 | 28 | 17 | 10 | 24 | 13 | 11 | 10 | 14 | 17 | 21 | 23 | 11 | 13 | 10 | 12 | 17 | 12,18 | 14 | 12 |
| Wuxi035 | 21 | 12 | 18 | 28 | 16 | 10 | 25 | 12 | 12 | 10 | 15 | 16 | 21 | 23 | 11 | 14 | 13 | 12 | 18 | 12,16 | 16 | 12 |
| Wuxi036 | 18 | 11 | 19 | 29 | 15 | 11 | 22 | 12 | 12 | 10 | 14 | 17 | 22 | 23 | 10 | 13 | 11 | 12 | 18 | 12,19 | 15 | 11 |
| Wuxi037 | 18 | 12 | 18 | 28 | 14 | 11 | 25 | 12 | 11 | 10 | 14 | 18 | 19 | 23 | 11 | 14 | 11 | 12 | 16 | 13,13 | 16 | 12 |
| Wuxi038 | 17 | 12 | 20 | 28 | 17 | 10 | 26 | 11 | 11 | 10 | 15 | 16 | 21 | 24 | 13 | 14 | 12 | 12 | 18 | 13,21 | 15 | 11 |
| Wuxi039 | 17 | 13 | 20 | 29 | 17 | 10 | 25 | 12 | 11 | 10 | 14 | 21 | 23 | 24 | 11 | 14 | 10 | 12 | 18 | 15,18 | 14 | 12 |
| Wuxi040 | 20 | 13 | 19 | 29 | 15 | 10 | 22 | 12 | 11 | 10 | 15 | 16 | 19 | 23 | 12 | 12 | 10 | 12 | 19 | 12,13 | 15 | 13 |
| Wuxi041 | 18 | 13 | 19 | 28 | 17 | 10 | 24 | 12 | 11 | 10 | 14 | 17 | 26 | 25 | 12 | 13 | 10 | 12 | 20 | 13,19 | 14 | 12 |
| Wuxi042 | 17 | 12 | 18 | 30 | 16 | 11 | 26 | 12 | 12 | 9 | 14 | 18 | 19 | 23 | 11 | 14 | 11 | 13 | 15 | 13,13 | 17 | 12 |
| Wuxi043 | 17 | 13 | 19 | 31 | 16 | 11 | 24 | 12 | 13 | 11 | 14 | 20 | 23 | 26 | 10 | 11 | 10 | 13 | 15 | 11,14 | 15 | 13 |
| Wuxi044 | 18 | 13 | 20 | 29 | 14 | 10 | 23 | 12 | 12 | 11 | 15 | 18 | 20 | 24 | 12 | 14 | 12 | 12 | 18 | 13,19 | 14 | 12 |
| Wuxi045 | 14 | 14 | 19 | 31 | 14 | 10 | 22 | 12 | 12 | 10 | 14 | 18 | 23 | 23 | 11 | 14 | 10 | 13 | 15 | 11,12 | 16 | 12 |
| Wuxi046 | 16 | 12 | 20 | 29 | 17 | 10 | 24 | 13 | 11 | 10 | 14 | 20 | 22 | 25 | 11 | 13 | 11 | 12 | 18 | 14,18 | 14 | 12 |
| Wuxi047 | 19 | 13 | 19 | 28 | 15 | 10 | 22 | 14 | 11 | 10 | 15 | 18 | 20 | 22 | 11 | 14 | 10 | 12 | 17 | 11,11 | 16 | 12 |
| Wuxi048 | 19 | 12 | 18 | 27 | 16 | 11 | 21 | 13 | 11 | 10 | 14 | 19 | 21 | 23 | 12 | 14 | 11 | 13 | 15 | 12,13 | 18 | 12 |
| Wuxi049 | 18 | 12 | 19 | 28 | 15 | 11 | 23 | 13 | 11 | 10 | 15 | 18 | 20 | 23 | 13 | 12 | 11 | 12 | 17 | 12,16 | 16 | 11 |
| Wuxi050 | 20 | 12 | 18 | 28 | 15 | 10 | 23 | 12 | 11 | 11 | 14 | 20 | 20 | 23 | 12 | 14 | 11 | 13 | 16 | 13,13 | 15 | 11 |
| Wuxi051 | 17 | 12 | 18 | 30 | 15 | 10 | 25 | 14 | 11 | 10 | 14 | 17 | 19 | 23 | 11 | 14 | 11 | 13 | 15 | 13,13 | 16 | 11 |
| Wuxi052 | 18 | 12 | 18 | 28 | 15 | 10 | 24 | 13 | 11 | 10 | 14 | 18 | 18 | 23 | 11 | 14 | 11 | 13 | 15 | 13,14 | 16 | 12 |
| Wuxi053 | 14 | 14 | 19 | 30 | 14 | 10 | 22 | 12 | 12 | 10 | 14 | 18 | 22 | 23 | 11 | 12 | 10 | 13 | 15 | 11,12 | 17 | 12 |
| Wuxi054 | 20 | 13 | 18 | 30 | 15 | 10 | 22 | 12 | 10 | 11 | 14 | 17 | 22 | 25 | 12 | 13 | 11 | 14 | 17 | 14,18 | 15 | 11 |
| Wuxi055 | 17 | 13 | 19 | 28 | 15 | 11 | 22 | 14 | 11 | 10 | 14 | 17 | 21 | 24 | 12 | 12 | 12 | 12 | 17 | 14,18 | 16 | 11 |
| Wuxi056 | 19 | 13 | 18 | 29 | 14 | 11 | 21 | 12 | 13 | 10 | 14 | 18 | 20 | 23 | 10 | 14 | 11 | 13 | 17 | 11,12 | 13 | 11 |
| Wuxi057 | 19 | 13 | 19 | 30 | 15 | 10 | 22 | 12 | 11 | 10 | 14 | 15 | 21 | 23 | 11 | 13 | 9 | 12 | 16 | 12,19 | 15 | 11 |
| Wuxi058 | 17 | 13 | 20 | 29 | 15 | 10 | 26 | 12 | 12 | 10 | 14 | 16 | 20 | 23 | 11 | 11 | 9 | 14 | 16 | 11,21 | 16 | 12 |
| Wuxi059 | 18 | 12 | 18 | 29 | 16 | 11 | 28 | 12 | 12 | 10 | 14 | 17 | 19 | 23 | 12 | 14 | 12 | 13 | 15 | 13,13 | 16 | 12 |
| Wuxi060 | 16 | 13 | 17 | 30 | 14 | 11 | 27 | 13 | 11 | 10 | 14 | 21 | 21 | 24 | 10 | 11 | 10 | 13 | 17 | 14,19 | 16 | 11 |
| Wuxi061 | 18 | 13 | 20 | 29 | 14 | 10 | 22 | 12 | 12 | 10 | 14 | 18 | 21 | 23 | 11 | 15 | 11 | 13 | 17 | 12,13 | 15 | 11 |
| Wuxi062 | 16 | 12 | 17 | 29 | 16 | 10 | 24 | 12 | 10 | 10 | 14 | 19 | 21 | 23 | 12 | 12 | 11 | 14 | 14 | 12,14 | 16 | 12 |
| Wuxi063 | 18 | 13 | 18 | 29 | 16 | 10 | 23 | 13 | 10 | 10 | 14 | 19 | 23 | 23 | 12 | 13 | 12 | 14 | 17 | 12,21 | 15 | 11 |
| Wuxi064 | 14 | 14 | 19 | 29 | 14 | 10 | 22 | 12 | 12 | 10 | 14 | 17 | 22 | 23 | 11 | 14 | 10 | 13 | 15 | 11,12 | 16 | 12 |
| Wuxi065 | 19 | 13 | 19 | 29 | 17 | 10 | 24 | 12 | 11 | 10 | 14 | 20 | 21 | 25 | 12 | 13 | 11 | 12 | 19 | 12,18 | 14 | 11 |
| Wuxi066 | 17 | 12 | 20 | 28 | 14 | 10 | 23 | 12 | 11 | 11 | 15 | 20 | 20 | 23 | 11 | 14 | 12 | 12 | 22 | 13,19 | 14 | 12 |
| Wuxi067 | 20 | 12 | 18 | 30 | 15 | 11 | 25 | 13 | 11 | 10 | 14 | 20 | 21 | 22 | 13 | 14 | 11 | 13 | 15 | 13,14 | 16 | 12 |
| Wuxi068 | 17 | 12 | 20 | 30 | 15 | 10 | 24 | 12 | 11 | 10 | 15 | 18 | 21 | 25 | 11 | 13 | 11 | 12 | 16 | 13,21 | 14 | 13 |
| Wuxi069 | 19 | 14 | 19 | 31 | 13 | 9 | 24 | 13 | 12 | 12 | 14 | 18 | 22 | 24 | 11 | 14 | 10 | 13 | 16 | 16,21 | 16 | 10 |
| Wuxi070 | 17 | 13 | 20 | 30 | 16 | 11 | 28 | 13 | 11 | 10 | 15 | 15 | 22 | 25 | 11 | 13 | 10 | 12 | 18 | 12,20 | 16 | 11 |
| Wuxi071 | 18 | 12 | 18 | 29 | 16 | 11 | 25 | 12 | 11 | 10 | 14 | 16 | 19 | 23 | 12 | 15 | 11 | 13 | 15 | 13,13 | 17 | 11 |
| Wuxi072 | 17 | 14 | 19 | 30 | 14 | 10 | 23 | 13 | 12 | 10 | 14 | 20 | 21 | 22 | 10 | 14 | 11 | 13 | 18 | 11,12 | 15 | 13 |
| Wuxi073 | 19 | 12 | 20 | 29 | 14 | 11 | 23 | 13 | 10 | 11 | 15 | 19 | 21 | 25 | 12 | 13 | 12 | 12 | 17 | 13,17 | 16 | 11 |
| Wuxi074 | 19 | 12 | 19 | 27 | 15 | 10 | 22 | 14 | 11 | 11 | 15 | 18 | 20 | 25 | 11 | 14 | 11 | 12 | 18 | 13,17 | 15 | 12 |
| Wuxi075 | 18 | 12 | 18 | 27 | 16 | 11 | 21 | 13 | 11 | 10 | 14 | 18 | 20 | 23 | 12 | 14 | 10 | 13 | 15 | 12,13 | 16 | 12 |
| Wuxi076 | 18 | 12 | 18 | 28 | 15 | 11 | 25 | 12 | 11 | 10 | 14 | 19 | 19 | 23 | 11 | 14 | 11 | 13 | 14 | 13,14 | 17 | 12 |
| Wuxi077 | 19 | 14 | 18 | 30 | 14 | 9 | 24 | 12 | 12 | 12 | 14 | 20 | 22 | 24 | 11 | 14 | 10 | 14 | 17 | 15,21 | 17 | 10 |
| Wuxi078 | 18 | 12 | 19 | 28 | 15 | 10 | 23 | 12 | 11 | 11 | 14 | 19 | 20 | 23 | 12 | 14 | 11 | 12 | 18 | 13,15 | 16 | 12 |
| Wuxi079 | 18 | 13 | 20 | 29 | 14 | 10 | 23 | 12 | 11 | 11 | 15 | 18 | 20 | 24 | 11 | 14 | 11 | 12 | 19 | 13,19 | 15 | 12 |
| Wuxi080 | 18 | 13 | 21 | 29 | 16 | 10 | 24 | 13 | 12 | 10 | 14 | 15 | 23 | 23 | 12 | 11 | 9 | 14 | 15 | 11,17 | 15 | 11 |
| Wuxi081 | 18 | 12 | 19 | 28 | 13 | 11 | 23 | 12 | 11 | 10 | 15 | 17 | 19 | 24 | 13 | 12 | 12 | 12 | 18 | 12,16 | 15 | 12 |
| Wuxi082 | 20 | 13 | 19 | 29 | 15 | 10 | 25 | 12 | 12 | 10 | 15 | 14 | 21 | 24 | 12 | 13 | 11 | 12 | 19 | 12,17 | 16 | 11 |
| Wuxi083 | 16 | 12 | 20 | 27 | 16 | 10 | 22 | 12 | 11 | 11 | 14 | 19 | 21 | 25 | 11 | 13 | 10 | 12 | 18 | 14,17 | 15 | 12 |
| Wuxi084 | 18 | 12 | 20 | 28 | 14 | 10 | 24 | 12 | 11 | 11 | 15 | 19 | 20 | 24 | 11 | 14 | 11 | 12 | 19 | 14,20 | 15 | 12 |
| Wuxi085 | 19 | 12 | 19 | 31 | 15 | 10 | 22 | 12 | 11 | 8 | 15 | 16 | 19 | 23 | 12 | 12 | 10 | 13 | 18 | 12,13 | 15 | 12 |
| Wuxi086 | 18 | 12 | 18 | 27 | 15 | 10 | 27 | 12 | 11 | 10 | 14 | 18 | 20 | 22 | 13 | 14 | 11 | 13 | 16 | 13,13 | 15 | 12 |
| Wuxi087 | 18 | 12 | 19 | 28 | 14 | 10 | 22 | 12 | 12 | 10 | 14 | 15 | 20 | 23 | 12 | 12 | 11 | 12 | 17 | 12,16 | 17 | 12 |
| Wuxi088 | 19 | 13 | 19 | 29 | 16 | 10 | 23 | 12 | 11 | 10 | 15 | 16 | 19 | 23 | 11 | 12 | 12 | 13 | 18 | 12,16 | 16 | 12 |
| Wuxi089 | 16 | 12 | 19 | 28 | 14 | 11 | 24 | 13 | 11 | 11 | 15 | 17 | 21 | 24 | 11 | 14 | 11 | 12 | 19 | 13,19 | 15 | 12 |
| Wuxi090 | 18 | 13 | 19 | 29 | 17 | 10 | 22 | 12 | 11 | 10 | 15 | 17 | 20 | 23 | 14 | 12 | 11 | 12 | 20 | 12,16 | 16 | 12 |
| Wuxi091 | 19 | 12 | 19 | 28 | 16 | 12 | 25 | 13 | 11 | 10 | 14 | 19 | 21 | 25 | 12 | 13 | 10 | 12 | 17 | 14,19 | 15 | 11 |
| Wuxi092 | 17 | 12 | 20 | 29 | 15 | 11 | 22 | 13 | 11 | 10 | 15 | 16 | 20 | 23 | 14 | 12 | 11 | 12 | 16 | 12,16 | 15 | 11 |
| Wuxi093 | 19 | 12 | 18 | 28 | 15 | 11 | 25 | 12 | 11 | 10 | 14 | 18 | 19 | 23 | 11 | 14 | 11 | 13 | 15 | 13,13 | 17 | 12 |
| Wuxi094 | 17 | 12 | 18 | 29 | 15 | 10 | 25 | 12 | 11 | 10 | 14 | 18 | 19 | 23 | 11 | 14 | 11 | 13 | 15 | 13,15 | 17 | 12 |
| Wuxi095 | 16 | 14 | 19 | 30 | 14 | 10 | 21 | 12 | 12 | 10 | 14 | 17 | 22 | 22 | 10 | 14 | 11 | 13 | 17 | 12,12 | 15 | 12 |
| Wuxi096 | 17 | 13 | 19 | 29 | 15 | 11 | 22 | 13 | 12 | 10 | 14 | 17 | 22 | 23 | 11 | 13 | 11 | 12 | 20 | 12,12 | 15 | 12 |
| Wuxi097 | 18 | 14 | 18 | 30 | 15 | 10 | 23 | 12 | 11 | 10 | 14 | 18 | 21 | 24 | 11 | 13 | 11 | 14 | 14 | 12,15 | 15 | 12 |
| Wuxi098 | 20 | 14 | 19 | 30 | 13 | 9 | 24 | 12 | 12 | 12 | 14 | 19 | 22 | 24 | 11 | 14 | 11 | 14 | 19 | 15,22 | 17 | 10 |
|  |  |  |  |  |  |  |  |  |  |  |  |  |  |  |  |  |  |  |  |  |  |  |
| Lianyungang001 | 17 | 14 | 17 | 29 | 14 | 10 | 25 | 12 | 12 | 10 | 14 | 19 | 22 | 22 | 10 | 14 | 11 | 13 | 19 | 11,12 | 14 | 11 |
| Lianyungang002 | 17 | 12 | 20 | 29 | 16 | 10 | 22 | 12 | 11 | 10 | 15 | 18 | 20 | 23 | 12 | 13 | 11 | 13 | 17 | 11,11 | 16 | 12 |
| Lianyungang003 | 19 | 12 | 19 | 27 | 16 | 10 | 27 | 14 | 11 | 10 | 14 | 17 | 20 | 25 | 13 | 13 | 10 | 12 | 18 | 12,20 | 14 | 12 |
| Lianyungang004 | 18 | 13 | 19 | 28 | 15 | 10 | 29 | 12 | 11 | 10 | 15 | 18 | 20 | 24 | 13 | 14 | 12 | 12 | 18 | 13,20 | 15 | 11 |
| Lianyungang005 | 18 | 12 | 17 | 30 | 15 | 10 | 24 | 13 | 11 | 10 | 14 | 16 | 20 | 23 | 11 | 14 | 11 | 13 | 15 | 13,13 | 16 | 10 |
| Lianyungang006 | 19 | 12 | 20 | 28 | 14 | 10 | 23 | 13 | 12 | 11 | 16 | 18 | 20 | 25 | 13 | 14 | 11 | 12 | 19 | 17,19 | 15 | 12 |
| Lianyungang007 | 16 | 12 | 18 | 29 | 15 | 10 | 23 | 12 | 11 | 10 | 14 | 19 | 19 | 23 | 11 | 14 | 11 | 13 | 16 | 13,13 | 18 | 12 |
| Lianyungang008 | 18 | 12 | 18 | 29 | 15 | 11 | 25 | 12 | 11 | 10 | 14 | 19 | 19 | 23 | 11 | 14 | 11 | 13 | 15 | 13,13 | 17 | 12 |
| Lianyungang009 | 19 | 12 | 19 | 29 | 13 | 11 | 22 | 12 | 11 | 10 | 15 | 15 | 20 | 23 | 11 | 12 | 11 | 12 | 17 | 12,12 | 15 | 12 |
| Lianyungang010 | 16 | 12 | 19 | 28 | 16 | 10 | 25 | 12 | 12 | 10 | 13 | 20 | 22 | 23 | 11 | 13 | 11 | 12 | 19 | 15,16 | 14 | 12 |
| Lianyungang011 | 16 | 12 | 18 | 27 | 16 | 11 | 21 | 12 | 11 | 10 | 14 | 18 | 20 | 23 | 12 | 14 | 11 | 13 | 15 | 12,13 | 17 | 12 |
| Lianyungang012 | 18 | 13 | 18 | 30 | 17 | 10 | 24 | 12 | 10 | 10 | 14 | 18 | 21 | 25 | 12 | 13 | 13 | 13 | 16 | 13,18 | 15 | 11 |
| Lianyungang013 | 19 | 14 | 22 | 29 | 16 | 10 | 25 | 12 | 12 | 10 | 14 | 14 | 21 | 23 | 11 | 11 | 9 | 15 | 16 | 10,19 | 15 | 11 |
| Lianyungang014 | 17 | 15 | 18 | 33 | 16 | 10 | 24 | 13 | 12 | 10 | 14 | 18 | 21 | 23 | 12 | 13 | 10 | 12 | 17 | 11,20 | 16 | 12 |
| Lianyungang015 | 19 | 13 | 19 | 31 | 17 | 10 | 24 | 13 | 12 | 10 | 15 | 17 | 21 | 22 | 12 | 13 | 10 | 12 | 19 | 11,18 | 15 | 12 |
| Lianyungang016 | 18 | 13 | 20 | 29 | 15 | 10 | 28 | 11 | 12 | 10 | 14 | 21 | 20 | 24 | 12 | 10 | 9 | 12 | 21 | 15,17 | 14 | 11 |
| Lianyungang017 | 20 | 12 | 19 | 27 | 15 | 11 | 24 | 13 | 11 | 10 | 15 | 17 | 20 | 23 | 12 | 12 | 11 | 13 | 19 | 12,16 | 15 | 11 |
| Lianyungang018 | 19 | 14 | 19 | 31 | 15 | 10 | 24 | 12 | 11 | 10 | 14 | 19 | 21 | 23 | 11 | 13 | 11 | 14 | 15 | 12,19 | 15 | 11 |
| Lianyungang019 | 18 | 13 | 19 | 28 | 16 | 10 | 24 | 12 | 12 | 10 | 14 | 18 | 24 | 23 | 12 | 13 | 10 | 12 | 18 | 13,20 | 14 | 13 |
| Lianyungang020 | 17 | 14 | 21 | 30 | 16 | 10 | 24 | 12 | 14 | 10 | 14 | 17 | 21 | 23 | 11 | 11 | 10 | 15 | 16 | 11,17 | 15 | 11 |
| Lianyungang021 | 18 | 12 | 20 | 28 | 14 | 10 | 24 | 13 | 11 | 11 | 15 | 16 | 20 | 24 | 11 | 14 | 11 | 13 | 19 | 13,17 | 15 | 12 |
| Lianyungang022 | 20 | 12 | 19 | 29 | 15 | 10 | 22 | 13 | 11 | 10 | 14 | 16 | 19 | 23 | 12 | 12 | 11 | 13 | 16 | 13,17 | 15 | 13 |
| Lianyungang023 | 18 | 13 | 19 | 29 | 15 | 10 | 28 | 11 | 12 | 10 | 14 | 19 | 21 | 24 | 11 | 10 | 9 | 12 | 23 | 16,16 | 15 | 11 |
| Lianyungang024 | 17 | 12 | 19 | 29 | 16 | 11 | 24 | 14 | 12 | 10 | 15 | 19 | 23 | 24 | 11 | 12 | 11 | 12 | 15 | 13,19 | 15 | 12 |
| Lianyungang025 | 16 | 12 | 19 | 28 | 16 | 10 | 26 | 12 | 11 | 10 | 14 | 19 | 22 | 24 | 12 | 13 | 10 | 12 | 17 | 14,17 | 14 | 11 |
| Lianyungang026 | 19 | 13 | 19 | 30 | 16 | 10 | 23 | 12 | 11 | 10 | 14 | 18 | 21 | 22 | 13 | 13 | 10 | 12 | 18 | 11,20 | 16 | 11 |
| Lianyungang027 | 19 | 12 | 19 | 28 | 17 | 11 | 25 | 13 | 11 | 10 | 14 | 17 | 21 | 25 | 13 | 13 | 11 | 12 | 18 | 14,19 | 15 | 12 |
| Lianyungang028 | 17 | 13 | 19 | 29 | 15 | 10 | 28 | 11 | 12 | 10 | 14 | 20 | 21 | 24 | 11 | 10 | 9 | 12 | 23 | 16,16 | 15 | 11 |
| Lianyungang029 | 16 | 12 | 20 | 29 | 16 | 10 | 26 | 13 | 11 | 10 | 14 | 20 | 22 | 25 | 11 | 13 | 10 | 12 | 20 | 14,19 | 14 | 11 |
| Lianyungang030 | 16 | 13 | 19 | 27 | 14 | 11 | 24 | 12 | 12 | 10 | 14 | 19 | 21 | 24 | 11 | 15 | 11 | 13 | 16 | 11,12 | 15 | 12 |
| Lianyungang031 | 17 | 13 | 19 | 28 | 15 | 11 | 22 | 12 | 11 | 10 | 14 | 17 | 22 | 23 | 11 | 12 | 12 | 13 | 17 | 14,18 | 16 | 13 |
| Lianyungang032 | 18 | 14 | 19 | 30 | 13 | 9 | 26 | 12 | 12 | 12 | 14 | 19 | 22 | 24 | 10 | 14 | 10 | 14 | 17 | 15,19 | 15 | 10 |
| Lianyungang033 | 19 | 12 | 19 | 28 | 15 | 11 | 25 | 12 | 11 | 10 | 14 | 18 | 21 | 24 | 9 | 13 | 10 | 12 | 19 | 12,19 | 14 | 11 |
| Lianyungang034 | 16 | 12 | 20 | 28 | 17 | 10 | 24 | 13 | 11 | 11 | 14 | 19 | 22 | 25 | 11 | 13 | 10 | 12 | 19 | 17,18 | 14 | 13 |
| Lianyungang035 | 18 | 14 | 19 | 30 | 14 | 11 | 21 | 12 | 13 | 10 | 14 | 19 | 20 | 23 | 10 | 14 | 11 | 13 | 16 | 11,12 | 14 | 11 |
| Lianyungang036 | 17 | 12 | 19 | 28 | 17 | 11 | 24 | 13 | 12 | 10 | 15 | 19 | 22 | 24 | 11 | 12 | 11 | 12 | 15 | 13,20 | 15 | 12 |
| Lianyungang037 | 19 | 14 | 18 | 30 | 17 | 10 | 21 | 12 | 11 | 13 | 14 | 18 | 20 | 23 | 11 | 13 | 12 | 13 | 17 | 10,21 | 16 | 11 |
| Lianyungang038 | 20 | 12 | 19 | 28 | 15 | 11 | 22 | 15 | 11 | 11 | 15 | 14 | 20 | 23 | 11 | 12 | 11 | 12 | 17 | 12,16 | 15 | 12 |
| Lianyungang039 | 17 | 12 | 19 | 28 | 16 | 10 | 25 | 13 | 11 | 10 | 14 | 19 | 22 | 25 | 12 | 13 | 12 | 12 | 21 | 13,18 | 14 | 11 |
| Lianyungang040 | 18 | 12 | 19 | 29 | 15 | 10 | 22 | 11 | 11 | 10 | 15 | 17 | 21 | 23 | 13 | 12 | 12 | 12 | 20 | 12,15 | 15 | 12 |
| Lianyungang041 | 16 | 13 | 21 | 29 | 15 | 11 | 24 | 13 | 12 | 10 | 14 | 16 | 21 | 23 | 10 | 11 | 10 | 15 | 19 | 11,18 | 16 | 12 |
| Lianyungang042 | 18 | 14 | 20 | 30 | 15 | 10 | 24 | 11 | 12 | 10 | 14 | 17 | 22 | 23 | 11 | 13 | 11 | 14 | 17 | 13,17 | 14 | 12 |
| Lianyungang043 | 19 | 13 | 18 | 30 | 15 | 11 | 21 | 13 | 12 | 10 | 14 | 16 | 22 | 22 | 11 | 13 | 11 | 12 | 16 | 12,19 | 15 | 11 |
| Lianyungang044 | 18 | 12 | 20 | 30 | 15 | 10 | 24 | 12 | 11 | 11 | 15 | 18 | 20 | 25 | 11 | 14 | 11 | 12 | 17 | 13,19 | 15 | 12 |
| Lianyungang045 | 17 | 13 | 21 | 30 | 16 | 10 | 23 | 12 | 12 | 10 | 14 | 17 | 24 | 23 | 11 | 11 | 9 | 15 | 18 | 11,17 | 15 | 11 |
| Lianyungang046 | 16 | 13 | 18 | 30 | 15 | 10 | 23 | 11 | 11 | 10 | 14 | 21 | 21 | 24 | 11 | 13 | 11 | 14 | 15 | 12,17 | 15 | 12 |
| Lianyungang047 | 20 | 12 | 19 | 29 | 14 | 11 | 22 | 12 | 11 | 10 | 15 | 17 | 20 | 23 | 13 | 12 | 11 | 12 | 18 | 12,17 | 16 | 12 |
| Lianyungang048 | 19 | 12 | 18 | 28 | 16 | 11 | 25 | 12 | 11 | 10 | 14 | 16 | 20 | 23 | 10 | 14 | 11 | 13 | 15 | 13,13 | 17 | 12 |
| Lianyungang049 | 19 | 12 | 19 | 29 | 15 | 10 | 18 | 12 | 11 | 10 | 14 | 16 | 19 | 24 | 12 | 12 | 11 | 12 | 18 | 12,17 | 15 | 12 |
| Lianyungang050 | 18 | 12 | 20 | 28 | 14 | 10 | 23 | 12 | 11 | 11 | 14 | 18 | 21 | 24 | 13 | 14 | 11 | 12 | 16 | 12,17 | 15 | 12 |
| Lianyungang051 | 21 | 12 | 20 | 28 | 14 | 10 | 22 | 12 | 11 | 12 | 15 | 17 | 20 | 23 | 12 | 14 | 11 | 12 | 18 | 13,19 | 16 | 12 |
| Lianyungang052 | 18 | 13 | 22 | 29 | 15 | 10 | 25 | 12 | 11 | 10 | 14 | 16 | 23 | 23 | 11 | 11 | 9 | 15 | 17 | 11,18 | 15 | 12 |
| Lianyungang053 | 18 | 12 | 20 | 28 | 14 | 10 | 23 | 13 | 11 | 11 | 14 | 18 | 20 | 23 | 11 | 14 | 11 | 12 | 17 | 13,19 | 15 | 12 |
| Lianyungang054 | 19 | 12 | 20 | 28 | 16 | 11 | 25 | 13 | 11 | 10 | 15 | 18 | 20 | 24 | 13 | 13 | 11 | 12 | 18 | 13,18 | 15 | 12 |
| Lianyungang055 | 18 | 13 | 18 | 30 | 17 | 10 | 24 | 12 | 10 | 10 | 14 | 19 | 22 | 25 | 11 | 13 | 12 | 13 | 17 | 13,18 | 15 | 11 |
| Lianyungang056 | 22 | 12 | 19 | 29 | 15 | 10 | 22 | 12 | 12 | 10 | 15 | 17 | 19 | 23 | 12 | 7 | 11 | 12 | 16 | 12,16 | 15 | 12 |
| Lianyungang057 | 19 | 13 | 19 | 29 | 14 | 10 | 23 | 12 | 12 | 11 | 14 | 18 | 22 | 23 | 11 | 14 | 10 | 13 | 16 | 11,12 | 15 | 12 |
| Lianyungang058 | 19 | 12 | 20 | 29 | 16 | 10 | 23 | 13 | 11 | 10 | 15 | 19 | 21 | 25 | 12 | 13 | 13 | 12 | 19 | 15,20 | 15 | 12 |
| Lianyungang059 | 16 | 12 | 18 | 28 | 15 | 10 | 26 | 13 | 11 | 10 | 14 | 21 | 20 | 22 | 11 | 14 | 11 | 13 | 15 | 13,14 | 15 | 12 |
| Lianyungang060 | 20 | 14 | 18 | 30 | 13 | 9 | 23 | 12 | 12 | 12 | 14 | 20 | 23 | 24 | 11 | 15 | 10 | 14 | 16 | 15,22 | 16 | 10 |
| Lianyungang061 | 17 | 12 | 18 | 28 | 15 | 10 | 24 | 14 | 11 | 10 | 14 | 19 | 21 | 23 | 12 | 14 | 11 | 13 | 14 | 12,13 | 14 | 12 |
| Lianyungang062 | 18 | 12 | 18 | 29 | 16 | 11 | 24 | 12 | 11 | 10 | 14 | 18 | 19 | 23 | 11 | 14 | 11 | 13 | 15 | 13,13 | 17 | 12 |
| Lianyungang063 | 18 | 14 | 21 | 30 | 15 | 10 | 23 | 11 | 11 | 9 | 14 | 18 | 22 | 24 | 12 | 12 | 10 | 12 | 17 | 15,21 | 15 | 11 |
| Lianyungang064 | 19 | 13 | 20 | 29 | 14 | 10 | 23 | 12 | 10 | 10 | 15 | 20 | 21 | 24 | 14 | 13 | 12 | 12 | 16 | 13,20 | 15 | 10 |
| Lianyungang065 | 18 | 13 | 21 | 29 | 17 | 10 | 24 | 13 | 12 | 10 | 14 | 16 | 21 | 23 | 11 | 11 | 9 | 14 | 16 | 11,15 | 15 | 11 |
| Lianyungang066 | 16 | 14 | 21 | 33 | 13 | 11 | 25 | 12 | 10 | 10 | 14 | 20 | 23 | 25 | 12 | 11 | 12 | 12 | 18 | 15,17 | 15 | 11 |
| Lianyungang067 | 18 | 13 | 19 | 29 | 16 | 10 | 24 | 12 | 13 | 10 | 14 | 15 | 22 | 23 | 13 | 11 | 9 | 14 | 18 | 11,19 | 15 | 11 |
| Lianyungang068 | 18 | 12 | 20 | 28 | 15 | 10 | 23 | 12 | 11 | 11 | 15 | 18 | 21 | 25 | 13 | 14 | 11 | 12 | 16 | 14,17 | 14 | 12 |
| Lianyungang069 | 21 | 12 | 19 | 28 | 15 | 10 | 21 | 12 | 11 | 10 | 15 | 17 | 19 | 23 | 12 | 12 | 11 | 12 | 18 | 12,16 | 16 | 12 |
| Lianyungang070 | 17 | 12 | 19 | 28 | 15 | 10 | 25 | 14 | 11 | 10 | 14 | 18 | 22 | 25 | 12 | 13 | 11 | 12 | 19 | 12,19 | 13 | 13 |
| Lianyungang071 | 19 | 14 | 18 | 31 | 15 | 10 | 25 | 12 | 10 | 10 | 14 | 18 | 19 | 23 | 12 | 14 | 11 | 13 | 15 | 13,13 | 16 | 12 |
|  |  |  |  |  |  |  |  |  |  |  |  |  |  |  |  |  |  |  |  |  |  |  |
| Yancheng001 | 19 | 12 | 19 | 27 | 15 | 10 | 25 | 13 | 12 | 10 | 15 | 16 | 21 | 24 | 13 | 13 | 12 | 13 | 17 | 14,17 | 15 | 12 |
| Yancheng002 | 20 | 12 | 19 | 27 | 15 | 11 | 25 | 14 | 11 | 10 | 14 | 18 | 21 | 25 | 13 | 13 | 10 | 12 | 19 | 12,18 | 14 | 11 |
| Yancheng003 | 18 | 13 | 20 | 29 | 16 | 10 | 23 | 13 | 10 | 10 | 15 | 18 | 21 | 23 | 12 | 13 | 11 | 12 | 19 | 12,21 | 13 | 12 |
| Yancheng004 | 10 | 12 | 19 | 28 | 13 | 9 | 24 | 12 | 12 | 12 | 14 | 20 | 22 | 24 | 12 | 14 | 10 | 14 | 16 | 15,22 | 15 | 10 |
| Yancheng005 | 18 | 12 | 19 | 29 | 16 | 10 | 25 | 13 | 12 | 10 | 15 | 15 | 21 | 25 | 11 | 13 | 11 | 12 | 16 | 12,20 | 17 | 11 |
| Yancheng006 | 20 | 12 | 19 | 30 | 15 | 10 | 22 | 12 | 11 | 8 | 15 | 16 | 19 | 23 | 13 | 12 | 10 | 13 | 19 | 12,13 | 15 | 12 |
| Yancheng007 | 18 | 12 | 20 | 28 | 15 | 10 | 26 | 12 | 12 | 10 | 14 | 22 | 20 | 24 | 11 | 10 | 9 | 12 | 20 | 17,17 | 14 | 12 |
| Yancheng008 | 18 | 12 | 18 | 29 | 15 | 11 | 26 | 12 | 11 | 10 | 14 | 18 | 19 | 23 | 11 | 14 | 11 | 13 | 15 | 13,13 | 16 | 12 |
| Yancheng009 | 19 | 13 | 20 | 30 | 15 | 10 | 27 | 12 | 12 | 10 | 14 | 16 | 22 | 23 | 11 | 11 | 8 | 15 | 15 | 10,11 | 15 | 11 |
| Yancheng010 | 18 | 12 | 19 | 28 | 13 | 10 | 22 | 15 | 12 | 10 | 15 | 19 | 21 | 25 | 13 | 13 | 9 | 14 | 18 | 12,12 | 14 | 12 |
| Yancheng011 | 22 | 13 | 19 | 31 | 15 | 10 | 22 | 12 | 11 | 10 | 15 | 16 | 19 | 24 | 11 | 12 | 11 | 12 | 17 | 12,13 | 15 | 12 |
| Yancheng012 | 17 | 14 | 19 | 29 | 16 | 10 | 23 | 13 | 12 | 10 | 15 | 18 | 22 | 22 | 11 | 14 | 10 | 12 | 17 | 11,11 | 16 | 12 |
| Yancheng013 | 17 | 13 | 20 | 31 | 16 | 11 | 24 | 12 | 13 | 10 | 14 | 16 | 21 | 24 | 11 | 11 | 9 | 13 | 17 | 11,17 | 15 | 11 |
| Yancheng014 | 19 | 12 | 19 | 29 | 13 | 11 | 23 | 12 | 11 | 11 | 15 | 18 | 22 | 24 | 12 | 14 | 11 | 13 | 17 | 13,20 | 16 | 12 |
| Yancheng015 | 17 | 13 | 20 | 31 | 15 | 10 | 23 | 13 | 12 | 11 | 15 | 19 | 23 | 24 | 10 | 11 | 10 | 13 | 16 | 11,12 | 16 | 13 |
| Yancheng016 | 17 | 12 | 20 | 28 | 14 | 10 | 23 | 13 | 11 | 11 | 15 | 20 | 21 | 23 | 12 | 14 | 10 | 12 | 18 | 13,20 | 15 | 12 |
| Yancheng017 | 20 | 12 | 19 | 30 | 14 | 10 | 24 | 12 | 12 | 10 | 15 | 15 | 19 | 23 | 11 | 12 | 11 | 12 | 17 | 12,12 | 15 | 12 |
| Yancheng018 | 18 | 13 | 18 | 29 | 15 | 10 | 23 | 14 | 10 | 10 | 14 | 20 | 22 | 23 | 13 | 13 | 11 | 14 | 17 | 12,20 | 15 | 11 |
| Yancheng019 | 18 | 12 | 19 | 29 | 14 | 10 | 22 | 14 | 12 | 10 | 15 | 16 | 19 | 22 | 11 | 12 | 11 | 12 | 21 | 12,17 | 15 | 12 |
| Yancheng020 | 18 | 12 | 20 | 28 | 17 | 10 | 26 | 13 | 11 | 10 | 14 | 19 | 23 | 25 | 11 | 13 | 10 | 12 | 16 | 14,17 | 14 | 12 |
| Yancheng021 | 19 | 14 | 19 | 30 | 16 | 10 | 24 | 13 | 12 | 10 | 14 | 20 | 21 | 24 | 12 | 13 | 11 | 12 | 18 | 13,19 | 15 | 11 |
| Yancheng022 | 17 | 13 | 18 | 30 | 16 | 10 | 23 | 12 | 10 | 10 | 14 | 19 | 21 | 25 | 12 | 13 | 12 | 14 | 18 | 13,19 | 16 | 11 |
| Yancheng023 | 19 | 12 | 19 | 28 | 17 | 10 | 25 | 14 | 11 | 10 | 14 | 16 | 20 | 25 | 12 | 13 | 10 | 12 | 17 | 12,20 | 14 | 12 |
| Yancheng024 | 17 | 14 | 20 | 29 | 14 | 10 | 23 | 12 | 11 | 10 | 15 | 16 | 20 | 25 | 11 | 11 | 11 | 13 | 16 | 13,18 | 14 | 12 |
| Yancheng025 | 18 | 12 | 20 | 26 | 14 | 10 | 23 | 13 | 11 | 10 | 15 | 17 | 20 | 24 | 13 | 14 | 11 | 12 | 17 | 13,21 | 15 | 12 |
| Yancheng026 | 18 | 12 | 18 | 27 | 15 | 11 | 25 | 12 | 11 | 10 | 14 | 18 | 19 | 23 | 13 | 15 | 11 | 13 | 15 | 13,13 | 17 | 12 |
| Yancheng027 | 18 | 12 | 20 | 27 | 14 | 10 | 23 | 13 | 11 | 11 | 14 | 19 | 20 | 23 | 11 | 14 | 11 | 12 | 17 | 13,20 | 15 | 12 |
| Yancheng028 | 17 | 12 | 19 | 28 | 15 | 10 | 24 | 13 | 11 | 10 | 14 | 17 | 23 | 25 | 11 | 13 | 11 | 12 | 18 | 12,18 | 14 | 11 |
| Yancheng029 | 19 | 12 | 19 | 30 | 15 | 10 | 22 | 12 | 11 | 10 | 14 | 16 | 20 | 23 | 13 | 12 | 12 | 12 | 18 | 12,16 | 15 | 11 |
| Yancheng030 | 21 | 13 | 21 | 29 | 17 | 10 | 24 | 12 | 11 | 9 | 14 | 17 | 22 | 24 | 12 | 13 | 10 | 12 | 19 | 12,20 | 13 | 12 |
| Yancheng031 | 18 | 12 | 19 | 29 | 14 | 11 | 22 | 13 | 11 | 10 | 16 | 18 | 20 | 23 | 12 | 12 | 11 | 12 | 19 | 12,16 | 16 | 12 |
| Yancheng032 | 18 | 14 | 21 | 29 | 14 | 10 | 24 | 12 | 13 | 10 | 14 | 15 | 21 | 23 | 14 | 11 | 9 | 13 | 17 | 18,18 | 16 | 11 |
| Yancheng033 | 19 | 14 | 18 | 31 | 15 | 10 | 23 | 11 | 12 | 10 | 14 | 21 | 21 | 22 | 11 | 14 | 10 | 13 | 19 | 11,14 | 15 | 11 |
| Yancheng034 | 17 | 14 | 19 | 28 | 15 | 10 | 23 | 13 | 11 | 10 | 14 | 19 | 22 | 23 | 11 | 12 | 12 | 14 | 17 | 15,17 | 15 | 13 |
| Yancheng035 | 19 | 13 | 18 | 29 | 15 | 11 | 25 | 12 | 11 | 10 | 14 | 18 | 19 | 23 | 11 | 14 | 11 | 13 | 15 | 13,13 | 15 | 12 |
| Yancheng036 | 19 | 12 | 19 | 25 | 16 | 9 | 26 | 11 | 11 | 10 | 14 | 17 | 20 | 24 | 12 | 13 | 10 | 12 | 17 | 13,19 | 14 | 12 |
| Yancheng037 | 17 | 12 | 22 | 28 | 16 | 10 | 24 | 14 | 12 | 10 | 14 | 17 | 21 | 23 | 11 | 11 | 9 | 14 | 17 | 11,16 | 16 | 11 |
| Yancheng038 | 17 | 12 | 20 | 28 | 14 | 10 | 23 | 12 | 12 | 11 | 15 | 17 | 20 | 25 | 12 | 14 | 11 | 12 | 16 | 13,20 | 15 | 12 |
| Yancheng039 | 20 | 12 | 18 | 28 | 15 | 10 | 22 | 12 | 11 | 10 | 15 | 18 | 23 | 24 | 11 | 12 | 12 | 12 | 18 | 11,19 | 15 | 11 |
| Yancheng040 | 18 | 12 | 19 | 28 | 15 | 10 | 24 | 13 | 11 | 10 | 15 | 17 | 21 | 23 | 13 | 12 | 11 | 12 | 17 | 12,14 | 17 | 11 |
| Yancheng041 | 17 | 13 | 19 | 28 | 16 | 10 | 26 | 13 | 11 | 10 | 15 | 18 | 24 | 25 | 12 | 13 | 9 | 12 | 21 | 13,18 | 15 | 13 |
| Yancheng042 | 19 | 12 | 20 | 27 | 14 | 10 | 22 | 12 | 12 | 11 | 15 | 18 | 20 | 23 | 12 | 14 | 11 | 12 | 17 | 13,19 | 15 | 12 |
| Yancheng043 | 20 | 12 | 19 | 30 | 15 | 10 | 22 | 12 | 11 | 8 | 15 | 16 | 19 | 23 | 12 | 12 | 10 | 13 | 20 | 12,13 | 15 | 12 |
| Yancheng044 | 19 | 12 | 18 | 29 | 16 | 11 | 25 | 12 | 11 | 10 | 14 | 16 | 20 | 23 | 11 | 14 | 11 | 13 | 15 | 13,13 | 17 | 12 |
| Yancheng045 | 19 | 12 | 20 | 30 | 17 | 10 | 25 | 11 | 11 | 10 | 14 | 18 | 22 | 24 | 11 | 14 | 11 | 12 | 18 | 13,13 | 15 | 12 |
| Yancheng046 | 16 | 12 | 19 | 28 | 16 | 10 | 24 | 13 | 11 | 10 | 14 | 18 | 21 | 23 | 11 | 13 | 10 | 12 | 17 | 12,20 | 14 | 12 |
| Yancheng047 | 17 | 12 | 20 | 28 | 14 | 10 | 24 | 12 | 11 | 10 | 15 | 18 | 20 | 24 | 12 | 14 | 11 | 12 | 16 | 13,19 | 15 | 13 |
| Yancheng048 | 18 | 13 | 20 | 29 | 17 | 11 | 24 | 14 | 11 | 10 | 14 | 18 | 22 | 23 | 12 | 13 | 10 | 12 | 18 | 12,20 | 15 | 11 |
| Yancheng049 | 17 | 13 | 18 | 28 | 15 | 10 | 25 | 12 | 10 | 10 | 14 | 18 | 22 | 25 | 11 | 13 | 13 | 14 | 17 | 13,18 | 16 | 10 |
| Yancheng050 | 19 | 12 | 20 | 29 | 16 | 11 | 25 | 12 | 11 | 9 | 14 | 17 | 21 | 25 | 12 | 13 | 10 | 12 | 18 | 12,22 | 14 | 12 |
| Yancheng051 | 20 | 12 | 20 | 27 | 14 | 11 | 23 | 12 | 12 | 11 | 15 | 20 | 20 | 24 | 11 | 14 | 11 | 12 | 18 | 13,18 | 15 | 11 |
| Yancheng052 | 16 | 13 | 18 | 28 | 15 | 10 | 23 | 12 | 10 | 10 | 14 | 17 | 21 | 23 | 11 | 15 | 10 | 14 | 17 | 12,19 | 15 | 12 |
| Yancheng053 | 15 | 12 | 21 | 30 | 14 | 11 | 23 | 11 | 12 | 10 | 14 | 18 | 22 | 25 | 10 | 14 | 11 | 13 | 16 | 9,12 | 15 | 12 |
| Yancheng054 | 18 | 13 | 22 | 29 | 16 | 10 | 26 | 13 | 13 | 10 | 14 | 17 | 22 | 23 | 12 | 11 | 9 | 14 | 17 | 11,18 | 16 | 11 |
| Yancheng055 | 16 | 12 | 20 | 28 | 16 | 11 | 25 | 12 | 11 | 10 | 14 | 20 | 23 | 25 | 12 | 13 | 10 | 12 | 16 | 14,18 | 14 | 12 |
| Yancheng056 | 17 | 12 | 18 | 28 | 15 | 11 | 26 | 12 | 11 | 10 | 14 | 18 | 19 | 23 | 12 | 14 | 11 | 13 | 15 | 13,13 | 17 | 12 |
| Yancheng057 | 19 | 12 | 20 | 27 | 14 | 10 | 23 | 12 | 11 | 11 | 15 | 20 | 21 | 24 | 12 | 14 | 11 | 12 | 18 | 11,18 | 15 | 12 |
| Yancheng058 | 20 | 13 | 19 | 31 | 15 | 10 | 23 | 13 | 11 | 10 | 14 | 16 | 21 | 24 | 11 | 13 | 12 | 13 | 17 | 12,19 | 15 | 11 |
| Yancheng059 | 17 | 13 | 20 | 29 | 16 | 10 | 24 | 15 | 14 | 10 | 14 | 16 | 22 | 23 | 12 | 11 | 9 | 15 | 17 | 11,18 | 17 | 12 |
| Yancheng060 | 17 | 12 | 20 | 28 | 16 | 10 | 27 | 12 | 11 | 10 | 14 | 18 | 21 | 23 | 11 | 13 | 11 | 12 | 19 | 13,13 | 15 | 12 |
| Yancheng061 | 19 | 12 | 19 | 28 | 15 | 10 | 22 | 12 | 11 | 10 | 15 | 17 | 19 | 23 | 13 | 12 | 11 | 12 | 18 | 13,16 | 15 | 12 |
| Yancheng062 | 17 | 12 | 20 | 28 | 14 | 10 | 22 | 12 | 12 | 11 | 15 | 17 | 20 | 24 | 13 | 14 | 11 | 12 | 17 | 13,18 | 17 | 13 |
|  |  |  |  |  |  |  |  |  |  |  |  |  |  |  |  |  |  |  |  |  |  |  |
| Huai'an001 | 20 | 14 | 19 | 30 | 17 | 10 | 24 | 12 | 11 | 10 | 14 | 19 | 22 | 25 | 12 | 13 | 11 | 12 | 18 | 12,19 | 14 | 11 |
| Huai'an002 | 17 | 13 | 19 | 28 | 16 | 10 | 23 | 14 | 11 | 10 | 16 | 18 | 20 | 22 | 11 | 14 | 10 | 12 | 17 | 11,12 | 16 | 12 |
| Huai'an003 | 18 | 12 | 20 | 27 | 14 | 10 | 23 | 13 | 11 | 11 | 14 | 18 | 21 | 23 | 11 | 14 | 10 | 12 | 17 | 14,19 | 15 | 12 |
| Huai'an004 | 18 | 13 | 21 | 30 | 17 | 11 | 24 | 13 | 11 | 10 | 14 | 17 | 21 | 23 | 12 | 11 | 9 | 15 | 16 | 11,19 | 17 | 11 |
| Huai'an005 | 17 | 14 | 21 | 30 | 14 | 10 | 24 | 12 | 12 | 10 | 14 | 17 | 21 | 23 | 11 | 11 | 8 | 14 | 15 | 11,19 | 15 | 11 |
| Huai'an006 | 16 | 12 | 17 | 29 | 15 | 10 | 24 | 12 | 10 | 10 | 14 | 20 | 21 | 23 | 12 | 12 | 12 | 14 | 14 | 12,14 | 16 | 11 |
| Huai'an007 | 20 | 12 | 19 | 27 | 15 | 10 | 21 | 12 | 11 | 10 | 14 | 16 | 19 | 23 | 12 | 12 | 11 | 12 | 19 | 12,17 | 16 | 12 |
| Huai'an008 | 18 | 13 | 20 | 31 | 16 | 11 | 23 | 11 | 12 | 11 | 14 | 18 | 23 | 25 | 11 | 11 | 10 | 13 | 15 | 11,14 | 15 | 12 |
| Huai'an009 | 21 | 12 | 19 | 29 | 15 | 11 | 22 | 12 | 11 | 10 | 16 | 16 | 19 | 24 | 14 | 12 | 11 | 12 | 19 | 12,16 | 15 | 12 |
| Huai'an010 | 18 | 13 | 21 | 30 | 16 | 11 | 24 | 13 | 11 | 10 | 14 | 17 | 21 | 23 | 12 | 11 | 9 | 15 | 17 | 11,17 | 16 | 11 |
| Huai'an011 | 18 | 12 | 19 | 28 | 15 | 10 | 22 | 13 | 11 | 10 | 15 | 17 | 20 | 23 | 12 | 14 | 13 | 12 | 16 | 12,17 | 15 | 12 |
| Huai'an012 | 18 | 13 | 22 | 31 | 15 | 11 | 27 | 12 | 12 | 10 | 14 | 16 | 21 | 23 | 11 | 11 | 8 | 15 | 15 | 11,12 | 15 | 11 |
| Huai'an013 | 20 | 14 | 21 | 30 | 15 | 10 | 25 | 12 | 13 | 10 | 14 | 16 | 21 | 23 | 11 | 11 | 10 | 15 | 15 | 11,19 | 16 | 11 |
| Huai'an014 | 17 | 12 | 20 | 27 | 14 | 11 | 21 | 12 | 11 | 11 | 15 | 18 | 20 | 24 | 12 | 14 | 11 | 12 | 16 | 14,16 | 15 | 12 |
| Huai'an015 | 17 | 12 | 20 | 27 | 14 | 10 | 23 | 12 | 12 | 11 | 15 | 18 | 20 | 25 | 13 | 14 | 11 | 12 | 18 | 13,19 | 15 | 11 |
| Huai'an016 | 20 | 12 | 19 | 27 | 15 | 10 | 23 | 13 | 12 | 10 | 15 | 16 | 20 | 23 | 12 | 12 | 10 | 12 | 19 | 12,16 | 15 | 12 |
| Huai'an017 | 19 | 12 | 18 | 26 | 15 | 11 | 26 | 13 | 11 | 10 | 14 | 19 | 19 | 23 | 11 | 14 | 11 | 13 | 15 | 13,14 | 17 | 12 |
| Huai'an018 | 15 | 12 | 18 | 31 | 15 | 10 | 25 | 12 | 11 | 10 | 14 | 18 | 20 | 23 | 12 | 14 | 11 | 13 | 15 | 12,13 | 15 | 12 |
| Huai'an019 | 19 | 12 | 18 | 29 | 16 | 11 | 25 | 12 | 11 | 10 | 14 | 19 | 19 | 23 | 11 | 14 | 11 | 13 | 15 | 13,13 | 16 | 12 |
| Huai'an020 | 17 | 12 | 19 | 28 | 15 | 10 | 25 | 12 | 11 | 10 | 14 | 18 | 20 | 22 | 11 | 14 | 12 | 13 | 15 | 13,14 | 16 | 12 |
| Huai'an021 | 17 | 14 | 20 | 30 | 16 | 10 | 24 | 12 | 12 | 10 | 14 | 16 | 20 | 24 | 11 | 11 | 9 | 13 | 18 | 11,18 | 15 | 12 |
| Huai'an022 | 18 | 12 | 20 | 30 | 15 | 10 | 25 | 13 | 11 | 10 | 15 | 18 | 21 | 25 | 11 | 13 | 11 | 12 | 15 | 13,21 | 14 | 12 |
| Huai'an023 | 17 | 13 | 18 | 31 | 15 | 10 | 24 | 12 | 11 | 10 | 14 | 17 | 20 | 24 | 11 | 14 | 11 | 13 | 15 | 13,13 | 16 | 10 |
| Huai'an024 | 18 | 14 | 18 | 31 | 15 | 10 | 23 | 12 | 11 | 10 | 14 | 20 | 21 | 24 | 11 | 13 | 11 | 14 | 15 | 12,17 | 15 | 11 |
| Huai'an025 | 18 | 13 | 18 | 29 | 15 | 11 | 24 | 13 | 11 | 10 | 14 | 19 | 21 | 23 | 12 | 14 | 11 | 13 | 14 | 12,13 | 15 | 11 |
| Huai'an026 | 17 | 12 | 18 | 28 | 15 | 10 | 24 | 12 | 11 | 10 | 14 | 18 | 20 | 23 | 11 | 14 | 11 | 12 | 15 | 13,13 | 16 | 11 |
| Huai'an027 | 19 | 12 | 20 | 28 | 15 | 10 | 22 | 12 | 12 | 10 | 14 | 16 | 21 | 22 | 12 | 12 | 10 | 13 | 17 | 12,17 | 15 | 12 |
| Huai'an028 | 18 | 12 | 20 | 28 | 15 | 10 | 22 | 12 | 11 | 11 | 15 | 19 | 23 | 23 | 11 | 12 | 11 | 12 | 19 | 11,12 | 15 | 12 |
| Huai'an029 | 20 | 12 | 19 | 28 | 15 | 10 | 24 | 12 | 12 | 10 | 15 | 15 | 20 | 23 | 12 | 12 | 11 | 12 | 19 | 12,17 | 15 | 11 |
| Huai'an030 | 19 | 12 | 20 | 28 | 15 | 10 | 24 | 12 | 12 | 11 | 15 | 18 | 20 | 25 | 13 | 14 | 11 | 12 | 18 | 14,18 | 15 | 12 |
| Huai'an031 | 21 | 12 | 19 | 27 | 15 | 10 | 24 | 12 | 12 | 10 | 15 | 17 | 21 | 24 | 12 | 13 | 11 | 14 | 18 | 14,17 | 16 | 12 |
| Huai'an032 | 19 | 13 | 19 | 30 | 15 | 10 | 23 | 12 | 11 | 10 | 15 | 19 | 21 | 22 | 12 | 13 | 10 | 12 | 17 | 11,11 | 16 | 11 |
| Huai'an033 | 19 | 14 | 19 | 29 | 13 | 9 | 23 | 12 | 12 | 11 | 14 | 21 | 22 | 25 | 11 | 14 | 10 | 14 | 16 | 15,23 | 17 | 10 |
| Huai'an034 | 19 | 13 | 18 | 29 | 16 | 10 | 25 | 12 | 10 | 10 | 14 | 19 | 19 | 23 | 12 | 14 | 12 | 13 | 16 | 13,13 | 17 | 10 |
| Huai'an035 | 21 | 12 | 18 | 29 | 15 | 10 | 25 | 11 | 10 | 10 | 14 | 17 | 19 | 25 | 11 | 14 | 11 | 13 | 15 | 13,13 | 17 | 13 |
| Huai'an036 | 20 | 12 | 20 | 28 | 15 | 10 | 23 | 13 | 11 | 11 | 15 | 18 | 22 | 23 | 12 | 14 | 11 | 12 | 16 | 14,17 | 15 | 12 |
| Huai'an037 | 18 | 12 | 19 | 28 | 15 | 10 | 22 | 12 | 11 | 10 | 15 | 17 | 20 | 23 | 12 | 12 | 11 | 12 | 18 | 13,16 | 16 | 12 |
| Huai'an038 | 18 | 13 | 19 | 29 | 14 | 10 | 22 | 12 | 12 | 10 | 14 | 17 | 21 | 23 | 11 | 14 | 12 | 13 | 17 | 12,13 | 15 | 12 |
| Huai'an039 | 18 | 13 | 21 | 30 | 16 | 10 | 23 | 13 | 14 | 10 | 14 | 17 | 22 | 23 | 11 | 11 | 9 | 15 | 16 | 11,17 | 15 | 11 |
| Huai'an040 | 18 | 12 | 18 | 28 | 15 | 11 | 24 | 13 | 11 | 10 | 14 | 19 | 21 | 23 | 12 | 14 | 11 | 13 | 15 | 13,13 | 15 | 12 |
| Huai'an041 | 20 | 12 | 19 | 29 | 15 | 10 | 25 | 12 | 11 | 10 | 14 | 19 | 21 | 23 | 12 | 14 | 11 | 12 | 16 | 13,13 | 15 | 12 |
| Huai'an042 | 19 | 14 | 18 | 31 | 16 | 10 | 24 | 12 | 12 | 13 | 14 | 17 | 20 | 23 | 12 | 13 | 12 | 13 | 18 | 10,18 | 15 | 11 |
| Huai'an043 | 17 | 13 | 19 | 28 | 15 | 11 | 22 | 13 | 11 | 10 | 14 | 17 | 21 | 22 | 12 | 12 | 12 | 12 | 17 | 11,17 | 16 | 12 |
| Huai'an044 | 18 | 13 | 20 | 29 | 16 | 9 | 23 | 11 | 11 | 11 | 15 | 19 | 20 | 24 | 11 | 15 | 12 | 12 | 18 | 13,19 | 15 | 12 |
| Huai'an045 | 17 | 12 | 19 | 28 | 13 | 10 | 24 | 12 | 11 | 10 | 14 | 19 | 23 | 25 | 13 | 14 | 10 | 12 | 17 | 12,18 | 14 | 12 |
| Huai'an046 | 17 | 12 | 20 | 30 | 17 | 10 | 24 | 13 | 11 | 10 | 14 | 17 | 23 | 25 | 12 | 13 | 10 | 12 | 21 | 14,19 | 14 | 12 |
| Huai'an047 | 18 | 12 | 20 | 28 | 14 | 10 | 23 | 12 | 11 | 11 | 15 | 18 | 21 | 23 | 11 | 14 | 11 | 12 | 17 | 13,17 | 16 | 12 |
| Huai'an048 | 19 | 13 | 17 | 29 | 15 | 10 | 22 | 10 | 11 | 10 | 14 | 18 | 20 | 23 | 13 | 13 | 11 | 14 | 16 | 10,18 | 15 | 11 |
| Huai'an049 | 19 | 12 | 20 | 28 | 14 | 10 | 23 | 13 | 11 | 11 | 14 | 18 | 20 | 23 | 11 | 15 | 11 | 12 | 17 | 13,19 | 15 | 12 |
| Huai'an050 | 18 | 12 | 18 | 26 | 16 | 11 | 21 | 13 | 12 | 10 | 14 | 18 | 20 | 23 | 12 | 14 | 11 | 13 | 15 | 12,13 | 18 | 11 |
| Huai'an051 | 18 | 12 | 21 | 28 | 16 | 10 | 23 | 12 | 11 | 10 | 15 | 21 | 22 | 23 | 12 | 13 | 13 | 12 | 16 | 12,22 | 15 | 12 |
| Huai'an052 | 19 | 12 | 19 | 28 | 15 | 9 | 26 | 13 | 11 | 10 | 14 | 17 | 22 | 25 | 11 | 13 | 10 | 12 | 17 | 12,21 | 14 | 12 |
| Huai'an053 | 18 | 12 | 20 | 28 | 16 | 10 | 24 | 13 | 11 | 9 | 14 | 19 | 21 | 25 | 12 | 13 | 10 | 12 | 19 | 12,18 | 14 | 12 |
| Huai'an054 | 16 | 12 | 18 | 29 | 15 | 10 | 26 | 12 | 11 | 10 | 14 | 18 | 20 | 22 | 10 | 14 | 11 | 13 | 14 | 13,14 | 14 | 12 |
| Huai'an055 | 18 | 14 | 20 | 29 | 14 | 11 | 23 | 13 | 10 | 10 | 14 | 19 | 21 | 25 | 13 | 13 | 12 | 12 | 16 | 13,18 | 16 | 11 |
|  |  |  |  |  |  |  |  |  |  |  |  |  |  |  |  |  |  |  |  |  |  |  |
| Soochow001 | 18 | 12 | 19 | 29 | 13 | 10 | 22 | 12 | 12 | 10 | 15 | 19 | 21 | 25 | 11 | 13 | 9 | 13 | 19 | 12,12 | 15 | 12 |
| Soochow002 | 18 | 13 | 19 | 31 | 17 | 11 | 24 | 12 | 11 | 10 | 15 | 20 | 22 | 23 | 11 | 13 | 10 | 13 | 20 | 12,19 | 15 | 12 |
| Soochow003 | 17 | 13 | 21 | 29 | 15 | 10 | 24 | 14 | 11 | 10 | 14 | 16 | 21 | 23 | 11 | 11 | 9 | 14 | 16 | 11,18 | 16 | 11 |
| Soochow004 | 16 | 13 | 19 | 30 | 15 | 10 | 23 | 12 | 11 | 11 | 14 | 17 | 21 | 25 | 11 | 13 | 10 | 12 | 17 | 13,19 | 16 | 12 |
| Soochow005 | 18 | 13 | 18 | 29 | 15 | 10 | 24 | 12 | 10 | 10 | 14 | 18 | 20 | 24 | 11 | 13 | 12 | 14 | 17 | 13,18 | 16 | 10 |
| Soochow006 | 18 | 14 | 21 | 29 | 16 | 10 | 24 | 13 | 12 | 10 | 14 | 16 | 21 | 23 | 12 | 11 | 9 | 14 | 17 | 11,21 | 14 | 11 |
| Soochow007 | 18 | 12 | 19 | 28 | 16 | 11 | 25 | 12 | 11 | 10 | 14 | 18 | 19 | 23 | 11 | 14 | 11 | 13 | 15 | 12,13 | 17 | 13 |
| Soochow008 | 18 | 12 | 18 | 29 | 15 | 11 | 25 | 13 | 11 | 10 | 14 | 18 | 19 | 23 | 11 | 14 | 11 | 13 | 15 | 13,14 | 18 | 13 |
| Soochow009 | 18 | 12 | 18 | 30 | 15 | 11 | 25 | 12 | 11 | 10 | 14 | 18 | 19 | 23 | 12 | 14 | 11 | 13 | 15 | 13,13 | 17 | 12 |
| Soochow010 | 16 | 12 | 20 | 28 | 16 | 10 | 24 | 12 | 11 | 10 | 14 | 18 | 22 | 25 | 12 | 13 | 10 | 12 | 20 | 14,18 | 14 | 12 |
| Soochow011 | 22 | 12 | 20 | 28 | 15 | 10 | 24 | 13 | 11 | 10 | 15 | 17 | 21 | 23 | 14 | 12 | 10 | 13 | 20 | 12,16 | 16 | 11 |
| Soochow012 | 18 | 12 | 20 | 31 | 14 | 10 | 24 | 12 | 11 | 11 | 15 | 18 | 21 | 24 | 11 | 14 | 11 | 12 | 17 | 15,18 | 16 | 13 |
| Soochow013 | 18 | 14 | 18 | 31 | 16 | 10 | 23 | 12 | 11 | 10 | 14 | 22 | 21 | 24 | 11 | 13 | 11 | 14 | 15 | 13,16 | 15 | 13 |
| Soochow014 | 18 | 12 | 20 | 29 | 15 | 10 | 24 | 13 | 11 | 10 | 15 | 18 | 21 | 24 | 11 | 13 | 11 | 12 | 16 | 13,21 | 14 | 14 |
| Soochow015 | 14 | 14 | 18 | 32 | 15 | 10 | 23 | 12 | 12 | 10 | 14 | 17 | 22 | 23 | 11 | 14 | 10 | 14 | 15 | 11,12 | 17 | 12 |
| Soochow016 | 17 | 12 | 20 | 30 | 15 | 10 | 24 | 13 | 11 | 10 | 15 | 19 | 21 | 25 | 11 | 13 | 11 | 12 | 16 | 13,21 | 14 | 13 |
| Soochow017 | 17 | 12 | 19 | 28 | 15 | 10 | 23 | 13 | 12 | 10 | 14 | 17 | 23 | 25 | 11 | 13 | 11 | 12 | 19 | 12,18 | 15 | 11 |
| Soochow018 | 15 | 14 | 19 | 31 | 14 | 10 | 22 | 12 | 12 | 10 | 14 | 18 | 23 | 23 | 11 | 14 | 10 | 13 | 16 | 11,12 | 18 | 12 |
| Soochow019 | 17 | 12 | 18 | 28 | 15 | 11 | 25 | 12 | 11 | 10 | 14 | 17 | 19 | 23 | 11 | 14 | 11 | 13 | 15 | 13,13 | 15 | 11 |
| Soochow020 | 14 | 14 | 19 | 31 | 14 | 10 | 22 | 12 | 12 | 10 | 14 | 18 | 23 | 23 | 11 | 14 | 10 | 13 | 16 | 11,12 | 17 | 11 |
| Soochow021 | 19 | 12 | 18 | 27 | 16 | 11 | 21 | 12 | 12 | 10 | 14 | 18 | 20 | 23 | 12 | 14 | 11 | 13 | 15 | 12,13 | 18 | 12 |
| Soochow022 | 18 | 13 | 21 | 29 | 15 | 10 | 26 | 12 | 11 | 10 | 14 | 16 | 21 | 23 | 11 | 11 | 9 | 15 | 17 | 10,19 | 15 | 12 |
| Soochow023 | 17 | 14 | 18 | 31 | 14 | 11 | 22 | 12 | 12 | 10 | 14 | 18 | 20 | 23 | 10 | 14 | 11 | 13 | 18 | 12,12 | 14 | 11 |
| Soochow024 | 22 | 12 | 19 | 29 | 15 | 11 | 23 | 12 | 11 | 10 | 15 | 16 | 19 | 24 | 13 | 12 | 11 | 12 | 21 | 12,16 | 15 | 12 |
| Soochow025 | 19 | 12 | 20 | 28 | 15 | 10 | 22 | 13 | 11 | 11 | 14 | 19 | 22 | 23 | 11 | 12 | 11 | 12 | 18 | 12,12 | 15 | 12 |
| Soochow026 | 18 | 12 | 19 | 28 | 13 | 10 | 22 | 12 | 11 | 10 | 15 | 16 | 21 | 25 | 11 | 12 | 10 | 12 | 19 | 12,16 | 14 | 12 |
| Soochow027 | 20 | 13 | 21 | 30 | 16 | 10 | 24 | 12 | 13 | 10 | 14 | 16 | 22 | 23 | 11 | 11 | 9 | 14 | 12 | 11,16 | 15 | 11 |
| Soochow028 | 19 | 12 | 19 | 27 | 15 | 10 | 22 | 12 | 10 | 10 | 15 | 16 | 20 | 24 | 11 | 12 | 10 | 12 | 16 | 12,18 | 15 | 11 |
| Soochow029 | 20 | 13 | 19 | 28 | 17 | 10 | 24 | 12 | 11 | 10 | 14 | 18 | 24 | 25 | 12 | 13 | 10 | 12 | 18 | 13,19 | 14 | 12 |
| Soochow030 | 17 | 12 | 19 | 29 | 16 | 10 | 24 | 13 | 11 | 10 | 14 | 18 | 22 | 25 | 10 | 13 | 11 | 12 | 20 | 12,19 | 14 | 11 |
| Soochow031 | 18 | 13 | 21 | 30 | 16 | 10 | 24 | 12 | 11 | 10 | 15 | 17 | 22 | 24 | 12 | 13 | 11 | 12 | 19 | 12,22 | 13 | 12 |
| Soochow032 | 17 | 14 | 19 | 32 | 15 | 11 | 23 | 13 | 12 | 10 | 14 | 16 | 24 | 23 | 13 | 13 | 12 | 12 | 16 | 12,19 | 14 | 11 |
| Soochow033 | 20 | 12 | 19 | 29 | 15 | 10 | 21 | 12 | 11 | 10 | 14 | 16 | 19 | 23 | 13 | 12 | 11 | 12 | 18 | 12,17 | 16 | 12 |
| Soochow034 | 18 | 12 | 18 | 29 | 16 | 11 | 24 | 12 | 11 | 10 | 14 | 17 | 19 | 23 | 11 | 14 | 11 | 13 | 14 | 13,13 | 17 | 12 |
| Soochow035 | 18 | 12 | 21 | 28 | 15 | 10 | 26 | 11 | 12 | 10 | 14 | 16 | 21 | 23 | 10 | 11 | 8 | 14 | 16 | 12,18 | 15 | 11 |
| Soochow036 | 17 | 12 | 18 | 30 | 16 | 11 | 26 | 12 | 12 | 9 | 14 | 18 | 19 | 23 | 11 | 14 | 11 | 13 | 15 | 13,13 | 17 | 11 |
| Soochow037 | 17 | 12 | 18 | 29 | 15 | 10 | 25 | 13 | 11 | 10 | 14 | 18 | 19 | 23 | 9 | 14 | 11 | 13 | 16 | 13,15 | 17 | 11 |
| Soochow038 | 21 | 12 | 19 | 30 | 15 | 10 | 24 | 12 | 11 | 10 | 15 | 16 | 20 | 23 | 12 | 12 | 12 | 12 | 18 | 12,17 | 15 | 12 |
| Soochow039 | 18 | 12 | 18 | 28 | 15 | 10 | 25 | 12 | 11 | 10 | 14 | 19 | 19 | 23 | 11 | 14 | 11 | 13 | 15 | 13,13 | 17 | 12 |
| Soochow040 | 19 | 12 | 19 | 28 | 15 | 10 | 23 | 12 | 11 | 10 | 14 | 19 | 21 | 25 | 12 | 13 | 10 | 12 | 19 | 12,18 | 14 | 11 |
| Soochow041 | 18 | 12 | 20 | 27 | 13 | 10 | 23 | 12 | 11 | 11 | 14 | 18 | 20 | 23 | 13 | 14 | 11 | 12 | 18 | 13,18 | 15 | 12 |
| Soochow042 | 18 | 13 | 18 | 29 | 15 | 10 | 21 | 12 | 12 | 10 | 15 | 18 | 20 | 23 | 11 | 14 | 11 | 12 | 19 | 12,13 | 14 | 10 |
| Soochow043 | 19 | 13 | 18 | 31 | 15 | 11 | 25 | 12 | 11 | 10 | 14 | 18 | 19 | 24 | 11 | 14 | 11 | 13 | 15 | 13,13 | 16 | 12 |
| Soochow044 | 19 | 14 | 18 | 30 | 15 | 10 | 23 | 12 | 10 | 10 | 14 | 16 | 21 | 26 | 11 | 13 | 12 | 14 | 16 | 14,20 | 16 | 11 |
| Soochow045 | 18 | 12 | 18 | 30 | 15 | 10 | 24 | 13 | 12 | 10 | 14 | 17 | 21 | 24 | 12 | 13 | 10 | 12 | 18 | 13,14 | 13 | 12 |
| Soochow046 | 19 | 12 | 19 | 29 | 15 | 10 | 28 | 16 | 11 | 10 | 14 | 17 | 23 | 25 | 12 | 13 | 11 | 13 | 19 | 12,18 | 16 | 12 |
| Soochow047 | 21 | 12 | 19 | 27 | 16 | 11 | 24 | 13 | 11 | 10 | 15 | 17 | 20 | 23 | 13 | 12 | 11 | 13 | 20 | 12,16 | 15 | 11 |
|  |  |  |  |  |  |  |  |  |  |  |  |  |  |  |  |  |  |  |  |  |  |  |
| Nanjing001 | 17 | 13 | 19 | 28 | 17 | 12 | 24 | 12 | 11 | 10 | 14 | 18 | 23 | 25 | 12 | 15 | 10 | 11 | 19 | 13,19 | 14 | 13 |
| Nanjing002 | 17 | 13 | 18 | 28 | 17 | 10 | 24 | 12 | 11 | 10 | 14 | 16 | 22 | 25 | 11 | 13 | 12 | 14 | 16 | 13,18 | 15 | 11 |
| Nanjing003 | 19 | 12 | 20 | 27 | 17 | 10 | 25 | 13 | 11 | 10 | 16 | 18 | 22 | 24 | 12 | 13 | 12 | 12 | 17 | 13,21 | 14 | 11 |
| Nanjing004 | 17 | 13 | 21 | 29 | 15 | 11 | 27 | 13 | 12 | 10 | 14 | 15 | 21 | 23 | 11 | 11 | 8 | 14 | 15 | 11,19 | 15 | 11 |
| Nanjing005 | 17 | 12 | 18 | 29 | 15 | 12 | 27 | 13 | 11 | 10 | 14 | 17 | 19 | 23 | 11 | 14 | 11 | 13 | 16 | 13,13 | 17 | 11 |
| Nanjing006 | 19 | 13 | 20 | 30 | 14 | 10 | 23 | 11 | 11 | 11 | 15 | 17 | 20 | 24 | 12 | 14 | 12 | 12 | 20 | 13,18 | 15 | 12 |
| Nanjing007 | 20 | 12 | 19 | 27 | 16 | 10 | 23 | 12 | 11 | 10 | 15 | 16 | 19 | 23 | 13 | 12 | 11 | 13 | 18 | 12,12 | 15 | 12 |
| Nanjing008 | 18 | 12 | 20 | 30 | 16 | 10 | 24 | 13 | 11 | 10 | 15 | 20 | 21 | 25 | 11 | 13 | 12 | 12 | 17 | 13,20 | 13 | 13 |
| Nanjing009 | 18 | 14 | 20 | 31 | 15 | 10 | 24 | 13 | 11 | 10 | 15 | 19 | 21 | 24 | 12 | 13 | 12 | 13 | 17 | 13,24 | 15 | 12 |
| Nanjing010 | 18 | 14 | 19 | 31 | 15 | 10 | 22 | 13 | 12 | 10 | 14 | 17 | 21 | 22 | 10 | 13 | 11 | 12 | 16 | 12,20 | 15 | 11 |
| Nanjing011 | 19 | 12 | 19 | 30 | 15 | 10 | 22 | 13 | 11 | 10 | 14 | 17 | 19 | 23 | 12 | 12 | 11 | 12 | 18 | 12,17 | 16 | 12 |
| Nanjing012 | 16 | 12 | 20 | 27 | 15 | 9 | 24 | 12 | 12 | 10 | 14 | 19 | 23 | 24 | 11 | 13 | 10 | 12 | 17 | 14,19 | 14 | 10 |
| Nanjing013 | 17 | 13 | 19 | 29 | 15 | 11 | 23 | 12 | 12 | 10 | 14 | 17 | 21 | 23 | 11 | 13 | 11 | 12 | 18 | 12,19 | 15 | 11 |
| Nanjing014 | 18 | 12 | 18 | 29 | 15 | 11 | 25 | 12 | 11 | 10 | 14 | 17 | 19 | 24 | 12 | 14 | 11 | 13 | 15 | 13,13 | 17 | 12 |
| Nanjing015 | 18 | 12 | 20 | 28 | 14 | 10 | 23 | 12 | 11 | 11 | 15 | 18 | 20 | 24 | 12 | 14 | 11 | 12 | 16 | 13,17 | 16 | 12 |
| Nanjing016 | 20 | 12 | 20 | 28 | 15 | 10 | 23 | 12 | 11 | 11 | 15 | 17 | 20 | 24 | 12 | 14 | 11 | 12 | 16 | 13,17 | 14 | 12 |
| Nanjing017 | 23 | 12 | 20 | 28 | 13 | 10 | 23 | 12 | 11 | 10 | 15 | 18 | 19 | 23 | 13 | 12 | 11 | 12 | 18 | 12,16 | 15 | 12 |
| Nanjing018 | 19 | 14 | 19 | 31 | 14 | 10 | 23 | 13 | 12 | 10 | 14 | 17 | 21 | 23 | 10 | 14 | 12 | 13 | 14 | 12,15 | 15 | 11 |
| Nanjing019 | 17 | 12 | 19 | 28 | 15 | 10 | 26 | 12 | 11 | 10 | 14 | 17 | 22 | 26 | 12 | 13 | 10 | 12 | 17 | 12,19 | 16 | 11 |
| Nanjing020 | 18 | 12 | 19 | 28 | 15 | 10 | 23 | 12 | 11 | 11 | 15 | 16 | 22 | 24 | 12 | 12 | 11 | 12 | 15 | 13,17 | 15 | 12 |
| Nanjing021 | 17 | 12 | 20 | 28 | 15 | 10 | 21 | 13 | 11 | 11 | 15 | 19 | 22 | 23 | 11 | 12 | 11 | 12 | 18 | 12,12 | 15 | 12 |
| Nanjing022 | 17 | 13 | 19 | 30 | 14 | 10 | 24 | 13 | 12 | 10 | 14 | 17 | 21 | 23 | 11 | 15 | 12 | 13 | 15 | 12,13 | 15 | 12 |
| Nanjing023 | 20 | 12 | 19 | 29 | 15 | 10 | 22 | 12 | 11 | 10 | 15 | 16 | 19 | 23 | 11 | 12 | 10 | 12 | 17 | 12,13 | 15 | 12 |
| Nanjing024 | 17 | 12 | 18 | 29 | 15 | 11 | 25 | 12 | 11 | 10 | 14 | 19 | 19 | 23 | 11 | 14 | 11 | 13 | 15 | 13,13 | 17 | 12 |
| Nanjing025 | 17 | 13 | 18 | 30 | 15 | 11 | 25 | 13 | 11 | 11 | 14 | 15 | 21 | 23 | 13 | 13 | 12 | 14 | 16 | 12,17 | 16 | 12 |
| Nanjing026 | 19 | 13 | 19 | 28 | 15 | 10 | 23 | 12 | 11 | 10 | 15 | 18 | 21 | 22 | 11 | 14 | 10 | 12 | 16 | 11,11 | 16 | 12 |
| Nanjing027 | 19 | 12 | 18 | 28 | 15 | 10 | 25 | 12 | 11 | 10 | 14 | 18 | 19 | 23 | 11 | 14 | 11 | 13 | 15 | 13,13 | 17 | 12 |
| Nanjing028 | 17 | 12 | 18 | 28 | 15 | 11 | 25 | 11 | 11 | 10 | 14 | 18 | 19 | 23 | 11 | 14 | 11 | 13 | 15 | 13,13 | 17 | 12 |
| Nanjing029 | 19 | 13 | 19 | 29 | 14 | 11 | 21 | 12 | 12 | 11 | 14 | 18 | 24 | 23 | 11 | 14 | 10 | 13 | 15 | 11,12 | 16 | 12 |
| Nanjing030 | 17 | 14 | 19 | 30 | 14 | 10 | 23 | 14 | 13 | 12 | 15 | 20 | 23 | 25 | 14 | 13 | 10 | 12 | 16 | 12,14 | 15 | 12 |
| Nanjing031 | 17 | 13 | 21 | 30 | 14 | 10 | 23 | 12 | 11 | 9 | 15 | 15 | 21 | 22 | 12 | 11 | 10 | 12 | 16 | 13,21 | 15 | 12 |
| Nanjing032 | 19 | 13 | 19 | 30 | 15 | 10 | 23 | 12 | 11 | 10 | 14 | 16 | 22 | 24 | 11 | 13 | 11 | 13 | 17 | 12,20 | 15 | 11 |
| Nanjing033 | 18 | 13 | 19 | 30 | 15 | 10 | 22 | 12 | 12 | 11 | 14 | 18 | 22 | 21 | 11 | 14 | 10 | 13 | 16 | 12,13 | 16 | 13 |
| Nanjing034 | 17 | 12 | 20 | 27 | 16 | 11 | 25 | 13 | 11 | 11 | 13 | 19 | 23 | 24 | 11 | 13 | 10 | 12 | 17 | 14,18 | 14 | 12 |
| Nanjing035 | 19 | 12 | 18 | 28 | 15 | 11 | 25 | 12 | 11 | 10 | 14 | 17 | 19 | 23 | 11 | 14 | 11 | 13 | 15 | 13,13 | 16 | 12 |
| Nanjing036 | 20 | 12 | 20 | 27 | 14 | 10 | 24 | 12 | 11 | 12 | 14 | 18 | 20 | 23 | 12 | 14 | 10 | 12 | 19 | 13,13 | 14 | 12 |
| Nanjing037 | 18 | 13 | 21 | 29 | 16 | 10 | 23 | 13 | 11 | 10 | 15 | 17 | 22 | 24 | 13 | 13 | 11 | 12 | 17 | 12,18 | 13 | 11 |
| Nanjing038 | 16 | 13 | 21 | 29 | 15 | 10 | 22 | 12 | 13 | 10 | 14 | 15 | 20 | 23 | 10 | 11 | 9 | 14 | 15 | 11,19 | 15 | 12 |
| Nanjing039 | 20 | 14 | 19 | 30 | 13 | 9 | 24 | 10 | 11 | 12 | 14 | 20 | 22 | 24 | 10 | 14 | 11 | 14 | 20 | 16,21 | 16 | 10 |
| Nanjing040 | 20 | 12 | 19 | 28 | 14 | 11 | 22 | 12 | 11 | 10 | 15 | 17 | 20 | 23 | 13 | 12 | 11 | 12 | 17 | 12,16 | 16 | 11 |
| Nanjing041 | 21 | 12 | 20 | 28 | 14 | 10 | 23 | 13 | 11 | 11 | 15 | 17 | 20 | 25 | 11 | 14 | 12 | 13 | 18 | 13,17 | 15 | 12 |
|  |  |  |  |  |  |  |  |  |  |  |  |  |  |  |  |  |  |  |  |  |  |  |
| Nantong001 | 18 | 13 | 21 | 28 | 16 | 10 | 24 | 12 | 12 | 10 | 14 | 17 | 21 | 24 | 13 | 11 | 9 | 15 | 17 | 11,19 | 15 | 11 |
| Nantong002 | 19 | 12 | 18 | 29 | 15 | 10 | 25 | 12 | 11 | 10 | 14 | 18 | 19 | 23 | 12 | 14 | 11 | 13 | 15 | 13,13 | 17 | 12 |
| Nantong003 | 18 | 12 | 18 | 28 | 15 | 11 | 25 | 12 | 11 | 10 | 14 | 18 | 19 | 23 | 11 | 14 | 11 | 12 | 15 | 13,13 | 17 | 13 |
| Nantong004 | 14 | 14 | 19 | 30 | 14 | 10 | 22 | 12 | 12 | 10 | 14 | 19 | 23 | 23 | 11 | 12 | 10 | 13 | 15 | 11,12 | 16 | 12 |
| Nantong005 | 17 | 14 | 21 | 30 | 16 | 10 | 24 | 12 | 13 | 10 | 14 | 15 | 21 | 23 | 11 | 11 | 9 | 15 | 17 | 11,17 | 16 | 11 |
| Nantong006 | 20 | 12 | 18 | 27 | 16 | 10 | 21 | 13 | 11 | 10 | 14 | 19 | 20 | 23 | 12 | 14 | 10 | 13 | 15 | 12,13 | 18 | 12 |
| Nantong007 | 19 | 12 | 20 | 27 | 14 | 10 | 25 | 14 | 12 | 11 | 15 | 20 | 20 | 23 | 12 | 14 | 12 | 12 | 20 | 13,18 | 15 | 12 |
| Nantong008 | 19 | 12 | 19 | 27 | 14 | 10 | 22 | 13 | 11 | 10 | 15 | 16 | 19 | 23 | 12 | 12 | 11 | 12 | 18 | 13,16 | 16 | 12 |
| Nantong009 | 19 | 13 | 19 | 29 | 15 | 10 | 23 | 12 | 11 | 10 | 15 | 17 | 21 | 22 | 12 | 13 | 10 | 12 | 18 | 11,11 | 16 | 12 |
| Nantong010 | 18 | 12 | 18 | 28 | 15 | 10 | 24 | 12 | 11 | 10 | 14 | 19 | 19 | 23 | 12 | 14 | 11 | 13 | 15 | 13,13 | 17 | 12 |
| Nantong011 | 21 | 13 | 21 | 30 | 16 | 11 | 24 | 13 | 12 | 10 | 14 | 16 | 21 | 23 | 13 | 11 | 9 | 15 | 18 | 11,18 | 15 | 12 |
| Nantong012 | 18 | 12 | 20 | 28 | 14 | 10 | 24 | 13 | 11 | 11 | 14 | 20 | 20 | 23 | 11 | 14 | 11 | 12 | 17 | 13,19 | 15 | 12 |
| Nantong013 | 18 | 12 | 18 | 29 | 16 | 10 | 25 | 13 | 11 | 10 | 14 | 18 | 19 | 23 | 9 | 14 | 11 | 13 | 15 | 13,13 | 18 | 12 |
| Nantong014 | 20 | 14 | 20 | 31 | 16 | 10 | 28 | 12 | 12 | 10 | 14 | 17 | 22 | 23 | 11 | 11 | 8 | 15 | 16 | 12,19 | 16 | 11 |
| Nantong015 | 19 | 13 | 19 | 28 | 15 | 10 | 22 | 13 | 12 | 10 | 14 | 17 | 21 | 24 | 11 | 15 | 12 | 13 | 16 | 12,13 | 14 | 12 |
| Nantong016 | 17 | 12 | 19 | 29 | 15 | 10 | 22 | 12 | 11 | 10 | 15 | 17 | 21 | 23 | 12 | 12 | 11 | 12 | 19 | 12,12 | 15 | 12 |
| Nantong017 | 17 | 12 | 19 | 29 | 15 | 10 | 22 | 13 | 11 | 10 | 15 | 17 | 19 | 23 | 12 | 12 | 12 | 12 | 19 | 13,18 | 15 | 12 |
| Nantong018 | 14 | 14 | 19 | 31 | 14 | 10 | 22 | 12 | 12 | 10 | 14 | 19 | 23 | 23 | 11 | 12 | 10 | 13 | 15 | 11,12 | 18 | 12 |
| Nantong019 | 18 | 12 | 19 | 28 | 15 | 10 | 23 | 12 | 11 | 10 | 15 | 16 | 21 | 24 | 11 | 12 | 10 | 12 | 17 | 11,12 | 15 | 12 |
| Nantong020 | 19 | 13 | 20 | 28 | 14 | 11 | 23 | 12 | 11 | 11 | 15 | 22 | 20 | 24 | 12 | 14 | 11 | 12 | 19 | 13,18 | 15 | 10 |
| Nantong021 | 18 | 13 | 21 | 29 | 14 | 10 | 23 | 12 | 11 | 11 | 15 | 20 | 20 | 24 | 12 | 14 | 12 | 12 | 17 | 12,13 | 14 | 12 |
| Nantong022 | 19 | 14 | 19 | 31 | 15 | 10 | 23 | 11 | 12 | 10 | 14 | 18 | 21 | 22 | 11 | 14 | 10 | 13 | 20 | 11,14 | 17 | 12 |
| Nantong023 | 19 | 13 | 18 | 29 | 15 | 11 | 23 | 15 | 10 | 10 | 14 | 18 | 22 | 23 | 12 | 13 | 11 | 14 | 17 | 12,20 | 15 | 11 |
| Nantong024 | 17 | 12 | 19 | 28 | 16 | 10 | 24 | 12 | 11 | 10 | 14 | 17 | 20 | 24 | 11 | 14 | 11 | 13 | 15 | 13,14 | 15 | 11 |
| Nantong025 | 19 | 12 | 19 | 28 | 15 | 10 | 24 | 13 | 11 | 10 | 14 | 19 | 20 | 23 | 13 | 14 | 11 | 12 | 19 | 13,14 | 16 | 12 |
| Nantong026 | 17 | 13 | 21 | 29 | 16 | 10 | 25 | 14 | 12 | 10 | 14 | 17 | 22 | 23 | 11 | 11 | 9 | 14 | 16 | 11,20 | 15 | 12 |
| Nantong027 | 19 | 12 | 18 | 30 | 15 | 11 | 25 | 12 | 11 | 10 | 14 | 17 | 19 | 24 | 11 | 14 | 11 | 13 | 15 | 13,14 | 17 | 12 |
| Nantong028 | 19 | 12 | 20 | 28 | 14 | 10 | 23 | 13 | 12 | 11 | 15 | 21 | 20 | 24 | 12 | 14 | 11 | 12 | 17 | 14,18 | 15 | 12 |
| Nantong029 | 20 | 13 | 20 | 29 | 14 | 10 | 23 | 12 | 10 | 10 | 15 | 20 | 21 | 26 | 12 | 13 | 13 | 12 | 16 | 13,21 | 15 | 11 |
| Nantong030 | 17 | 12 | 20 | 29 | 16 | 10 | 26 | 14 | 10 | 10 | 14 | 18 | 21 | 25 | 12 | 13 | 10 | 12 | 17 | 14,18 | 14 | 12 |
| Nantong031 | 19 | 12 | 18 | 28 | 17 | 10 | 25 | 12 | 11 | 10 | 14 | 18 | 21 | 24 | 11 | 13 | 10 | 12 | 18 | 12,20 | 14 | 12 |
| Nantong032 | 17 | 12 | 18 | 28 | 15 | 11 | 25 | 12 | 11 | 10 | 14 | 19 | 19 | 23 | 11 | 14 | 11 | 13 | 15 | 13,13 | 17 | 12 |
| Nantong033 | 19 | 12 | 19 | 28 | 15 | 10 | 21 | 14 | 11 | 10 | 15 | 17 | 20 | 23 | 11 | 12 | 11 | 12 | 17 | 12,12 | 15 | 12 |
| Nantong034 | 19 | 12 | 20 | 29 | 16 | 10 | 24 | 12 | 11 | 10 | 15 | 16 | 22 | 23 | 10 | 13 | 12 | 12 | 18 | 13,18 | 14 | 11 |
| Nantong035 | 14 | 14 | 19 | 31 | 14 | 10 | 22 | 12 | 11 | 10 | 14 | 18 | 23 | 23 | 11 | 14 | 10 | 13 | 15 | 11,12 | 17 | 12 |
| Nantong036 | 19 | 13 | 19 | 30 | 15 | 10 | 21 | 12 | 11 | 10 | 14 | 16 | 20 | 23 | 12 | 12 | 11 | 12 | 20 | 12,16 | 17 | 12 |
| Nantong037 | 18 | 14 | 18 | 31 | 15 | 10 | 23 | 12 | 12 | 10 | 14 | 18 | 22 | 24 | 11 | 13 | 12 | 14 | 16 | 12,16 | 15 | 12 |
| Nantong038 | 17 | 12 | 18 | 28 | 15 | 10 | 24 | 12 | 11 | 10 | 14 | 19 | 19 | 23 | 12 | 14 | 11 | 14 | 15 | 13,13 | 19 | 12 |
| Nantong039 | 18 | 12 | 20 | 28 | 15 | 10 | 23 | 12 | 11 | 12 | 15 | 17 | 21 | 24 | 13 | 14 | 11 | 13 | 18 | 15,20 | 15 | 11 |
|  |  |  |  |  |  |  |  |  |  |  |  |  |  |  |  |  |  |  |  |  |  |  |
| Zhenjiang001 | 18 | 12 | 19 | 28 | 13 | 10 | 22 | 12 | 12 | 10 | 15 | 18 | 22 | 25 | 13 | 13 | 9 | 13 | 18 | 12,12 | 14 | 12 |
| Zhenjiang002 | 18 | 12 | 18 | 28 | 15 | 10 | 22 | 13 | 11 | 10 | 15 | 17 | 23 | 23 | 12 | 12 | 12 | 12 | 16 | 11,18 | 16 | 11 |
| Zhenjiang003 | 19 | 12 | 19 | 28 | 15 | 11 | 23 | 12 | 11 | 10 | 15 | 16 | 19 | 23 | 11 | 11 | 11 | 12 | 20 | 12,12 | 15 | 12 |
| Zhenjiang004 | 18 | 13 | 18 | 29 | 14 | 10 | 23 | 12 | 10 | 10 | 14 | 20 | 21 | 24 | 13 | 13 | 10 | 14 | 14 | 12,21 | 15 | 11 |
| Zhenjiang005 | 18 | 12 | 19 | 28 | 14 | 10 | 22 | 12 | 11 | 10 | 15 | 16 | 19 | 23 | 11 | 12 | 12 | 12 | 18 | 12,16 | 15 | 12 |
| Zhenjiang006 | 18 | 12 | 20 | 28 | 14 | 11 | 23 | 11 | 11 | 11 | 14 | 18 | 21 | 24 | 13 | 14 | 11 | 12 | 16 | 13,21 | 15 | 12 |
| Zhenjiang007 | 19 | 12 | 19 | 28 | 15 | 10 | 21 | 12 | 11 | 10 | 14 | 16 | 19 | 23 | 12 | 12 | 11 | 12 | 19 | 12,18 | 15 | 12 |
| Zhenjiang008 | 17 | 13 | 18 | 29 | 15 | 10 | 24 | 12 | 10 | 10 | 14 | 20 | 22 | 24 | 11 | 13 | 12 | 14 | 15 | 12,16 | 16 | 12 |
| Zhenjiang009 | 18 | 12 | 18 | 29 | 15 | 10 | 26 | 12 | 11 | 10 | 14 | 18 | 19 | 23 | 11 | 13 | 11 | 13 | 15 | 13,14 | 17 | 12 |
| Zhenjiang010 | 18 | 12 | 18 | 29 | 15 | 10 | 23 | 11 | 10 | 10 | 14 | 18 | 22 | 24 | 11 | 13 | 12 | 13 | 15 | 15,17 | 15 | 10 |
| Zhenjiang011 | 18 | 12 | 19 | 27 | 15 | 10 | 24 | 12 | 12 | 10 | 14 | 19 | 24 | 25 | 12 | 13 | 10 | 12 | 18 | 13,19 | 14 | 13 |
| Zhenjiang012 | 19 | 12 | 18 | 30 | 15 | 11 | 25 | 11 | 11 | 10 | 14 | 18 | 19 | 23 | 11 | 14 | 11 | 13 | 15 | 13,13 | 17 | 12 |
| Zhenjiang013 | 18 | 12 | 19 | 29 | 15 | 10 | 28 | 11 | 12 | 10 | 14 | 20 | 21 | 25 | 11 | 10 | 9 | 12 | 22 | 16,16 | 15 | 12 |
| Zhenjiang014 | 19 | 12 | 19 | 27 | 16 | 10 | 22 | 14 | 11 | 11 | 14 | 18 | 20 | 25 | 12 | 14 | 11 | 12 | 17 | 13,17 | 14 | 12 |
| Zhenjiang015 | 18 | 12 | 21 | 27 | 14 | 10 | 24 | 12 | 11 | 12 | 15 | 18 | 22 | 24 | 12 | 14 | 11 | 13 | 17 | 14,18 | 15 | 12 |
| Zhenjiang016 | 18 | 12 | 19 | 29 | 17 | 10 | 24 | 12 | 11 | 10 | 14 | 17 | 21 | 23 | 14 | 13 | 10 | 12 | 20 | 12,16 | 14 | 12 |
| Zhenjiang017 | 17 | 12 | 22 | 28 | 14 | 10 | 23 | 12 | 11 | 11 | 15 | 19 | 20 | 24 | 12 | 14 | 10 | 12 | 16 | 13,20 | 16 | 12 |
|  |  |  |  |  |  |  |  |  |  |  |  |  |  |  |  |  |  |  |  |  |  |  |
| Yangzhou001 | 19 | 14 | 18 | 30 | 15 | 11 | 24 | 13 | 11 | 10 | 14 | 19 | 21 | 24 | 11 | 13 | 11 | 14 | 14 | 12,17 | 16 | 12 |
| Yangzhou002 | 20 | 12 | 20 | 28 | 14 | 10 | 23 | 13 | 12 | 11 | 14 | 19 | 20 | 23 | 11 | 14 | 11 | 13 | 18 | 13,18 | 15 | 12 |
| Yangzhou003 | 16 | 13 | 20 | 31 | 15 | 10 | 25 | 12 | 12 | 11 | 14 | 16 | 21 | 23 | 12 | 11 | 9 | 14 | 16 | 11,18 | 15 | 12 |
| Yangzhou004 | 19 | 14 | 20 | 30 | 15 | 10 | 24 | 14 | 12 | 10 | 14 | 17 | 21 | 23 | 11 | 11 | 9 | 14 | 15 | 11,18 | 16 | 12 |
| Yangzhou005 | 19 | 12 | 20 | 29 | 15 | 10 | 24 | 13 | 12 | 10 | 14 | 17 | 20 | 23 | 13 | 13 | 11 | 13 | 17 | 12,20 | 15 | 12 |
| Yangzhou006 | 20 | 13 | 21 | 29 | 16 | 10 | 24 | 11 | 14 | 10 | 14 | 16 | 21 | 23 | 11 | 11 | 9 | 15 | 15 | 11,18 | 15 | 11 |
| Yangzhou007 | 18 | 14 | 18 | 31 | 15 | 10 | 19 | 10 | 10 | 10 | 14 | 17 | 21 | 23 | 11 | 15 | 10 | 15 | 17 | 12,19 | 15 | 12 |
| Yangzhou008 | 18 | 12 | 20 | 28 | 14 | 10 | 23 | 11 | 11 | 11 | 14 | 18 | 20 | 24 | 12 | 14 | 11 | 12 | 16 | 12,20 | 15 | 12 |
| Yangzhou009 | 16 | 12 | 18 | 29 | 17 | 10 | 25 | 12 | 12 | 10 | 14 | 19 | 22 | 23 | 13 | 14 | 11 | 13 | 15 | 14,14 | 15 | 12 |
| Yangzhou010 | 19 | 12 | 19 | 29 | 16 | 10 | 21 | 13 | 11 | 10 | 15 | 17 | 21 | 23 | 14 | 12 | 11 | 12 | 18 | 12,17 | 15 | 13 |
| Yangzhou011 | 18 | 12 | 18 | 29 | 16 | 11 | 25 | 12 | 11 | 10 | 14 | 18 | 19 | 23 | 11 | 14 | 11 | 13 | 15 | 13,13 | 16 | 13 |
| Yangzhou012 | 17 | 14 | 19 | 30 | 14 | 11 | 22 | 9 | 12 | 11 | 14 | 19 | 25 | 23 | 11 | 15 | 10 | 13 | 15 | 12,12 | 16 | 12 |
| Yangzhou013 | 20 | 12 | 18 | 27 | 16 | 11 | 23 | 13 | 11 | 10 | 14 | 18 | 21 | 23 | 13 | 14 | 11 | 13 | 14 | 13,13 | 16 | 12 |
| Yangzhou014 | 18 | 12 | 18 | 29 | 15 | 11 | 25 | 12 | 11 | 10 | 14 | 18 | 19 | 23 | 11 | 14 | 12 | 13 | 14 | 13,13 | 17 | 12 |
|  |  |  |  |  |  |  |  |  |  |  |  |  |  |  |  |  |  |  |  |  |  |  |
| Taizhou001 | 18 | 12 | 20 | 28 | 15 | 10 | 23 | 12 | 11 | 11 | 15 | 18 | 20 | 24 | 12 | 14 | 11 | 13 | 17 | 14,21 | 15 | 12 |
| Taizhou002 | 18 | 12 | 19 | 28 | 16 | 10 | 24 | 12 | 12 | 10 | 14 | 17 | 21 | 24 | 11 | 15 | 10 | 12 | 19 | 11,19 | 15 | 12 |
| Taizhou003 | 16 | 12 | 21 | 29 | 14 | 10 | 22 | 12 | 14 | 10 | 14 | 17 | 23 | 25 | 10 | 14 | 11 | 13 | 16 | 10,12 | 15 | 12 |
| Taizhou004 | 19 | 12 | 19 | 28 | 16 | 10 | 26 | 13 | 12 | 10 | 14 | 16 | 20 | 25 | 14 | 13 | 9 | 12 | 18 | 12,18 | 14 | 12 |
| Taizhou005 | 19 | 12 | 20 | 28 | 14 | 10 | 23 | 13 | 11 | 11 | 15 | 17 | 20 | 23 | 12 | 14 | 11 | 13 | 17 | 15,19 | 15 | 12 |
| Taizhou006 | 18 | 12 | 18 | 31 | 15 | 10 | 25 | 13 | 11 | 10 | 14 | 18 | 19 | 23 | 11 | 14 | 11 | 13 | 16 | 13,14 | 16 | 12 |
| Taizhou007 | 20 | 12 | 19 | 27 | 15 | 10 | 25 | 13 | 11 | 10 | 14 | 17 | 20 | 23 | 11 | 12 | 11 | 13 | 20 | 12,16 | 15 | 11 |

The value of "0" indicates the deletion of STR; Microvariants are labeled in bold form.
