# Supplementary material for: Inner and inter population structure construction of Chinese Jiangsu Han population based on Y23 STR system
Source: PLoS One. 2017 Jul 13;12(7):e0180921. doi: 10.1371/journal.pone.0180921 (PMC5509181; doi:10.1371/journal.pone.0180921)
Supplement: S3 Table — GD: Gene Diversity. (DOCX) [file pone.0180921.s003.docx]

**S3 Table. The distribution of allelic frequencies of 21 single-copy Y-STR loci in Jiangsu Han population (n=916).**

| Allele | DYS576 | DYS389I | DYS448 | DYS389II | DYS19 | DYS391 | DYS481 | DYS549 | DYS533 | DYS438 | DYS437 | DYS570 | DYS635 | DYS390 | DYS439 | DYS392 | DYS643 | DYS393 | DYS458 | DYS456 | YGATAH4 |
| --- | --- | --- | --- | --- | --- | --- | --- | --- | --- | --- | --- | --- | --- | --- | --- | --- | --- | --- | --- | --- | --- |
| 6 |  |  |  |  |  | 0.0044 |  |  |  |  |  |  |  |  |  |  |  |  |  |  |  |
| 7 |  |  |  |  |  | 0.0011 |  |  |  |  |  |  |  |  |  | 0.0011 |  |  |  |  |  |
| 8 |  |  |  |  |  | 0.0011 |  |  |  | 0.0033 |  |  |  |  |  |  | 0.0240 |  |  |  |  |
| 9 |  |  |  |  |  | 0.0306 |  | 0.0011 | 0.0011 | 0.0175 |  |  |  |  | 0.0044 |  | 0.0928 | 0.0011 |  |  | 0.0011 |
| 10 | 0.0011 |  |  |  |  | 0.7402 |  | 0.0044 | 0.0590 | 0.7533 |  |  |  |  | 0.0502 | 0.0109 | 0.2369 |  |  |  | 0.0437 |
| 11 |  | 0.0109 |  |  |  | 0.2183 |  | 0.0688 | 0.6146 | 0.1943 | 0.0011 |  |  |  | 0.4279 | 0.1070 | 0.5098 | 0.0022 |  |  | 0.2980 |
| 12 |  | 0.5819 |  |  |  | 0.0044 |  | 0.5459 | 0.2697 | 0.0273 |  |  |  |  | 0.3570 | 0.1703 | 0.1179 | 0.5251 | 0.0011 |  | 0.5731 |
| 13 |  | 0.2467 |  |  | 0.0448 |  |  | 0.3111 | 0.0426 | 0.0044 | 0.0087 |  |  |  | 0.1365 | 0.3264 | 0.0175 | 0.2893 | 0.0011 | 0.0218 | 0.0819 |
| 14 | 0.0142 | 0.1583 |  |  | 0.2118 |  |  | 0.0579 | 0.0131 |  | 0.6299 | 0.0120 |  |  | 0.0240 | 0.3461 | 0.0011 | 0.1266 | 0.0229 | 0.1692 | 0.0022 |
| 15 | 0.0120 | 0.0022 |  |  | 0.4607 |  |  | 0.0098 |  |  | 0.3439 | 0.0273 |  |  |  | 0.0371 |  | 0.0557 | 0.1889 | 0.5044 |  |
| 16 | 0.0710 |  | 0.0011 |  | 0.1965 |  |  | 0.0011 |  |  | 0.0164 | 0.1572 |  |  |  | 0.0011 |  |  | 0.1736 | 0.1900 |  |
| 16.1 |  |  |  |  |  |  |  |  |  |  |  |  |  |  |  |  |  |  | 0.0011 |  |  |
| 16.2 |  |  |  |  |  |  |  |  |  |  |  |  |  |  |  |  |  |  |  |  |  |
| 17 | 0.1932 |  | 0.0109 |  | 0.0852 |  |  |  |  |  |  | 0.2140 |  |  |  |  |  |  | 0.2489 | 0.0950 |  |
| 18 | 0.3199 |  | 0.2227 |  | 0.0011 |  | 0.0011 |  |  |  |  | 0.2806 | 0.0022 |  |  |  |  |  | 0.1932 | 0.0153 |  |
| 19 | 0.2456 |  | 0.3788 |  |  |  | 0.0011 |  |  |  |  | 0.1921 | 0.1692 |  |  |  |  |  | 0.0972 | 0.0044 |  |
| 19.2 |  |  | 0.0011 |  |  |  |  |  |  |  |  |  |  |  |  |  |  |  |  |  |  |
| 20 | 0.1059 |  | 0.2773 |  |  |  | 0.0011 |  |  |  |  | 0.0841 | 0.2413 | 0.0011 |  |  |  |  | 0.0480 |  |  |
| 21 | 0.0284 |  | 0.0928 |  |  |  | 0.0557 |  |  |  |  | 0.0229 | 0.3090 | 0.0033 |  |  |  |  | 0.0142 |  |  |
| 22 | 0.0076 |  | 0.0142 |  |  |  | 0.1670 |  |  |  |  | 0.0098 | 0.1703 | 0.0535 |  |  |  |  | 0.0066 |  |  |
| 23 | 0.0011 |  | 0.0011 |  |  |  | 0.2620 |  |  |  |  |  | 0.0753 | 0.4760 |  |  |  |  | 0.0033 |  |  |
| 24 |  |  |  |  |  |  | 0.2467 |  |  |  |  |  | 0.0284 | 0.2849 |  |  |  |  |  |  |  |
| 25 |  |  |  | 0.0011 |  |  | 0.1627 |  |  |  |  |  | 0.0033 | 0.1703 |  |  |  |  |  |  |  |
| 26 |  |  |  | 0.0087 |  |  | 0.0666 |  |  |  |  |  | 0.0011 | 0.0098 |  |  |  |  |  |  |  |
| 27 |  |  |  | 0.1004 |  |  | 0.0175 |  |  |  |  |  |  |  |  |  |  |  |  |  |  |
| 28 |  |  |  | 0.3319 |  |  | 0.0142 |  |  |  |  |  |  | 0.0011 |  |  |  |  |  |  |  |
| 29 |  |  |  | 0.2773 |  |  | 0.0044 |  |  |  |  |  |  |  |  |  |  |  |  |  |  |
| 30 |  |  |  | 0.1900 |  |  |  |  |  |  |  |  |  |  |  |  |  |  |  |  |  |
| 31 |  |  |  | 0.0775 |  |  |  |  |  |  |  |  |  |  |  |  |  |  |  |  |  |
| 32 |  |  |  | 0.0098 |  |  |  |  |  |  |  |  |  |  |  |  |  |  |  |  |  |
| 33 |  |  |  | 0.0033 |  |  |  |  |  |  |  |  |  |  |  |  |  |  |  |  |  |
|  |  |  |  |  |  |  |  |  |  |  |  |  |  |  |  |  |  |  |  |  |  |
| GD | 0.7834 | 0.5760 | 0.7219 | 0.7614 | 0.6958 | 0.4039 | 0.8089 | 0.5977 | 0.5447 | 0.3942 | 0.4851 | 0.8062 | 0.7831 | 0.6610 | 0.6684 | 0.7325 | 0.6613 | 0.6221 | 0.8233 | 0.6719 | 0.5747 |

GD: Gene Diversity.
