# Supplementary material for: Inner and inter population structure construction of Chinese Jiangsu Han population based on Y23 STR system
Source: PLoS One. 2017 Jul 13;12(7):e0180921. doi: 10.1371/journal.pone.0180921 (PMC5509181; doi:10.1371/journal.pone.0180921)
Supplement: S4 Table — GD: Gene Diversity. (DOCX) [file pone.0180921.s004.docx]

**S4 Table. The distribution of haplogroup frequencies of the multi-copy DYS385 locus in Jiangsu Han population (n=916).**

| Haplotype | Frequency |
| --- | --- |
| 9,12 | 0.0022 |
| 10,11 | 0.0011 |
| 10,12 | 0.0033 |
| 10,13 | 0.0011 |
| 10,15 | 0.0011 |
| 10,17 | 0.0011 |
| 10,18 | 0.0033 |
| 10,19 | 0.0033 |
| 10,20 | 0.0011 |
| 10,21 | 0.0011 |
| 11,11 | 0.0153 |
| 11,12 | 0.0415 |
| 11,13 | 0.0022 |
| 11,14 | 0.0076 |
| 11,15 | 0.0033 |
| 11,16 | 0.0087 |
| 11,16.2 | 0.0011 |
| 11,17 | 0.0186 |
| 11,18 | 0.0295 |
| 11,19 | 0.0186 |
| 11,20 | 0.0076 |
| 11,21 | 0.0044 |
| 11,22 | 0.0011 |
| 12,12 | 0.0349 |
| 12,13 | 0.0360 |
| 12,14 | 0.0055 |
| 12,15 | 0.0044 |
| 12,16 | 0.0557 |
| 12,17 | 0.0677 |
| 12,18 | 0.0524 |
| 12,19 | 0.0524 |
| 12,20 | 0.0295 |
| 12,21 | 0.0087 |
| 12,22 | 0.0044 |
| 13,13 | 0.0928 |
| 13,14 | 0.0306 |
| 13,15 | 0.0076 |
| 13,16 | 0.0109 |
| 13,17 | 0.0295 |
| 13,18 | 0.0491 |
| 13,19 | 0.0622 |
| 13,20 | 0.0251 |
| 13,21 | 0.0131 |
| 13,22 | 0.0011 |
| 13,23 | 0.0011 |
| 13,24 | 0.0011 |
| 13,26 | 0.0011 |
| 14,14 | 0.0022 |
| 14,15 | 0.0011 |
| 14,16 | 0.0022 |
| 14,17 | 0.0142 |
| 14,18 | 0.0328 |
| 14,19 | 0.0229 |
| 14,20 | 0.0066 |
| 14,21 | 0.0044 |
| 15,15 | 0.0011 |
| 15,16 | 0.0033 |
| 15,17 | 0.0044 |
| 15,18 | 0.0044 |
| 15,19 | 0.0087 |
| 15,20 | 0.0033 |
| 15,21 | 0.0033 |
| 15,22 | 0.0076 |
| 15,23 | 0.0011 |
| 16,16 | 0.0055 |
| 16,18 | 0.0011 |
| 16,19 | 0.0022 |
| 16,20 | 0.0022 |
| 16,21 | 0.0033 |
| 16,22 | 0.0011 |
| 17,17 | 0.0011 |
| 17,18 | 0.0011 |
| 17,19 | 0.0011 |
| 18,18 | 0.0011 |
| 19,19 | 0.0011 |
| 19,20 | 0.0011 |
| GD | 0.9607 |

GD: Gene Diversity.
